# Diterpenes Isolated from Three Different *Plectranthus Sensu Lato* Species and Their Antiproliferative Activities against Gynecological and Glioblastoma Cancer Cells

**DOI:** 10.1021/acsomega.4c00800

**Published:** 2024-04-08

**Authors:** Mária Gáborová, Máté Vágvölgyi, Bizhar Ahmed Tayeb, Renáta Minorics, István Zupkó, Ondřej Jurček, Szabolcs Béni, Renata Kubínová, György Tibor Balogh, Attila Hunyadi

**Affiliations:** † Department of Natural Drugs, Faculty of Pharmacy, Masaryk University, 612 00 Brno, Czechia; ‡ Institute of Pharmacognosy, Faculty of Pharmacy, University of Szeged, 6720 Szeged, Hungary; § Institute of Pharmacodynamics and Biopharmacy, Faculty of Pharmacy, University of Szeged, 6720 Szeged, Hungary; ∥ Department of Chemistry, Faculty of Science, Masaryk University, 625 00 Brno, Czechia; ⊥ National Center for Biomolecular Research, Faculty of Science, Masaryk University, 625 00 Brno, Czechia; # Department of Analytical Chemistry, Institute of Chemistry, Eötvös Loránd University, 1117 Budapest, Hungary; ¶ Department of Pharmacognosy, Semmelweis University, 1085 Budapest, Hungary; ∇ Department of Pharmaceutical Chemistry, Semmelweis University, 1092 Budapest, Hungary; ○ HUN-REN-SZTE Biologically Active Natural Products Research Group, 6720 Szeged, Hungary; ⧫ Interdisciplinary Centre of Natural Products, University of Szeged, 6720 Szeged, Hungary

## Abstract

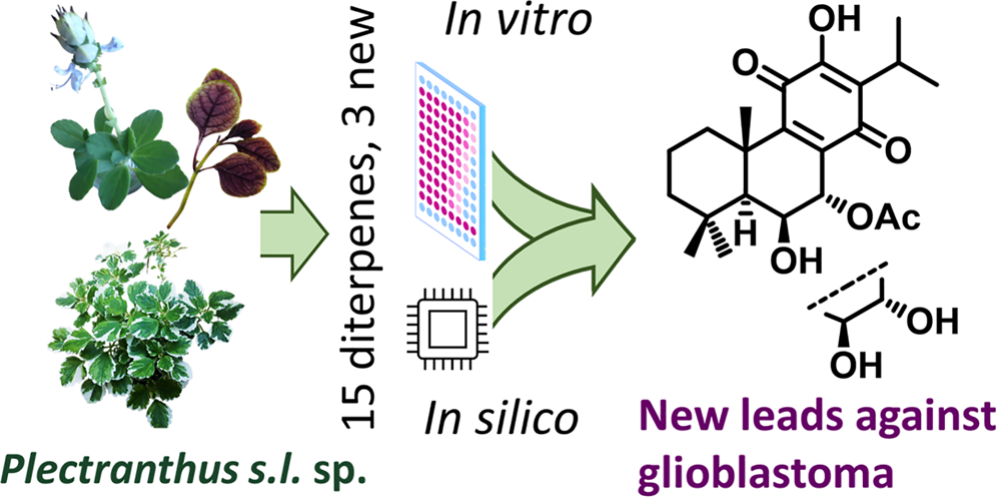

Fourteen diterpenes were isolated from methanol extracts of the aerial parts of*Coleus comosus*,*Coleus forsteri* “Marginatus”, and *Plectranthus ciliatus*. The compounds belong to the abietane (**1**–**4**, **9**–**11**, and **13**), *ent*-clerodane (**5**–**8**), and *ent*-kaurane (**14**, **15**) classes. Three new compounds were isolated from *C. comosus*, including 3-*O*-acetylornatin G (**2**), 3,12-di-*O*-acetylornatin G (**3**), ornatin B methyl ester (**5**), and ornatin F (**4**), for which we proposed a revised structure. The structures of the compounds were determined by comprehensive spectroscopic data analysis. The isolated diterpenes were examined in silico for their physicochemical and early ADME properties. Their antiproliferative effects were determined in vitro using human breast (MDA-MB-231 and MCF-7), cervical (HeLa), and glioblastoma (U-87 MG) cancer cell lines. The royleanone- and hydroquinone-type abietane diterpenes (**9**–**13**)exhibited the most potent antiproliferative activity against all cancer cell lines tested, particularly against glioblastoma cells, with IC_50_ values ranging from 1.1 to 15.6 μM.

Cancer is a leading cause of death worldwide, with almost 10 million deaths in 2020.^1^ Although many drugs were developed for their treatment, toxic side effects and acquired therapeutic resistance pose major limitations to their clinical use. Therefore, there is an urgent need for new treatments.^2^ Over 60% of the current antineoplastic drugs are derived from natural sources.^3^ Vinblastine, vincristine, paclitaxel, and the semisynthetic derivatives, etoposide and teniposide, are some outstanding examples of how plants represent an invaluable reservoir of active anticancer compounds.^2,4^

*Plectranthus sensu lato* (Lamiaceae), a large and widespread genus comprising three distinct genera [i.e., *Coleus* (294 species), *Plectranthus sensu stricto* (72 species), and *Equilabium* (42 species),^5^ has attracted interest because of its important role in traditional medicine. Many species have been used for centuries to treat various respiratory, digestive, genitourinary, and dermatological disorders.^6^ The anticancer potential of this genus has been extensively investigated. *Plectranthuss.l*. extracts and their diterpene constituents exert remarkable antiproliferative and cytotoxic activities on various cancer cell lines. Among the diterpenes, abietanes from the subclasses of royleanones (6,7-dehydroroyleanone and 7α-acetoxy-6β-hydroxyroyleanone), hydroquinones (coleon U), and quinone methides (parviflorone D) are the most promising.^7−9^ However, a recent study conducted by Ito and co-workers indicated that spirocoleon-type abietane diterpenes exhibit cytotoxicity against human breast (MCF-7), pancreatic (PSN-1), and cervical (HeLa) cancer cell lines with low toxicity against a normal lung fibroblast cell line (WI-38).^10^

*Coleus comosus* Hochst. ex Gürke (syn. *Plectranthus comosus*, *Plectranthus ornatus*)^5^ was reported to produce spirocoleon- and hydroquinone-type abietanes,^11,12^*ent*-clerodanes,^11−14^ labdanes,^12−16^ and halimanes.^16^*Coleus forsteri*“Marginatus” Benth. (syn. *Plectranthus forsteri* “Marginatus”)^5^ is a source of royleanone- and hydroquinone-type abietane diterpenes.^17−19^ To our knowledge, no diterpenes have been isolated from *Plectranthus ciliatus* E.Mey. (syn. *Plectranthus natalensis*).^5^ In a preliminary screen, however, the methanol extract of fresh leaves exhibited cytotoxic activity toward human drug-sensitive CCRF-CEM and multidrug-resistant CEM/ADR5000 leukemia cell lines.^20^ As a continuation of our previous study using this genus,^17,21−23^ the three above-mentioned *Plectranthuss.l*. species were selected to expand the available phytochemical data and to discover novel anticancer natural compounds. Fourteen diterpenes (**1**–**11, 13**–**15**) were isolated from *C. comosus*, *C. forsteri* “Marginatus”, and *P. ciliatus*. These compounds, along with 6,7-dehydroroyleanone (**12**) previously isolated by our group,^17^ were assessed for their drug-likeness based on the predicted physicochemical and early ADME parameters. Their antiproliferative activities were evaluated using MDA-MB-231 and MCF-7 human breast cancer cell lines, the HeLa human cervical cancer cell line, and the U-87 MG human glioblastoma cell line.

## Results and Discussion

Secondary metabolites isolated from *Plectranthuss.l*. species, particularly diterpenes and phenolic compounds, exhibited characteristic absorption patterns in the UV spectral region.^8^ Therefore, the chloroform-soluble phases prepared from the methanol extracts of *C. comosus*, *C. forsteri* “Marginatus”, and *P. ciliatus* were analyzed by high-performance liquid chromatography (HPLC)-DAD for fast screening of diterpene content. Preliminary HPLC-DAD analysis of *C. comosus* and *P. ciliatus* revealed the presence of constituents, whose UV spectra showed one absorption band with a maximum in the region 215–270 nm that could be attributed to different classes of diterpenes^13,24−26^ (Figures S1 and S3, Supporting Information). The chloroform-soluble phase of *C. comosus* was found to be a complex mixture with nine peaks between 11 and 31 min that were supposed to belong to diterpenes. On the other hand, the chloroform-soluble phases of *P. ciliatus* appeared as a simpler mixture, showing only two peaks (*t*_R_ = 19.007 and 21.538 min) attributable to diterpenes. The HPLC chromatogram of the chloroform-soluble phase of *C. forsteri* “Marginatus” (Figure S2, Supporting Information) contained two peaks (*t*_R_ = 20.206 and 24.081 min) with typical “royleanone-type” UV spectra,^27^ having an absorption band with a maximum at ∼270 nm and another broad band with a maximum at ∼400 nm. One peak (*t*_R_ = 29.832 min) possessed a “diosphenol-type” UV spectrum^28^ with four absorption bands characterized by maxima at ∼262, ∼ 286, ∼ 333, and ∼382 nm.

**Figure 1:**
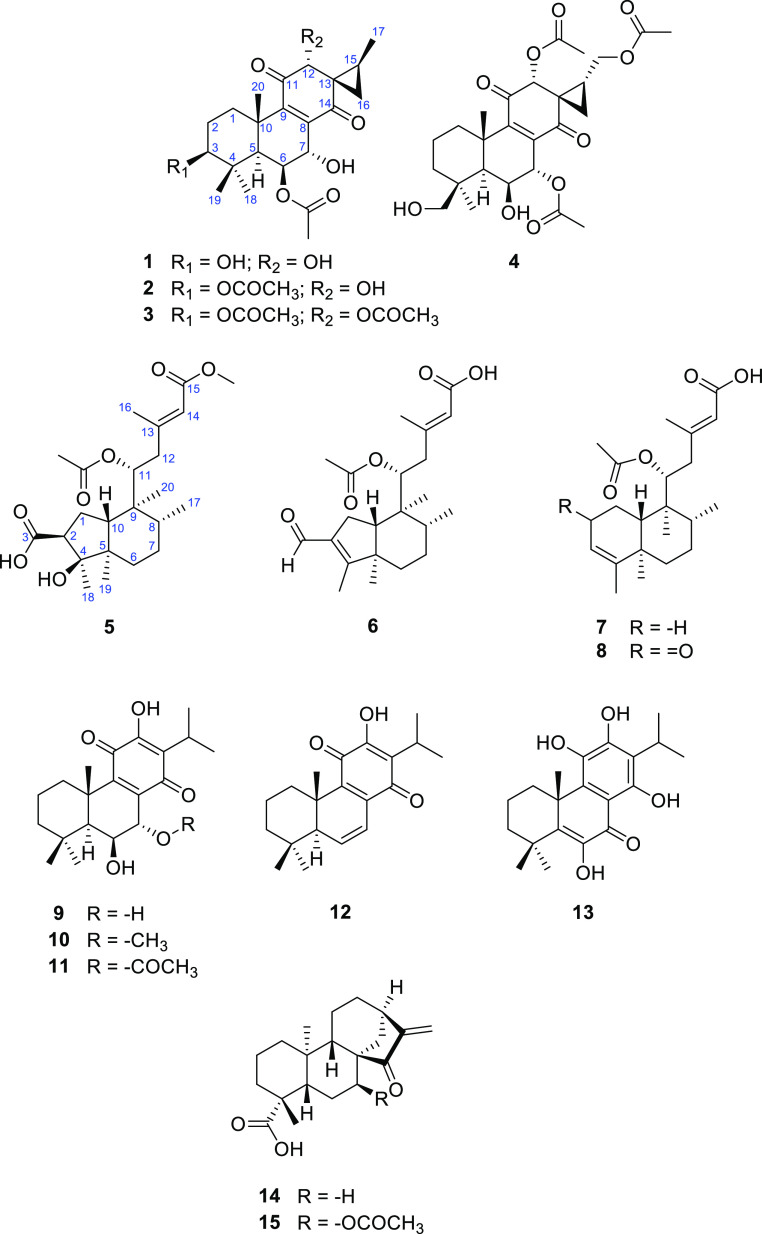
Structures of compounds **1**–**15**.

A dried methanol extract was prepared from the aerial parts of *C. comosus* and further subjected to solvent–solvent partitioning between chloroform and water. A diterpene-enriched, chloroform-soluble phase was further partitioned using 90% methanol and petroleum ether, which resulted in two phases (Experimental section). The methanol-soluble phase yielded four spirocoleon-type abietane diterpenes, including a known ornatin G (**1**),^11^ two new acetylated derivatives, 3-*O*-acetylornatin G (**2**) and 3,12-di-*O*-acetylornatin G (**3**), and ornatin F (**4**)^11^ whose structure was re-evaluated. The petroleum ether-soluble phase contained four *ent*-clerodane diterpenes: a new compound, ornatin B methyl ester (**5**), and three known compounds, ornatin D (**6**),^12^ 11R*-acetoxykolavenic acid (**7**),^14^ and 11R*-acetoxy-2-oxokolavenic acid (**8**).^14^ Four known abietane diterpenes, 6β,7α-dihydroxyroyleanone (**9**),^29,30^ 6β-hydroxy-7α-methoxyroyleanone (**10**),^31^ 7α-acetoxy-6β-hydroxyroyleanone (**11**),^29^ and coleon U (**13**),^18,32^ were isolated from *C. forsteri* “Marginatus”, whereas two known *ent*-kaurane diterpenes, *ent*-15-oxokaur-16-en-19-oic acid (**14**)^33^ and xylopinic acid (**15**),^34^ were isolated from *P. ciliatus*. These compounds were identified by spectroscopic data and comparison with the literature. The chemical structures of these isolated compounds are presented in Figure 1. A preliminary analysis of the NMR spectra indicated that the structures of compounds **2** and **3** were closely similar to ornatin G (**1**, C_22_H_30_O_7_).^11^ Therefore, the structure elucidation of **2** and **3** is discussed in relation to **1** as can be followed from Tables 1 and 2 containing ^1^H and ^13^C NMR data of **1**–**5**, respectively.

**Table 1:** ^1^H NMR Data for Compounds **1**–**5** (δ in ppm, *J* in Hz, Measured in CDCl_3_)

Position	**1**^a^	**2**^a^	**3**^a^	**4**^b^	**5**^a^
1	2.27, dt (13.1, 3.4)	2.23, dt (13.0, 3.0)	2.10, m	2.85, dt (13.2, 3.0)	2.12, m
	1.40, m	1.46 td (13.0, 5.0)	1.14, m	1.35, td (13.2, 4.1)	1.85, m
2	1.81, m	1.84, m	1.81, m	1.74, m	2.96, dd (11.2, 4.3)
				1.59, m	
3	3.32, dd (9.0, 7.2)	4.60, dd (10.9, 5.4)	4.53, m (ov)	1.54, br d (13.8)	
				1.23 td (13.8, 4.2)	
5	1.58, br s	1.70, br s	1.65, br s	1.52, s	
6	5.46, t (1.6)	5.44, br s	5.50, br t (1.4)	4.20, d (2.2)	1.61, m
					1.25, m
7	4.52, d (1.6)	4.53, br s	4.51, m (ov)	5.78, d (2.2)	1.46, m (ov)
8					1.45, m (ov)
10					2.28 m (ov)
11					5.08 dd (10.6, 1.5)
12	3.99, s	3.92, br s	4.89, s	5.67, s	2.44, d (13.0)
					2.27, m (ov)
14					5.66, br s
15	1.97, m	2.02, m	2.20, m	2.07, m	
16	1.40, dd (8.9, 4.0)	1.37, dd (8.9, 3.8)	1.33, dd (9.0, 4.2)	1.71, dd (6.9, 5.2)	2.18, d (1.1)
	1.01, dd (7.6, 4.0)	1.00, dd (7.1, 3.8)	1.05, m	1.15, dd (9.0, 5.2)	
17	1.27, d (6.4)	1.28, d (6.4)	1.13, d (6.4)	4.26, m (ov)	0.93, d (5.7)
				3.64, dd (11.9, 10.4)	
18	1.13, s	1.04, s	1.04, s	1.03, s	1.34, s
19	1.00, s	1.06, s	1.06, s	4.25, m (ov)	0.87, s
				3.35, d (11.0)	
20	1.65, s	1.67, s	1.71, s	1.58, s	0.81, s
3-OAc		2.07, s	2.08, s		
6- OAc	2.05, s	2.05, s	2.06, s		
7-OH		4.31, br s (ov)	2.71, d (3.2)		
7- OAc				1.99, s	
11-OAc					2.01, s
12-OH		4.29, br s (ov)			
12- OAc			2.13, s	2.17, s	
15-OCH_3_					3.67, s
17-OAc				1.93, s	

a Spectra taken at 400 MHz.

b Spectra taken at 700 MHz; (ov) Signals in the overlapped regions of the spectra and the multiplicities could not be recognized; Diastereotopic methylene protons are further discussed as “a” (deshielded ones) and “b” (shielded ones).

**Table 2:** ^13^C NMR Data for Compounds **1**–**5** (δ in ppm, Measured in CDCl_3_)

position	**1**^a^	**2**^a^	**3**^a^	**4**^b^	**5**^a^
1	35.3, CH_2_	34.7, CH_2_	35.2, CH_2_	38.5, CH_2_	25.6, CH_2_
2	27.5, CH_2_	23.9, CH_2_	23.9, CH_2_	18.8, CH_2_	50.8, CH
3	78.2, CH	79.6, CH	79.5, CH	38.6, CH_2_	178.7, C
4	39.2, C	38.1, C	38.3, C	38.8, C	82.4, C
5	46.9, CH	46.8, CH	47.2, CH	50.2, CH	49.3, C
6	70.9, CH	71.0, CH	70.7, CH	65.8, CH	29.8, CH_2_
7	65.1, CH	64.6, CH	65.2, CH	67.7, CH	28.8, CH_2_
8	140.3, C	139.7, C	140.7, C	139.7, C	36.8, CH
9	155.4, C	155.5, C	155.1, C	157.0, C	43.2, C
10	38.8, C	38.4, C	38.5, C	40.0, C	45.6, CH
11	198.6, C	198.3, C	194.3, C	194.5, C	75.9, CH
12	77.0, CH^c^	77.1, CH	78.2, CH	74.5, CH	42.1, CH_2_
13	36.9, C	36.8, C	34.9, C	34.8, C	157.1, C
14	197.2, C	196.6, C	196.3, C	192.6, C	118.2, CH
15	23.7, CH	22.9, CH	21.43, CH	23.3, CH	166.8, C
16	25.4, CH_2_	26.7, CH_2_	26.8, CH_2_	13.4, CH_2_	18.8, CH_3_
17	13.6, CH_3_	13.6, CH_3_	13.0, CH_3_	61.7, CH_2_	16.7, CH_3_
18	28.1, CH_3_	28.1, CH_3_	28.0, CH_3_	28.5, CH_3_	22.2, CH_3_
19	17.0, CH_3_	18.1, CH_3_	18.2, CH_3_	68.2, CH_2_	17.6, CH_3_
20	21.8, CH_3_	21.9, CH_3_	22.0, CH_3_	21.0, CH_3_	13.0, CH_3_
3-OAc		170.9, C	170.9, C		
		21.4, CH_3_	21.37, CH_3_		
6-OAc	170.2, C	170.3, C	170.0, C		
	21.6, CH_3_	21.6, CH_3_	21.6, CH_3_		
7-OAc				169.37, C^d^	
				20.9, CH_3_	
11-OAc					170.6, C
					21.1, CH_3_
12-OAc			169.9, C	169.35, C^d^	
			20.9, CH_3_	20.6, CH_3_	
15-OCH_3_					51.0, CH_3_
17-OAc				170.3, C	
				20.8, CH_3_	

a Spectra taken at 100 MHz.

b Spectra taken at 175 MHz.

c The signal appeared to overlap with the solvent signal and its chemical shift was deduced from HSQC.

d Carbonyl carbons indistinguishable in HMBC (the signals of 7-OAc and 12-OAc are interchangeable).

Compound **2** was isolated as a yellowish, amorphous solid. Its formula, C_24_H_32_O_8_, was determined using mass spectrometry detecting the protonated molecule at *m*/*z* 449.21691 [M + H]^+^ (calcd for C_24_H_33_O_8_, 449.21754), which accounted for nine indices of hydrogen deficiency. The ^1^H NMR spectrum exhibited signals for 32 protons. With the assistance of the HSQC spectrum, they were distinguished into six methyl, three methylene, and six methine protons, including four oxymethine protons (Table 1). The overlapping broad singlets at δ_H_ 4.31 and 4.29, which showed no correlation in the HSQC spectrum, were deduced to form part of the two hydroxyl residues. The APT spectrum exhibited 24 distinct carbon resonances (Table 2). Four carbonyl carbons (δ_C_ 198.3, 196.6, 170.9, and 170.3) and two nonprotonated sp^2^ carbons (δ_C_ 155.5 and 139.7) corresponding to a tetra-substituted double bond required four rings to satisfy the nine hydrogen deficiency indices. The characteristic signals attributed to a conjugated dicarbonyl moiety (δ_C_ 198.3, 196.6, 155.5, and 139.7) and a methylcyclopropane ring (δ_C_ 36.8, 26.7, 22.9, and 13.6) resembled a tetracyclic spirocoleon-type abietane skeleton.^11,35^ The ester carbonyl resonances (δ_C_ 170.9, and 170.3) with methyl signals (δ_C–H_ 21.6/2.05; 21.4/2.07) revealed two acetoxy groups. In contrast to **1**, there were additional signals for the methyl group (δ_C–H_ 21.4/2.07) and the carbonyl carbon (δ_C_ 170.9) as well as downfield shifted H-3 (δ_H_ 4.60, Δδ = +1.28), which together with the HMBC correlations of both methyl protons at δ_H_ 2.07 and H-3 with the carbonyl carbon at δ_C_ 170.9, indicated the occurrence of another acetoxy group at C-3 (Figure S7, Supporting Information).

To determine the relative configuration of **2**, an analysis of the coupling constants and NOE interactions was performed. The NOE correlations, H-5/H_3_-18 and H_3_-19/H_3_-20, and the lack of H-5/H_3_-20 correlation dictated the *trans*-annelation of rings A and B. Considering that only spirocoleons of the normal series are described in the literature,^9^ β-oriented Me-20 and α-oriented H-5 were suggested and selected as starting reference points. The NOE correlations, H-3/H-5, H-3/H_3_-18, H-5/H-6, H-5/H_3_-18, and H-6/H_3_-18, corroborated their cofacial α-orientation and indicated that both acetoxy groups (3-OAc and 6-OAc) were β-oriented. In addition, the large coupling constant between H-2_ax_ and H-3 (^3^*J*_H-2ax/3_ = 10.9 Hz) confirmed that H-3 occupied an α-axial position. H-7 was sited in a β-pseudoequatorial position because it was not correlated with H-5; thus, 7-OH was determined to be α-oriented. The NOE correlations, H-12/H-16b, H-12/H_3_-17, H-16b/H_3_-17, and H-15/H-16a, were consistent with an α-orientation of 12-OH^11^ and the *trans*-arrangement of Me-17 and C-14 carbonyl attached to the cyclopropyl ring (Figure S8, Supporting Information). Therefore, the structure of **2** was established and designated as 3-*O*-acetylornatin G (Figure 1).

Compound **3** appeared as a yellowish, amorphous powder. The HRESIMS ion at *m*/*z* 491.2277 [M + H]^+^ (calcd for C_26_H_35_O_9_, 491.22811) indicated the molecular formula C_26_H_34_O_9_, with ten indices of hydrogen deficiency. An assessment of the NMR spectra revealed that compound **3** is a derivative of **1** and contains the same skeleton (dicarbonyl moiety: δ_C_ 196.3, 194.3, 155.1, and 140.7; methylcyclopropane ring: δ_C_ 34.9, 26.8, 21.43 and 13.0), three acetoxy groups (δ_C_ 170.9, 21.37, δ_H_ 2.08; δ_C_ 170.0, 21.6, δ_H_ 2.06; δ_C_ 169.9, 20.9, δ_H_ 2.13), and one hydroxyl group (δ_H_ 2.71). The main differences between compounds **1** and **3** were the additional resonances of the methyl groups (δ_C–H_ 21.37/2.08 and 20.9/2.13) and the carbonyl carbons (δ_C_ 170.9 and 169.9) as well as downfield-shifted protons H-3 (δ_H_ 4.53, Δδ = +1.21) and H-12 (δ_H_ 4.89, Δδ = +0.90), which were evidence of two additional acetoxy groups at C-3 and C-12. These results were verified by the HMBC connectivities of the methyl protons at δ_H_ 2.08 and H-3, with the carbonyl carbon at δ_C_ 170.9 and the methyl protons at δ_H_ 2.13 and H-12 with the carbonyl carbon at δ_C_ 169.9 (Figure S7, Supporting Information). NOESY permitted the assignment of the same relative configuration for **3** as that of **1** and **2** (Figures S8, Supporting Information). Therefore, compound **3** was identified as 3,12-di-*O*-acetylornatin G with the structure shown in Figure 1.

Compound **4** was acquired as a white, amorphous solid. Its HRESIMS exhibited a protonated molecule at *m*/*z* 507.22272 [M + H]^+^ (calcd for C_26_H_35_O_10_, 507.22302), consistent with the molecular formula C_26_H_34_O_10_, suggesting ten indices of hydrogen deficiency. The NMR data revealed that the compound has a spirocoleon-type abietane skeleton (dicarbonyl moiety: δ_C_ 194.5, 192.6, 157.0, and 139.7; cyclopropane ring: δ_C_ 34.8, 23.3, and 13.4)^10,11^ with two hydroxy and three acetoxy groups (δ_C_ 170.3, 20.8, δ_H_ 1.93; δ_C_ 169.37, 20.9, δ_H_ 1.99; δ_C_ 169.35, 20.6, δ_H_ 2.17), and proposed the same planar structure as that reported for ornatin F;^11^ however, some assignments required revision. The NOE correlations, H-12/H-16b, H-15/H-16b, H-16a/H-17a, and H-16a/H-17b, implied a *cis* relationship between the 15-acetoxymethyl group and the carbonyl at C-14, as originally suggested.^11^ However, a correlation H-12/H_3_-20 and the absence of the previously reported correlation H-12/H-15^11^ placed H-12 in the β-pseudoaxial position (Figures S8, Supporting Information). In addition, the original assignment for CH_2_-1 and CH_2_-3^11^ was interchanged (Tables 1 and 2) based on the HMBC cross-peaks, H-1a/C-9, H-1a/C-20, H-1b/C-9, H-1b/C-20, H-3b/C-18, and H-3b/C-19 (Figures S7, Supporting Information). Thus, the revised structure of 7α,12α,17-triacetoxy-6β,19-dihydroxy-13β,16-spirocycloabiet-8-ene-11,14-dione was established for compound **4** (Figure 1).

Compound **5** was present as a white, amorphous powder. The peak of the protonated molecule at *m*/*z* 425.2561 [M + H]^+^ (calcd for C_23_H_37_O_7_, 425.25393) in the HRESIMS spectrum predicted the molecular formula C_23_H_36_O_7_ with six indices of hydrogen deficiency. Tandem analysis of the ^1^H NMR and HSQC spectra revealed the signals for 34 protons classified as seven methyl, four methylene, and five methine protons including one olefinic and one oxygenated methine proton (Table 1). The two remaining protons were likely part of the hydroxyl and carboxyl group. The APT spectrum exhibited 23 carbon resonances (Table 2). Three carbonyl carbons (δ_C_ 178.7, 170.6, 166.8) and a nonprotonated sp^2^ carbon (δ_C_ 157.1) along with an olefinic carbon (δ_C_ 118.2) indicated the presence of three carbonyl groups and one trisubstituted olefinic bond, respectively. To satisfy the six indices of hydrogen deficiency, a bicyclic ring system was proposed. In addition, the resonances assigned to an acetoxy group (δ_C_ 170.6, δ_C–H_ 21.1/2.01) and a methoxy group (δ_C–H_ 51.0/3.67) indicated that 20 carbon atoms were left for the skeleton suggesting a diterpene core for compound **5**. Compared with the published NMR data for the known compound ornatin B,^12^ only minor differences were observed. The presence of an additional methoxy group (δ_C–H_ 51.0/3.67) and its HMBC correlation with the carbonyl carbon C-15 (δ_C_ 166.8) indicated that compound **5** was a methyl ester of ornatin B (Figures S7, Supporting Information). The NOE cross-peaks revealed that this compound has the same relative configuration as ornatin B.^12^ A set of NOEs, H-1b/H_3_-19, H-1b/H_3_-20, H-2/H_3_-18, H-2/H_3_-19, H-6b/H_3_-19, and H_3_-18/H_3_-19, suggested that these protons are on the same side of the bicyclic ring core with α-orientation. Similarly, the correlation of H-10/H-6a indicated that these protons are located on the opposite side of the bicyclic diterpene skeleton; thus, they are β-oriented. The NOE correlation H-10/H-8 was decisive for determining Me-17 as α-oriented (Figure S8, Supporting Information). Only a configuration for the C-11 stereogenic center could not be assigned with the NOESY because of the free rotation ability of the side chain. However, based on previous reports^12−14^ on *ent*-clerodanes isolated from *C. comosus* substituted with the same C-9 side chain, the configuration of C-11 was proposed as R*. The *E*-configuration of the trisubstituted olefinic double bond Δ^13(14)^ was evident from the characteristic resonances of Me-16 (δ_H_ 2.18, δ_C_ 18.8),^13,14,36^ and the strong supportive NOE interaction H-12b/H-14.^16^ Compound **5** was identified as depicted (Figure 1) and designated as the ornatin B methyl ester.

Isolation of compounds **1**–**4** confirmed that spirocoleon-type abietane diterpenes esterified with acetic acid at different positions are the major secondary metabolites produced by *C. comosus*. A comparison of their ^13^C NMR spectroscopic data also suggests that chemical shift of C-16 may be considered diagnostic for determining the arrangement in ring C. While *trans*-spirocoleons **1**–**3** displayed the C-16-assigned signal at δ_C_ 25.4, 26.7, and 26.8, respectively, the corresponding signal of *cis*-spirocoleon **4** was observed at δ_C_ 13.4. This result is consistent with the data published for *cis*- and *trans*-spirocoleons with the absolute configuration (12*R*,13*S*,15*R*) and (12*R*,13*S*,15*S*), respectively, although their ^13^C NMR spectra were recorded in (CD_3_)_2_CO.^35^ Interestingly, *C. comosus* is the only known *Plectranthus* s.l. species that biosynthesizes *ent*-clerodane diterpenes having either a 6/6-fused *ent*-clerodane scaffold as present in the structures of **7** and **8** or a modified 5/6-fused (4 → 2)-*abeo*-*ent*-clerodane ring system present in **5** and **6**. All the *ent*-clerodanes isolated from *C. comosus* contain a C-9 side chain with an *E*-configured Δ^13(14)^ double bond and a 15-carboxyl group capable of forming methyl esters.^11−14^ While abietanes **9**–**13** have already been reported to be biosynthesized by *C. forsteri* “Marginatus”^17−19^ and are among the most widespread representatives of *Plectranthuss.l*. diterpenes,^9^*ent*-kauranes **14** and **15** were isolated only from *Plectranthus strigosus* Benth. ex E.Mey.^5,37^ Thus, their isolation from *P. ciliatus* was the first report of diterpenes in this species.

Fifteen diterpene compounds isolated from three different plants (*C. comosus*, *C. forsteri* “Marginatus”, and *P. ciliatus*) were subjected to in silico physicochemical and early ADME characterization (Table 3) and cell proliferation assays (Table 4). First, the compounds were evaluated based on the Lipinski rule of five (Ro5),^38,39^ which is associated with classical drug-likeness, and the central nervous system multiparameter optimization^40^ descriptor for BBB permeability. In addition, aqueous solubility, Caco-2 penetration, and Pgp efflux risk were also predicted as screening parameters using the ACD/Percepta software package.^41^

**Table 3:**
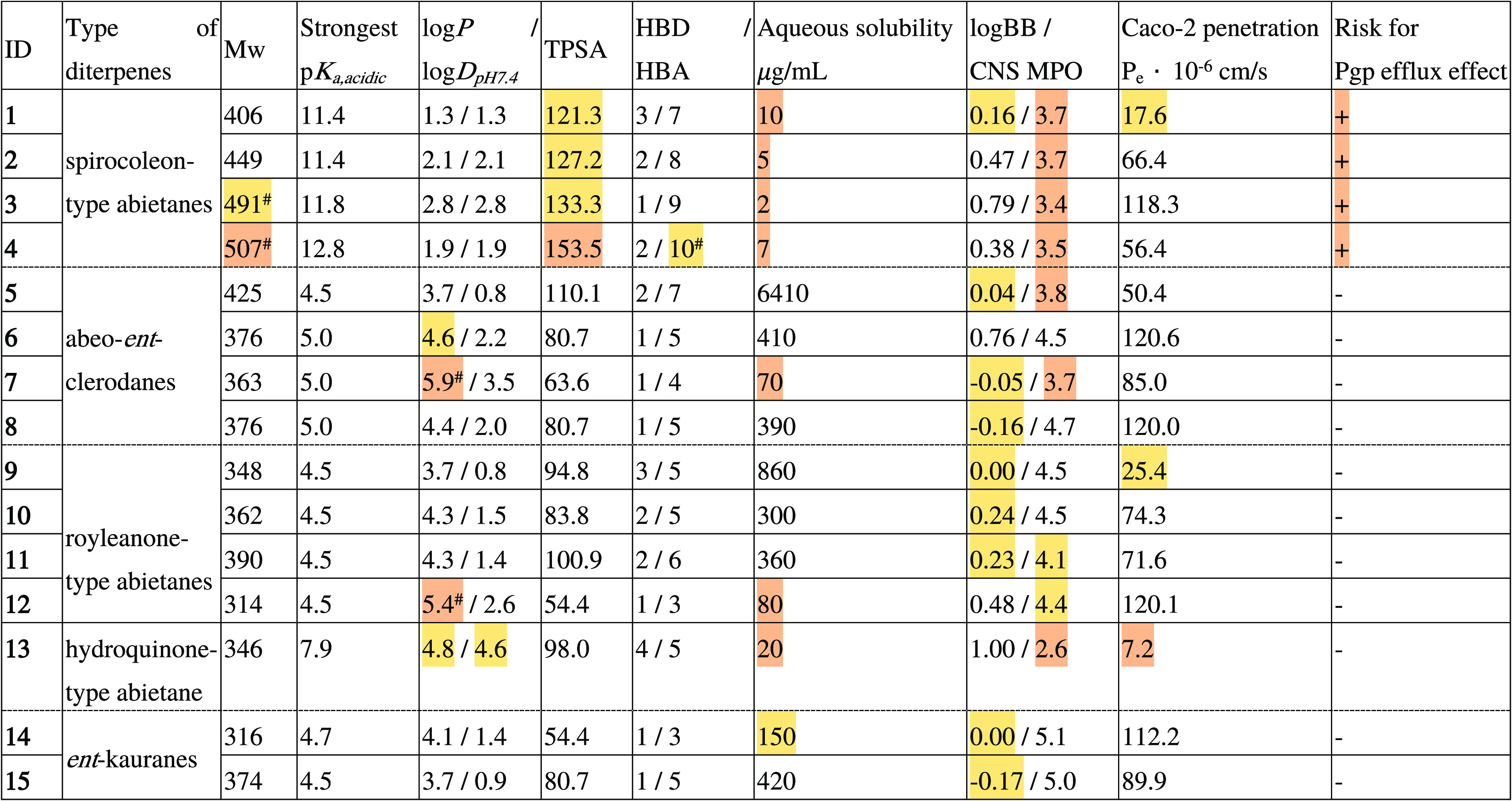
Predicted Physicochemical and Early ADME Parameters of Diterpene Compounds for Drug-Likeness Classification^a^

a Risk for drug- and CNS-agent-likeness (moderate—yellow, high—red).

b Lipinski Ro5 violation; HBD = hydrogen bond donor; HBA = hydrogen bond acceptor.

**Table 4:**
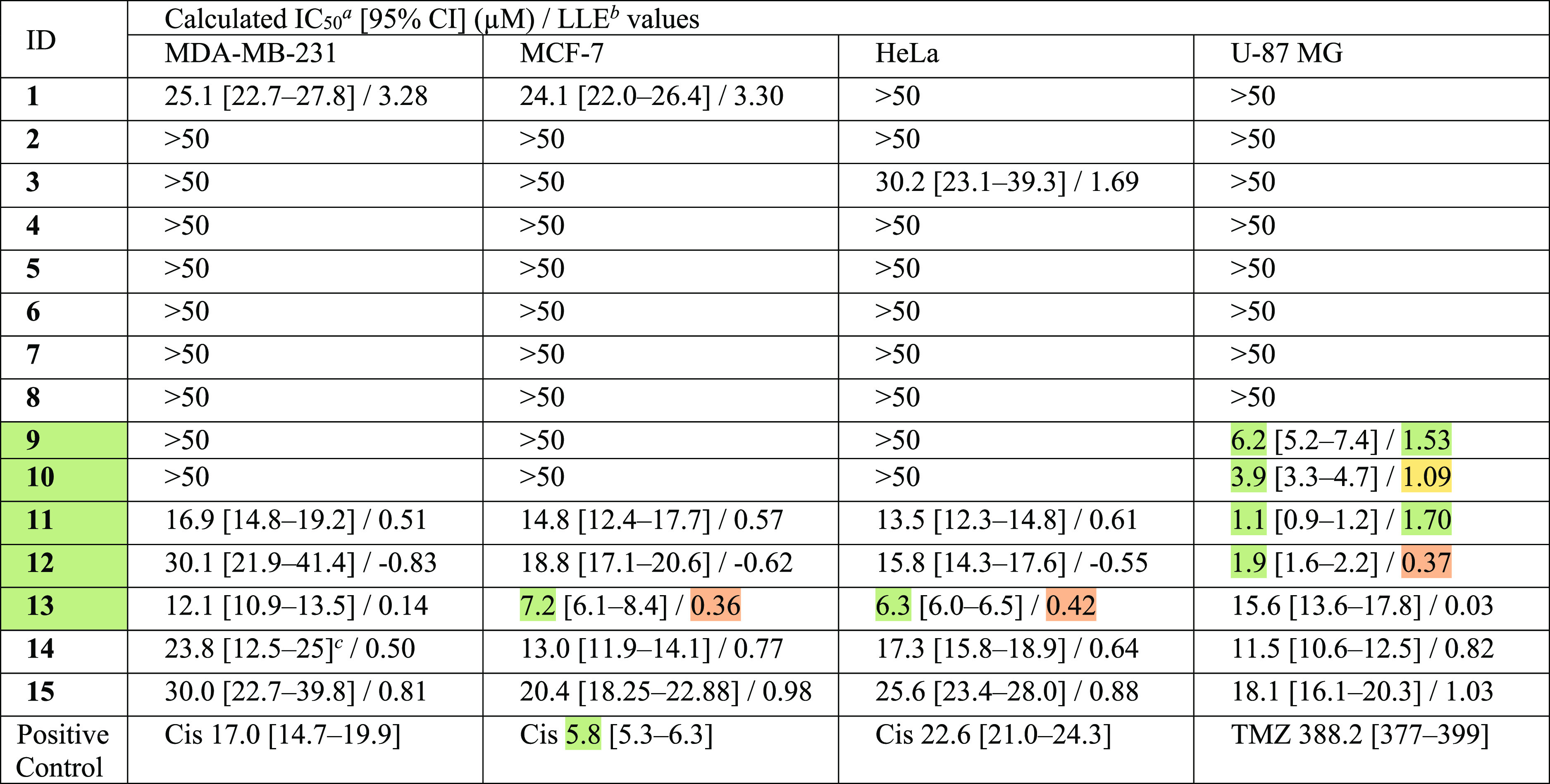
Antiproliferative Effect of the Isolated Diterpenes on Human Gynecological (MDA-MD-231, MCF-7, and HeLa) and Glioblastoma (U-87 MG) Cell Lines by MTT Assay for 72 h

a Limit for antiproliferative effect—IC_50_ < 10 μM (green values).

b Lipophilic ligand efficiency (LLE) parameters were calculated by the following equation: LLE = pIC_50_ – log *P* (using Table 3. data). Classification system for LLE: green (lower promiscuity risk): IC_50_ < 10 μM and LLE ≥ 1.5, yellow (moderate promiscuity risk): IC50 < 10 μM and 1.5 > LLE ≥ 1, red (higher promiscuity risk): IC_50_ < 10 μM and 1.0 > LLE. Values not marked with a color do not fulfill any of the classification criteria.

c Ambiguous fitting, confidence interval cannot be calculated due to the high slope of the regression curve, the provided values represent experimental dilutions below and above the IC_50_; growth inhibitory values were −3.7 and 63.7% at 12.5 and 25 μM, respectively. Cis = Cisplatin, TMZ = Temozolomide.

Based on the physicochemical data, compounds **5** and **12** did not conform to Ro5 because of their high lipophilicity (log *P*: 5.9 and 5.4, respectively) and the relatively high molecular weight of compound **4** (*M*_w_: 507). However, an increased log *D*_pH7.4_ value was also assigned to **13** because of its reduced acidic character (p*K*_a,acid_: 7.9, log *D*_pH7.4_:4.6). For the spirocoleon-type abietanes **1**–**4**, increased topological polar surface area (TPSA) values were identified, indicating a lower predicted bioavailability using the Egan filter.^42^ The increased polarity associated with TPSA for these compounds and their low predicted aqueous solubility (≤10 μg/mL) may be contradictory, but the predicted poor solubility may be explained by a rigid close-to-planar structure of spirocoleon-type abietanes. Combining log BB (≥−0.2) as a pharmacokinetic parameter and Wager’s optimization (CNS MPO ≥ 4.5) for distribution in the CNS, *ent*-clerodanes **6**, **8**, abietanes **9**–**10**, and *ent*-kauranes **14**–**15** are suitable candidates. An increased risk related to intestinal absorption was identified for compound **13** (Caco-2 – *P*_e_ × 10^–6^ cm/s < 10). For compounds **1**–**4**, the Pgp substrate positivity indicated by the ADME predictor should also be emphasized, which suggests an elevated risk for efflux transport and related pharmacokinetic consequences. This is consistent with previous studies reporting the alteration of Pgp function by abietane diterpenes.^23,43,44^

The antiproliferative effect of the isolated diterpenes was evaluated against selected human gynecological (MDA-MB-231, MCF-7, and HeLa) and glioblastoma (U-87 MG) cancer cell lines (Table 4). Spirocoleon-type abietanes (**1**–**4**) were weak or inactive as antiproliferative agents against these cells. Ornatin G and F (**1** and **4**) are already known diterpenes, but to our knowledge, their antiproliferative effects have not been determined. Compounds **5**–**8** with an *ent*-clerodane skeleton did not show any effect on any of the cell lines at the tested concentrations. This is consistent with previous reports for compounds **6** and **8**, which exerted cytotoxicity neither on soft tissues nor on leukemia cancer cells.^12^ The isopropyl-substituted royleanone- and hydroquinone-type abietanes **9**–**13** exhibited the highest antiproliferative activity. These compounds (**9**–**13)** were previously isolated from various*Plectranthus* species,^8,45,46^ and their antiproliferative effects were observed to be lower than the screening criterion of IC_50_ ≤ 10 μM in these cell lines. Finally, *ent*-kaurane diterpenes (**14**–**15**) exhibited moderate antiproliferative effects on both gynecological and glioblastoma cells.

In general, isopropyl-substituted abietanes from the royleanone and hydroquinone subclasses exhibited selective and significantly higher antiproliferative effects against glioblastoma compared with those of the gynecological cancer cell lines. Thus, while compounds **9**–**10** were not effective against gynecological cancer cells and IC_50_ values of compounds **11**–**12** ranged from 13.5 to 30.1 μM, all four abietanes (**9**–**12**) were effective against glioblastoma cell lines with IC_50_ values below 10 μM. The IC_50_ values of compound **13** were also below 10 μM for HeLa and MCF-7 cancer cell lines. The results of compound **13** against MCF-7 were consistent with those of previous reports.^8,45^ Royleanone diterpenes (**9**–**12**) containing a *p*-benzoquinoid C-ring exhibited stronger antiproliferative effects on glioblastoma compared with hydroquinone coleon U (**13**) containing an aromatic C-ring. Therefore, the *p*-benzoquinone moiety of the isopropyl-substituted abietanes is important for their antiproliferative activity. Previously, the antiproliferative effect of compound **11** was determined using another CNS tumor cell line, SF-268.^45^ It showed a potent activity with IC_50_ values of 8.6 μM. Another abietane diterpene, compound **9**, exhibited a 1 order of magnitude weaker effect on cell proliferation. This suggests that the higher lipophilicity of **11** may facilitate cell membrane penetration. Based on our results, we made a similar observation given that compound **11** demonstrated markedly higher antiproliferative activity against all of the cell lines compared with compound **9**.

To select the most promising lead compound(s) and reduce the inherent risk of promiscuity of anticancer agents, we calculated the respective lipophilic ligand efficiency (LLE = pIC_50_ – log *P*) values.^47,48^ Adhering to the double criteria, IC_50_ ≤ 10 μM and LLE ≥ 1.5, compounds **9** and **11** were identified as primary candidates against the U-87 MG human glioblastoma cell line. These compounds were predicted to have the lowest off-target or toxicological risk. With a slightly higher risk of promiscuity, compound **10** may be considered to be a secondary hit. Although hydroquinone coleon U (**13**) did not meet our LLE criterion because of its marginally increased lipophilicity, it may be considered the most effective member of the isopropyl-substituted abietanes against gynecological cancer cells, as it exhibited efficacy greater than or equal to that of the positive control cisplatin.

In conclusion, the isolation of diterpenes **1**–**15** provides further insight into the phytochemistry of the genus *Plectranthuss.l*. and confirms that it is a rich source of structurally diverse diterpenes. The promising antiproliferative activity, particularly against glioblastoma cells, suggests that these compounds are potential anticancer leads worthy of further investigation.

## Experimental Section

### General Experimental Procedures

Optical rotations were measured in CHCl_3_ using an AUTOPOL IV polarimeter (Rudolph Research Analytical, Hackettstown, NJ, USA) with a 0.8 mL polarimetric cell at 23 °C (instrument room temperature). 1D and 2D NMR data were acquired with a JEOL ECZR 400 MHz NMR spectrometer (JEOL, Tokyo, Japan) operating at 400 MHz for ^1^H and 100 MHz for ^13^C, a Bruker AVANCE DRX 500 MHz spectrometer (Bruker, Billerica, MA, USA) at 500 MHz for ^1^H and 125 MHz for ^13^C, and a Bruker AVANCE III HD 700 MHz spectrometer (Bruker, Billerica, MA, USA) at 700 MHz for ^1^H and 175 MHz for ^13^C. The spectra were recorded in CDCl_3_ and signals for the residual solvent (δ_H_ 7.26 ppm; δ_C_ 77.16 ppm) were used as reference points. HRESIMS analyses were carried out using an LTQ Orbitrap XL mass spectrometer (Thermo Fisher Scientific, San Jose, CA, USA) by direct sample injection and on an Agilent 1100 LC–MS instrument (Agilent Technologies, Santa Clara, CA, USA) coupled with a Thermo Q-Exactive Plus Orbitrap analyzer (Thermo Fisher Scientific, Waltham, MA, USA) operating in positive and negative ionization modes. Column chromatography was performed using Silica gel 60, particle size 40–63 μm, and a 230–400 mesh particle size (Sigma-Aldrich, St. Louis, MO, USA). The fractions obtained by column chromatography were monitored by thin layer chromatography with precoated aluminum TLC plates Silica gel 60 F_254_, 20 × 20 cm, 200 μm (Merck KGaA, Darmstadt, Germany) at different ratios of CHCl_3_–EtOAc (15:1–1:1, v/v) as the mobile phase. The compounds were visualized by irradiation with UV light at 254 and 366 nm. Flash chromatography was performed using a Combiflash Rf+ instrument (Teledyne ISCO, Lincoln, NE, USA) equipped with a diode array and evaporative light scattering detectors. The apparatus was used with columns manually filled with MP EcoChromTM Polyamide, particle size 50–160 μm (MP Biomedicals Germany, GmbH, Eschwege, Germany). Analytical-scale, reversed-phase HPLC measurements were performed using two HPLC instruments. An Agilent 1100 HPLC instrument equipped with an Agilent 1100 Series diode array detector (Agilent Technologies, Santa Clara, CA, USA) was used with an analytical HPLC column Ascentis Express RP-Amide, 100 mm × 2.1 mm, particle size 2.7 μm (Sigma-Aldrich, St. Louis, MO, USA) heated to 40 °C. Mobile phases CH_3_CN–0.2% HCOOH (10–100% CH_3_CN in 36 min) or CH_3_OH–0.2% HCOOH (40–90% CH_3_OH in 36 min) with a constant flow rate of 0.3 mL/min were used. A Jasco HPLC instrument (Jasco International Co., Ltd., Hachioji, Tokyo, Japan) equipped with an “MD-2010 Plus” PDA detector was used with Kinetex XB-C18 250 × 4.6 mm, particle size 5 μm (Phenomenex, Torrance, CA, USA) or Gemini NX-C18, 250 × 4.6 mm, particle size 5 μm (Phenomenex, Torrance, CA, USA) analytical columns and mobile phases CH_3_CN–H_2_O (10–100% CH_3_CN in 40 min or 30–100% in 40 min) with a flow rate of 1 mL/min. Compound purities were assessed from the peak area percentage data of the chromatograms recorded at 215, 254, 280, and 350 nm (Agilent HPLC instrument) and between 200 and 400 nm (Jasco HPLC instrument). For the semipreparative reverse-phase HPLC, an Agilent 1100 HPLC instrument equipped with an Agilent 1100 Series diode array detector (Agilent Technologies, Santa Clara, CA, USA) or a Dionex UltiMate 3000 instrument (Thermo Scientific, Waltham, MA, USA) with an UltiMate 3000 Collector was used. The separations were performed with a semipreparative HPLC column Ascentis RP-Amide, 250 mm × 10 mm, particle size 5 μm (Sigma-Aldrich, St. Louis, MO, USA) heated to 40 °C. Preparative reverse-phase HPLC was performed on an Armen Spot Prep II HPLC purification system (Gilson, Middleton, WI, USA) with a dual-wavelength UV–Vis detector, employing the following preparative HPLC columns: Kinetex XB-C18, 250 mm × 21.2 mm, particle size 5 μm (Phenomenex, Torrance, CA, USA) or Gemini NX-C18 column 250 mm × 21.2 mm, particle size 5 μm (Phenomenex, Torrance, CA, USA). Deionized water was prepared using Milli-Q Direct and Direct Q-3 UV Water Purification Systems (Millipore, Billerica, MA, USA).

### Plant Material

*Plectranthuss.l*. species were cultivated in a greenhouse at the Faculty of Pharmacy, Masaryk University, Brno, Czechia. The aerial parts of *C. comosus* were collected in September 2018 and the aerial parts of *C. forsteri* “Marginatus” and *P. ciliatus* were harvested in August 2019. The fresh plant material was frozen and stored at −20 °C until extraction. The dried voucher specimens of *C. comosus*, *C. forsteri* “Marginatus” and *P. ciliatus* were deposited under the names PN 2018, PFM 2019, and PC 2019, respectively, in the herbarium of the Department of Natural Drugs, Faculty of Pharmacy, Masaryk University, Brno, Czech Republic.

### Extraction and Isolation

The starting plant material was extracted with methanol and subsequently solvent-partitioned. Diterpene-enriched phases were subjected to extensive multistep chromatographic procedures to obtain compounds **1**–**11** and **13**–**15**; the isolation procedures are described in detail in the Supporting Information.

3-*O*-Acetylornatin G (**2**): yellowish amorphous solid; [α]_D_^23^ + 19.5 (1.21, CHCl_3_); ^1^H and ^13^C NMR, see Tables 1 and 2; HRESIMS *m*/*z* 449.21691 [M + H]^+^ (calcd for C_24_H_33_O_8_, 449.21754).

3,12-Di-*O*-acetylornatin G (**3**): yellowish amorphous powder; [α]_D_^23^ + 79.3 (0.85, CHCl_3_); ^1^H and ^13^C NMR, see Tables 1 and 2; HRESIMS *m*/*z* 491.227 [M + H]^+^ (calcd for C_26_H_35_O_9_, 489.21246).

Ornatin F (**4**): white amorphous solid; [α]_D_^23^ + 92.8 (0.51, CHCl_3_); ^1^H and ^13^C NMR, see Tables 1 and 2; HRESIMS *m*/*z* 507.22272 [M + H]^+^ (calcd for C_26_H_35_O_10_, 507.22302).

Ornatin B methyl ester (**5**): white amorphous powder; [α]_D_^23^ – 8.4 (0.63, CHCl_3_); ^1^H and ^13^C NMR, see Tables 1 and 2; HRESIMS *m*/*z* 425.2561 [M + H]^+^ (calcd for C_23_H_37_O_7_, 425.25393).

### Cell Culture

Human gynecological cancer cell lines (MDA-MB-231, MCF-7, and HeLa) and the human glioblastoma U-87 MG cell line were purchased from the ECACC (European Collection of Cell Cultures, Salisbury, UK). Cells were grown in Eagle’s Modified Essential Medium supplemented with 10% heat-inactivated fetal bovine serum, 1% nonessential amino acids, and 1% penicillin–streptomycin–amphotericin B mixture in a humidified atmosphere containing 5% CO_2_ at 37 °C. To maintain the U-87 MG cell line, the basic medium was supplemented with 1% l-glutamine and 1% sodium pyruvate. All media and supplements were obtained from Capricorn Scientific Ltd. (Ebsdorfergrund, Germany). Cells in the near-confluent phase of growth were used for the assays described below.

### Antiproliferative Assay

The inhibitory effect of the 15 isolated compounds on cell proliferation was characterized by performing colorimetric MTT (3-(4,5-dimethylthiazol-2-yl)-2,5-diphenyltetrazolium bromide) assay.^49^ Briefly, 5 × 10^3^ cells per well were seeded in 96-well plates. After overnight incubation, the cells were treated with eight different concentrations of the compounds ranging from 50 to 0.39 μM. After 72 h of incubation, the cells were treated with MTT solution and incubated as recommended. The supernatant was removed from the wells, and the formazan crystals were dissolved in 100 μL dimethyl sulfoxide. The absorbance values at 545 nm for each sample were determined using a microplate UV–Vis reader (SPECTROstar Nano, BMG Labtech GmbH, Offenburg, Germany). At least two independent experiments were performed in triplicate. The resulting data were transformed into inhibitory percentages, which were used for the determination of IC_50_ values of the tested compounds using GraphPad Prism 9.5.1. software (GraphPad Software, San Diego, CA, USA). 95% confidence intervals (95% CI) for the IC_50_ values were also calculated to demonstrate the reliability of the experiments.

## Data Availability

The raw NMR spectra for compounds **2–5** are freely available on Zenodo with DOI: 10.5281/zenodo.10531934.

## Supporting Information Available

Detailed information on the screening of chloroform-soluble phases of *C. comosus*, *C. forsteri* “Marginatus”, and *P. ciliatus* for diterpene content, detailed information on extraction of plant material and isolation of compounds **1**–**11**, **13**–**15**, key HMBC and NOESY correlations of compounds **2**–**5**, and HRESIMS and 1D and 2D NMR spectra of compounds **1**–**11**, **13**–**15** (PDF)
