# Supplementary material for: Diterpenes Isolated from Three Different Plectranthus Sensu Lato Species and Their Antiproliferative Activities against Gynecological and Glioblastoma Cancer Cells
Source: ACS Omega. 2024 Apr 8;9(16):18495–504. doi: 10.1021/acsomega.4c00800 (PMC11044216; doi:10.1021/acsomega.4c00800)
Supplement: Supplementary file 1 — ao4c00800_si_001.pdf [file ao4c00800_si_001.pdf]

SUPPORTING INFORMATION

Diterpenes isolated from three different *Plectranthus sensu lato* species and their antiproliferative activities against gynecological and glioblastoma cancer cells

Mária Gáborová,‡[1] *Máté Vágvölgyi,*‡[2] *Bizhar Ahmed Tayeb,*[3] *Renáta Minorics,*[3] *István Zupkó,*[3] *Ondřej Jurček,*[1,4,5] *Szabolcs Béni,*[6,7] *Renata Kubínová,*[1] *György Tibor Balogh,* *[8] and *Attila Hunyadi*[*,2,9,10]

[1] Department of Natural Drugs, Faculty of Pharmacy, Masaryk University, 612 00 Brno, Czechia

[2] Institute of Pharmacognosy, Faculty of Pharmacy, University of Szeged, 6720 Szeged, Hungary

[3] Institute of Pharmacodynamics and Biopharmacy, Faculty of Pharmacy, University of Szeged, 6720 Szeged, Hungary

[4] Department of Chemistry, Faculty of Science, Masaryk University, 625 00 Brno, Czechia

[5] National Center for Biomolecular Research, Faculty of Science, Masaryk University, 625 00 Brno, Czechia

[6] Department of Analytical Chemistry, Institute of Chemistry, Eötvös Loránd University, 1117 Budapest, Hungary

[7] Department of Pharmacognosy, Semmelweis University, 1085 Budapest, Hungary

[8] Department of Pharmaceutical Chemistry, Semmelweis University, 1092 Budapest, Hungary

[9] HUN-REN-SZTE Biologically Active Natural Products Research Group, 6720 Szeged, Hungary

[10] Interdisciplinary Centre of Natural Products, University of Szeged, Szeged, Hungary

Email: balogh.gyorgy.tibor@semmelweis.hu, hunyadi.attila@szte.hu

Table of Contents

1. Screening of plant material for diterpene content 3
2. Extraction and isolation procedures 5
3. ^{1}H–^{1}H COSY and key HMBC correlations of compounds of compounds 2–5 11
4. Key NOESY correlations of compounds 2–5..........12
5. MS and NMR data for compound 1 13
6. MS and NMR data for compound 2 20
7. MS and NMR data for compound 3 29
8. MS and NMR data for compound 4 41
9. MS and NMR data for compound 5 48
10. MS and NMR data for compound 6 55
11. MS and NMR data for compound 7 63
12. MS and NMR data for compound 8 71
13. MS and NMR data for compound 9 79
14. MS and NMR data for compound 10 86
15. MS and NMR data for compound 11 93
16. MS and NMR data for compound 13 102
17. MS and NMR data for compound 14 109
18. MS and NMR data for compound 15 116

The raw NMR spectra for compounds **2–5** are freely available on Zenodo as DOI: 10.5281/zenodo.10531934.

1. Screening of plant material for diterpene content

HPLC-DAD analysis was carried out using Agilent 1100 HPLC instrument equipped with an Agilent 1100 Series diode array detector (Agilent Technologies, Santa Clara, CA, USA). An analytical HPLC column Ascentis Express RP-Amide, 100 mm × 2.1 mm, particle size 2.7 μm (Sigma-Aldrich, St. Louis, MO, USA) heated to 40°C and a mobile phase CH_3CN–0.2% HCOOH (10–100% CH_3CN in 36 min) were used. Detection by diode array was performed at 215, 254, 280, and 350 nm.

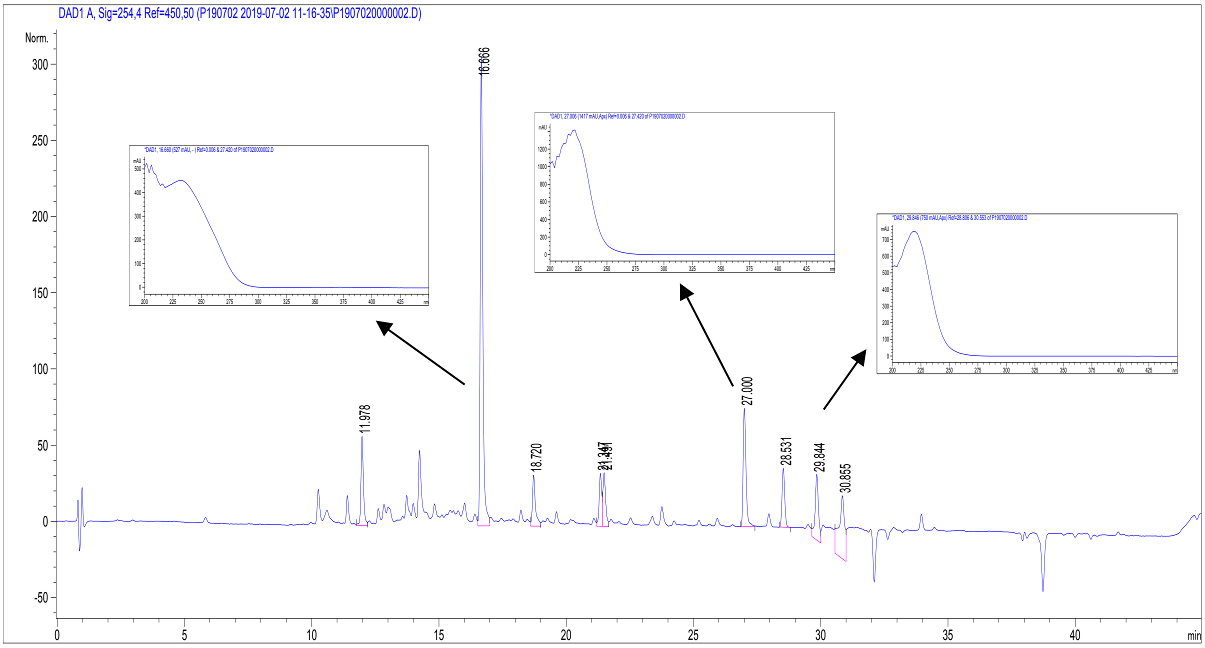

Figure S1. HPLC chromatogram of chloroform-soluble phase of *C. comosus* at 254 nm with selected UV spectra (only peaks of diterpenes are integrated).

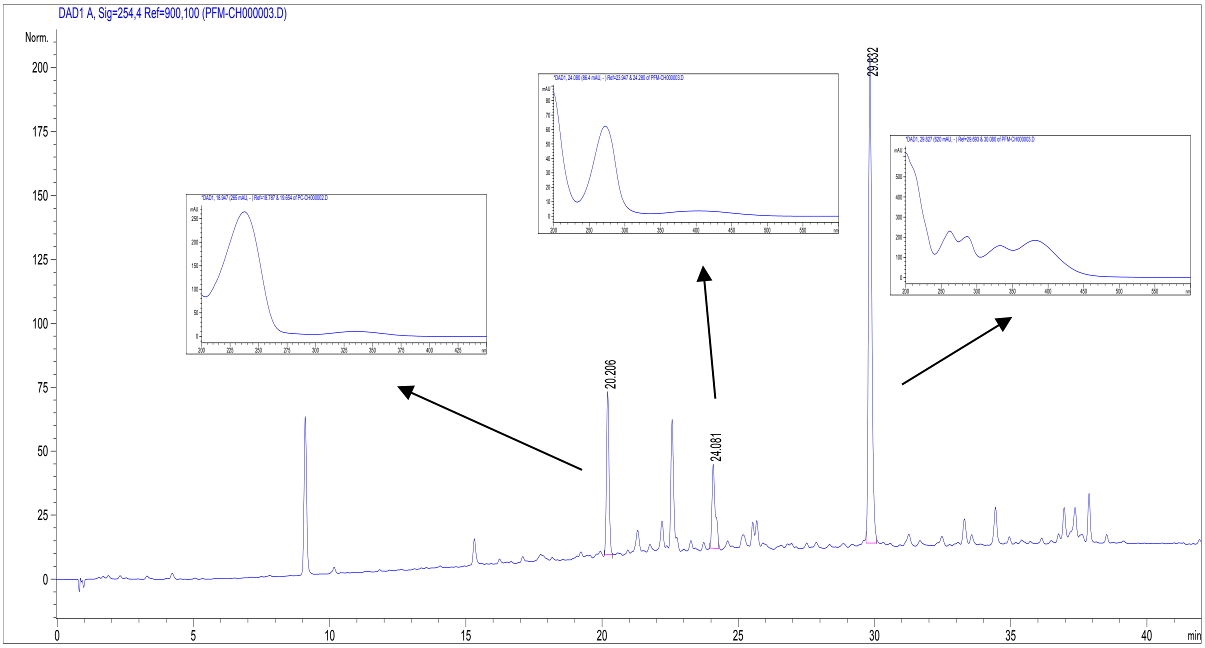

Figure S2. HPLC chromatogram of chloroform-soluble phase of *C. forsteri* 'Marginatus' at 254 nm with selected UV spectra (only peaks of diterpenes are integrated)

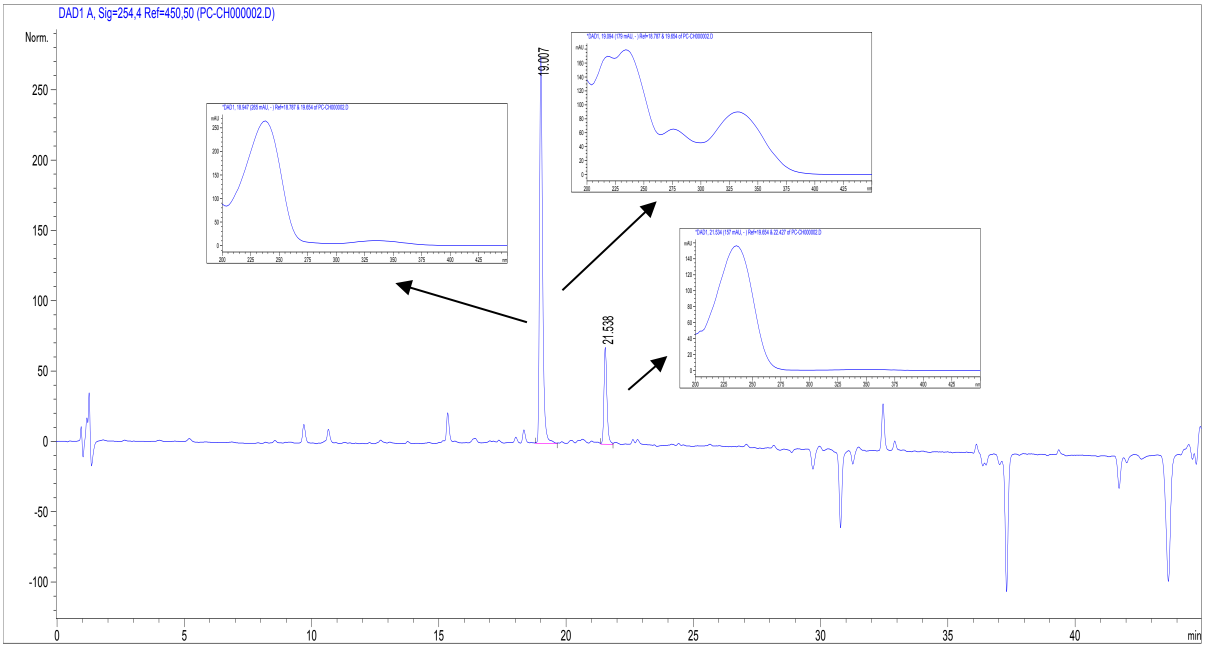

Figure S3. HPLC chromatogram of chloroform-soluble phase of *P. ciliatus* at 254 nm with selected UV spectra (only peaks of diterpenes are integrated; peak with t_R = 19.007 min is a mixture two components)

2. Extraction and isolation procedures

The frozen aerial parts of *C. comosus* (3.4 kg) were hand-crushed into small pieces and extracted with CH_3OH (12 L) in an ultrasonic bath at 40˚C for 1 h, followed by maceration at room temperature for 23 h. The extraction procedure was repeated three times. After filtration, the combined methanolic extracts were concentrated under reduced pressure at 40˚C to obtain a residue that was diluted with H_2O (700 mL) and subsequently subjected to solvent–solvent extraction with $CHCl_3$ (3 × 1 L). The chloroform portion (12.8 g) was subjected to further solvent–solvent extraction between 90% aqueous CH_3OH (500 mL) and petroleum ether (6 × 500 mL) to yield methanolic (5 g) and petroleum ether (5.4 g) portions.

Aliquots of the methanolic portion (1.3 g) and the petroleum ether portion (1.9 g) were fractionated directly by semipreparative HPLC. A linear gradient of CH_3CN–0.2% HCOOH (35–52% CH_3CN in 20 min, 5mL/min) was used for the methanolic portion, and four fractions (PNM/A–PNM/D) were collected based on a UV detector response at 280 nm. The fraction PNM/D (100 mg) was further purified by preparative HPLC using a Kinetex XB-C18 column, 60% aqueous CH_3OH (12 mL/min) as the mobile phase, and UV detection at 240 nm to obtain compounds **4** (15.5 mg) and **2** (74.8 mg). The petroleum ether extract was subjected to semipreparative HPLC with a linear gradient of CH_3CN–0.2% HCOOH (80–95% CH_3CN

in 25 min, 5 mL/min) and UV detection at 220 and 254 nm. Five fractions (PNP/A–PNP/E) were collected. The first fraction (PNP/A) was identified as pure compound **7** (185.6 mg).

The second aliquots of the methanolic portion (3.7 g) and the petroleum ether portion (3.4 g) were fractionated by column chromatography. The methanolic portion was added to the column (L × I.D. 720 × 3.9 mm) by dry loading (11.3 g silica gel) and chromatographed over a silica gel stationary phase (290 g) eluted with a stepwise gradient of $CHCl_3$–EtOAc (5:1–1:1, v/v) followed by pure CH_3OH to obtain 34 fractions (PNM-1–PNM-34) that were combined into 15 final fractions based on their TLC and HPLC profiles. The petroleum ether portion was prepared by dry loading (12.6 g silica gel) and fractionated over silica gel (300 g) in the column (L × I.D. 720 × 3.9 mm) using a stepwise gradient system of $CHCl_3$–EtOAc (15:1–1:1, v/v) followed by CH_3OH to obtain 35 fractions (PNP-1–PNP-35) combined into 11 final fractions based on their TLC and HPLC profiles. Four fractions were selected for further purification by semipreparative HPLC with UV detection at 254 nm. For the PNM-20-22 fraction (130 mg), a linear gradient system of CH_3CN–0.2% HCOOH (50–65% CH_3CN in 15 min, 4 mL/min) was selected to isolate compound **3** (22 mg). Separation of the fraction PNM-32-33 (108 mg) using a linear gradient of CH_3CN–0.2% HCOOH (30–50% CH_3CN in 14 min, 4 mL/min) yielded compound **1** (9 mg). Fraction PNP-35 (1406 mg) was separated with

a CH_3CN–0.2% HCOOH mobile phase using a linear gradient (60–100% CH_3CN in 20 min, 5 mL/min) to obtain compounds **8** (5 mg) and **6** (21 mg). A linear gradient of CH_3CN–0.2% HCOOH (55–100% CH_3CN in 30 min, 5 mL/min) was used for the PNP-5-8 fraction (589 mg) to obtain the compound **5** (12 mg).

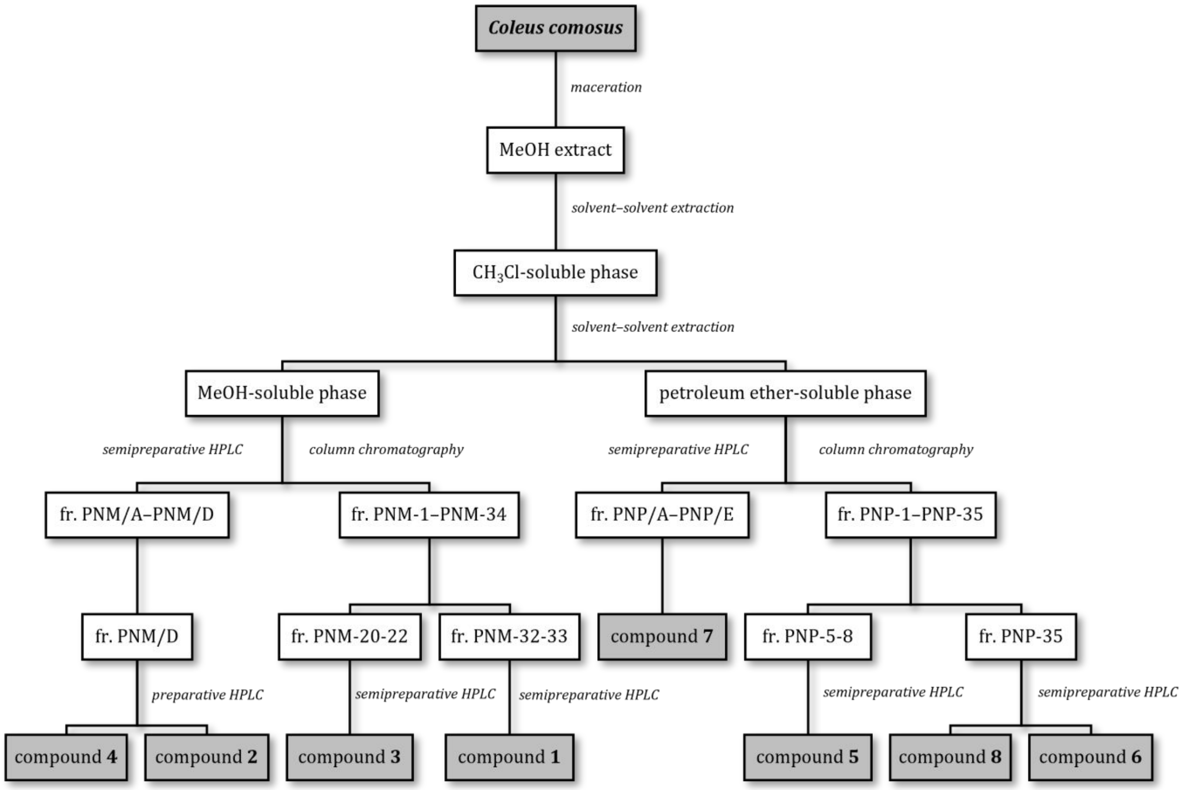

Figure S4. Separation scheme of the chloroform-soluble phase of *C. comosus*

The frozen aerial parts of *C. forsteri* 'Marginatus' (2.55 kg) were hand-crushed into small pieces and extracted with CH_3OH (9 L) by ultrasonication at 40˚C for 1 h and subsequently at room temperature for 23 h. The extraction procedure was performed three times. The

combined methanolic extracts were subjected to rotary evaporation at reduced pressure and a temperature of 40°C. The concentrated suspension was diluted with H_2O (400 mL) and subsequently extracted with CH_3Cl (3 × 500 mL).

The rough separation of the chloroform portion (3.5 g) was performed by dry-loading (18 g polyamide) flash chromatography over a polyamide stationary phase (200 g column) using a CH_3OH–H_2O stepwise gradient system (20:80 for 17 min, 40:60 for 33 min, 60:40 for 30 min, 80:20 for 45 min, 100:0 for 30 min, 19 mL/min), with PDA detection in the range of 210–410 nm to afford five fractions (PFM20%–PFM100%). The PFM60% fraction was subjected to preparative HPLC using a Kinetex XB-C18 column, with 55% aqueous CH_3CN (15 mL/min) as the mobile phase to yield five subfractions (PFM60%/1–PFM60%/5) collected based on a UV detector response at 210 nm. The PFM60%/2 subfraction was obtained as pure compound **9** (65 mg) and the PFM60%/5 subfraction (95.7 mg) was purified by preparative HPLC using a Kinetex XB-C18 column, with 67% aqueous CH_3OH (15 mL/min) as the eluent and UV detection at 210 nm to isolate compounds **10** (35.1 mg) and **11** (7.5 mg). The PFM80% fraction (1316.7 mg) was separated by preparative HPLC using a Kinetex XB-C18 column, with 65% aqueous CH_3CN (15 mL/min) as the mobile phase, and UV detection at 220 and 245 nm to obtain two subfractions (PFM80%/1 and PFM80%/2). Because of rapid

decomposition, the PFM80%/2 subfraction (133.6 mg) was repurified by preparative HPLC with a Gemini NX-C18 column, a mobile phase consisting of 68% aqueous CH_3CN with the addition of 2% CH_2Cl_2 calculated from the whole volume (15 mL/min) to yield compound **13** (11 mg) based on a UV detector response at 210 nm and 245 nm without exposure to light. After evaporation under a nitrogen stream without heating, pure **13** was stored at -20˚C.

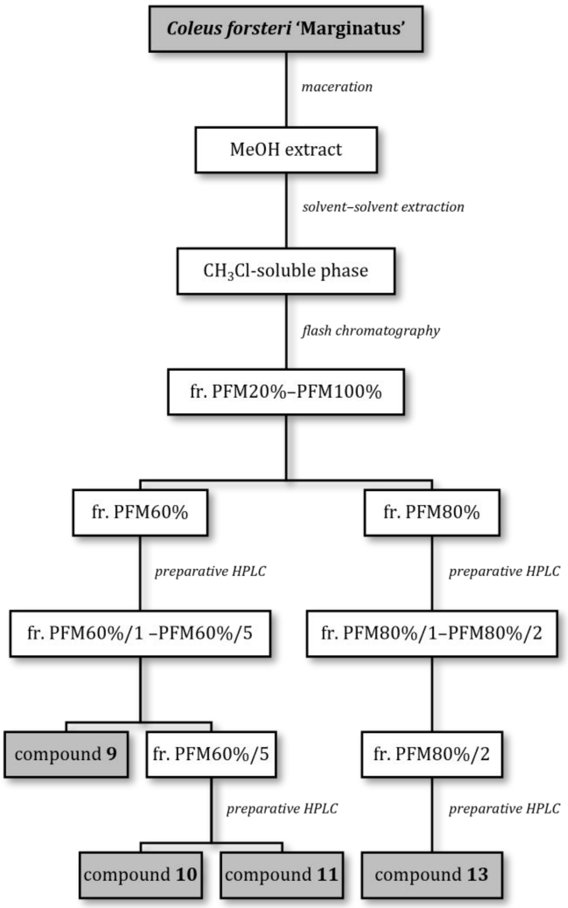

Figure S5. Separation scheme of the chloroform-soluble phase of *C. forsteri* 'Marginatus'

The frozen hand-crushed aerial parts of *P. ciliatus* (1.24 kg) were crushed into small pieces and extracted with CH_3OH (5 L) using ultrasonication at 40˚C for 1 h and subsequent maceration at room temperature for 23 h. The procedure was performed three times. The combined methanolic extracts were concentrated using a rotary evaporator under reduced pressure at 40˚C. After the addition of H_2O (400 mL), the suspension was extracted with chloroform (3 × 500 mL).

The dried chloroform portion (5.5 g) was fractionated by dry-loading (25 g polyamide) flash chromatography over a polyamide stationary phase (200 g column) eluted with a CH_3OH–H_2O stepwise gradient system (20:80, 40:60, 60:40, 80:20, 100:0, each ratio for approximately 20 min, 18 mL/min). Five fractions (PC20%–PC100%) were collected without UV detection until the solution became colorless. The PC60% fraction (1746 mg) was further separated by preparative HPLC using a Kinetex XB-C18 column, and a mixture of 45% aqueous CH_3CN with the addition of 1% CH_2Cl_2 calculated from the volume of the organic component (15 mL/min) as the mobile phase to obtain three subfractions (PC60%/1–PC60%/3) detected at 254 and 366 nm. The PC60%/1 (132.2 mg) and PC60%/3 (106.9 mg) subfractions were repurified by preparative HPLC using a Gemini NX-C18 column, a mobile phase composed of 50% aqueous CH_3CN with an addition of 1% of CH_2Cl_2 calculated from

the whole volume (15 mL/min), and a UV detector response at 227 nm for PC60%/1 to yield compound **15** (38.2 mg) and 225 nm for PC60%/3 to yield compound **14** (50.8 mg).

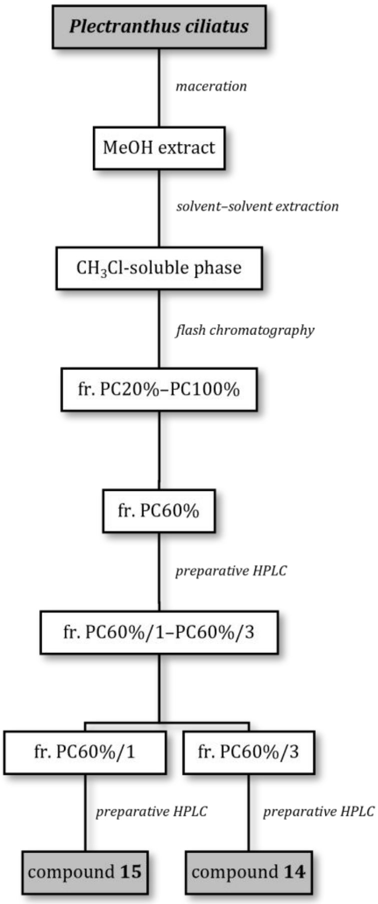

Figure S6. Separation scheme of the chloroform-soluble phase of *P. ciliatus*

3. ^{1}H–^{1}H COSY and key HMBC correlations of compounds of compounds 2–5

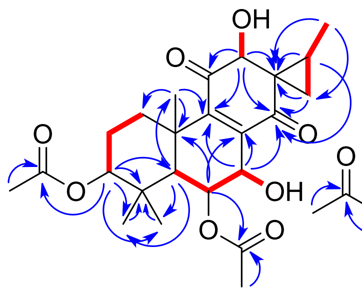

2

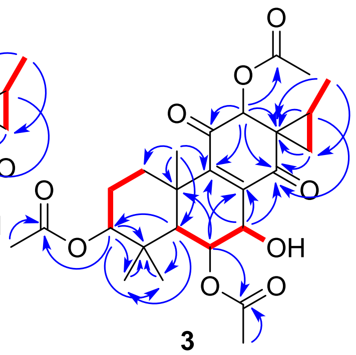

3

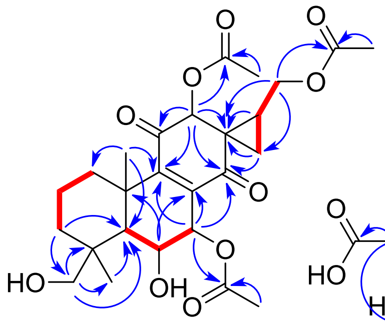

4

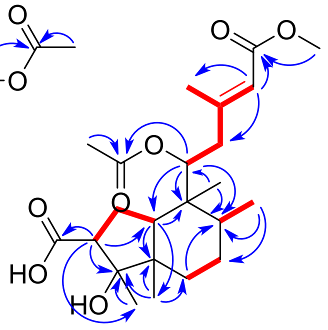

5

Figure S7. ^1H–^1H COSY (bold lines) and key HMBC ($^1H \rightarrow ^{13}C$, arrows) correlations of compounds **2**–**5**

4. Key NOESY correlations of compounds 2–5

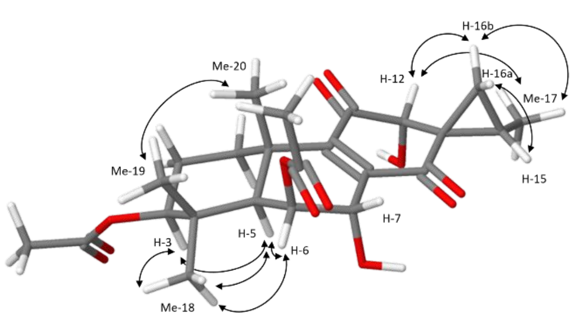

2

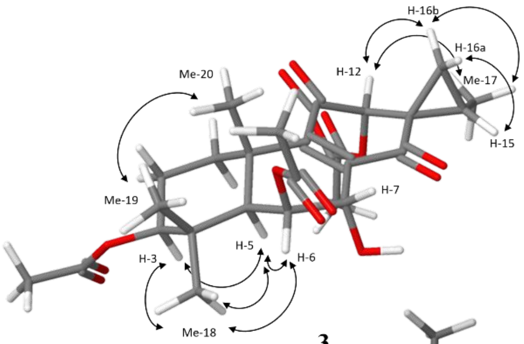

3

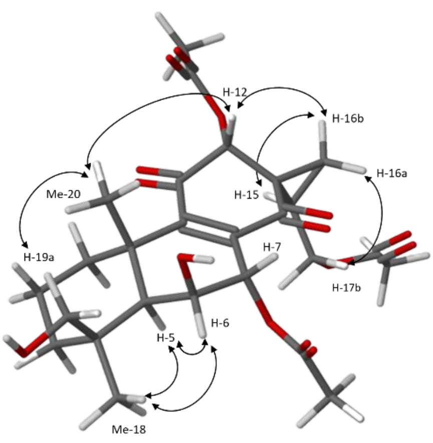

4

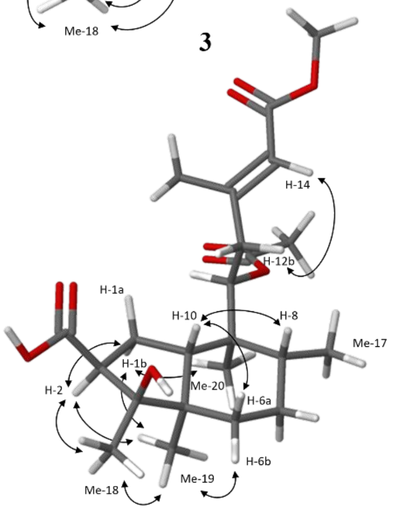

5

Figure S8. Key NOESY correlations (arrows) of compounds **2**–**5**

5. MS and NMR data for compound 1

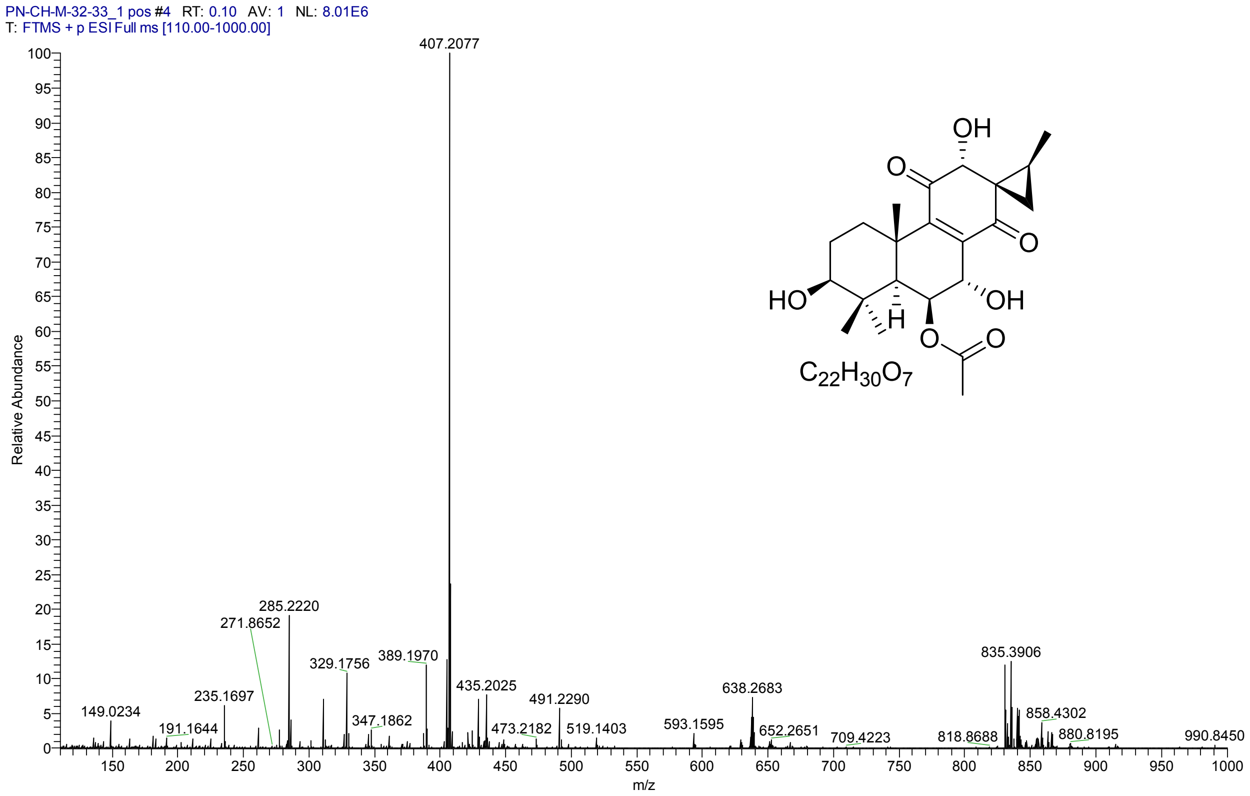

Figure S9. The HRESIMS spectrum of compound **1** (positive mode)

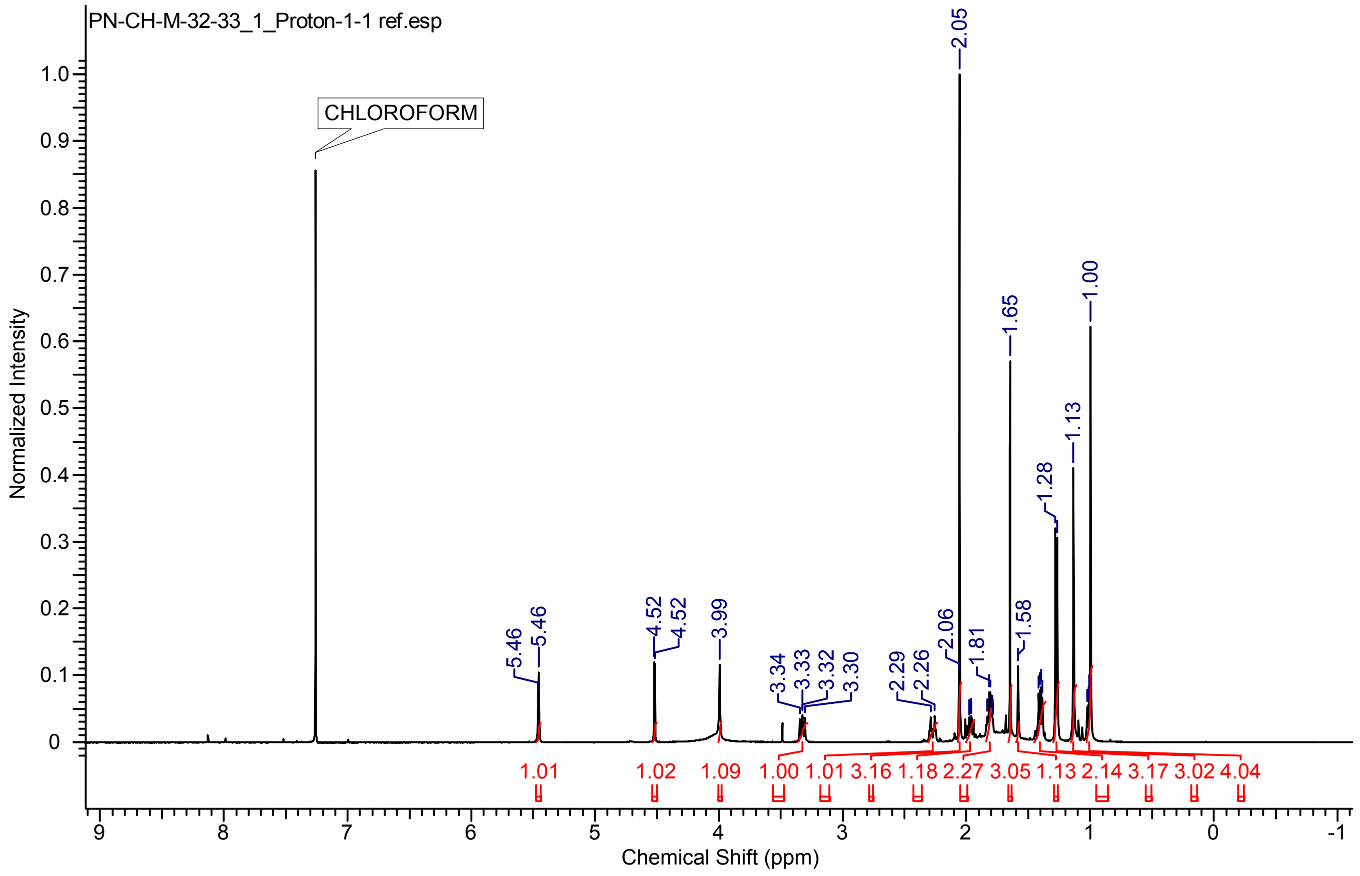

Figure S10. The ¹H NMR (400 MHz, $CDCl_3$) spectrum of compound **1**

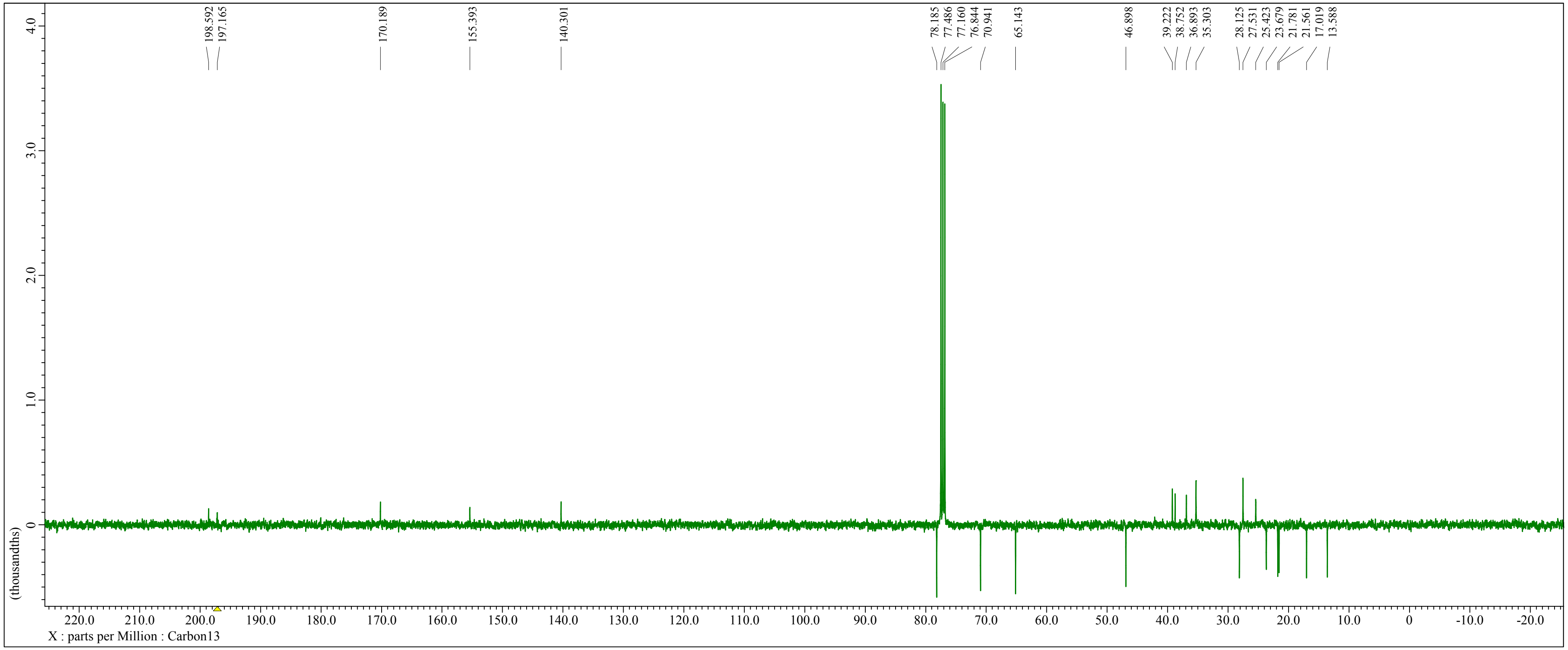

Figure S11. The ^{13}C NMR APT (100 MHz, $CDCl_3$) spectrum of compound **1**

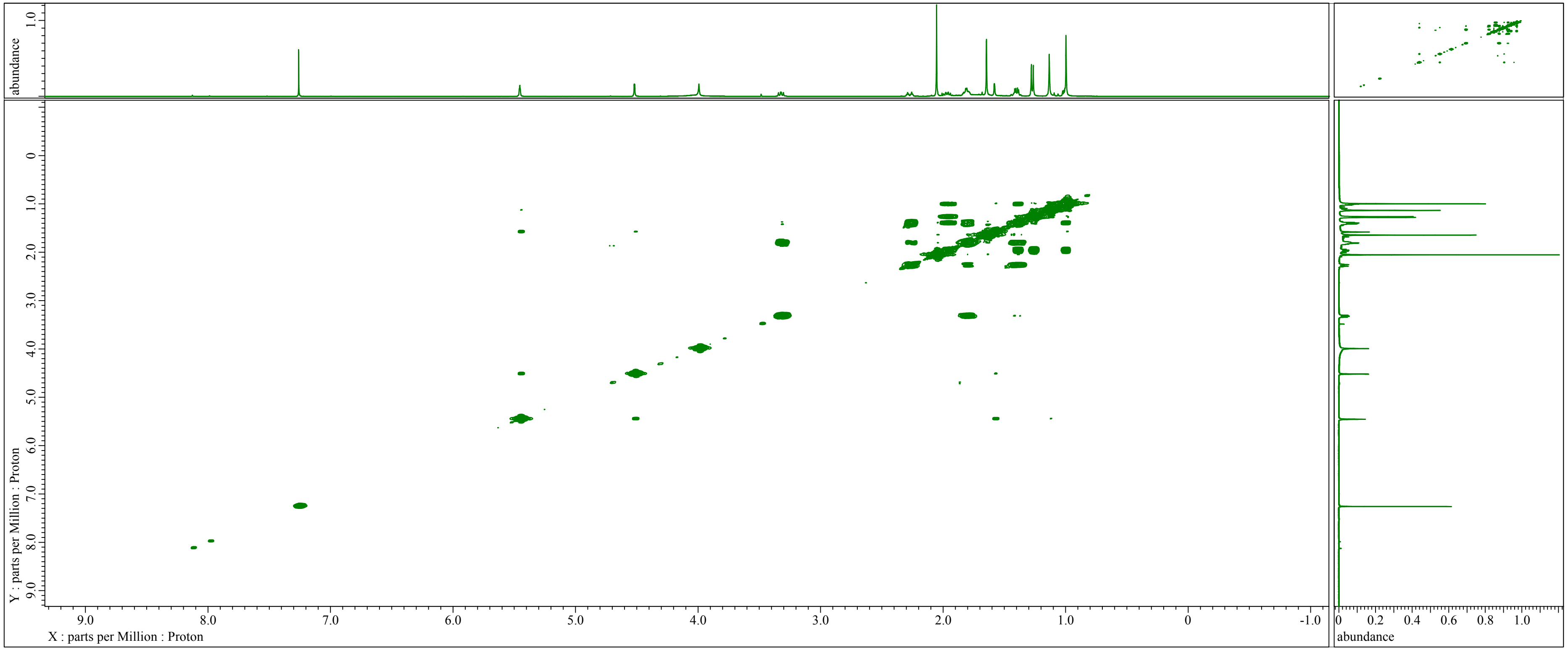

Figure S12. The COSY (400 MHz, $CDCl_3$) spectrum of compound **1**

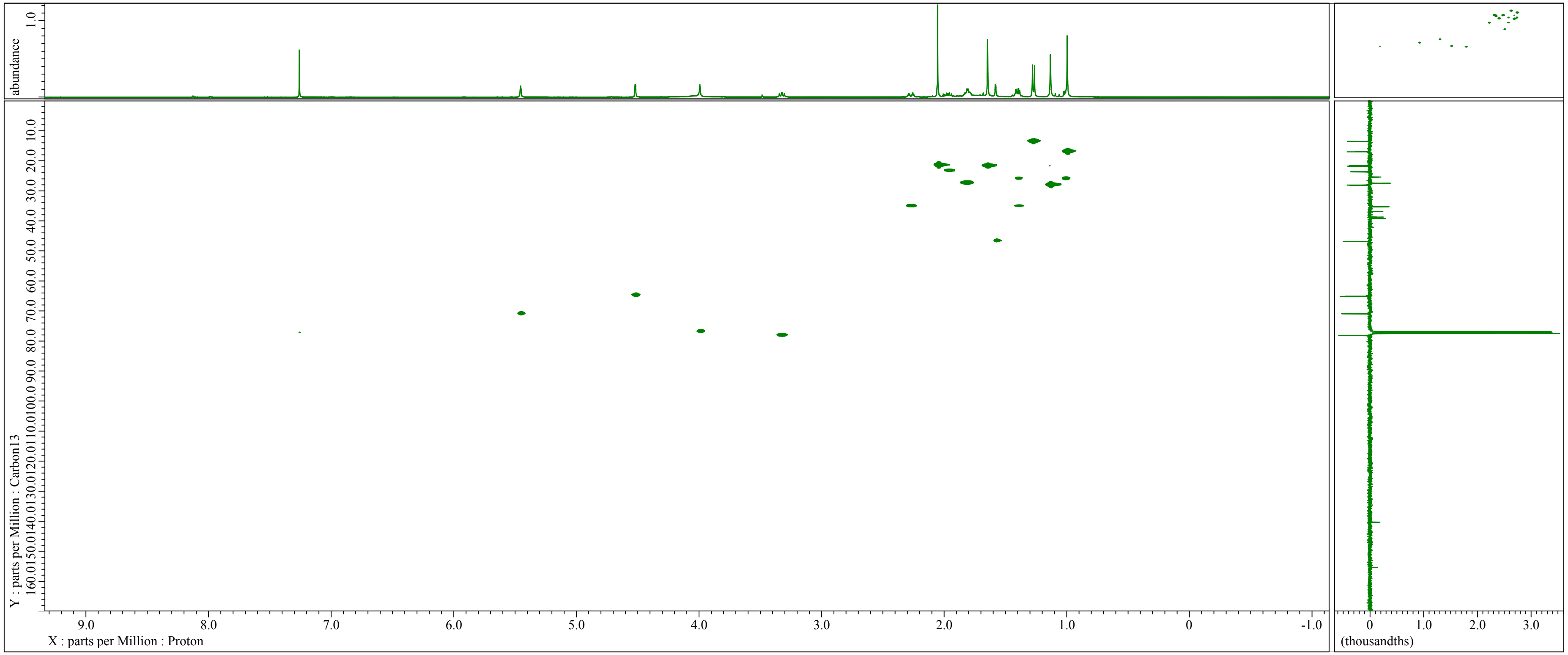

Figure S13. The HSQC (400/100 MHz, $CDCl_3$) spectrum of compound **1**

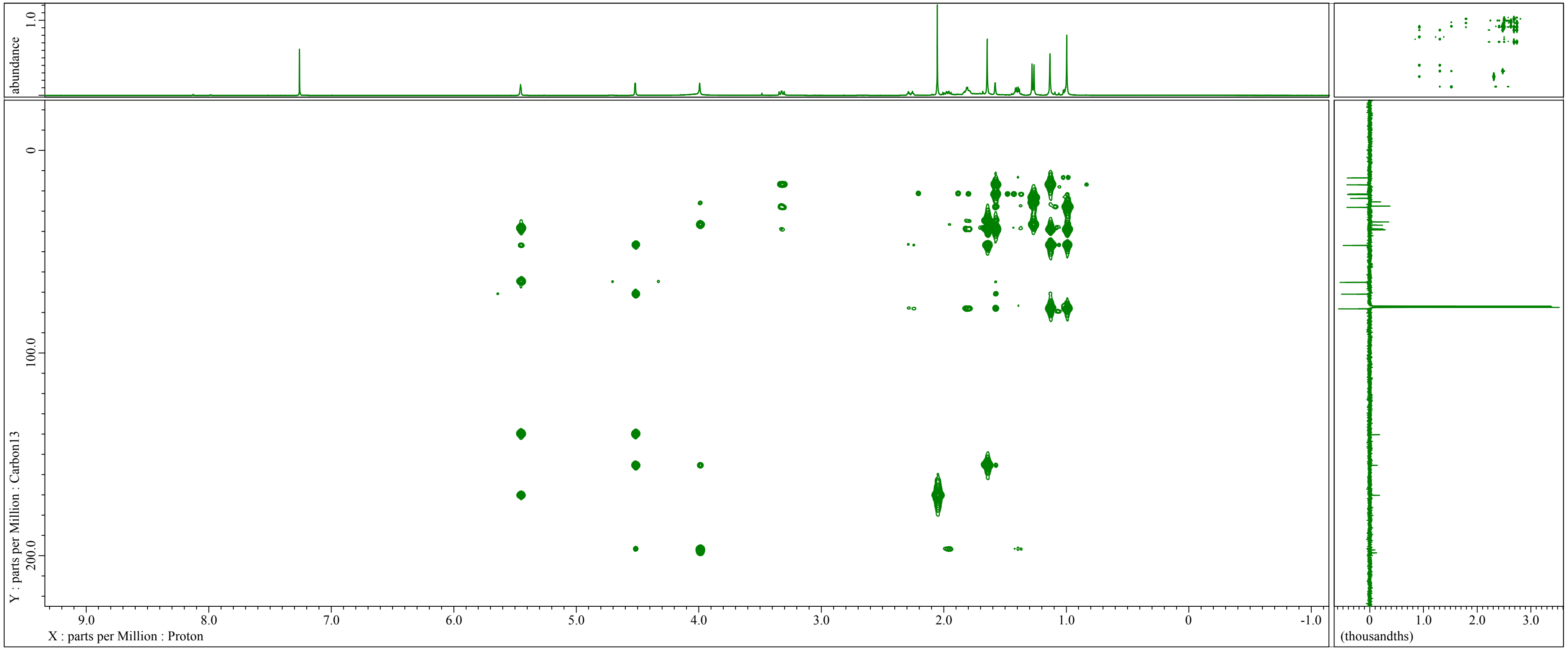

Figure S14. The HMBC (400/100 MHz, $CDCl_3$) spectrum of compound **1**

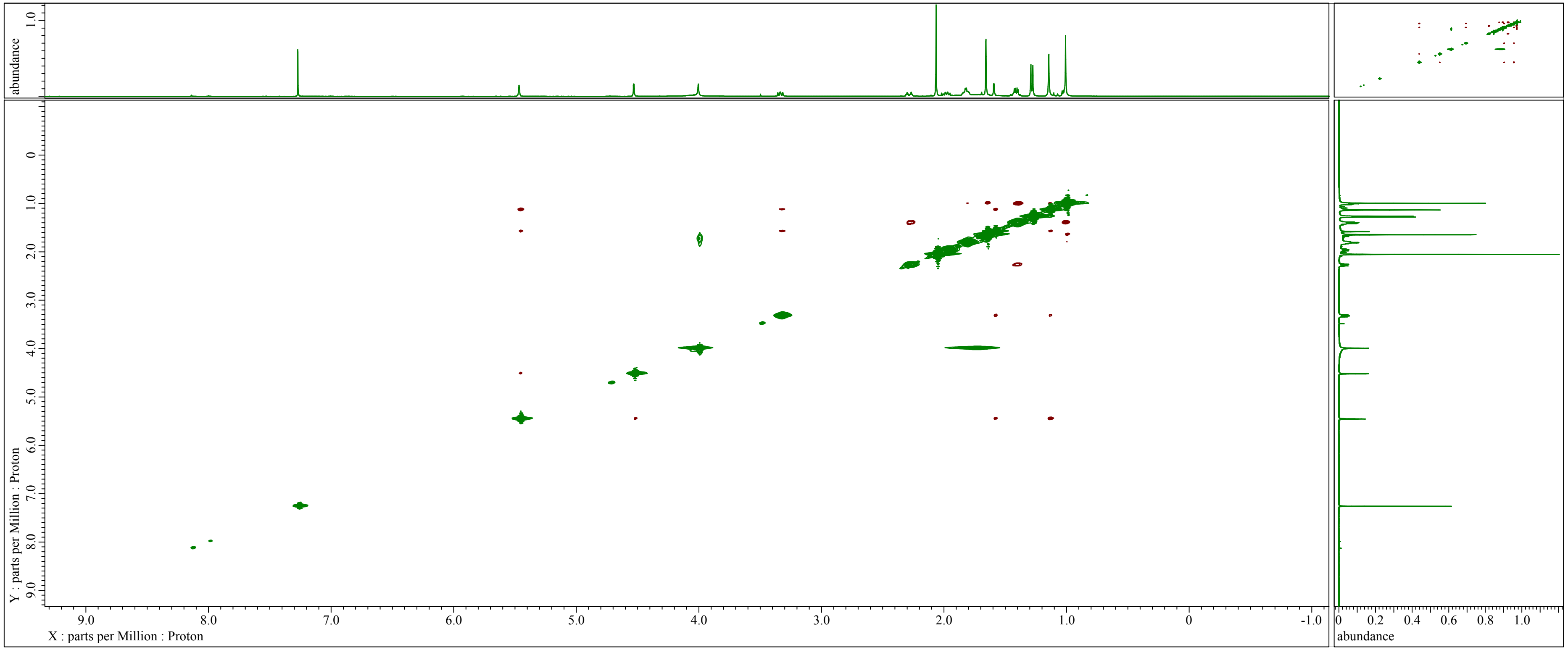

Figure S15. The NOESY (400 MHz, $CDCl_3$) spectrum of compound **1**

6. MS and NMR data for compound 2

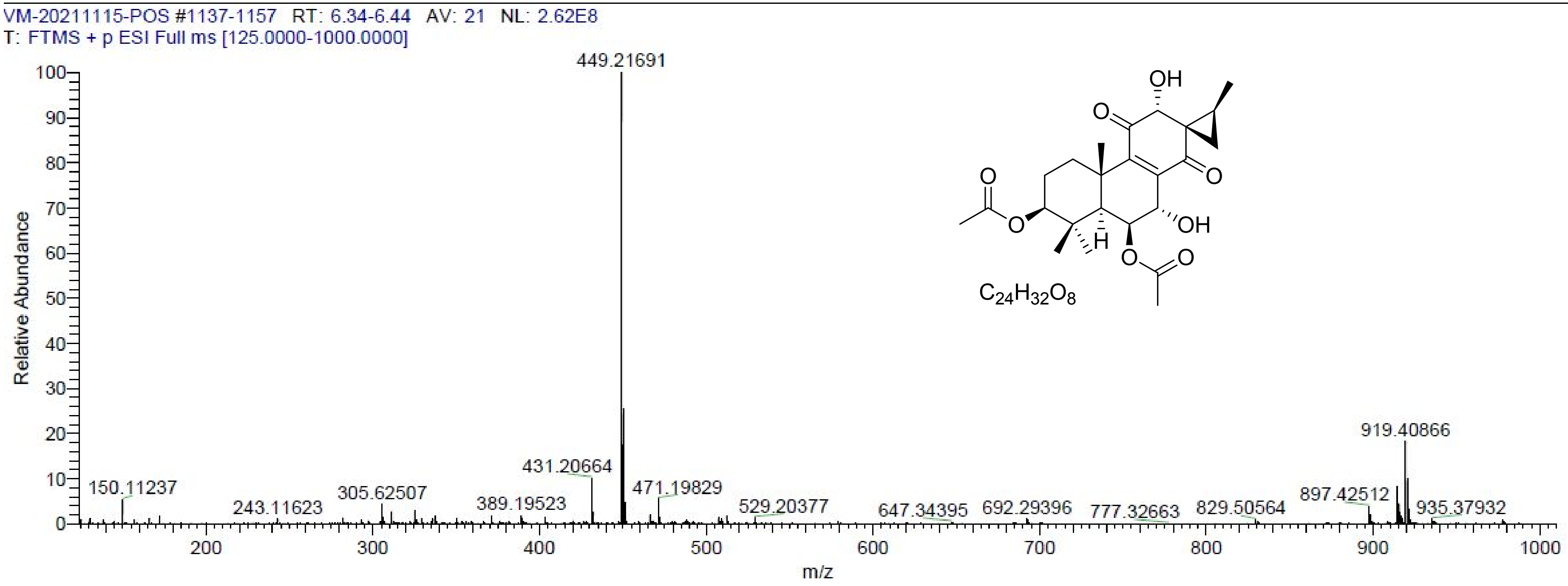

Figure S16. The HRESIMS spectrum of compound **2** (positive mode)

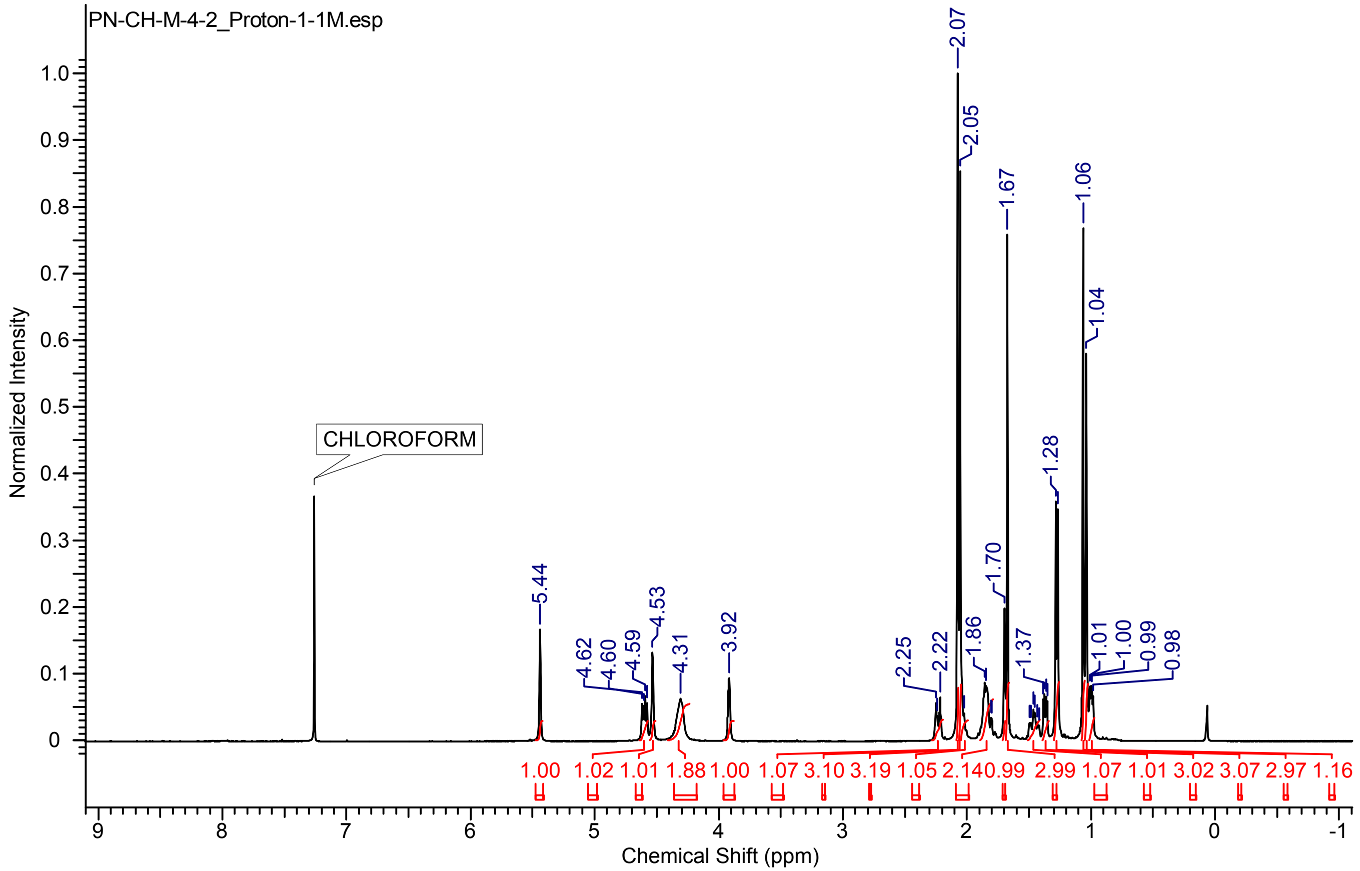

Figure S17. The ^{1}H NMR (400 MHz, $CDCl_3$) spectrum of compound **2**

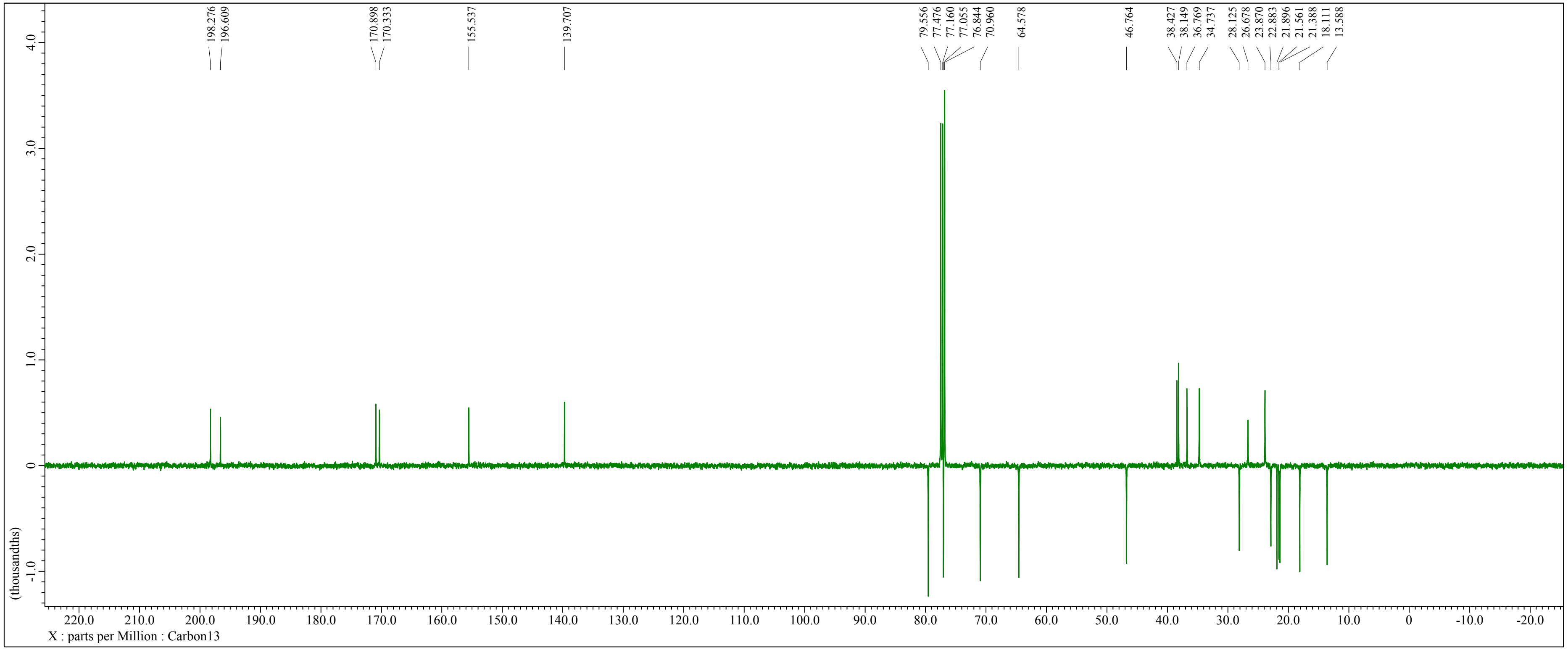

Figure S18. The ^{13}C NMR APT (100 MHz, $CDCl_3$) spectrum of compound **2**

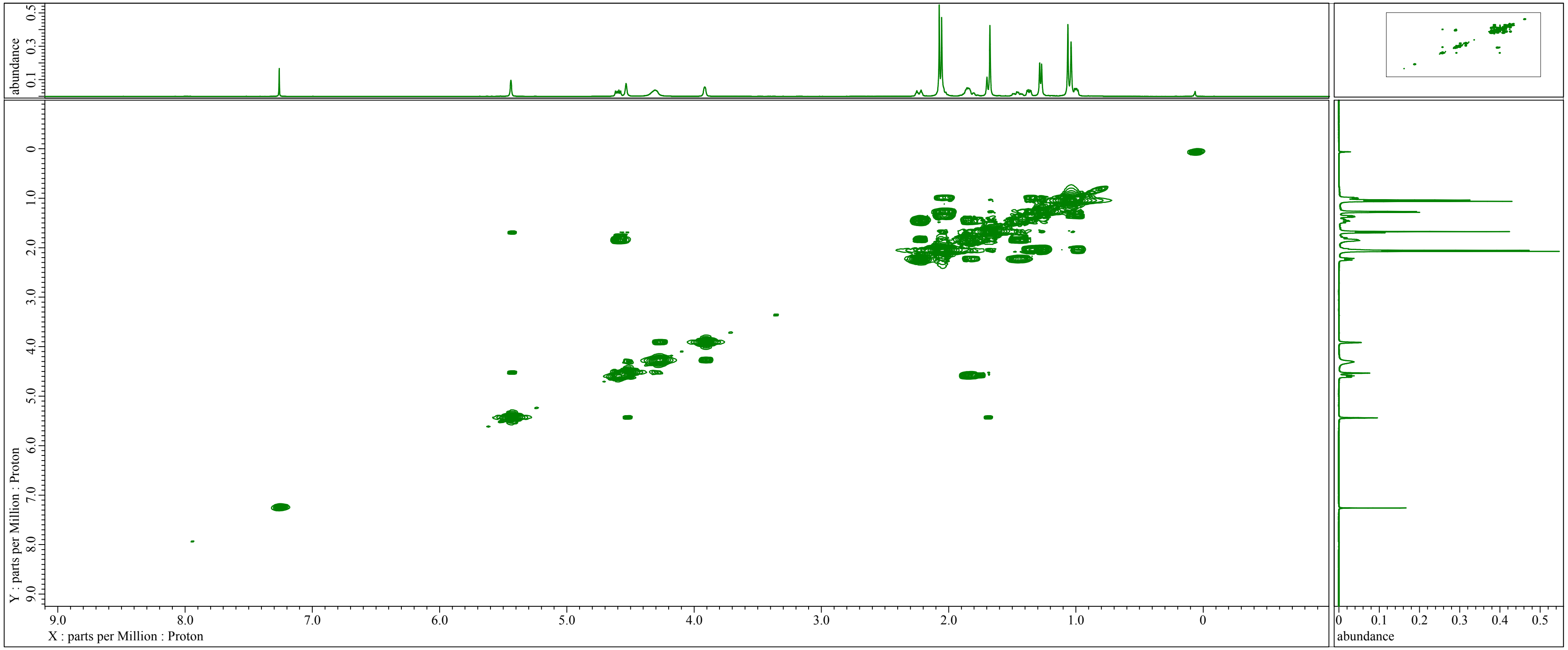

Figure S19. The COSY (400 MHz, $CDCl_3$) spectrum of compound **2**

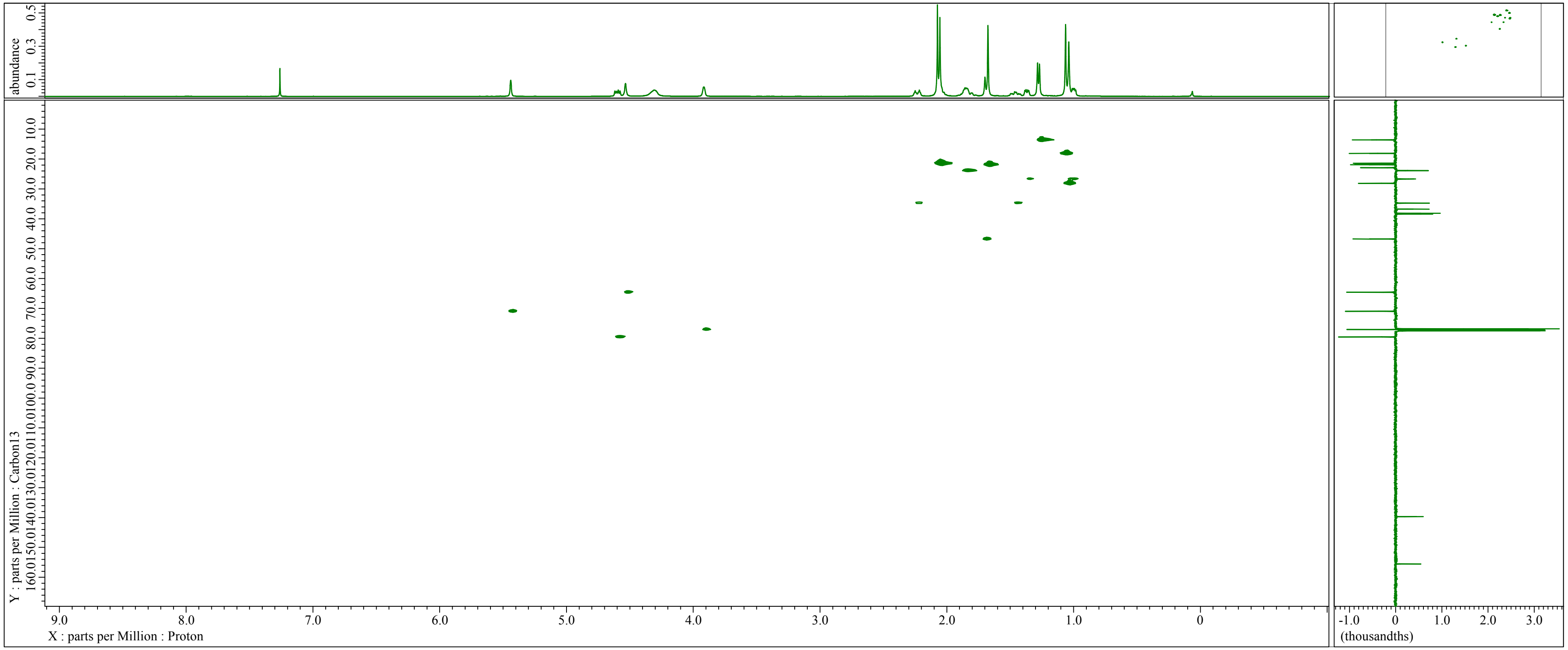

Figure S20. The HSQC (400/100 MHz, $CDCl_3$) spectrum of compound **2**

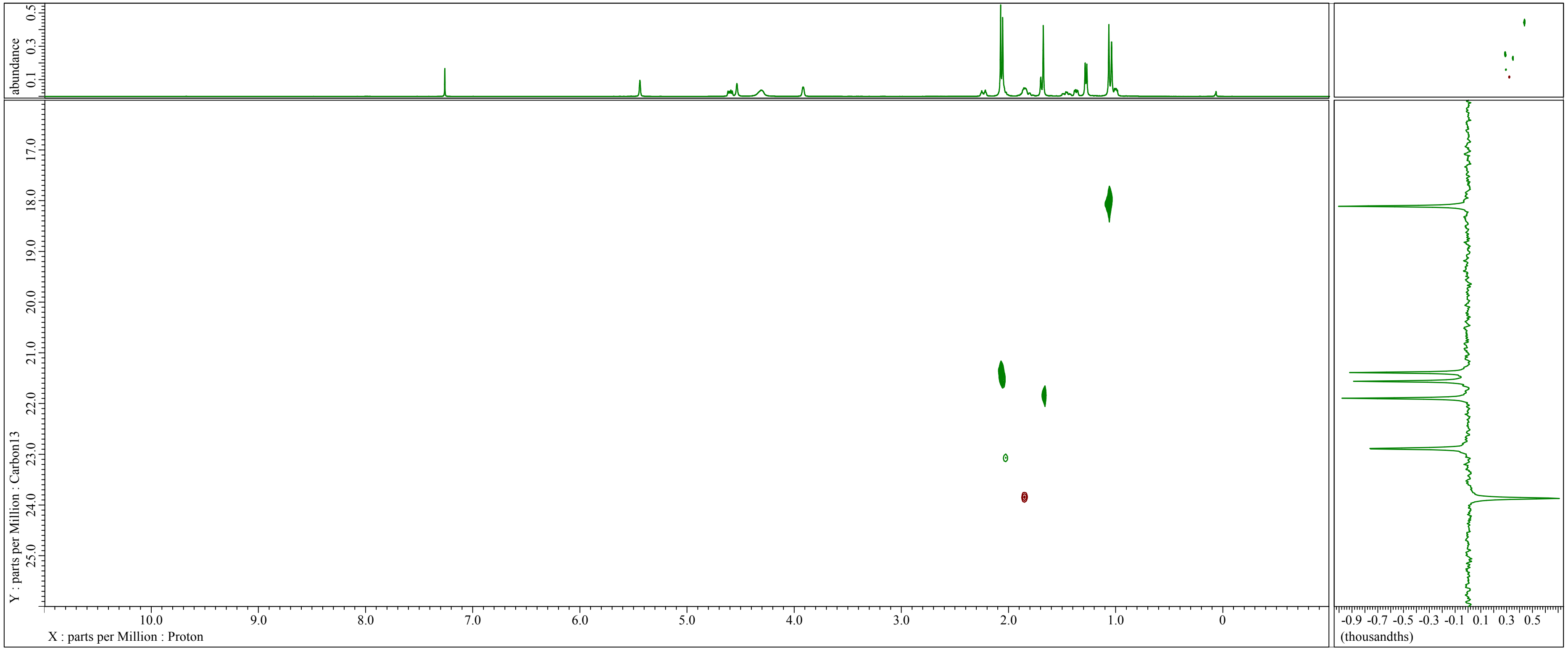

Figure S21. The band-selective HSQC (400/100 MHz, $CDCl_3$) spectrum of compound **2**

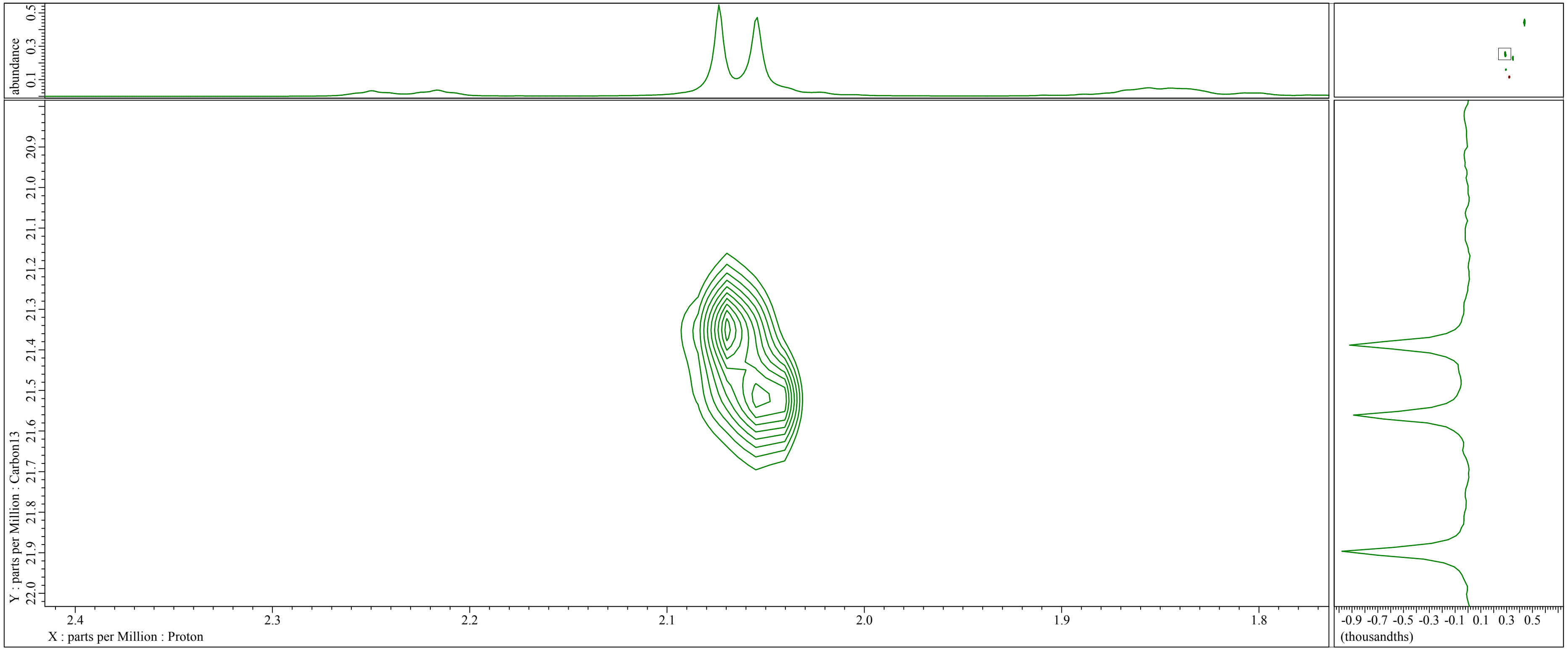

Figure S22. The inset (methyl region of the band-selective HSQC) (400/100 MHz, $CDCl_3$) spectrum of compound **2**

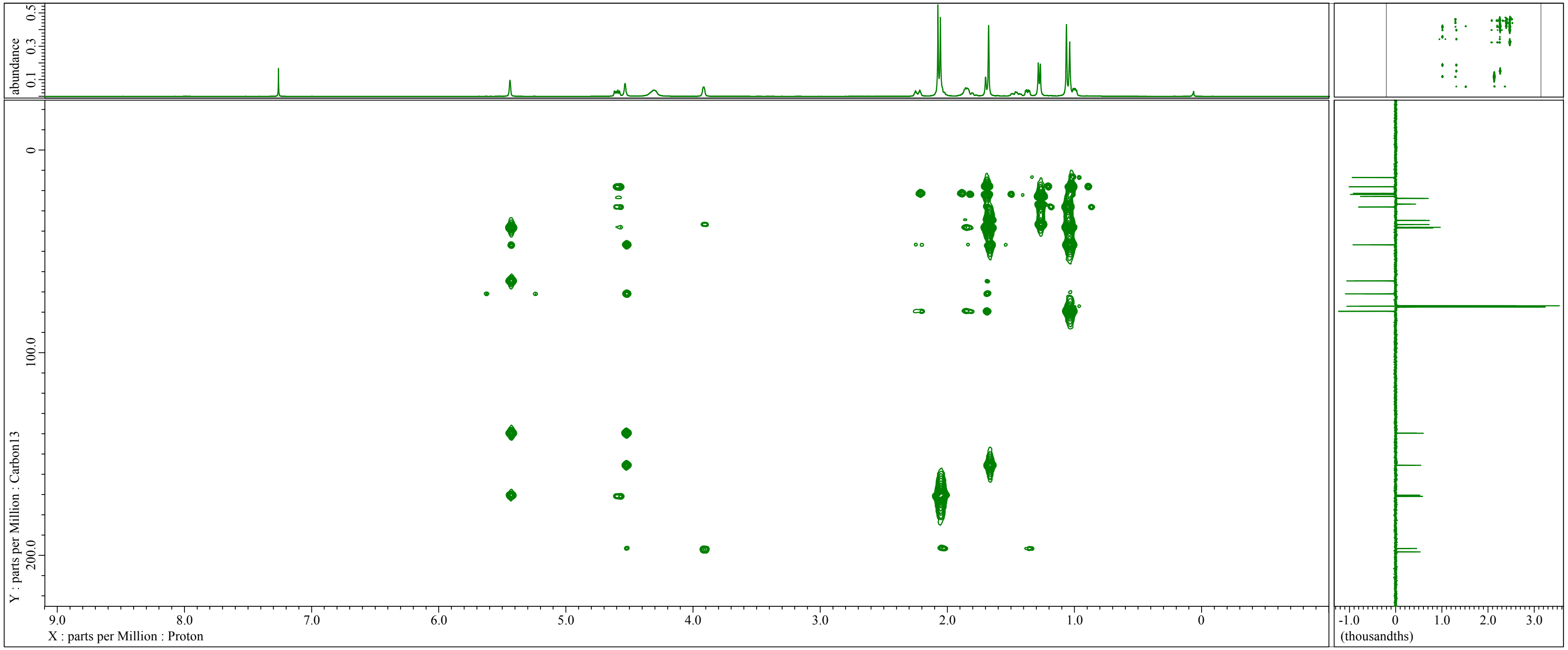

Figure S23. The HMBC (400/100 MHz, $CDCl_3$) spectrum of compound **2**

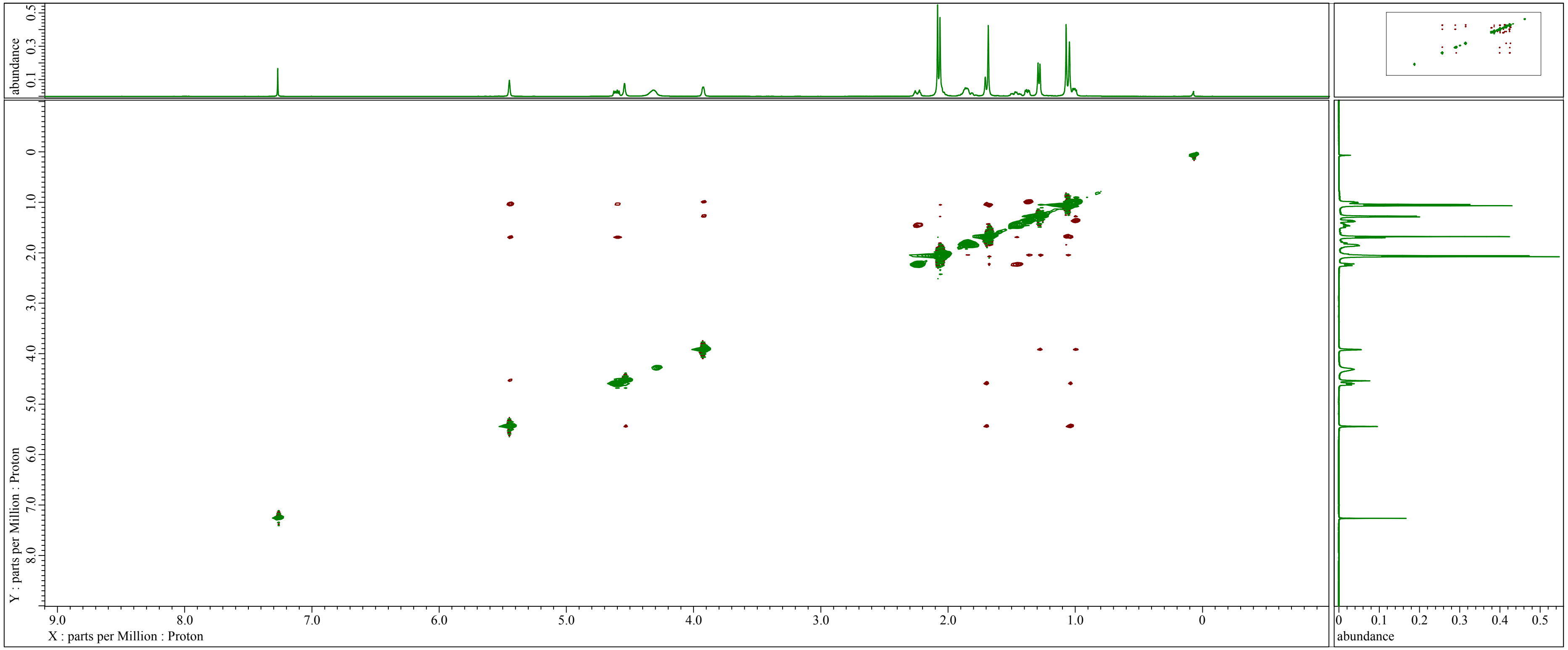

Figure S24. The NOESY (400 MHz, $CDCl_3$) spectrum of compound **2**

7. MS and NMR data for compound 3

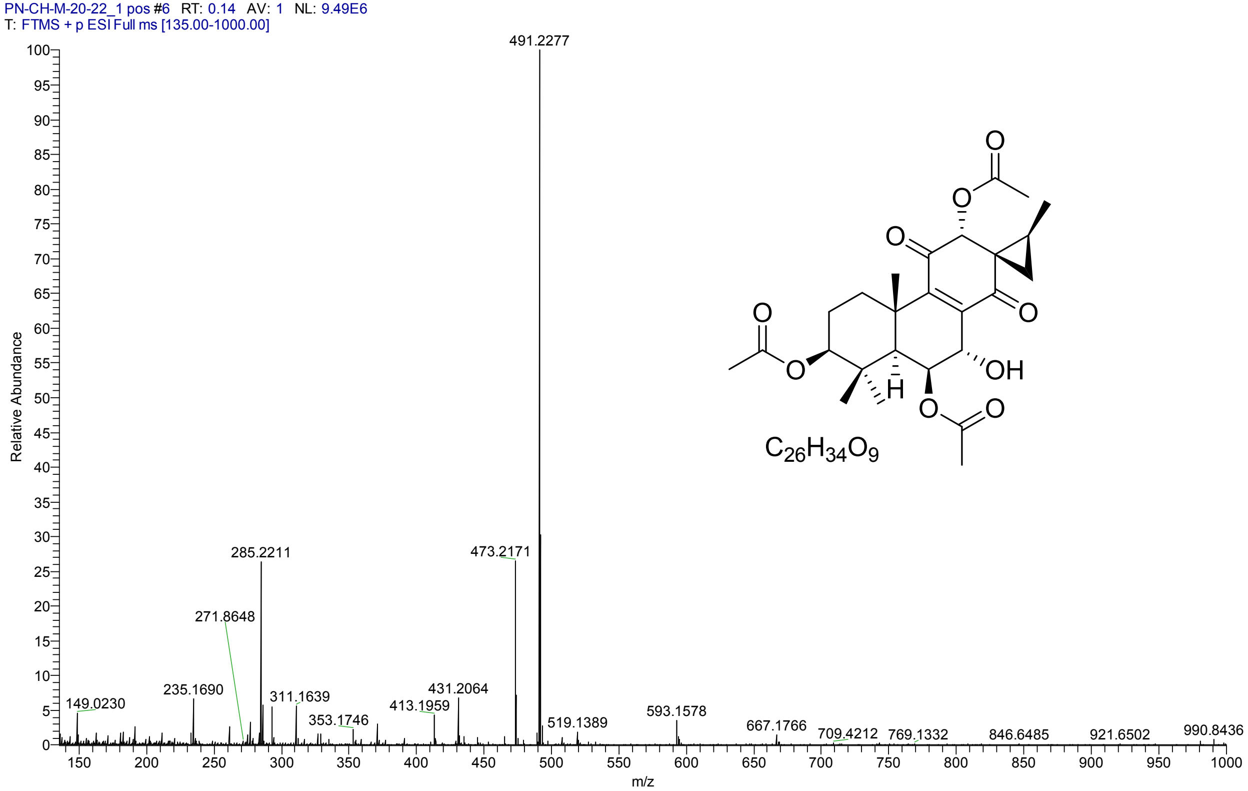

Figure S25. The HRESIMS spectrum of compound **3** (positive mode)

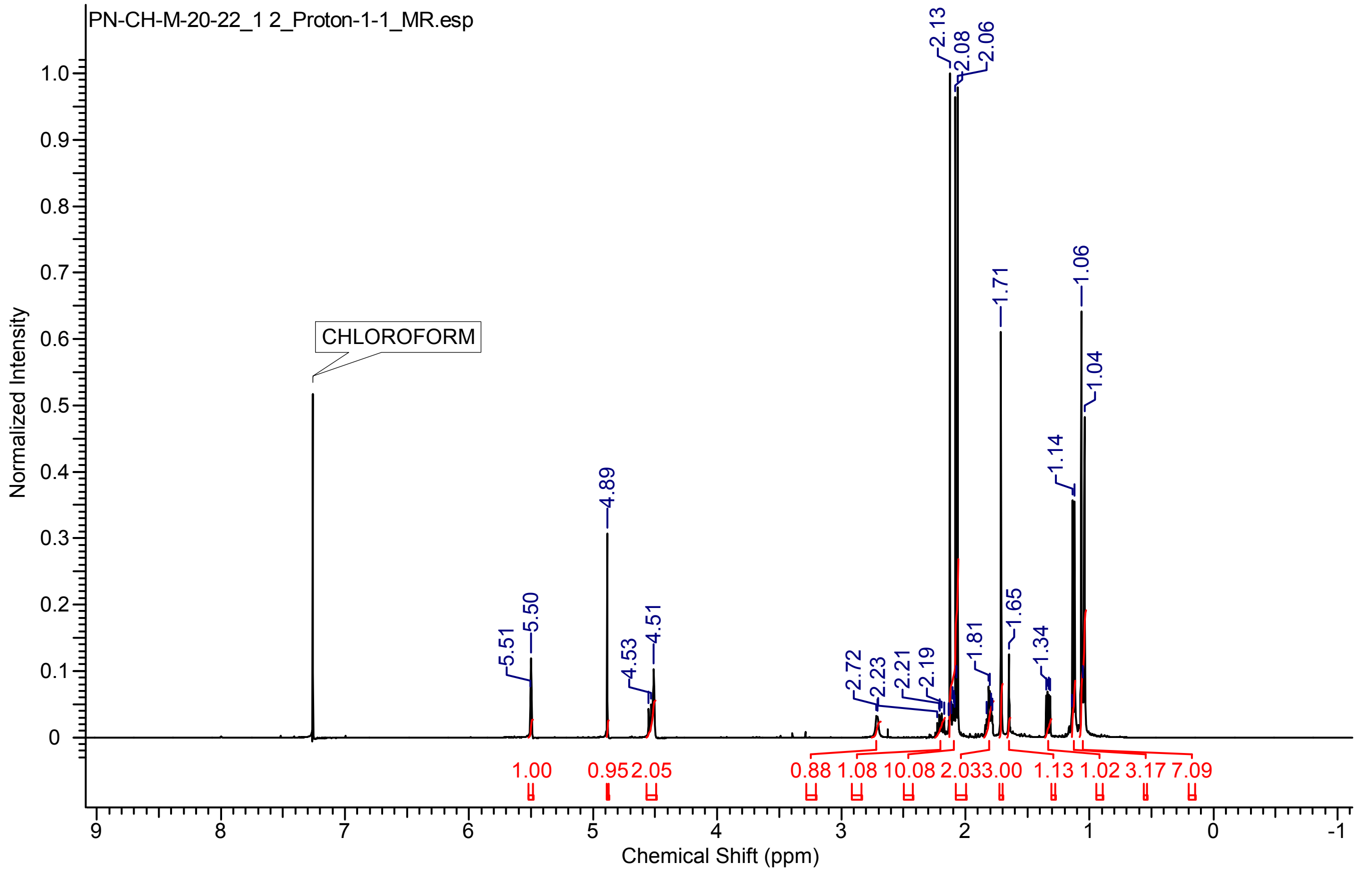

Figure S26. The ^1H NMR (400 MHz, $CDCl_3$) spectrum of compound **3**

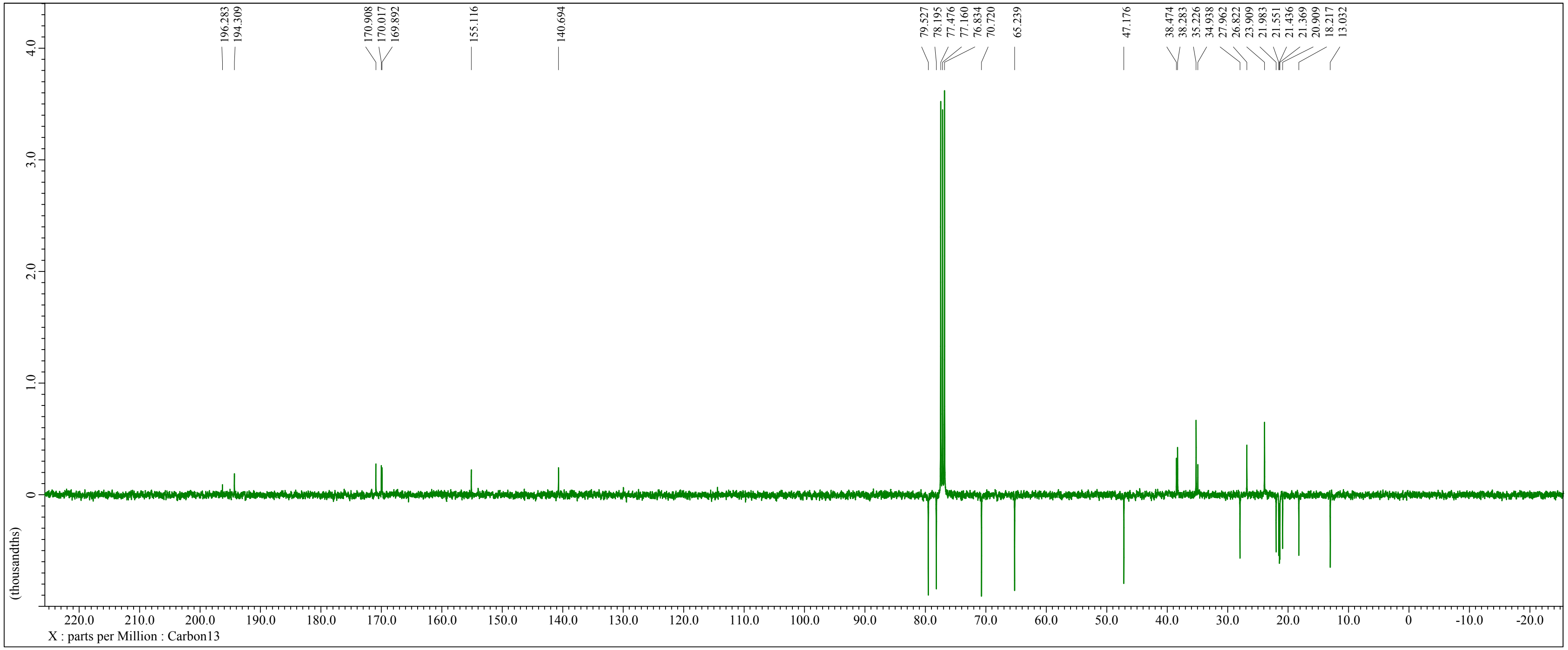

Figure S27. The ^{13}C NMR APT (100 MHz, $CDCl_3$) spectrum of compound **3**

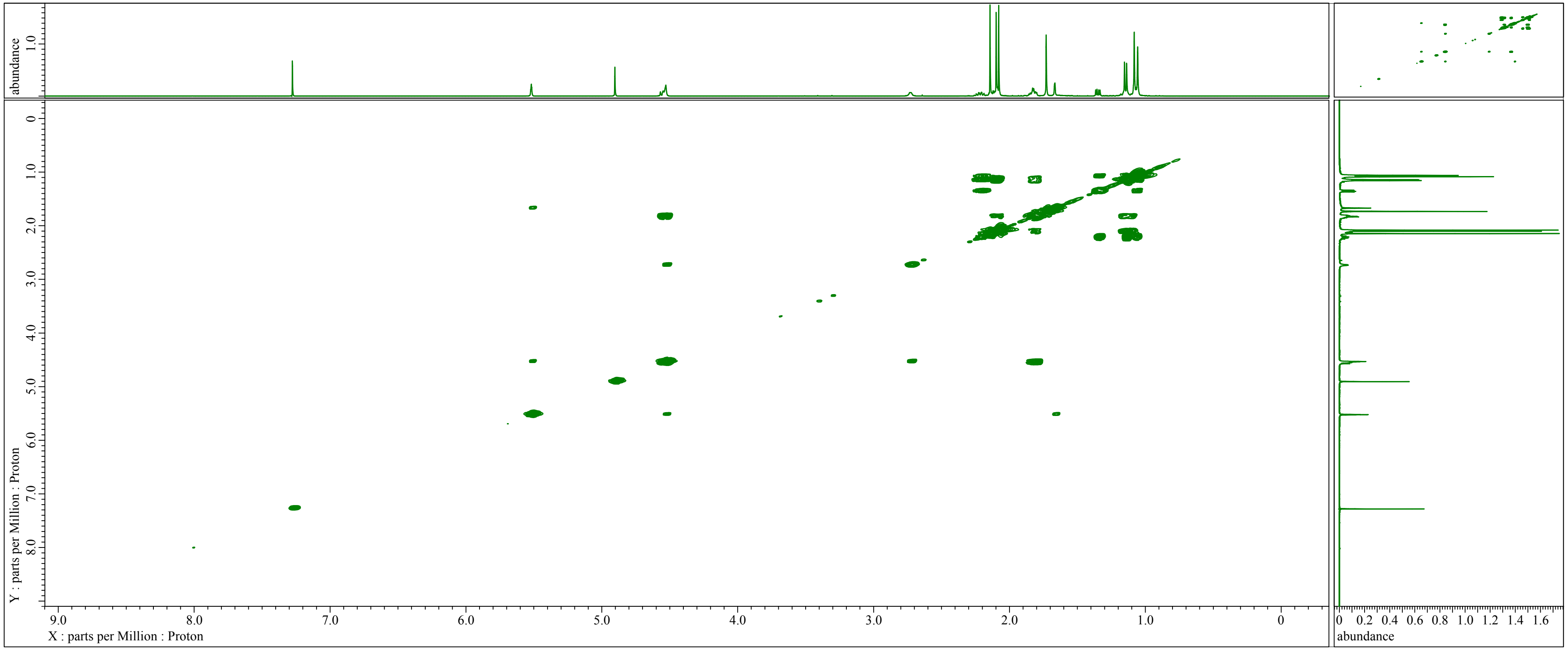

Figure S28. The COSY (400 MHz, $CDCl_3$) spectrum of compound **3**

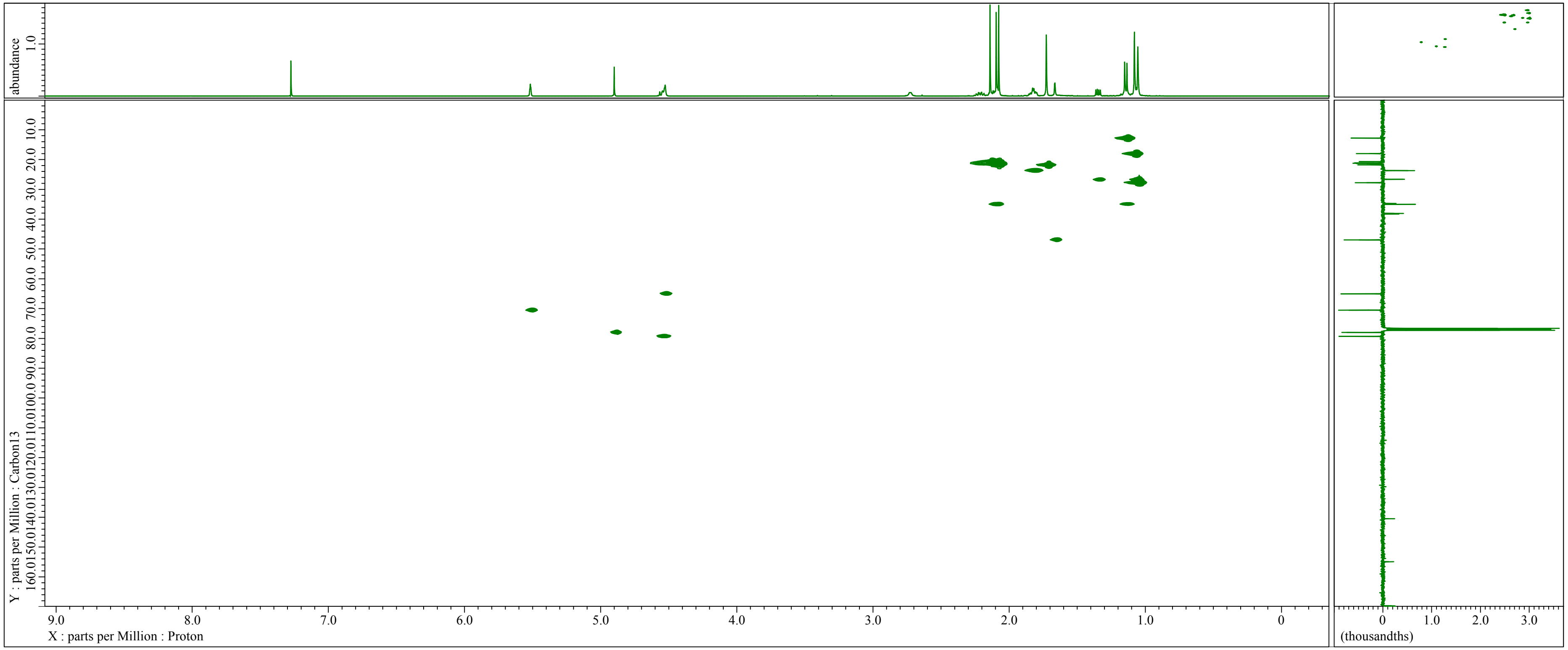

Figure S29. The HSQC (400/100 MHz, $CDCl_3$) spectrum of compound **3**

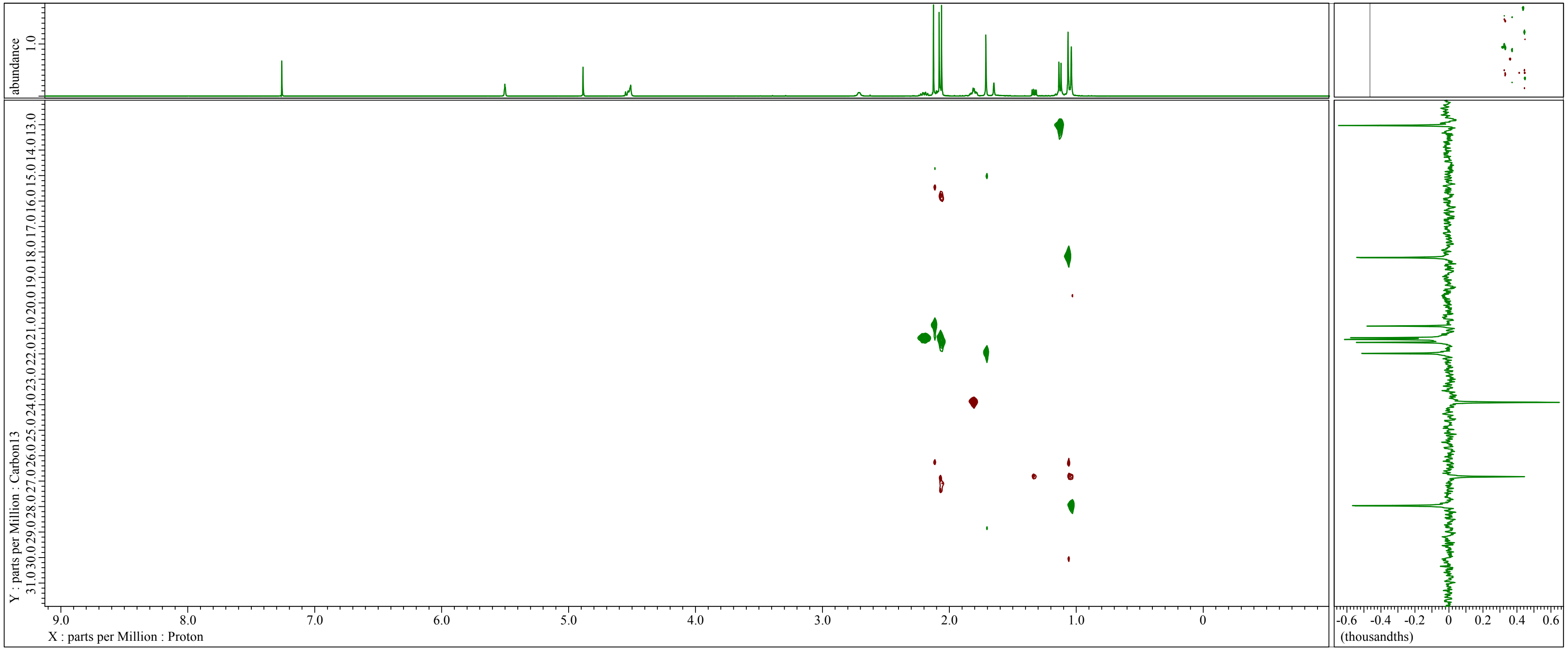

Figure S30. The band-selective HSQC (400/100 MHz, $CDCl_3$) spectrum of compound **3**

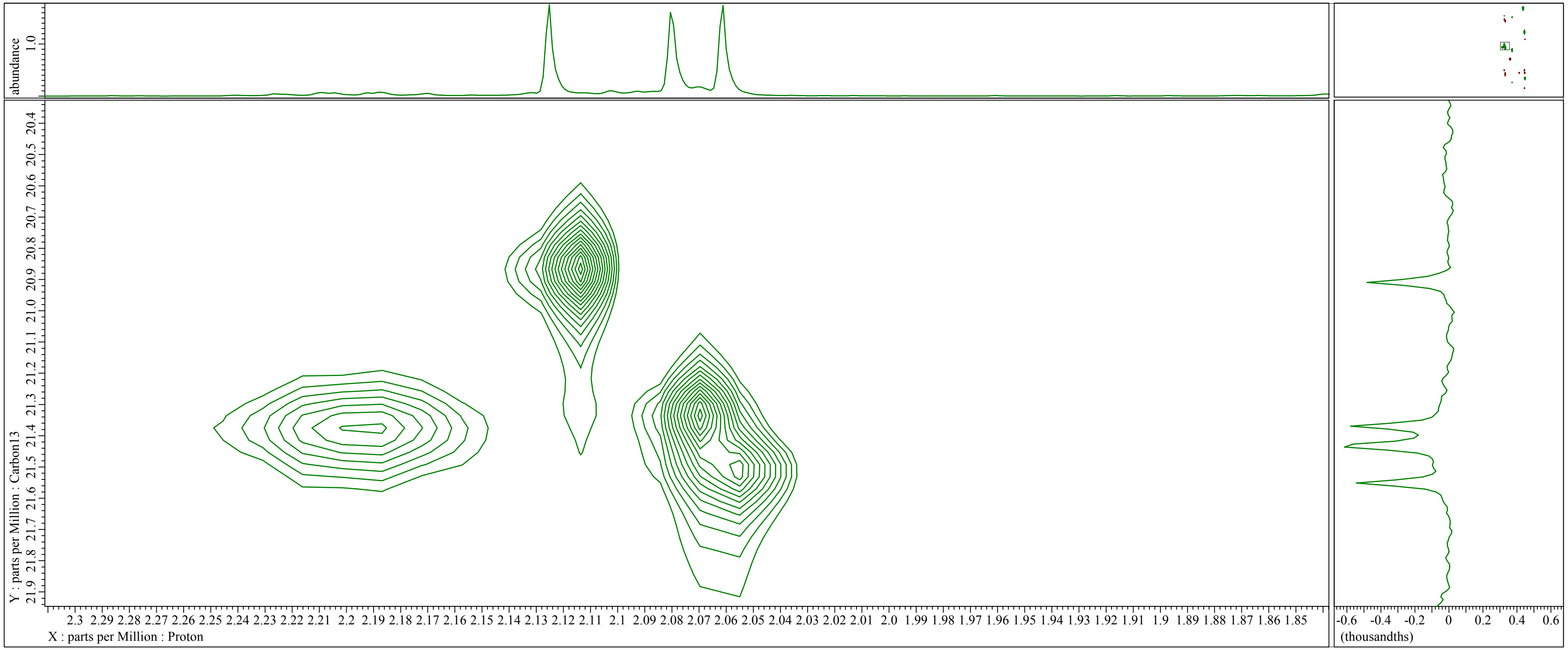

Figure S31. The inset (methyl and methine region of the band-selective HSQC) (400/100 MHz, $CDCl_3$) spectrum of compound **3**

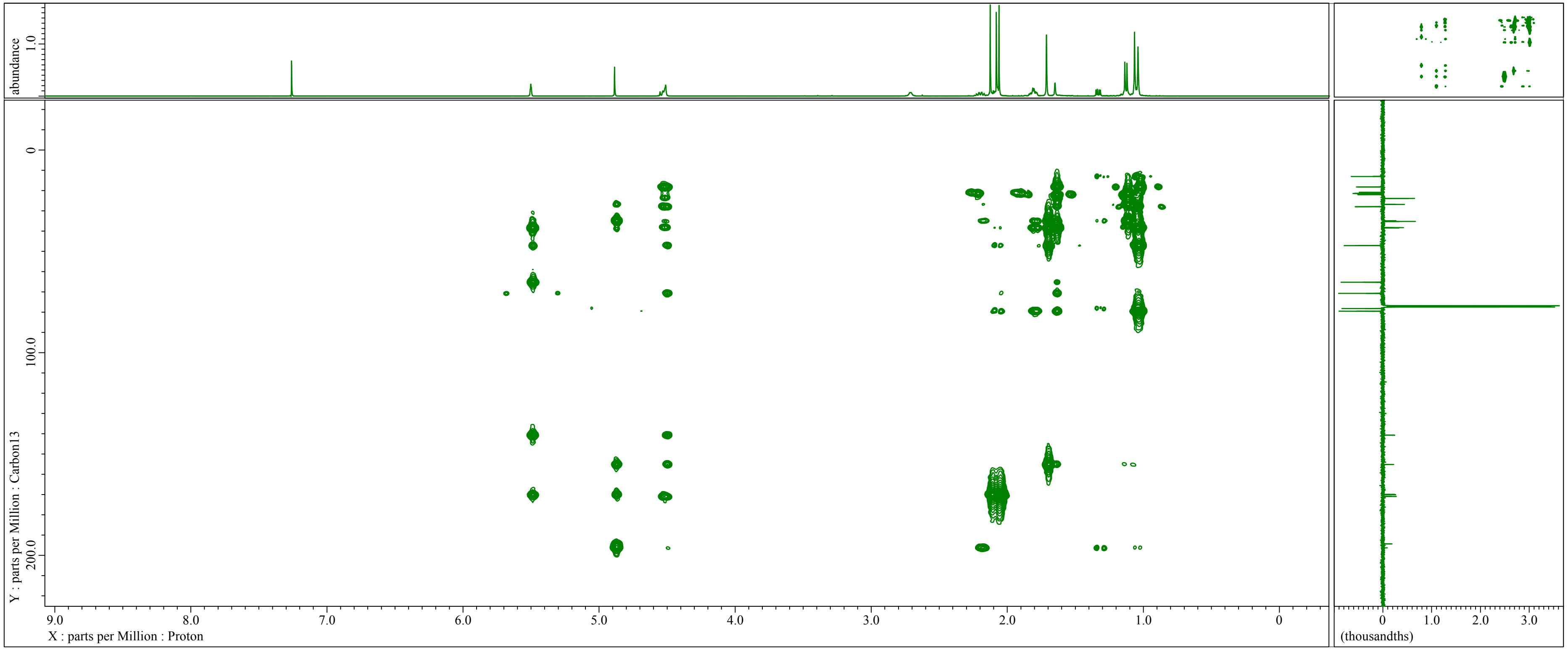

Figure S32. The HMBC (400/100 MHz, $CDCl_3$) spectrum of compound **3**

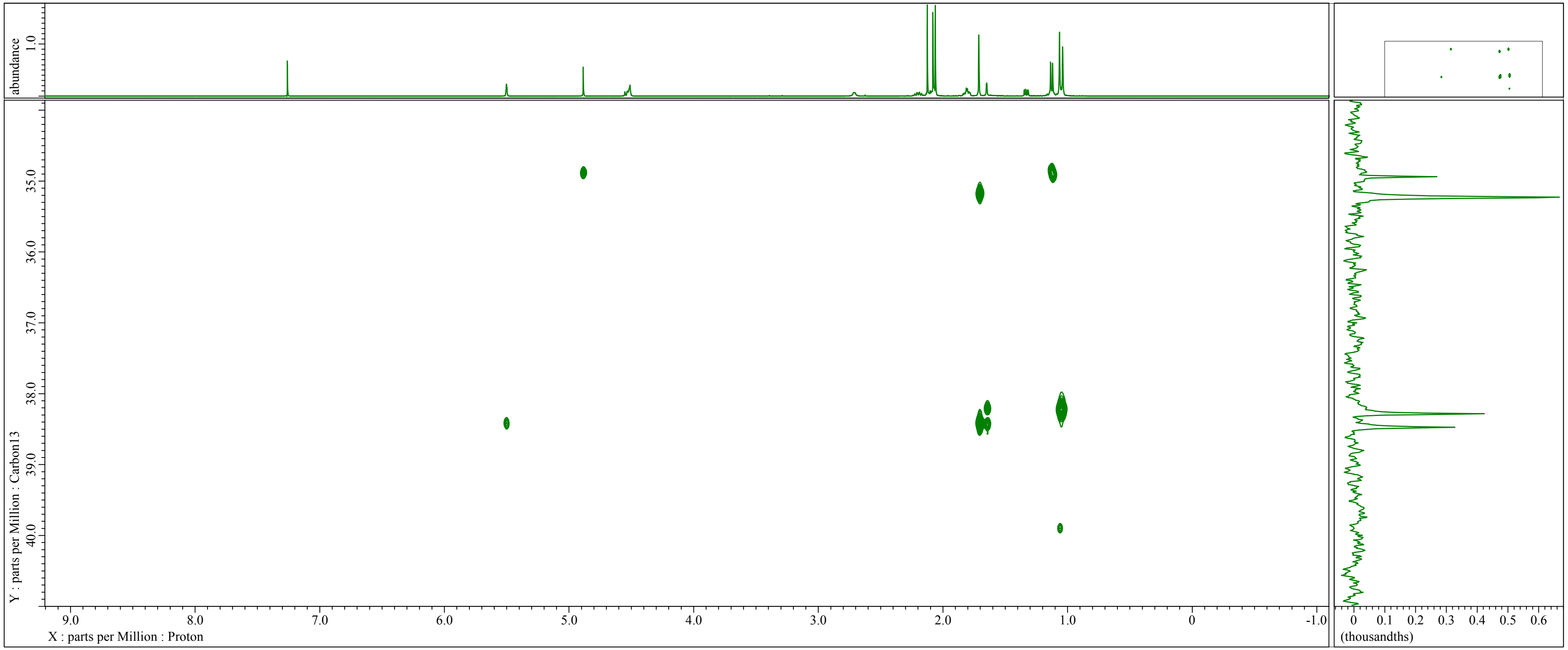

Figure S33. The band-selective HMBC (region 34–41 ppm) (400/100 MHz, $CDCl_3$) spectrum of compound **3**

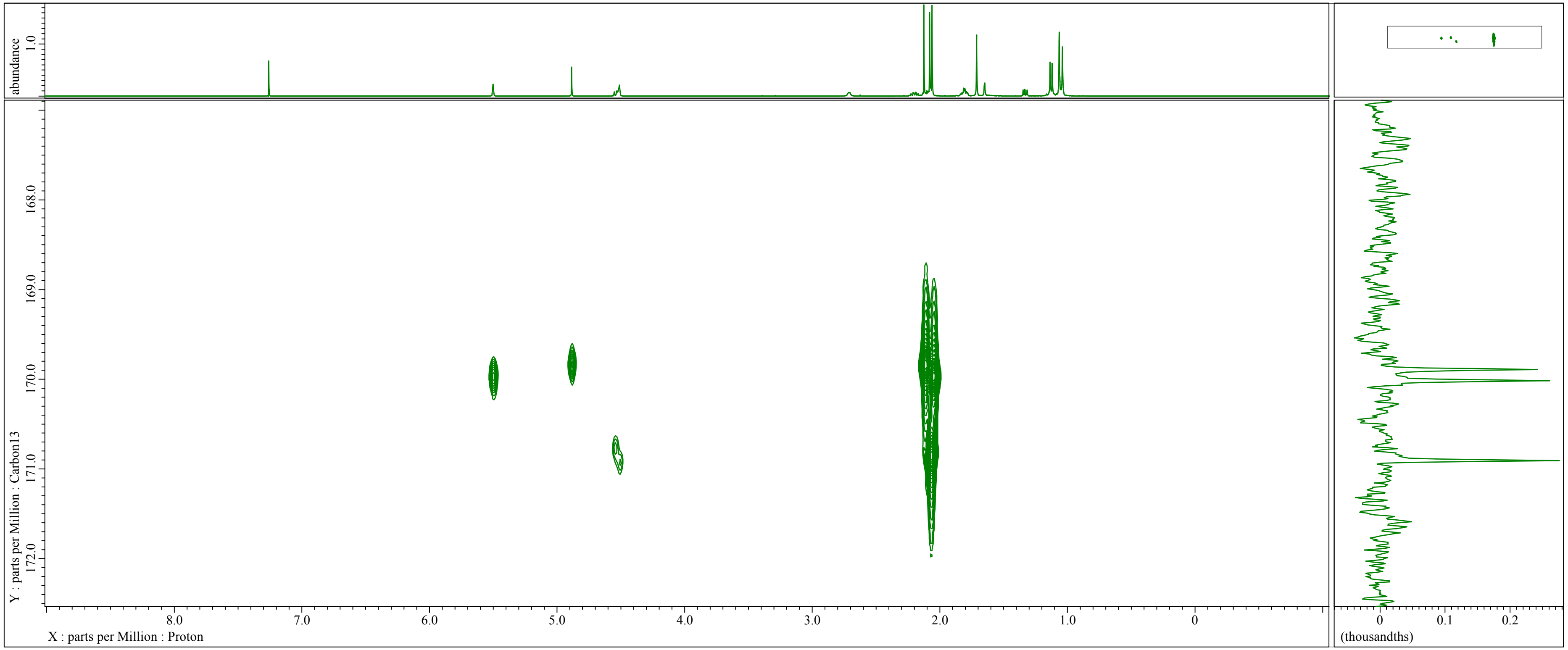

Figure S34. The band-selective HMBC (region 167–172 ppm) (400/100 MHz, $CDCl_3$) spectrum of compound **3**

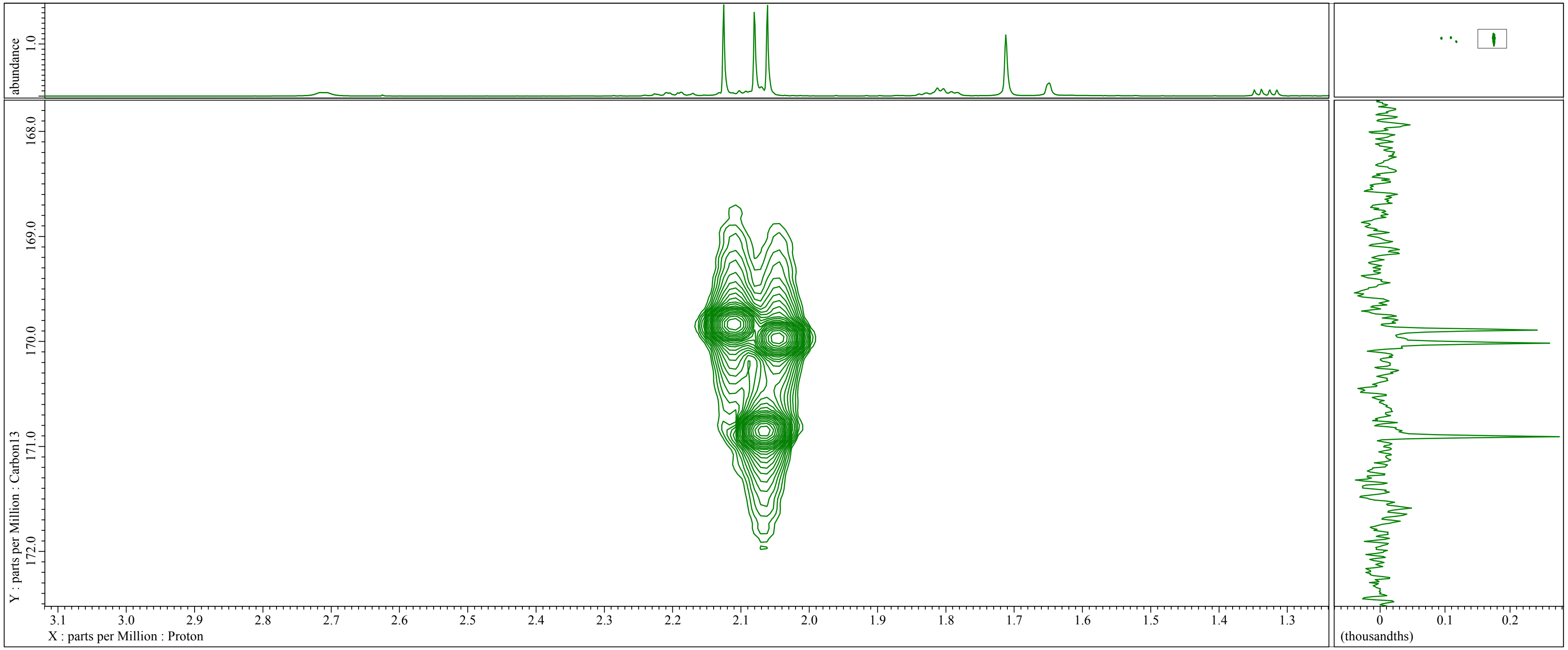

Figure S35. The inset (methyl region of the band-selective HMBC) (400/100 MHz, $CDCl_3$) spectrum of compound **3**

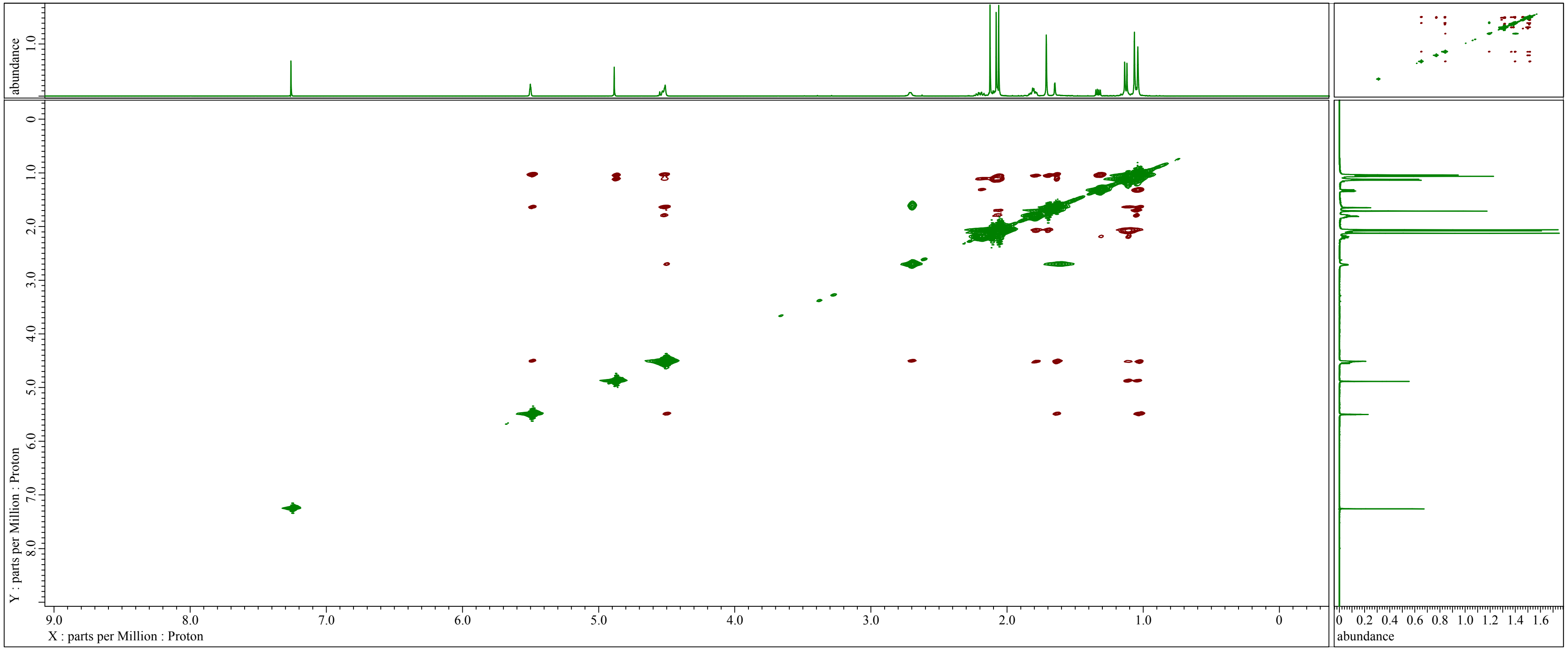

Figure S36. The NOESY (400 MHz, $CDCl_3$) spectrum of compound **3**

8. MS and NMR data for compound 4

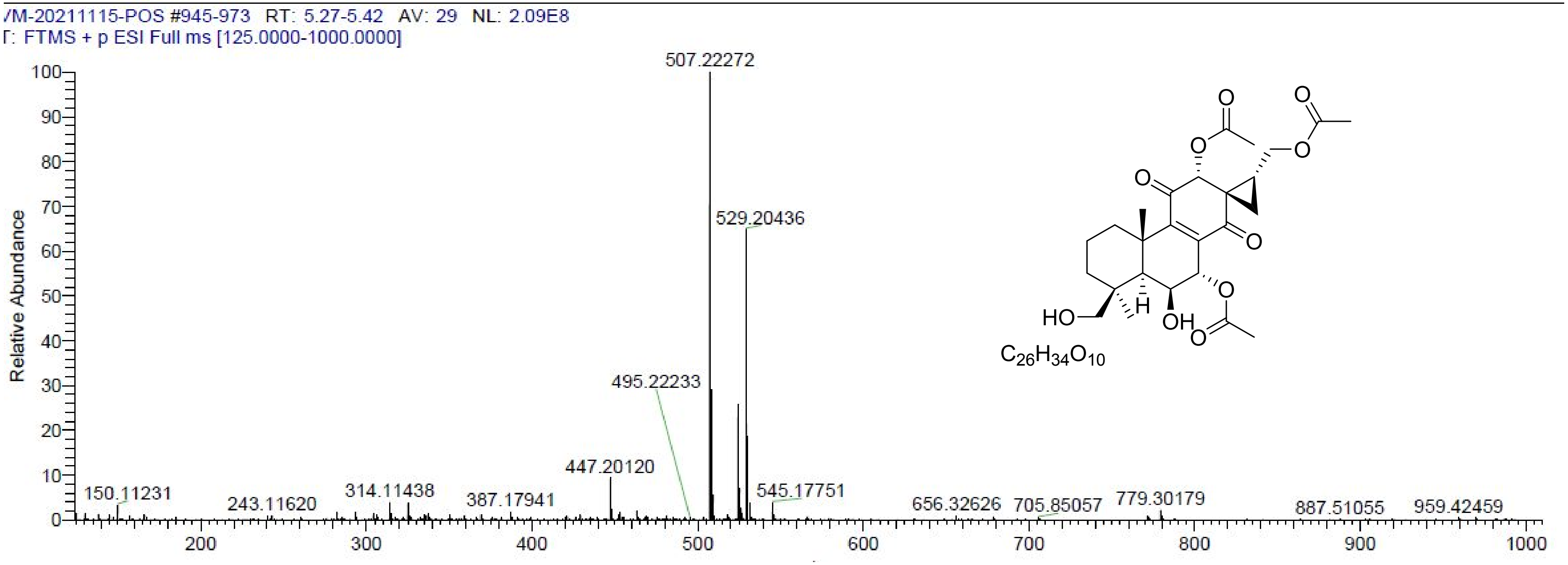

Figure S37. The HRESIMS spectrum of compound **4** (positive mode)

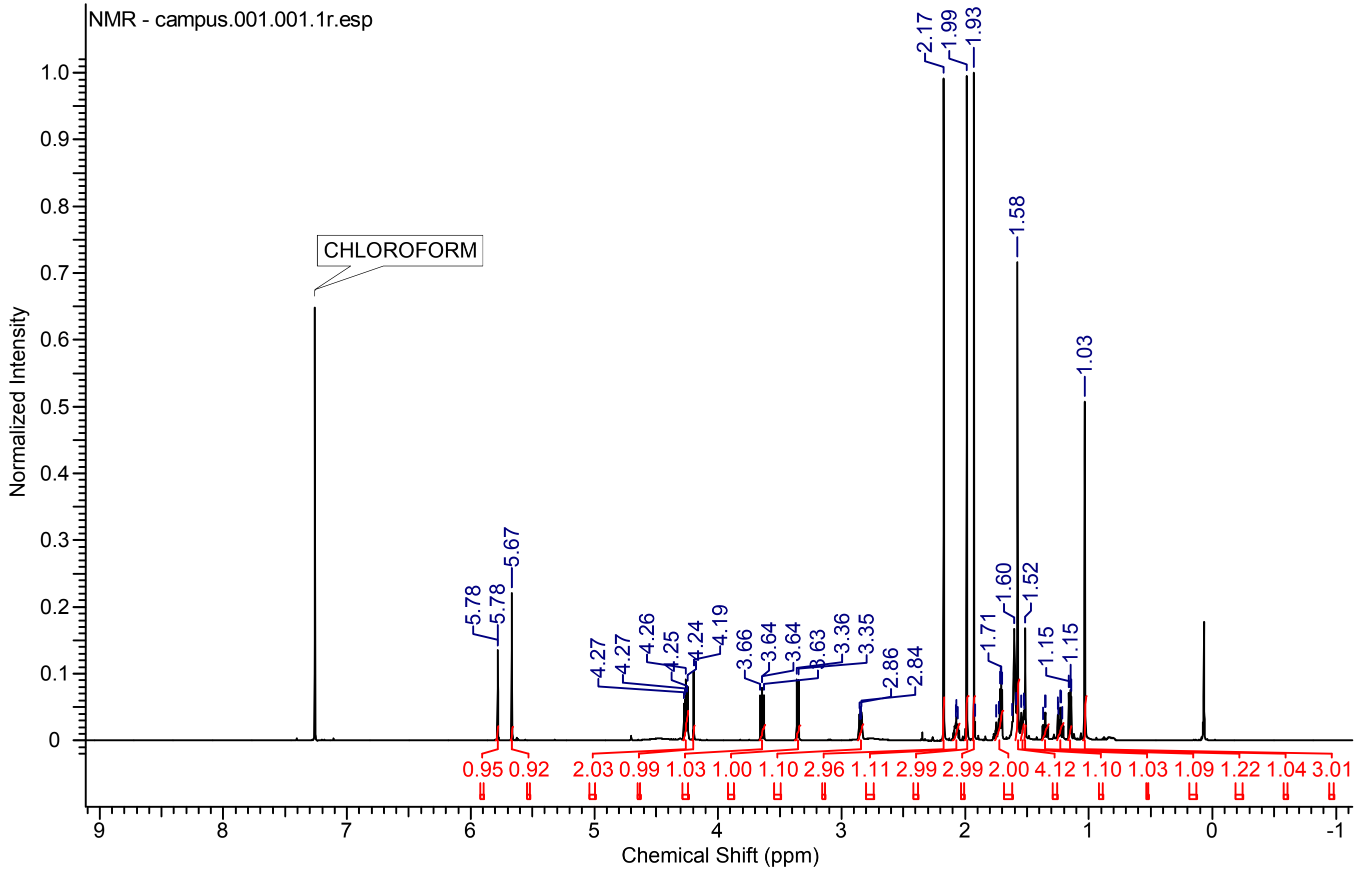

Figure S38. The ^{1}H NMR (700 MHz, $CDCl_3$) spectrum of compound **4**

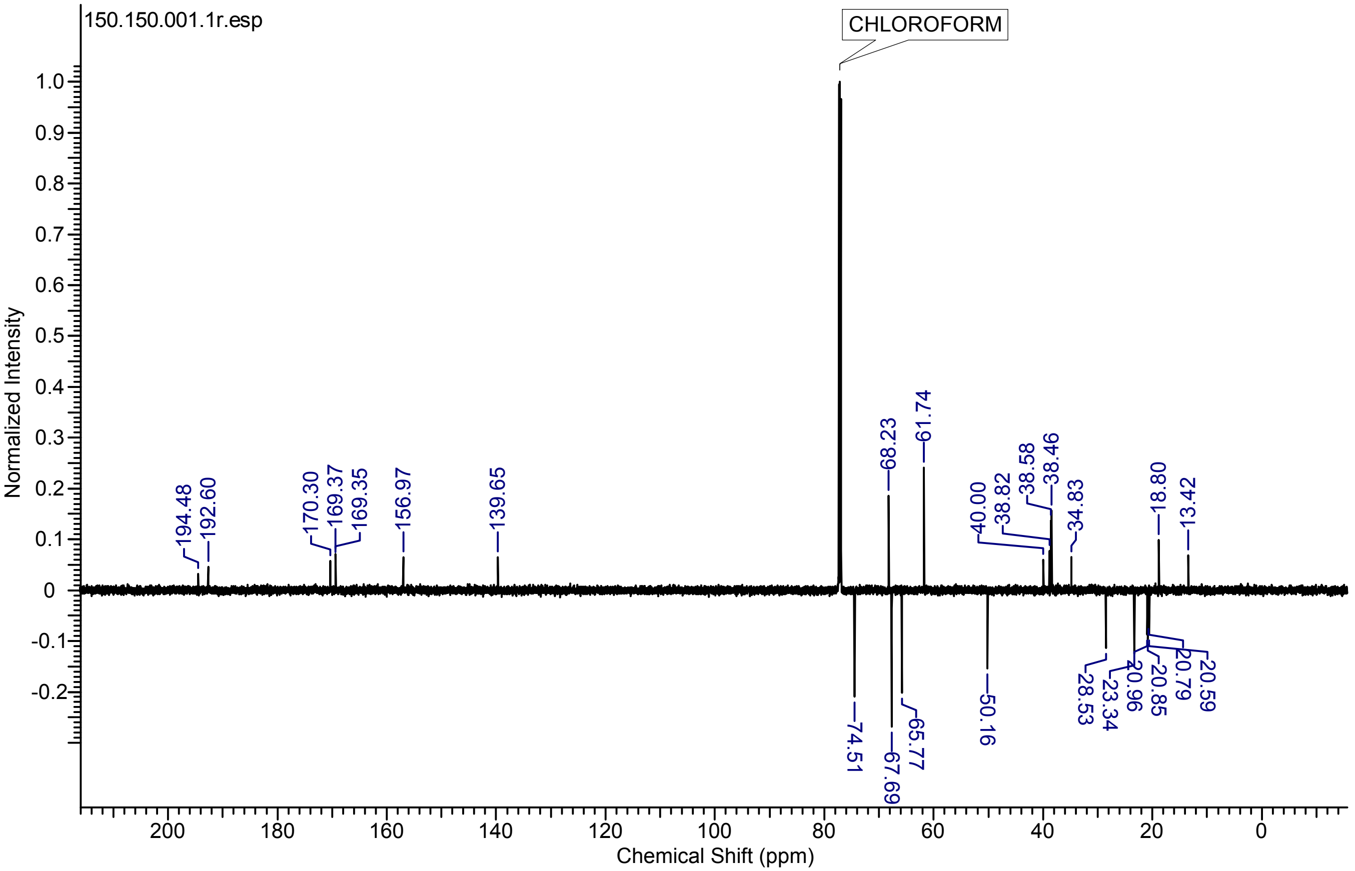

Figure S39. The ^{13}C NMR APT (175 MHz, $CDCl_3$) spectrum of compound **4**

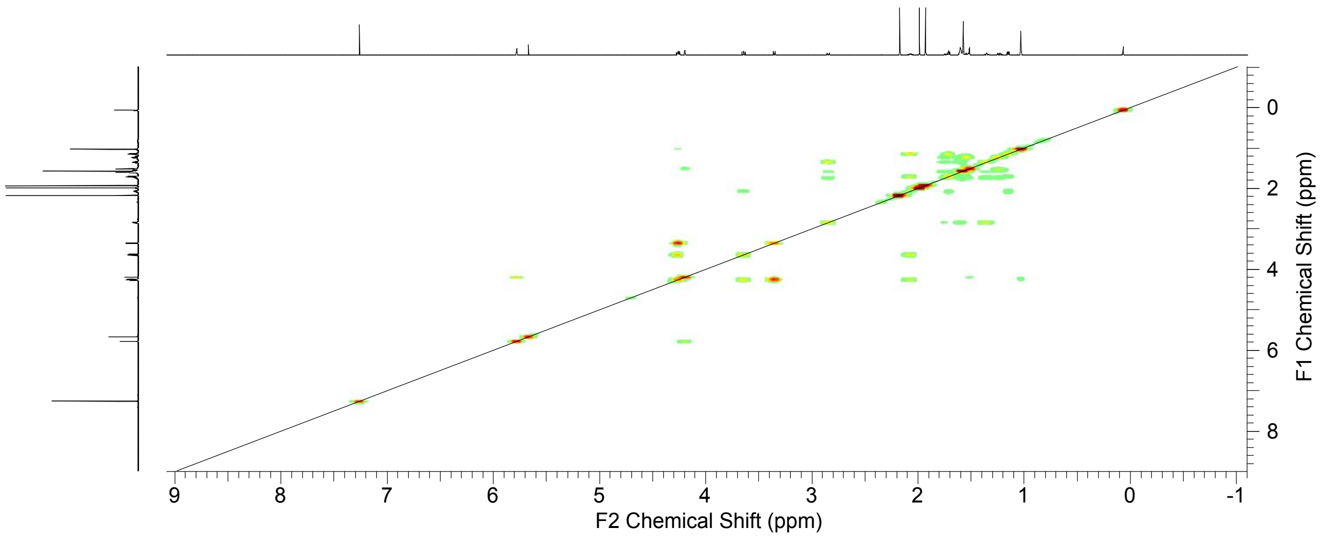

Figure S40. The COSY (700 MHz, $CDCl_3$) spectrum of compound **4**

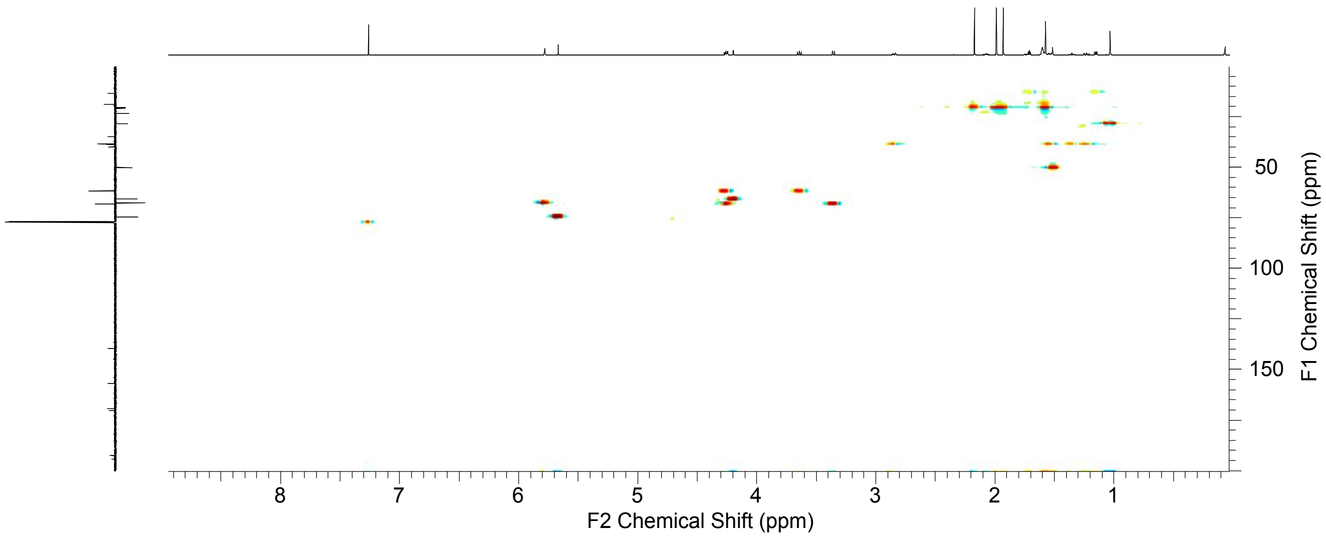

Figure S41. The HSQC (700/175 MHz, $CDCl_3$) spectrum of compound **4**

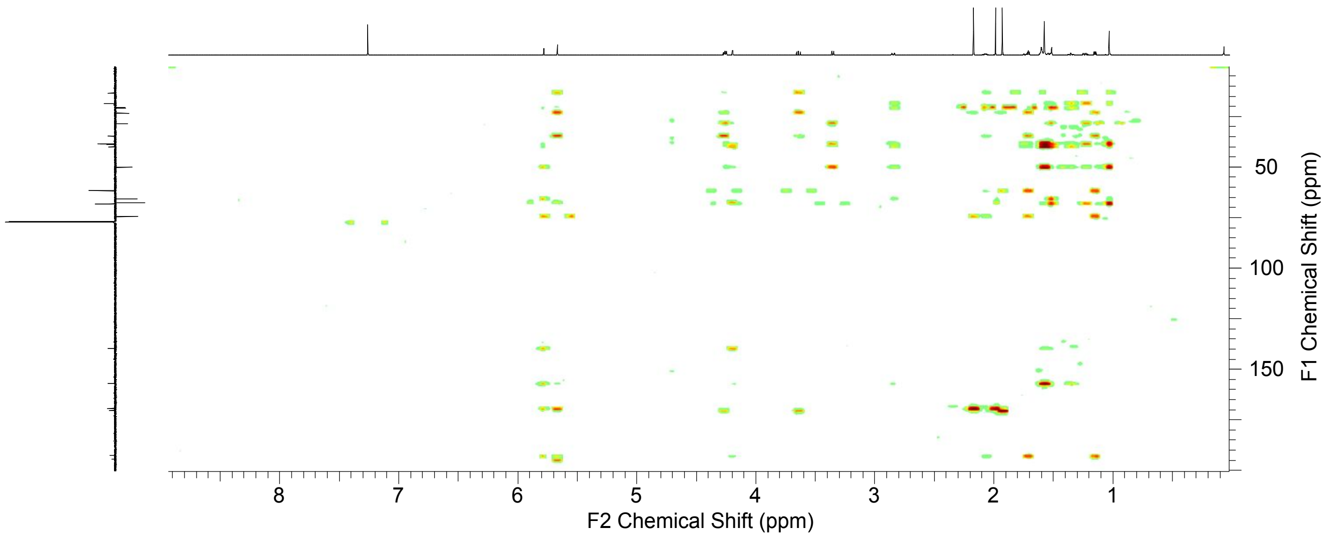

Figure S42. The HMBC (700/175 MHz, $CDCl_3$) spectrum of compound **4**

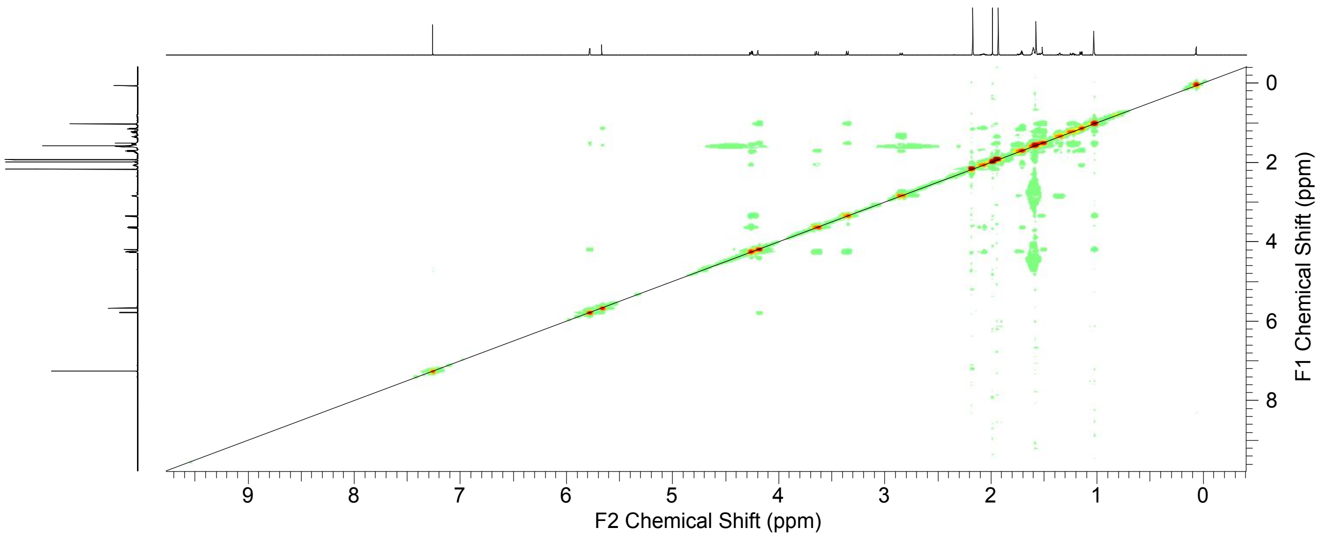

Figure S43. The NOESY (700 MHz, $CDCl_3$) spectrum of compound **4**

9. MS and NMR data for compound 5

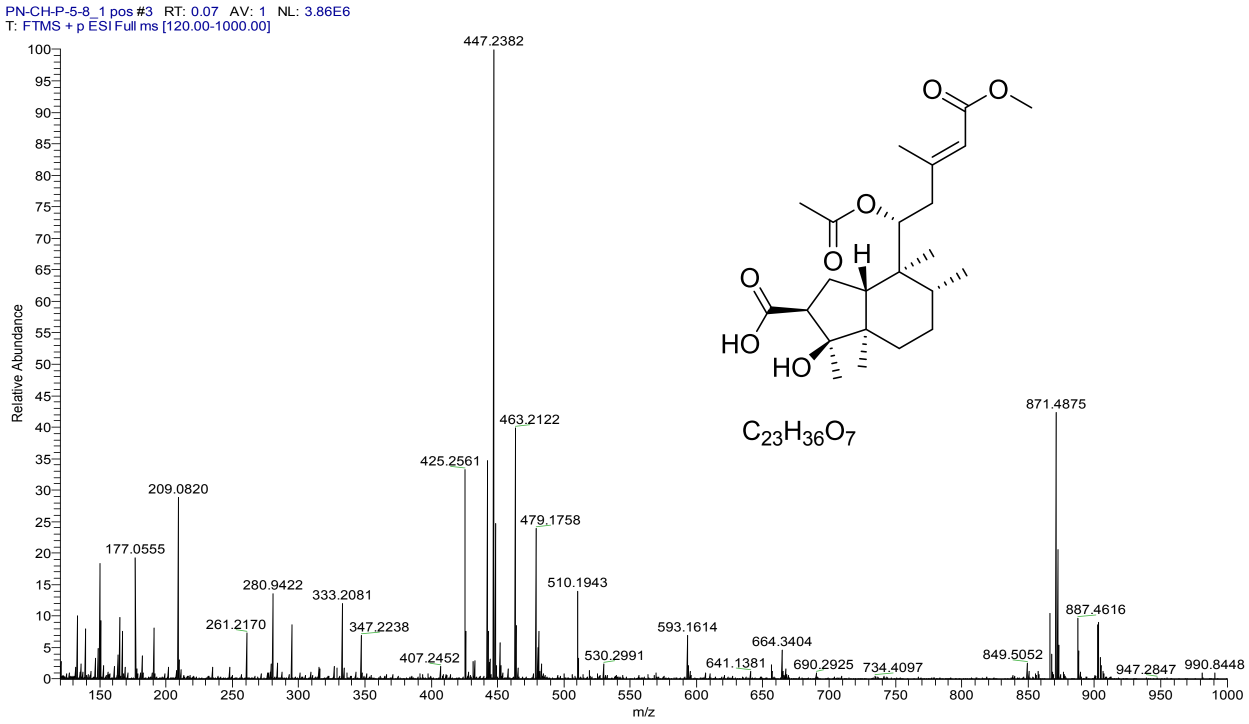

Figure S44. The HRESIMS spectrum of compound **5** (positive mode)

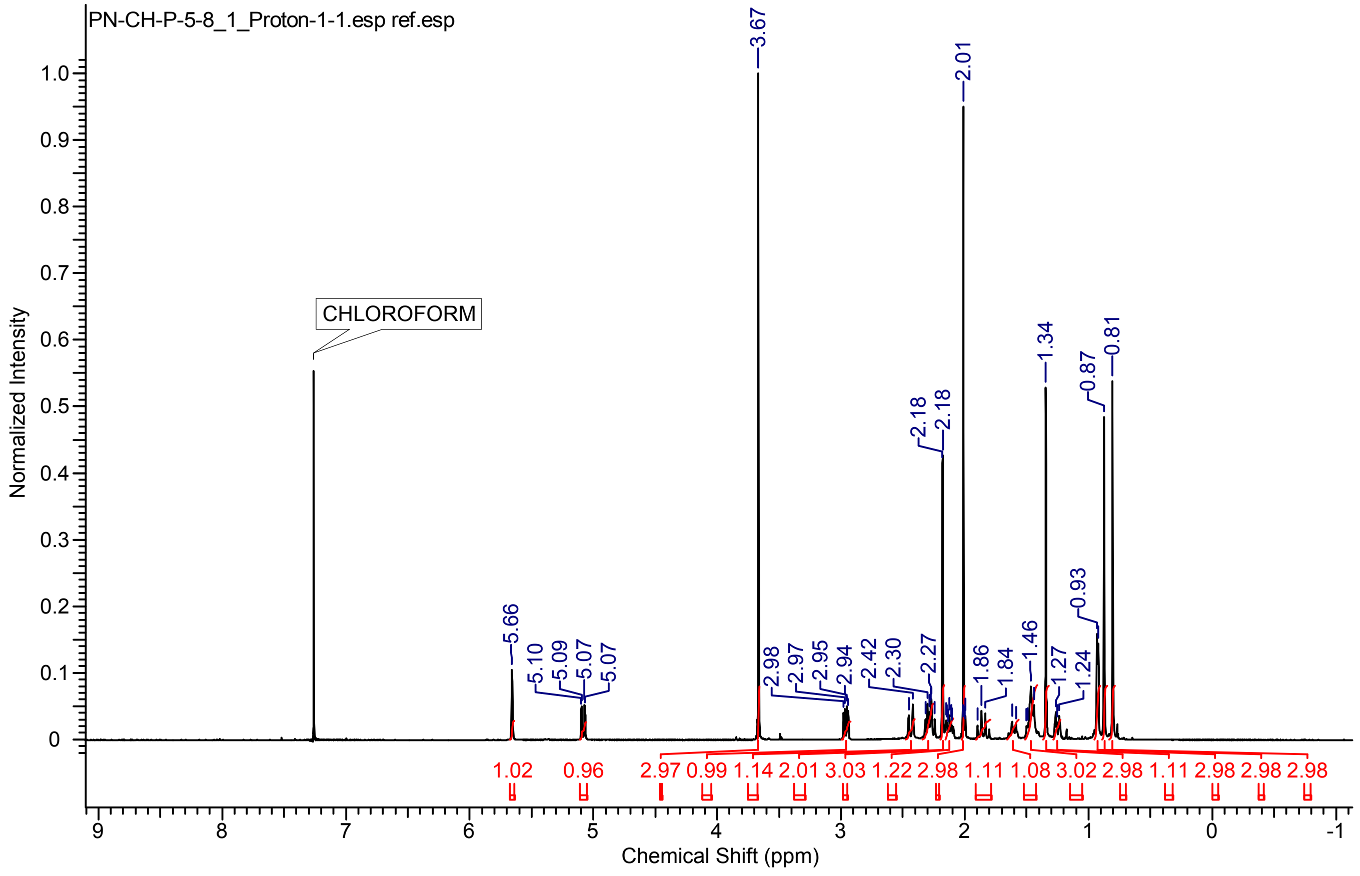

Figure S45. The ^{1}H NMR (400 MHz, $CDCl_3$) spectrum of compound **5**

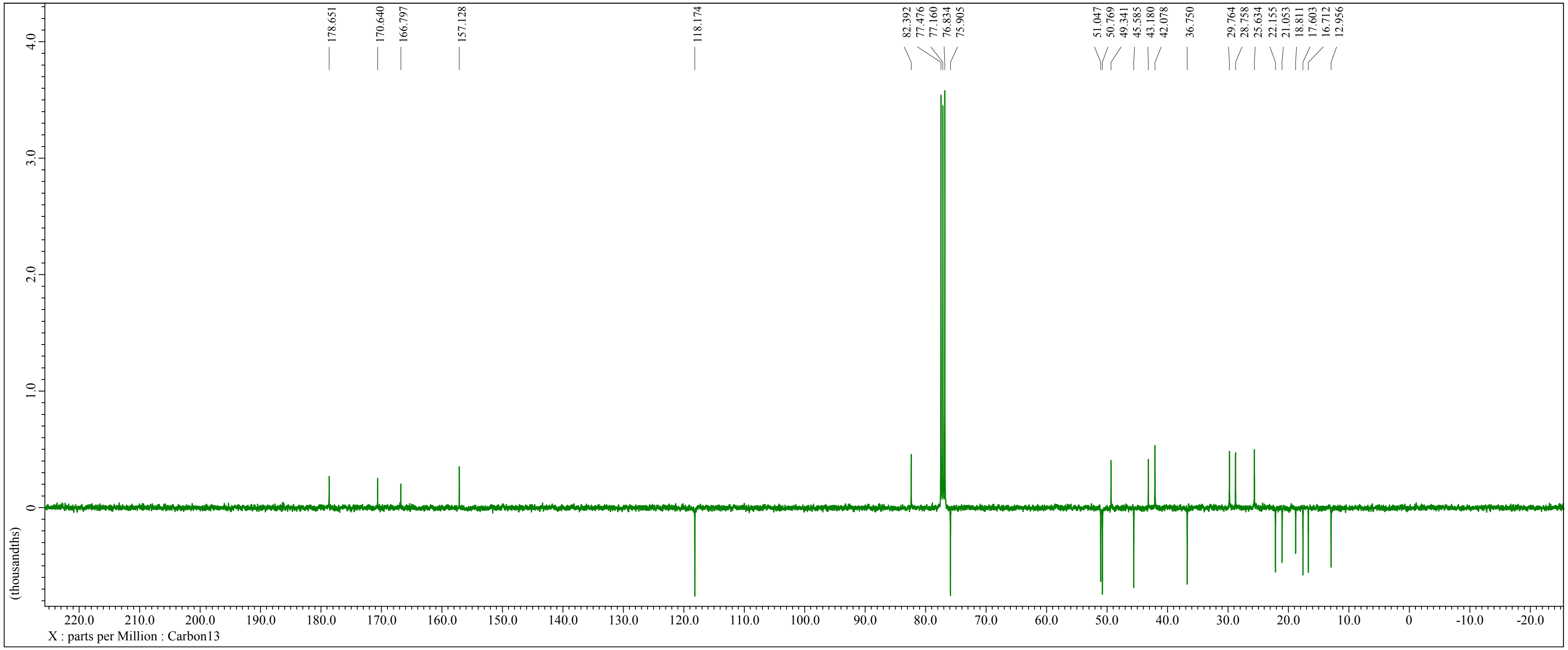

Figure S46. The ^{13}C NMR APT (100 MHz, $CDCl_3$) spectrum of compound **5**

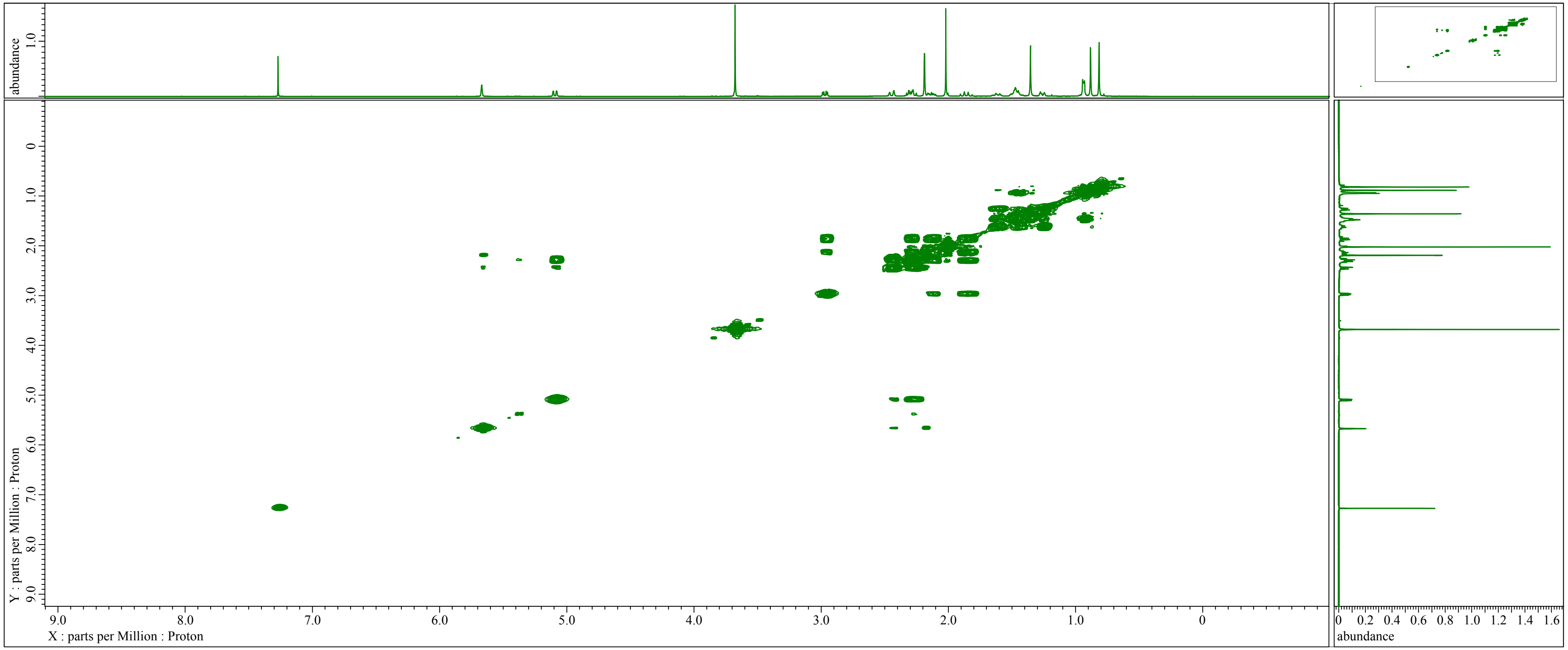

Figure S47. The COSY (400 MHz, $CDCl_3$) spectrum of compound **5**

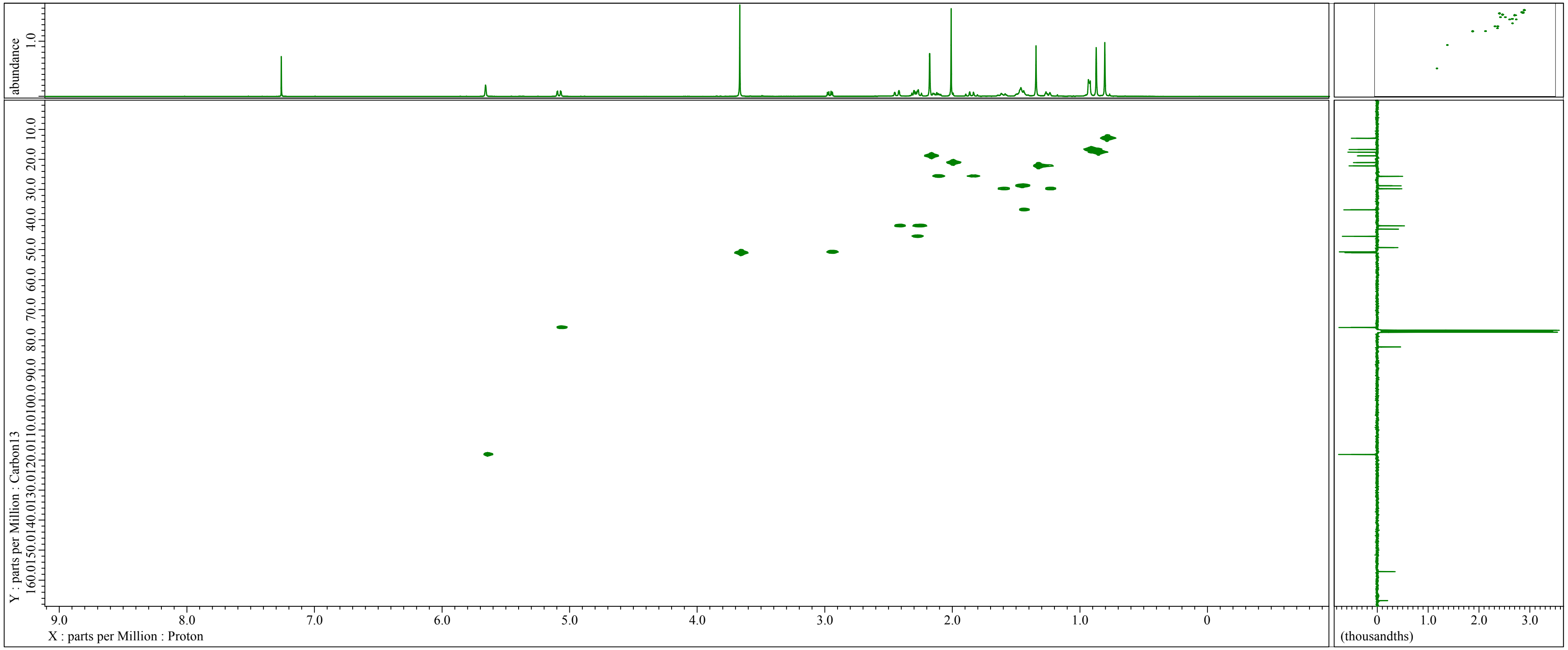

Figure S48. The HSQC (400/100 MHz, $CDCl_3$) spectrum of compound **5**

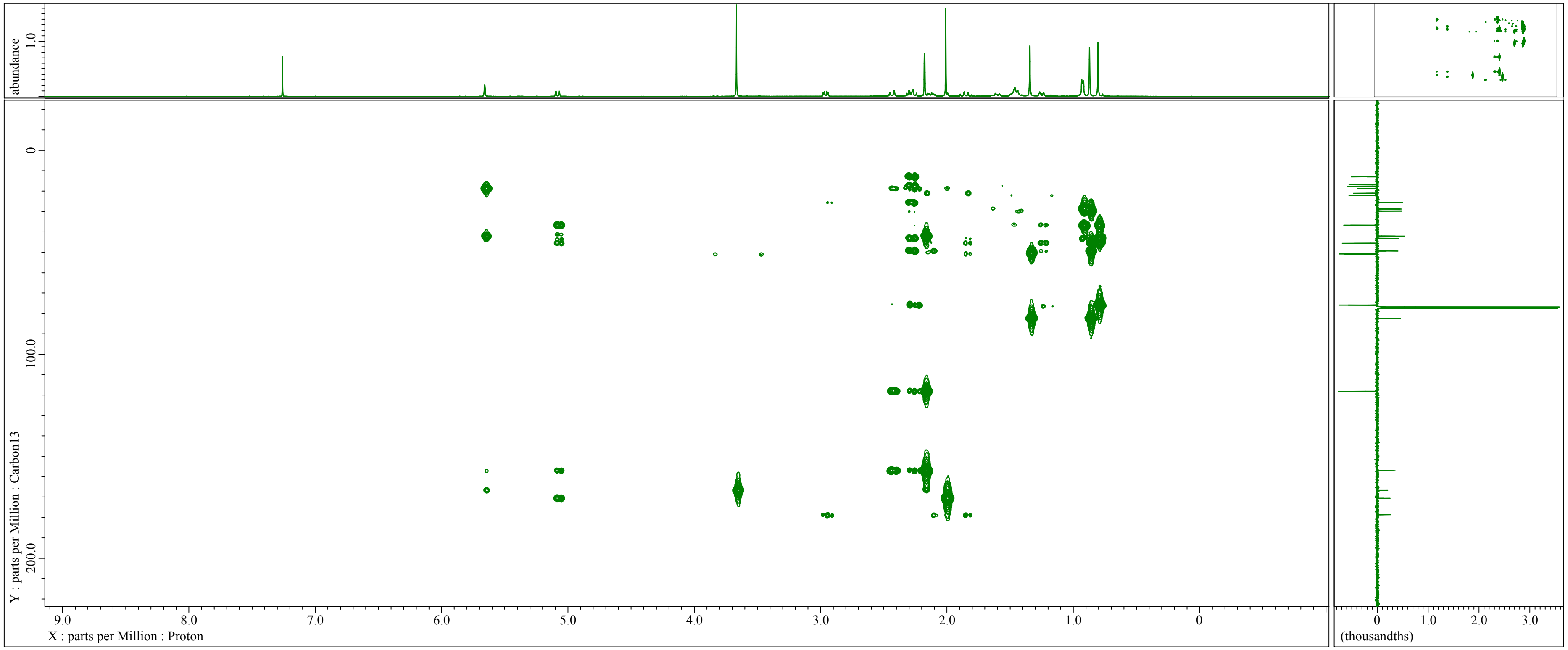

Figure S49. The HMBC (400/100 MHz, $CDCl_3$) spectrum of compound **5**

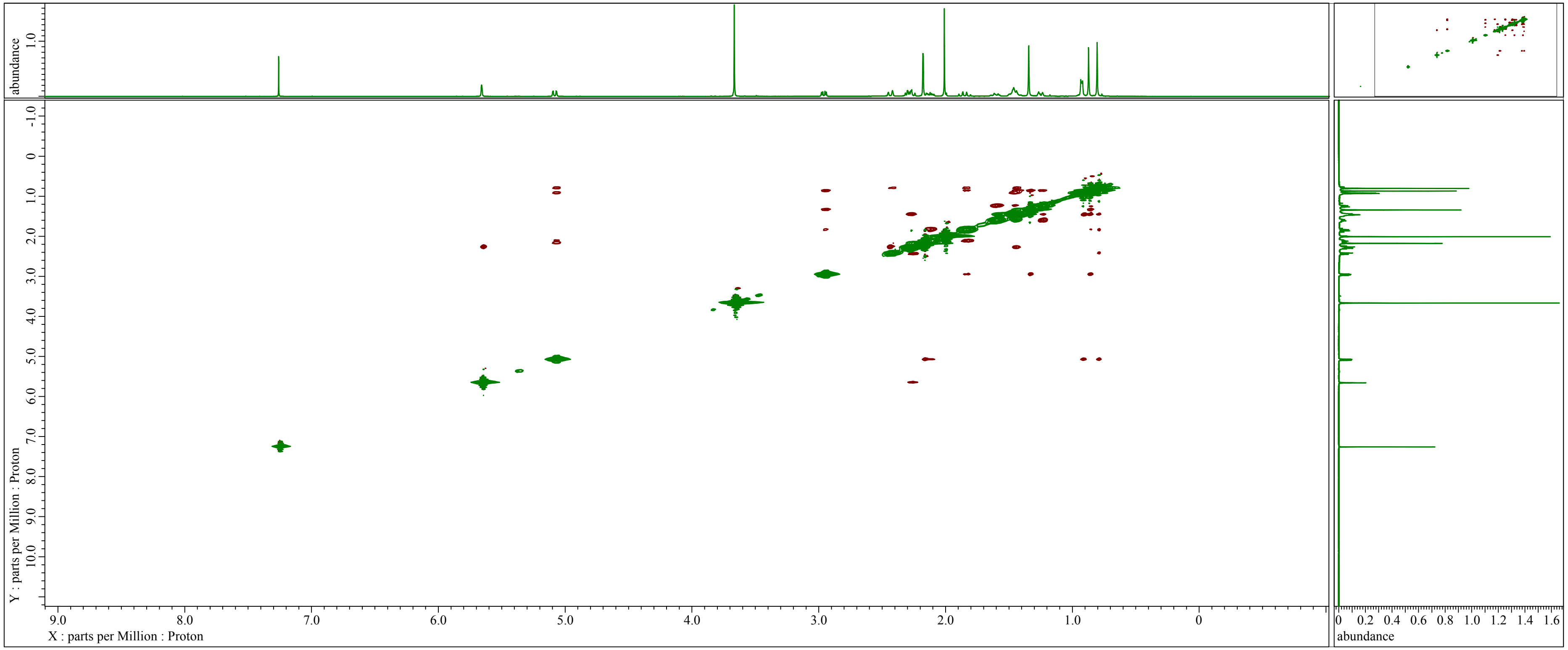

Figure S50. The NOESY (400 MHz, $CDCl_3$) spectrum of compound **5**

10. MS and NMR data for compound 6

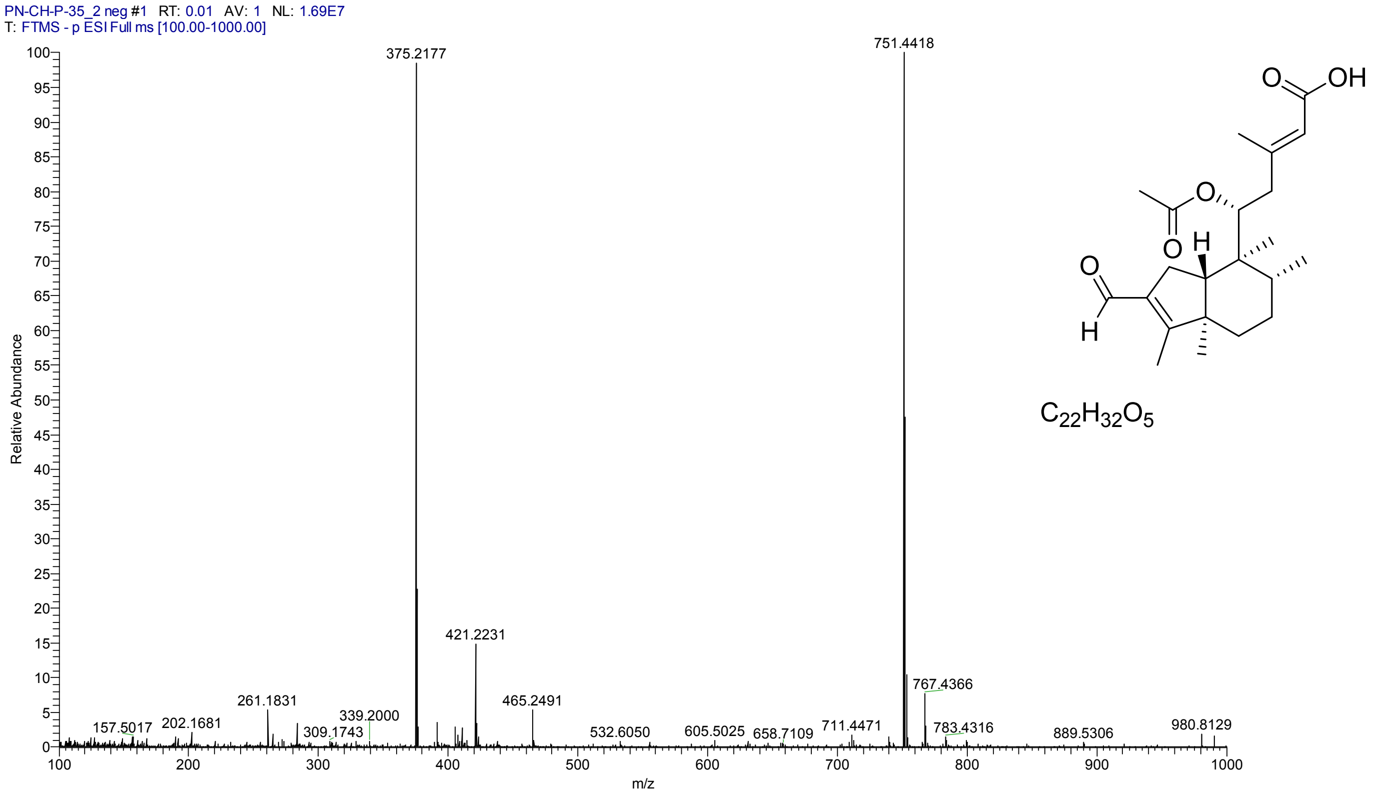

Figure S51. The HRESIMS spectrum of compound **6** (negative mode)

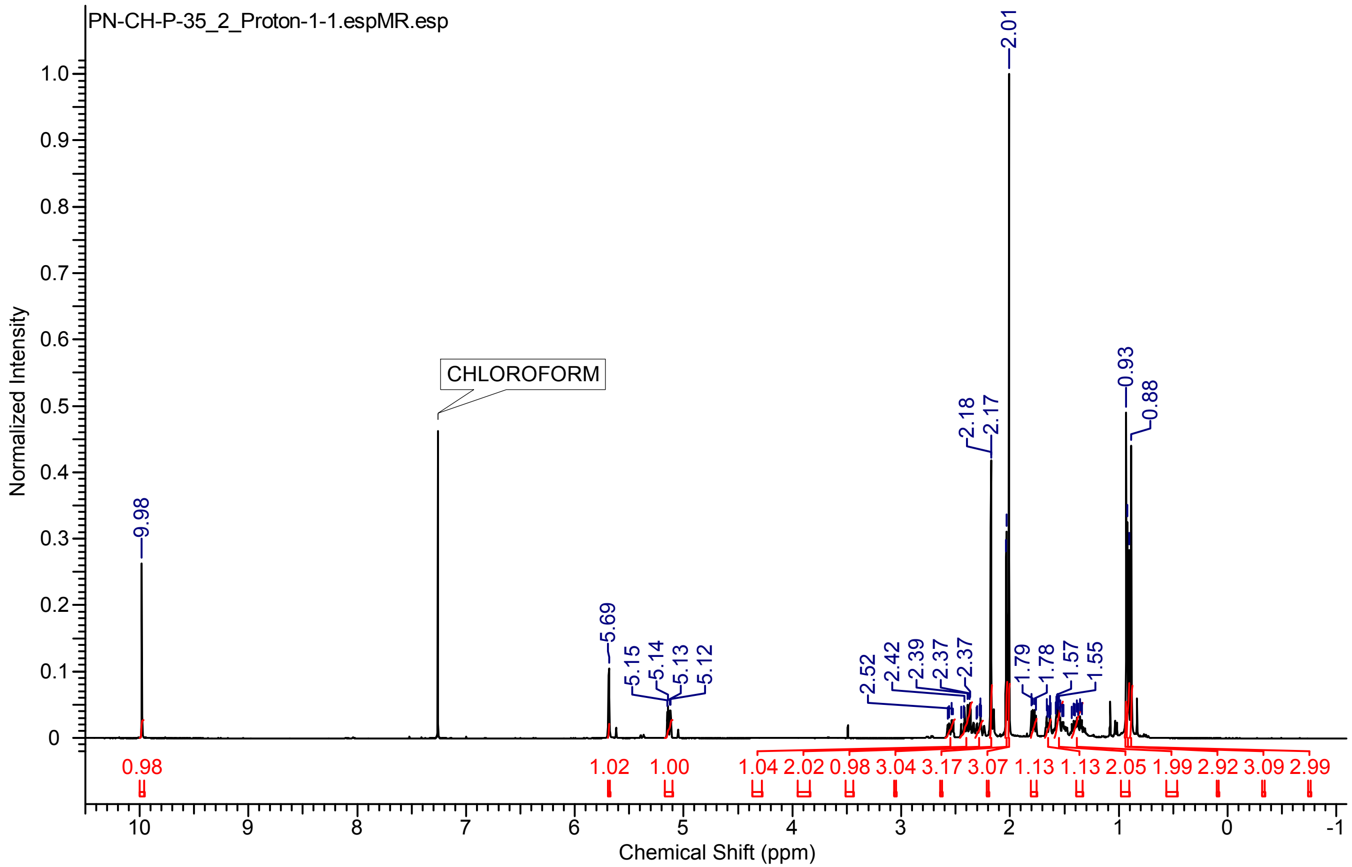

Figure S52. The ^{1}H NMR (400 MHz, $CDCl_3$) spectrum of compound **6**

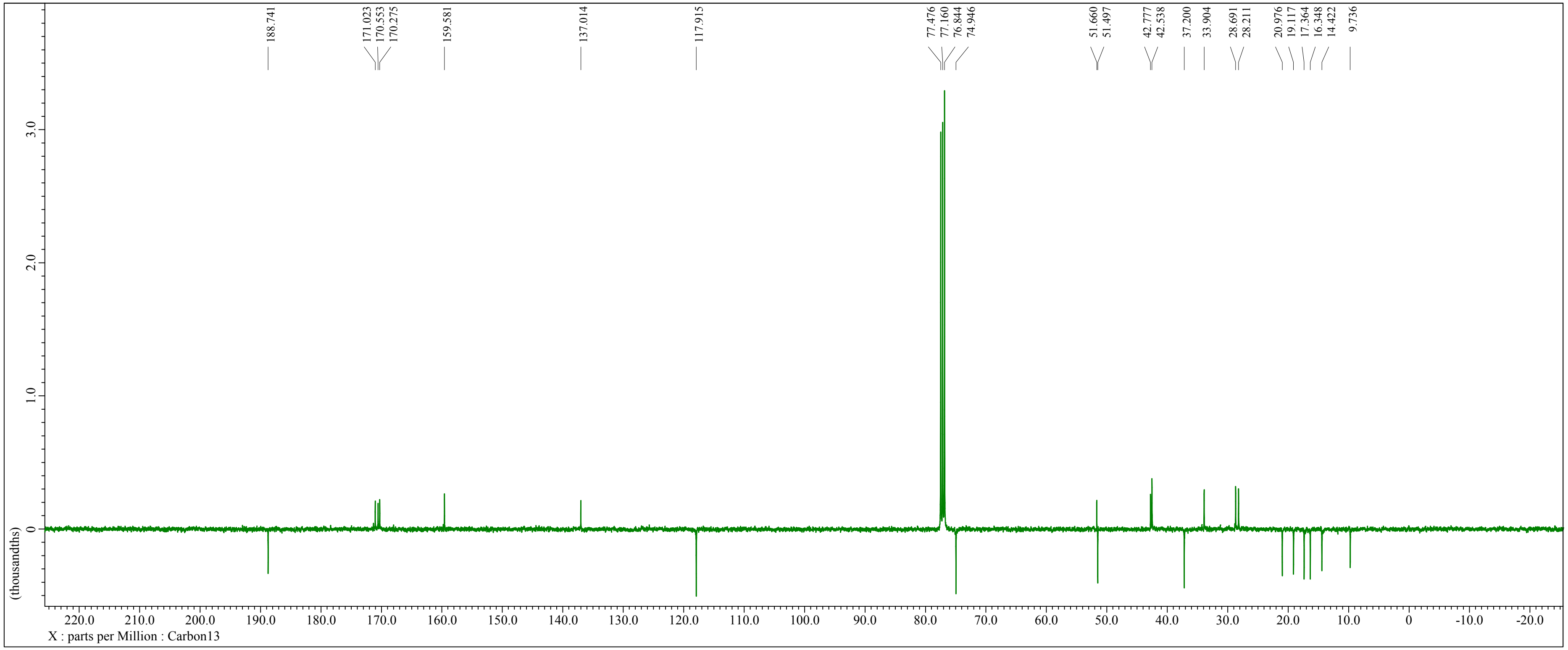

Figure S53. The ^{13}C NMR APT (100 MHz, $CDCl_3$) spectrum of compound **6**

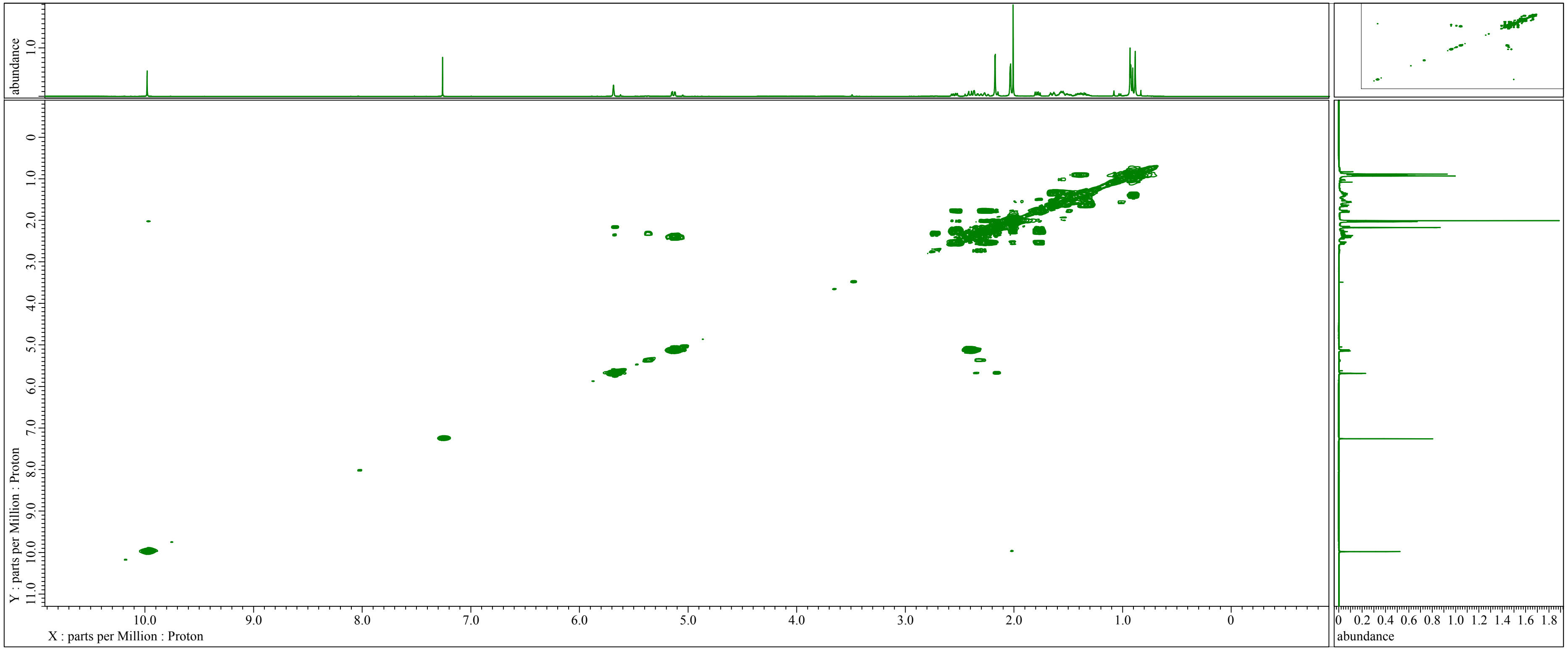

Figure S54. The COSY (400 MHz, $CDCl_3$) spectrum of compound **6**

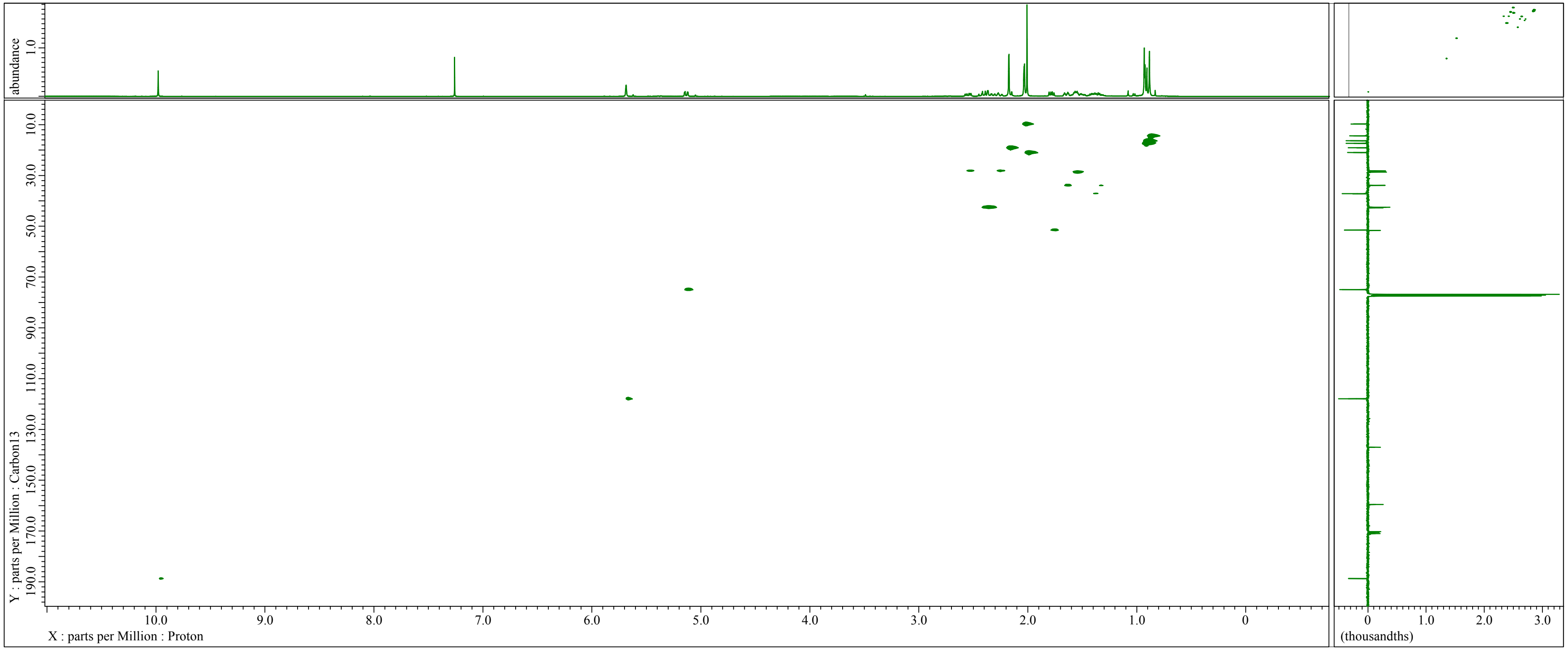

Figure S55. The HSQC (400/100 MHz, $CDCl_3$) spectrum of compound **6**

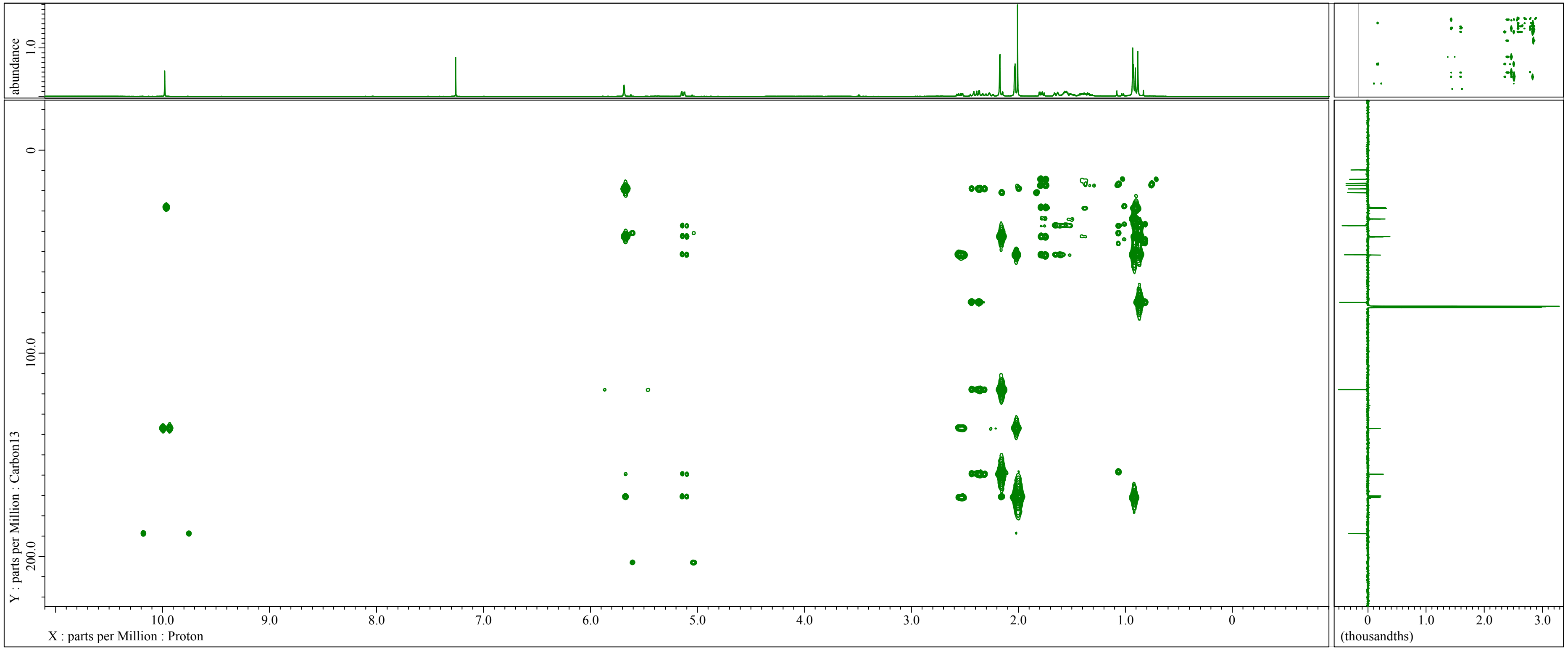

Figure S56. The HMBC (400/100 MHz, $CDCl_3$) spectrum of compound **6**

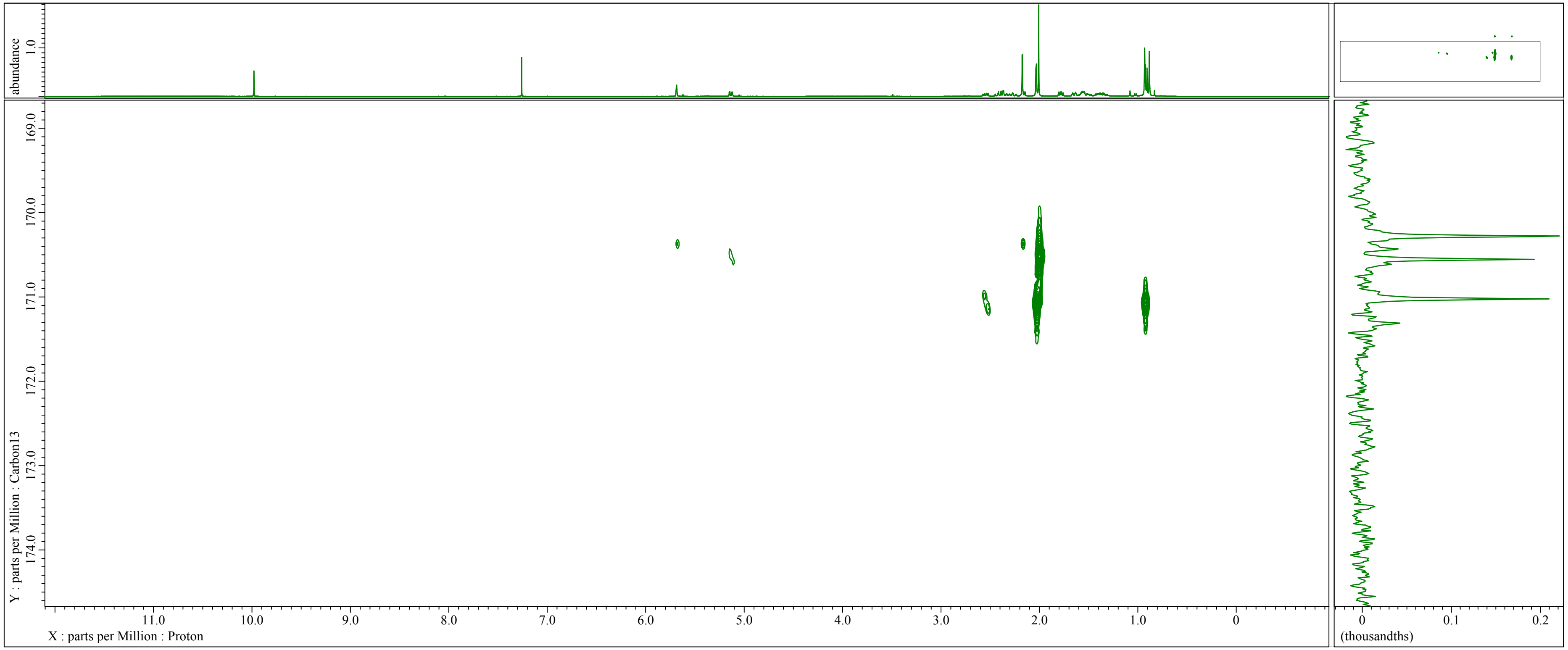

Figure S57. The band-selective HMBC (400/100 MHz, $CDCl_3$) spectrum of compound **6**

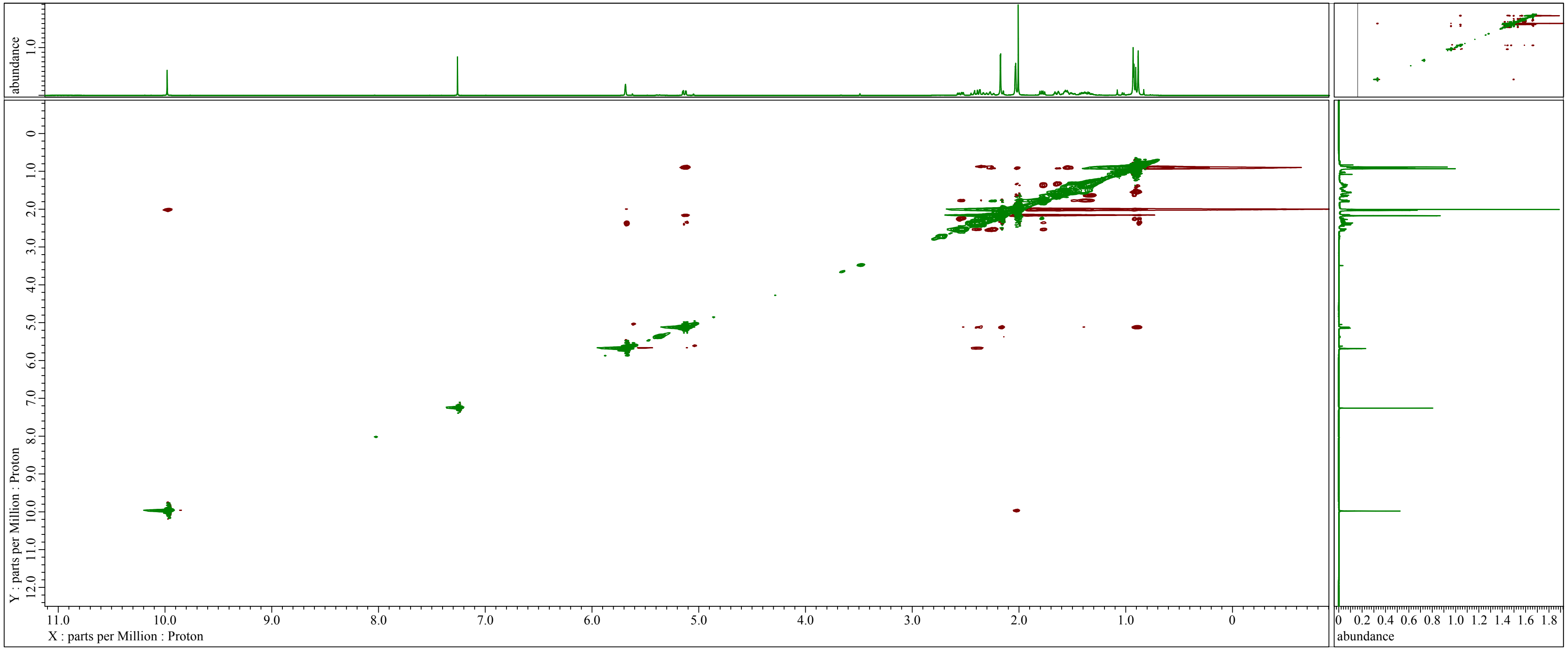

Figure S58. The NOESY (400 MHz, $CDCl_3$) spectrum of compound **6**

11. MS and NMR data for compound 7

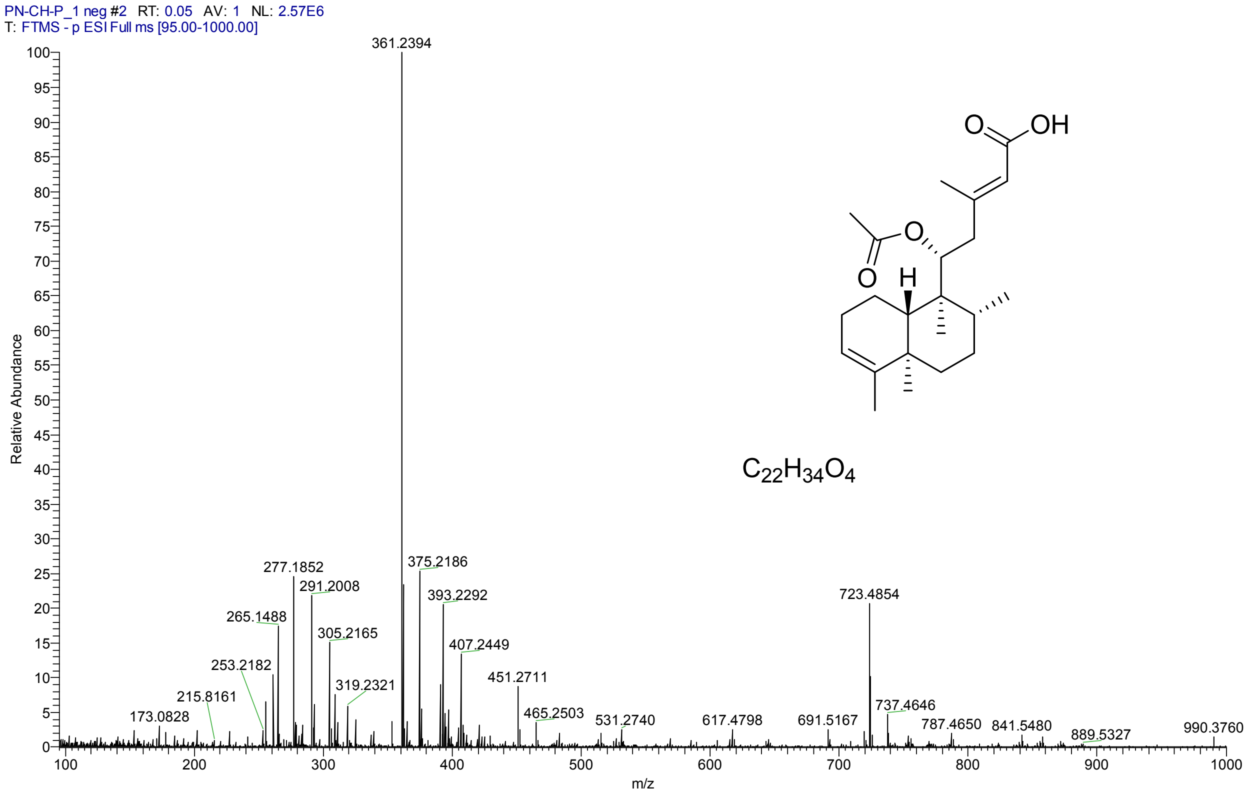

Figure S59. The HRESIMS spectrum of compound **7** (negative mode)

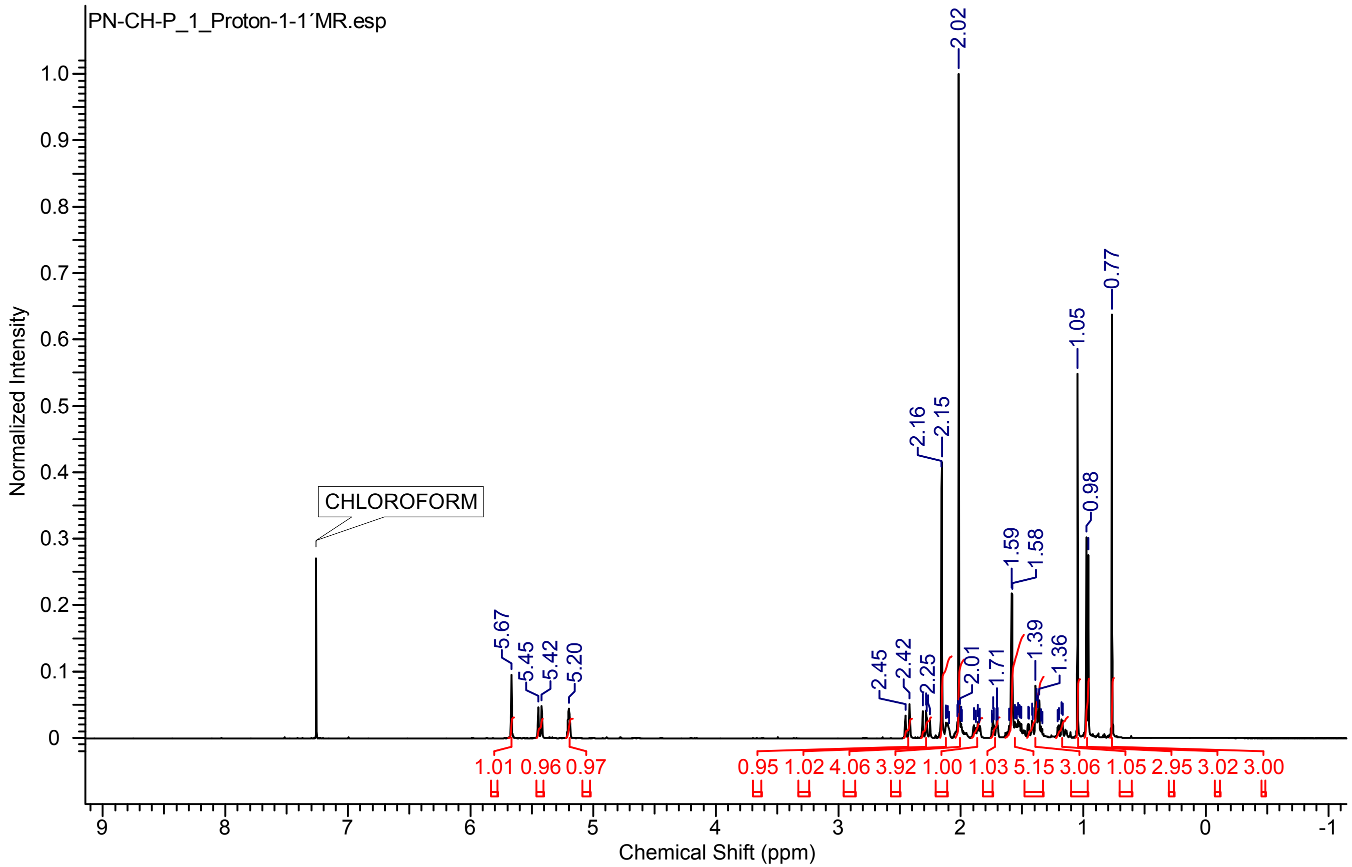

Figure S60. The ^{1}H NMR (400 MHz, $CDCl_3$) spectrum of compound **7**

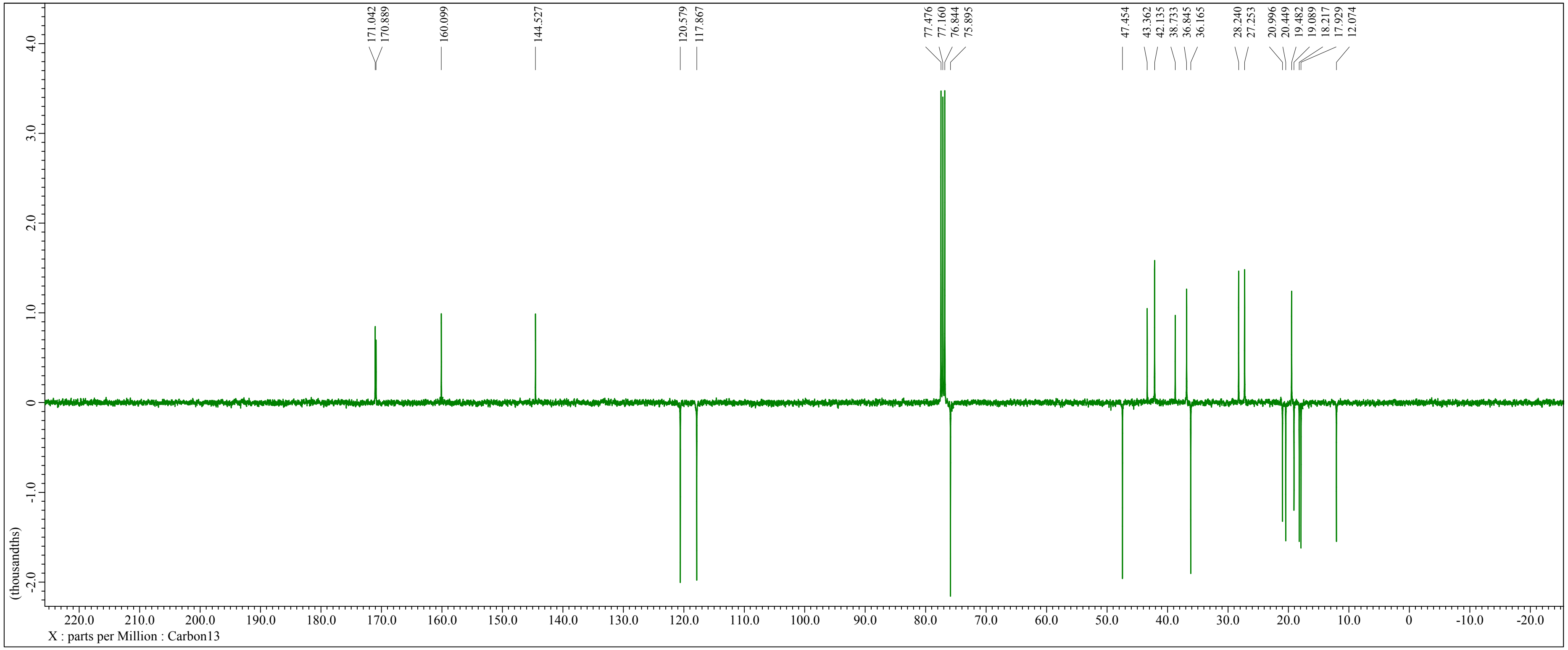

Figure S61. The ^{13}C NMR APT (100 MHz, $CDCl_3$) spectrum of compound **7**

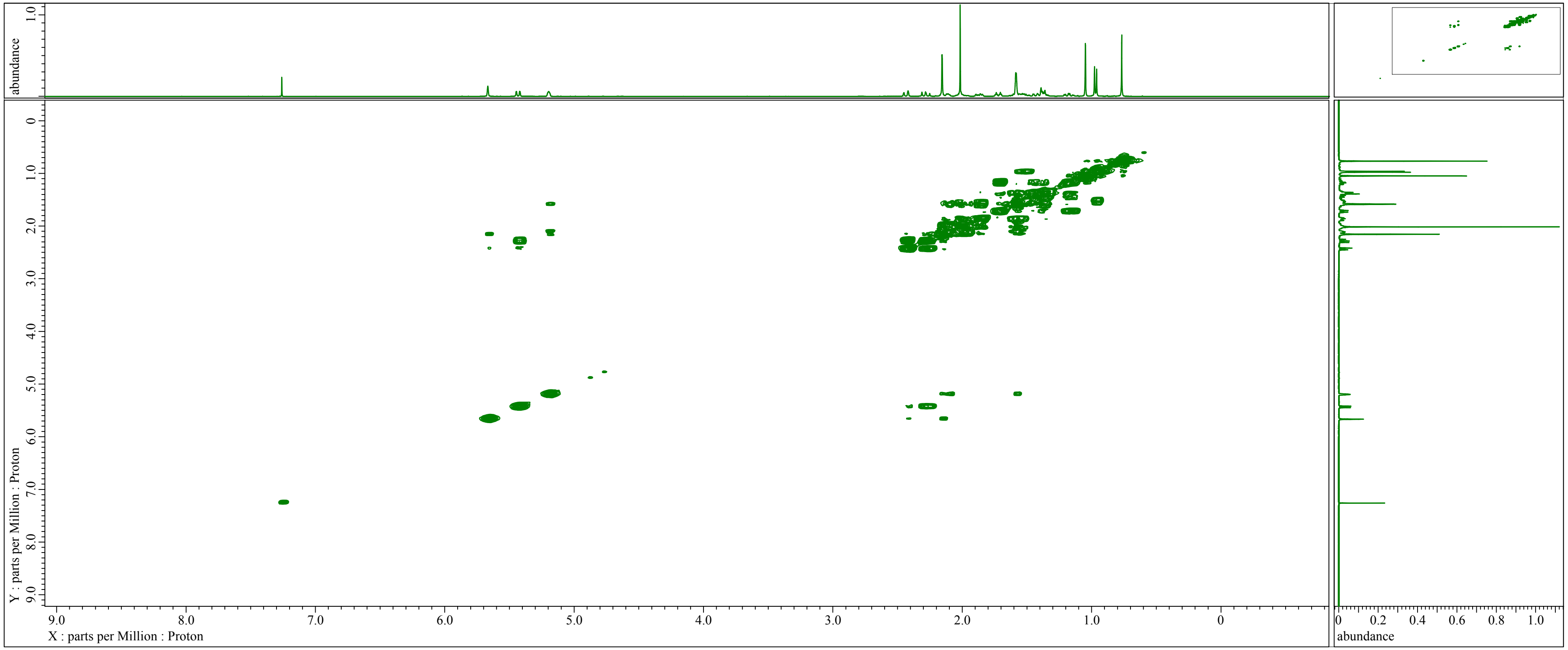

Figure S62. The COSY (400 MHz, $CDCl_3$) spectrum of compound **7**

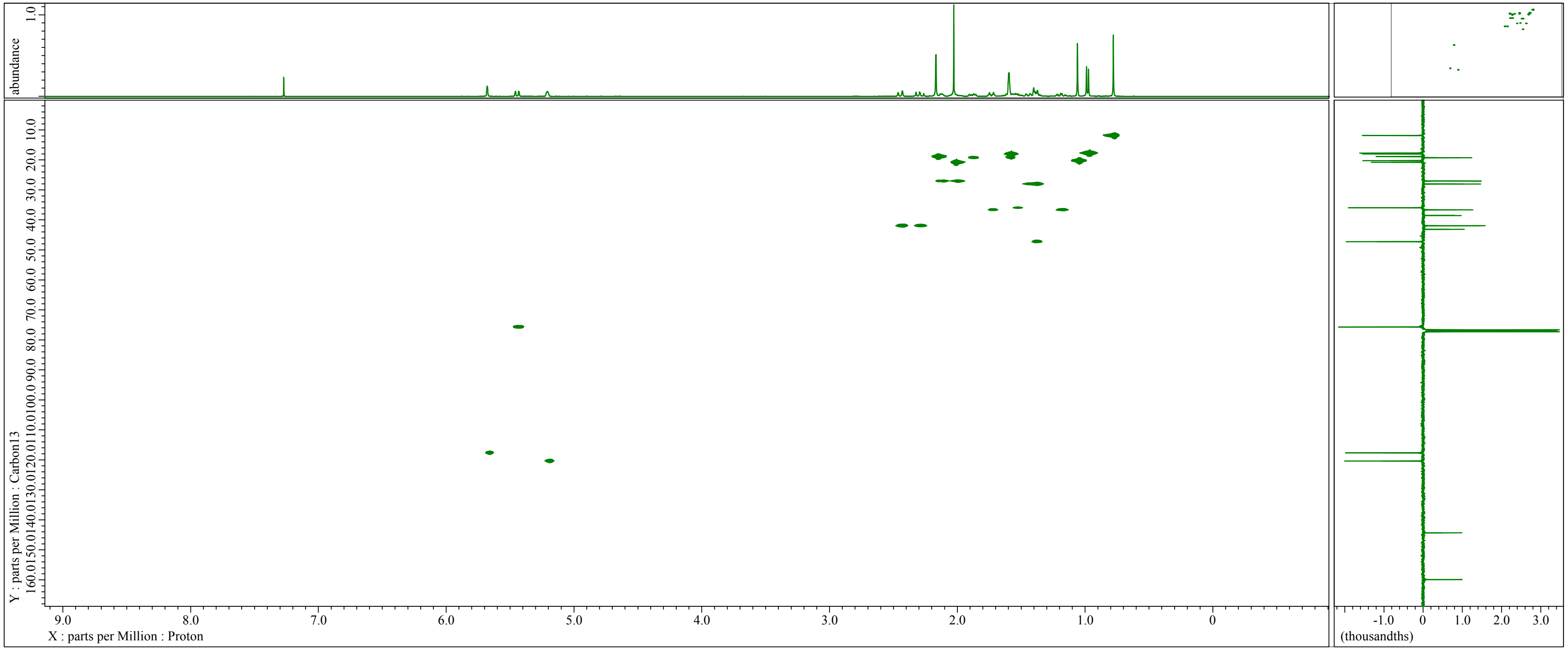

Figure S63. The HSQC (400/100 MHz, $CDCl_3$) spectrum of compound **7**

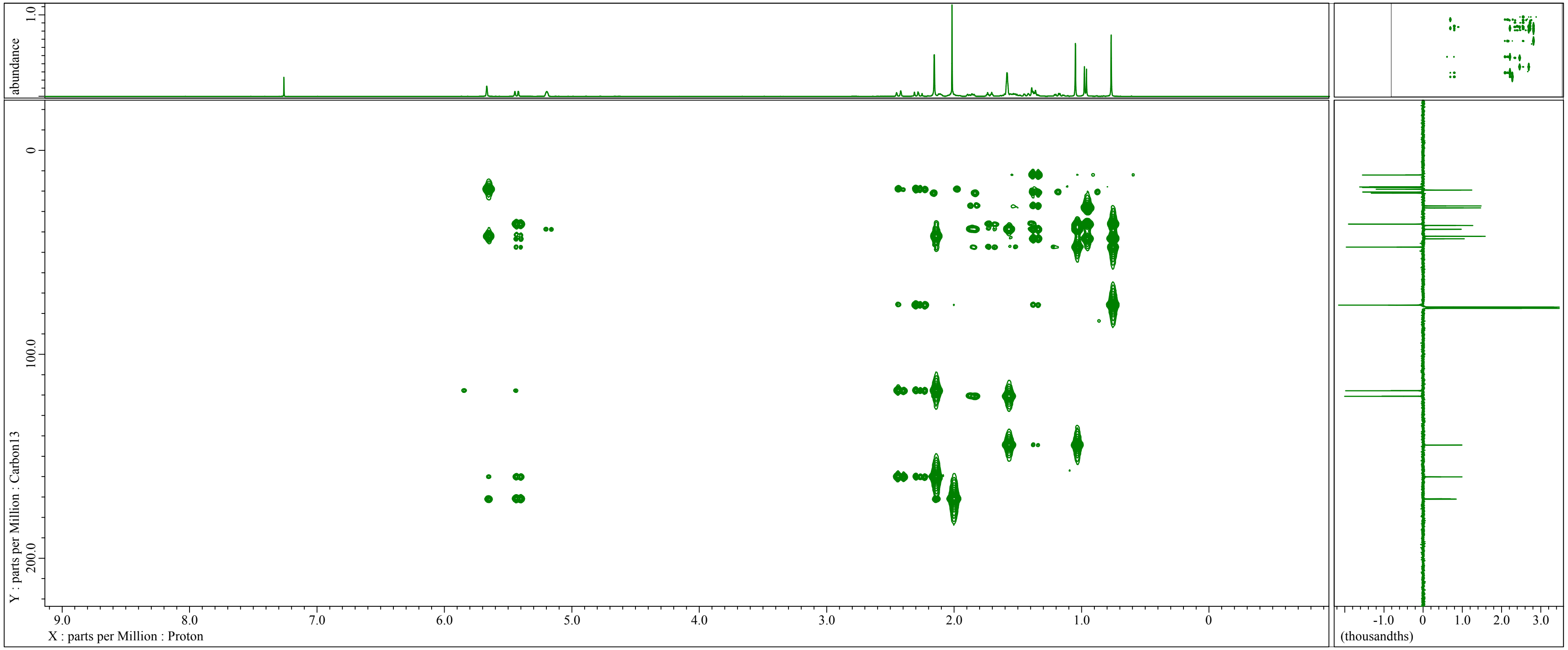

Figure S64. The HMBC (400/100 MHz, $CDCl_3$) spectrum of compound **7**

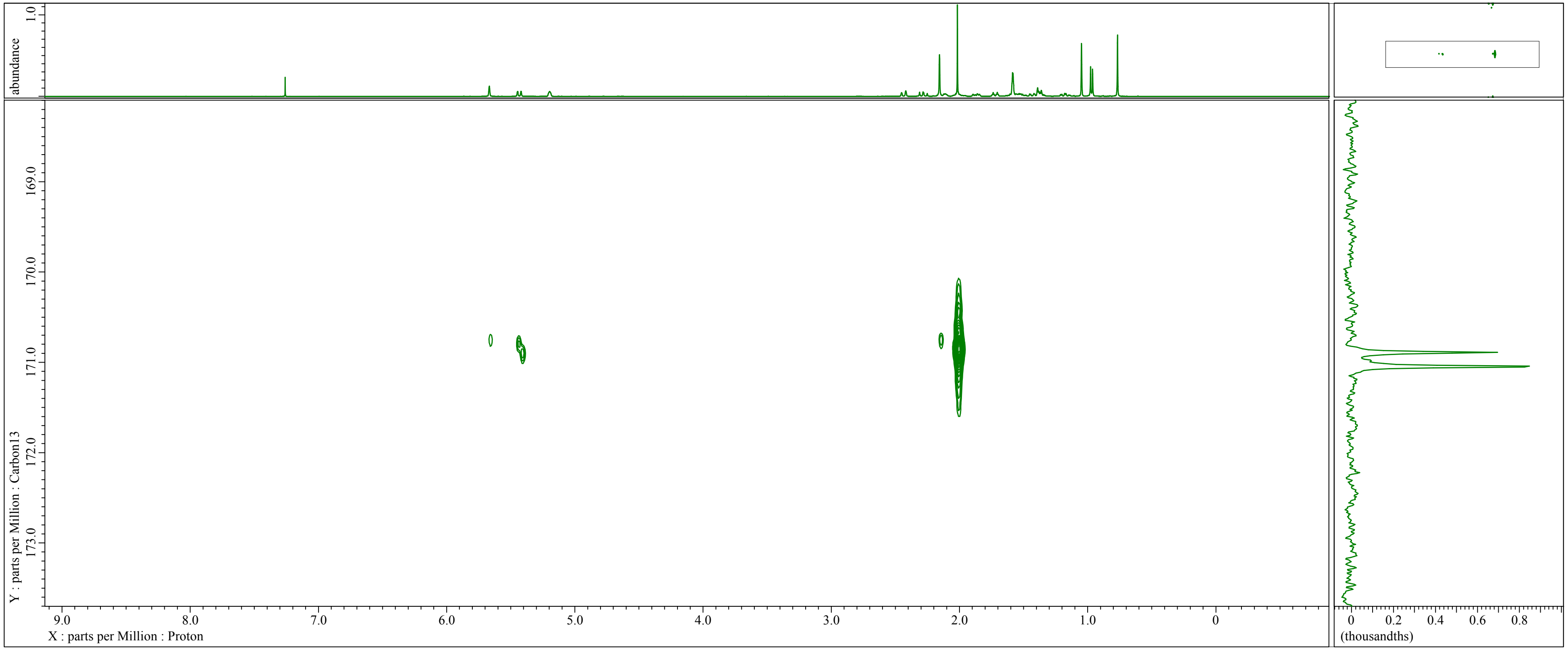

Figure S65. The band-selective HMBC (400/100 MHz, $CDCl_3$) spectrum of compound **7**

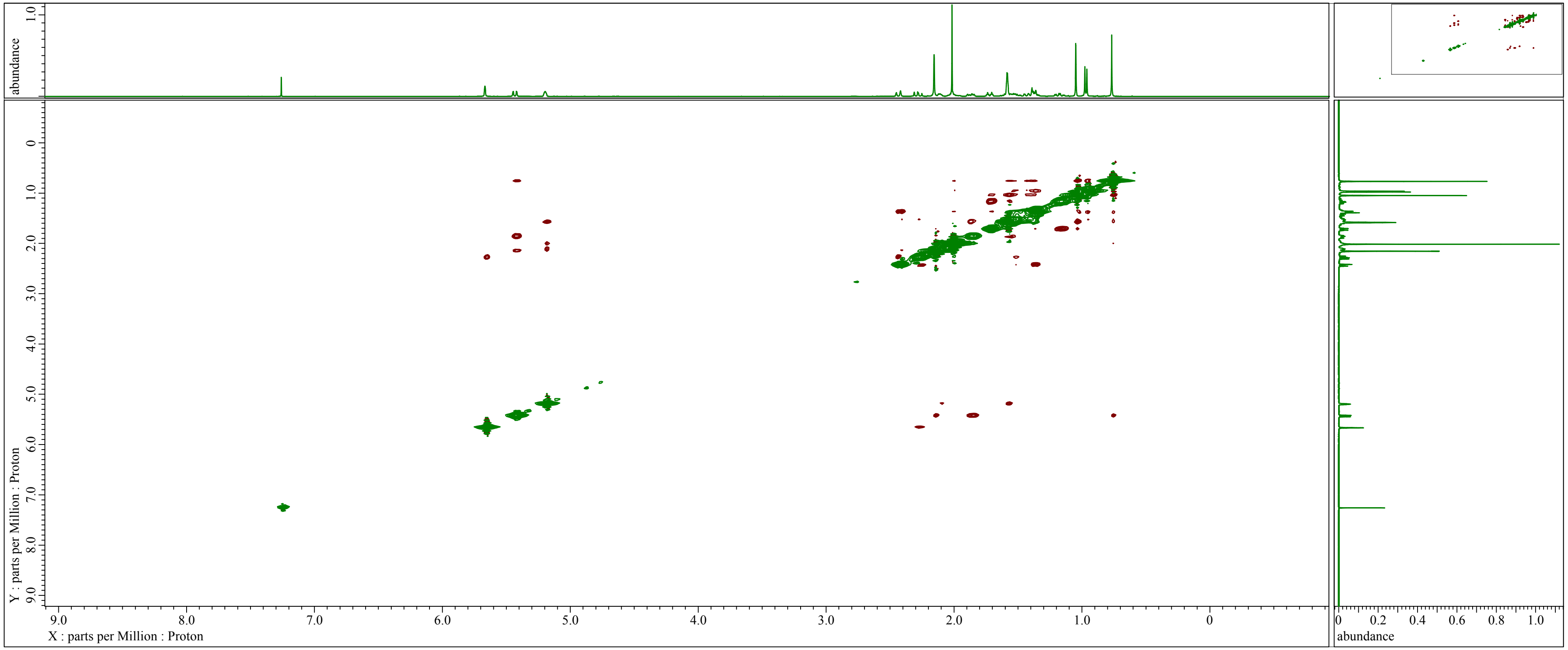

Figure S66. The NOESY (400 MHz, $CDCl_3$) spectrum of compound **7**

12. MS and NMR data for compound 8

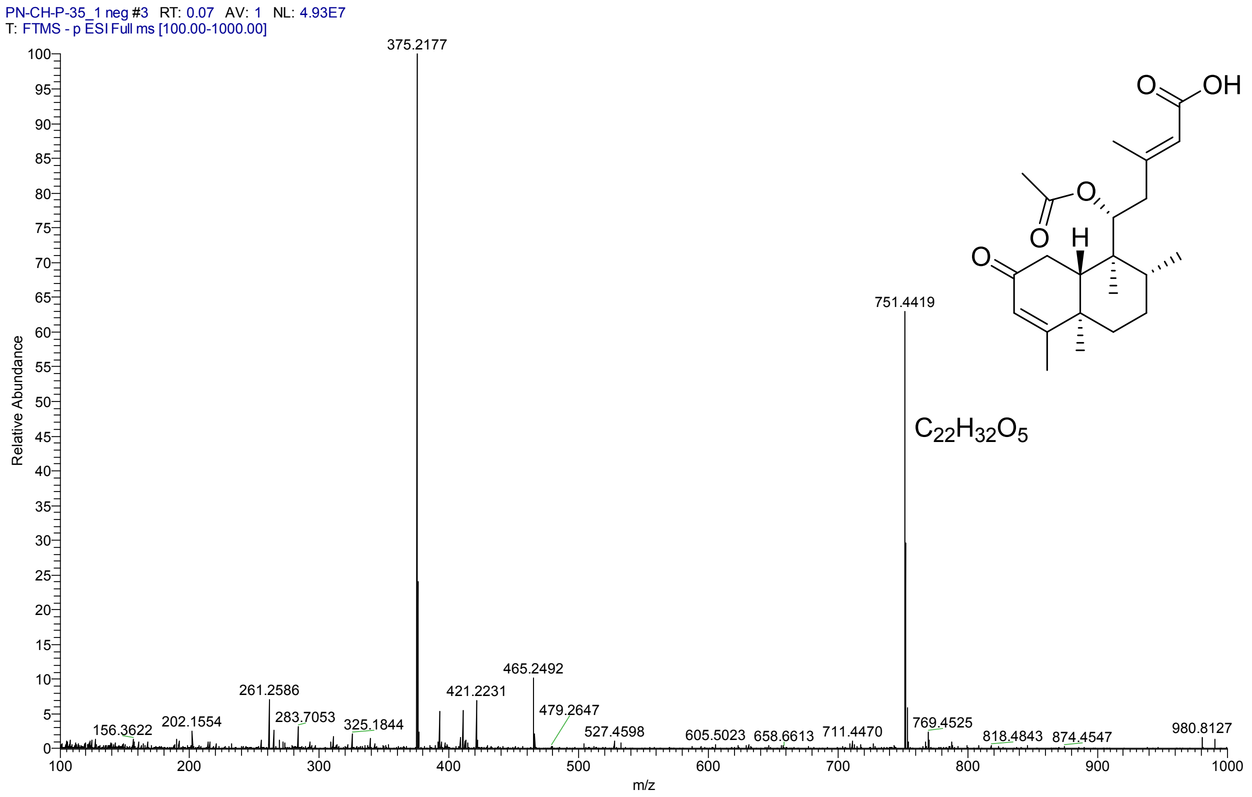

Figure S67. The HRESIMS spectrum of compound **8** (negative mode)

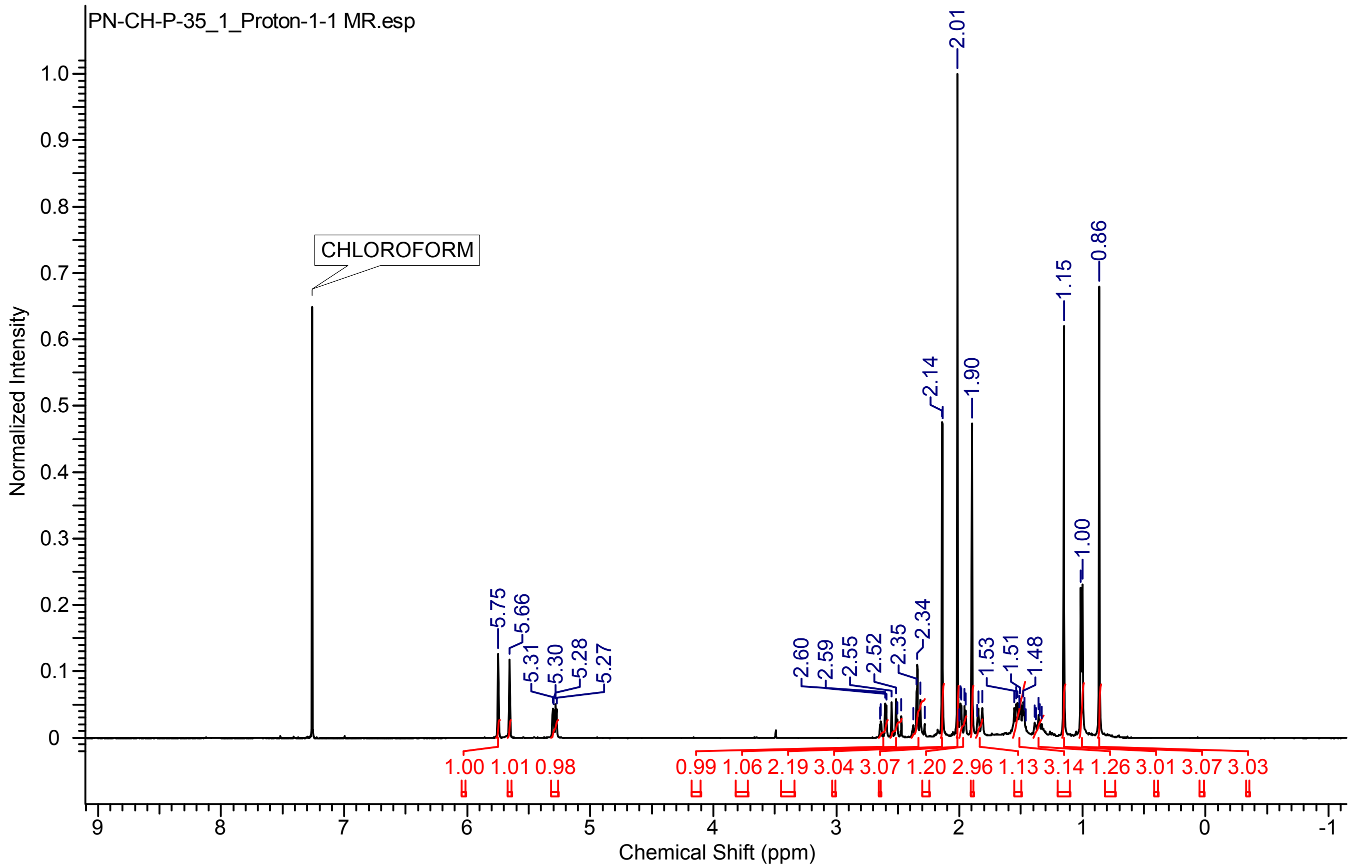

Figure S68. The ^{1}H NMR (400 MHz, $CDCl_3$) spectrum of compound **8**

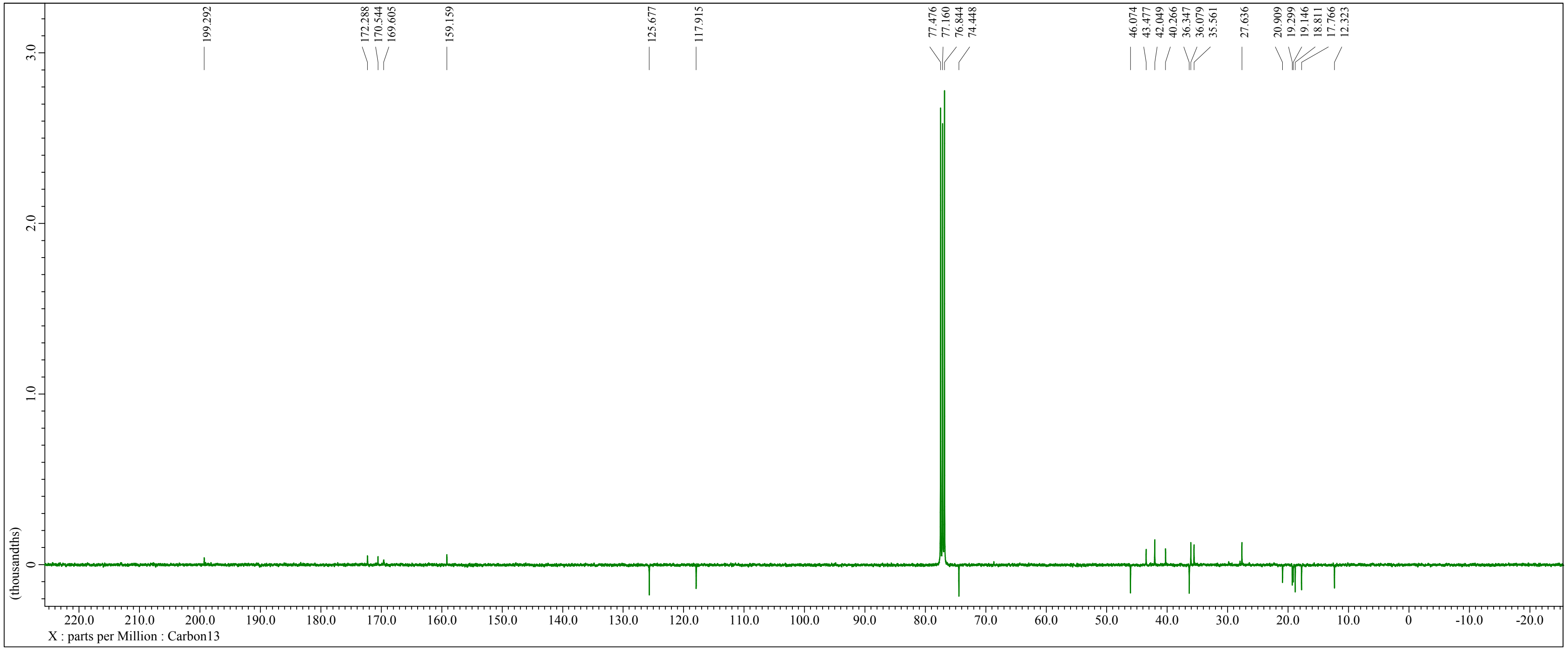

Figure S69. The ^{13}C NMR APT (100 MHz, $CDCl_3$) spectrum of compound **8**

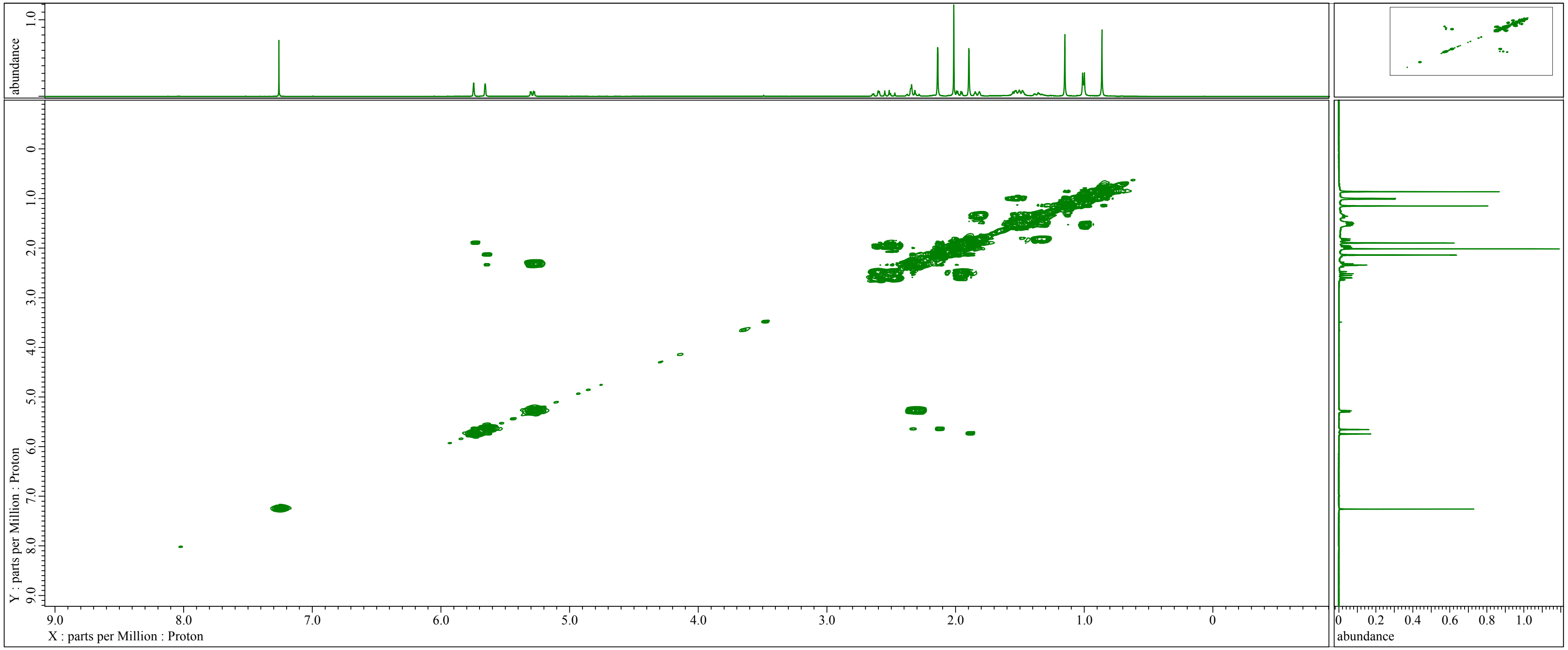

Figure S70. The COSY (400 MHz, $CDCl_3$) spectrum of compound **8**

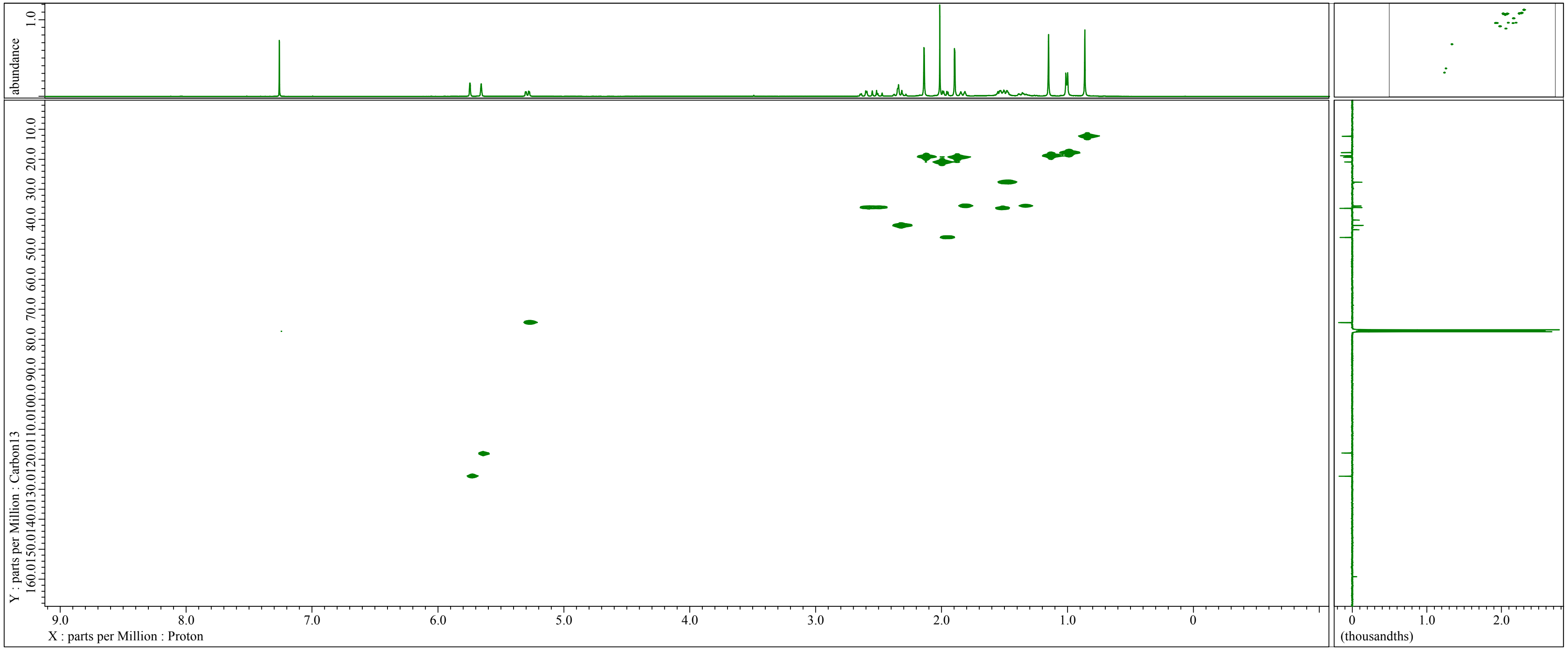

Figure S71. The HSQC (400/100 MHz, $CDCl_3$) spectrum of compound **8**

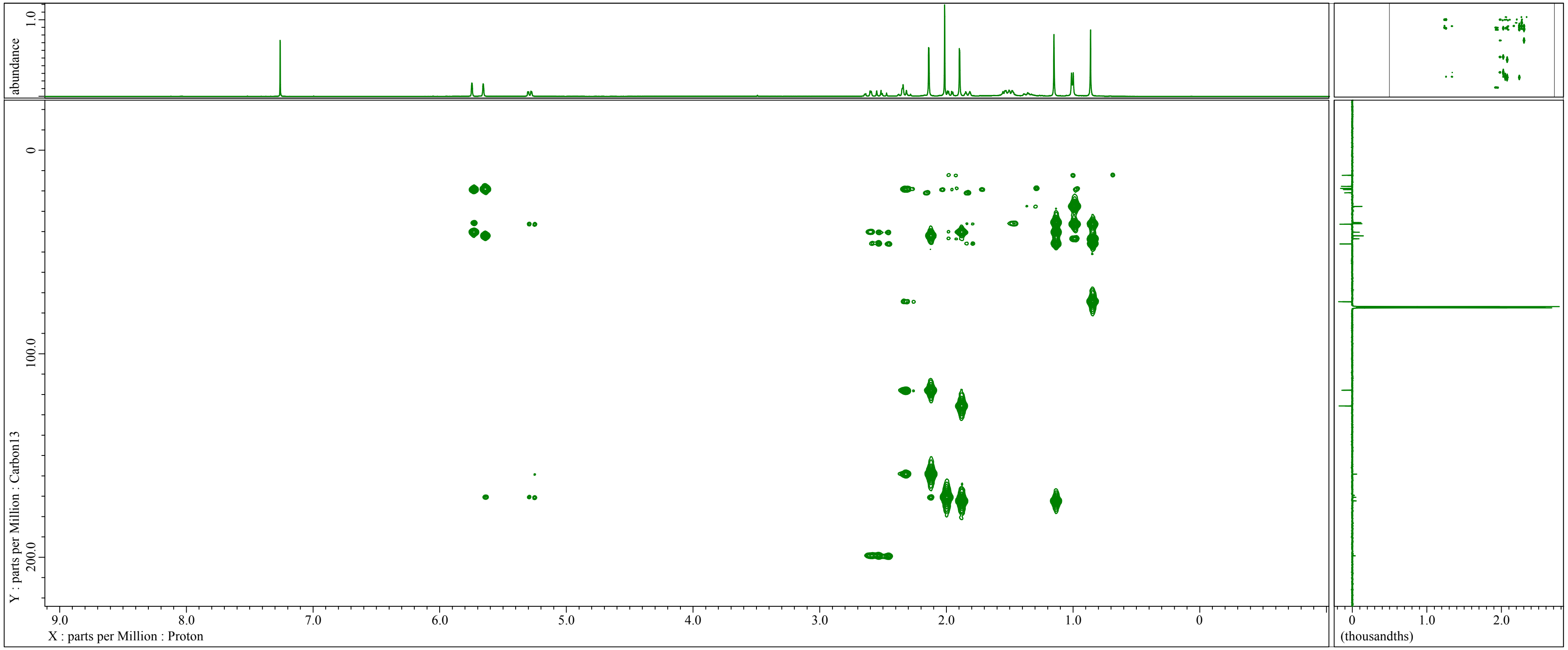

Figure S72. The HMBC (400/100 MHz, $CDCl_3$) spectrum of compound **8**

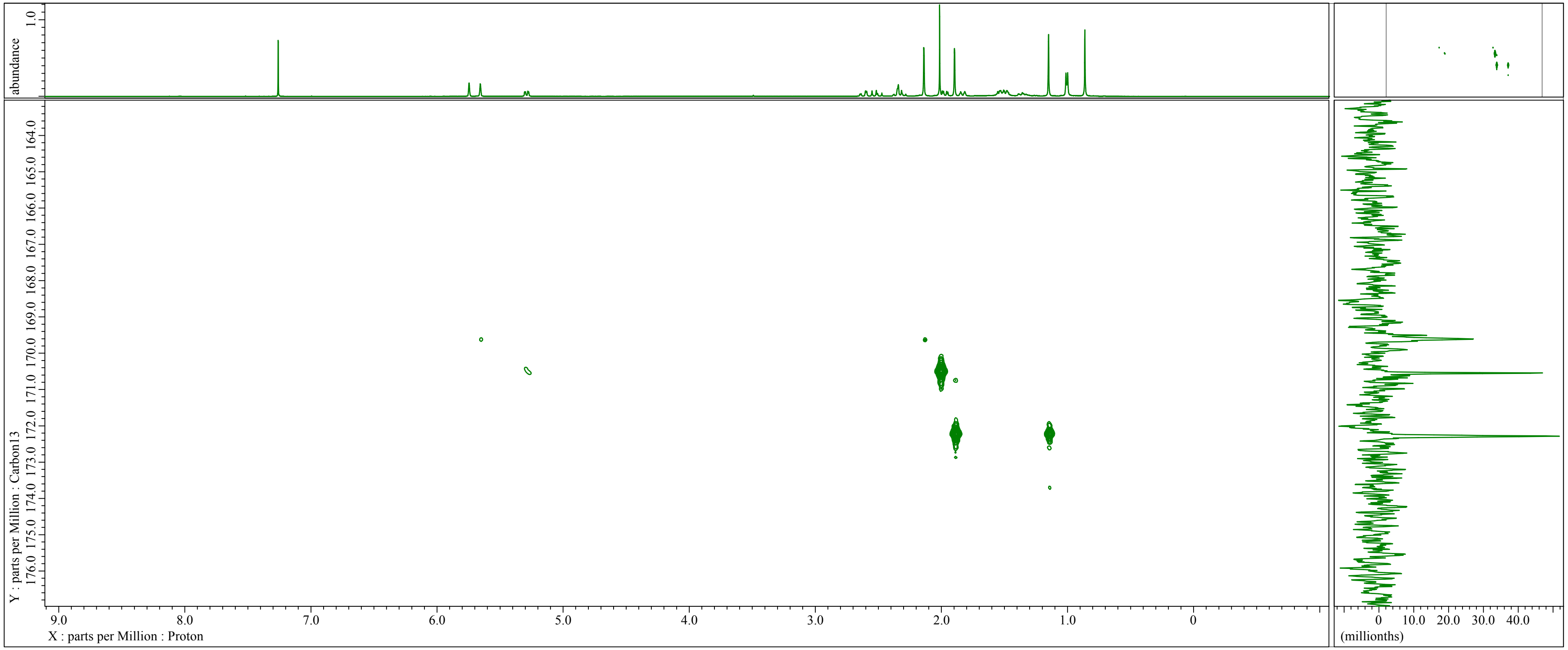

Figure S73. The band-selective HMBC (400/100 MHz, $CDCl_3$) spectrum of compound **8**

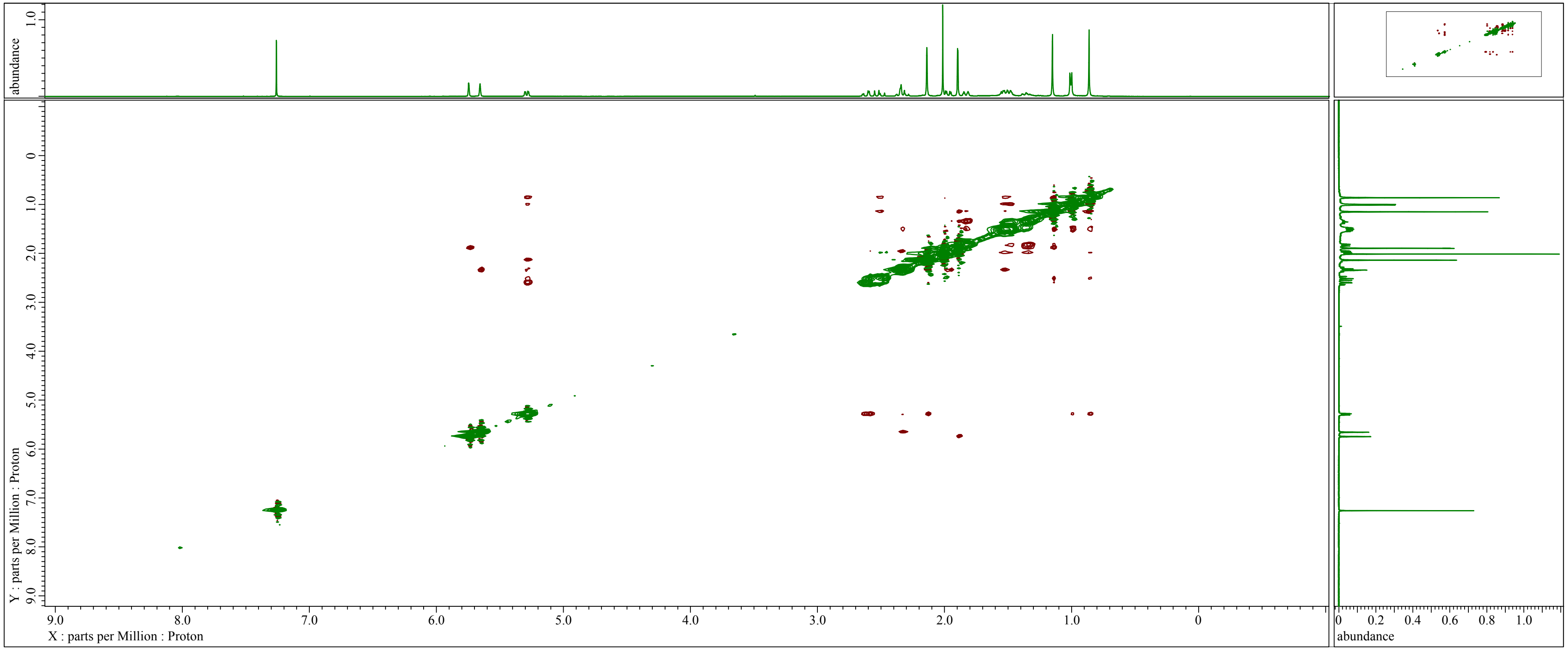

Figure S74. The NOESY (400 MHz, $CDCl_3$) spectrum of compound **8**

13. MS and NMR data for compound 9

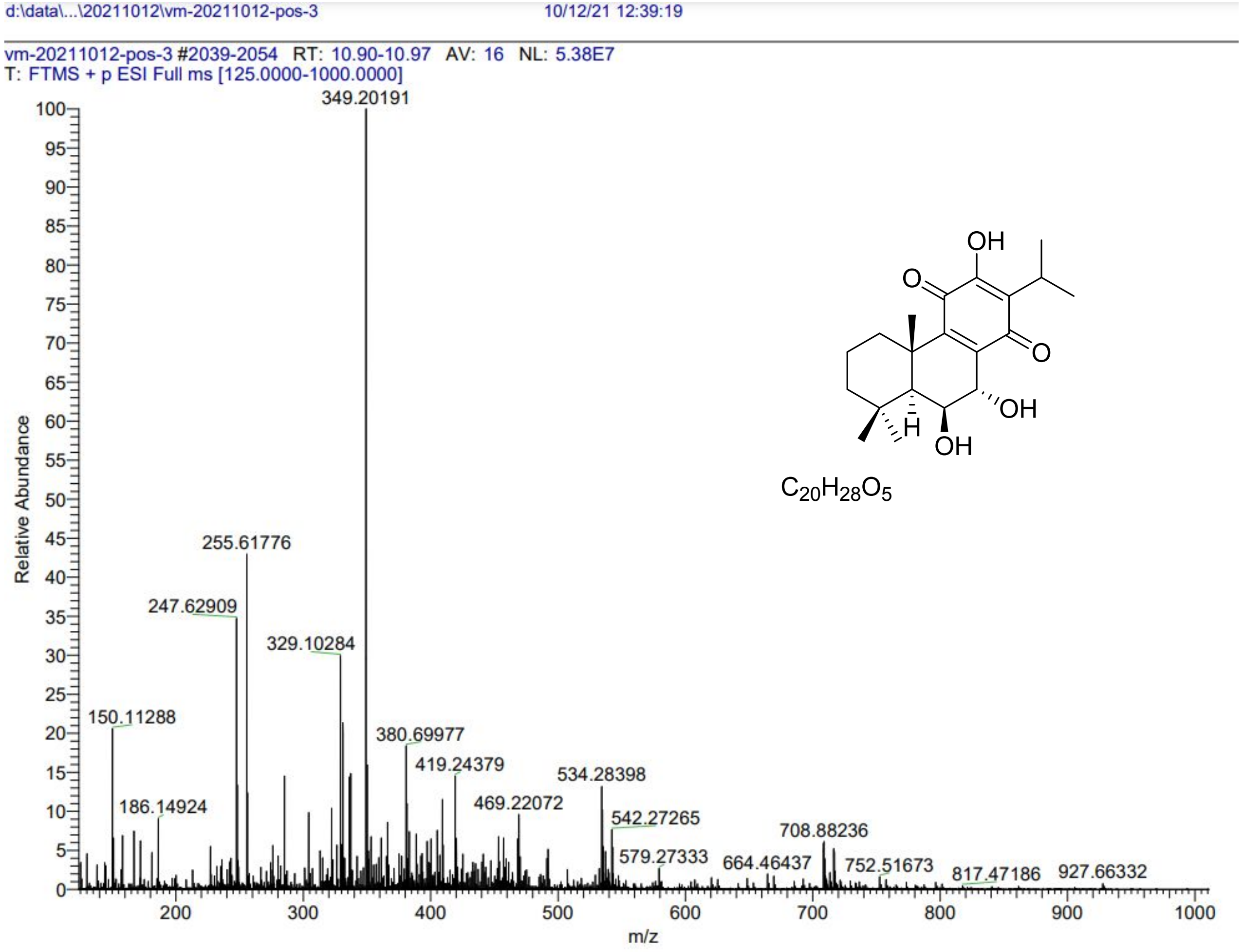

Figure S75. The HRESIMS spectrum of compound **9** (positive mode)

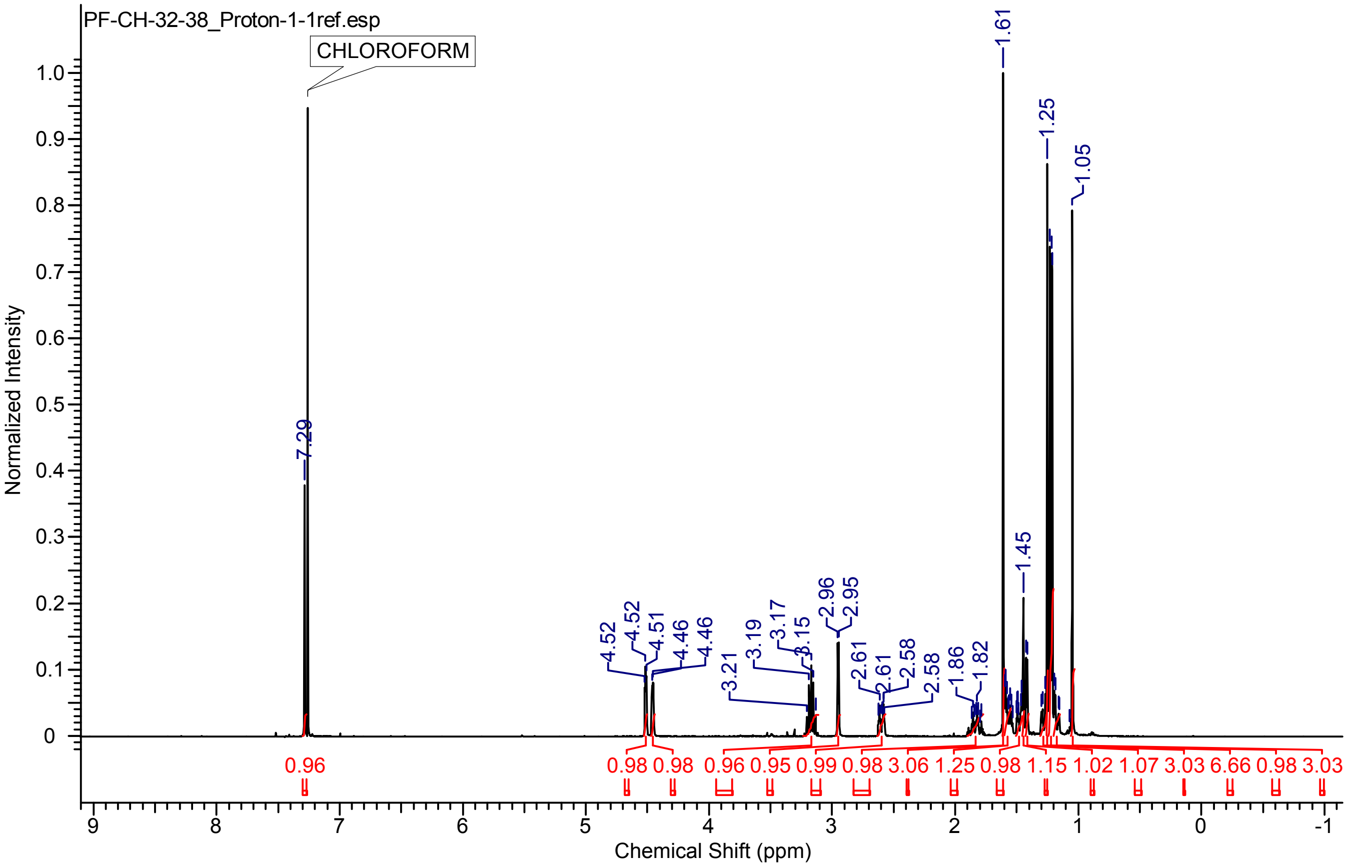

Figure S76. The ^{1}H NMR (400 MHz, $CDCl_3$) spectrum of compound **9**

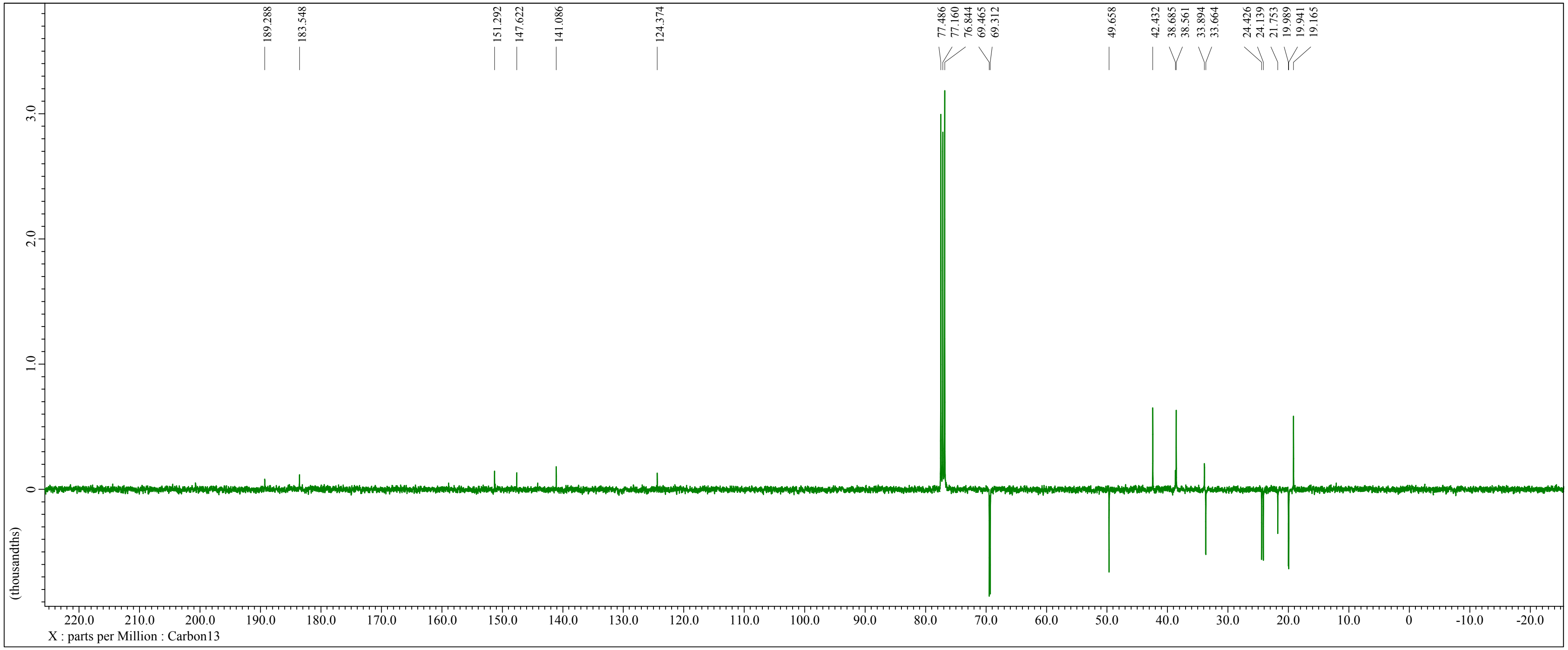

Figure S77. The ^{13}C NMR APT (100 MHz, $CDCl_3$) spectrum of compound **9**

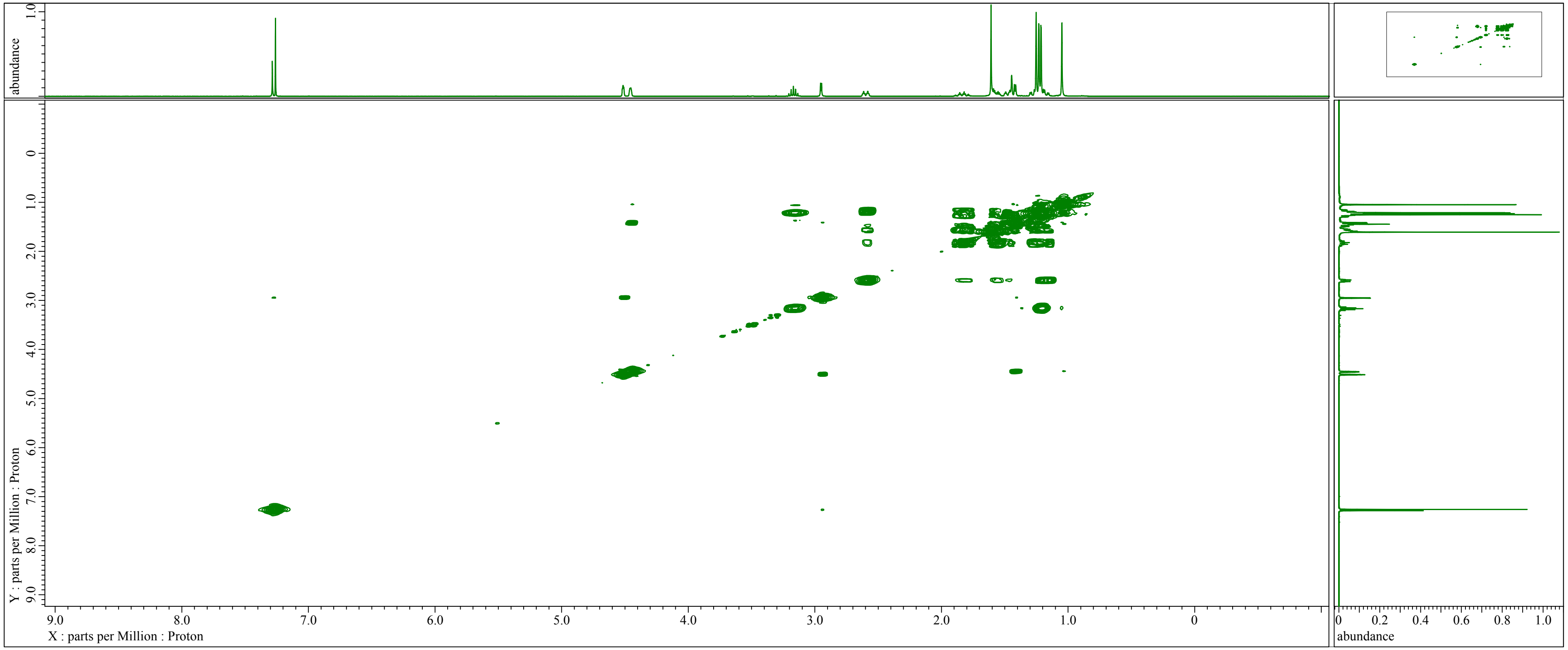

Figure S78. The COSY (400 MHz, $CDCl_3$) spectrum of compound **9**

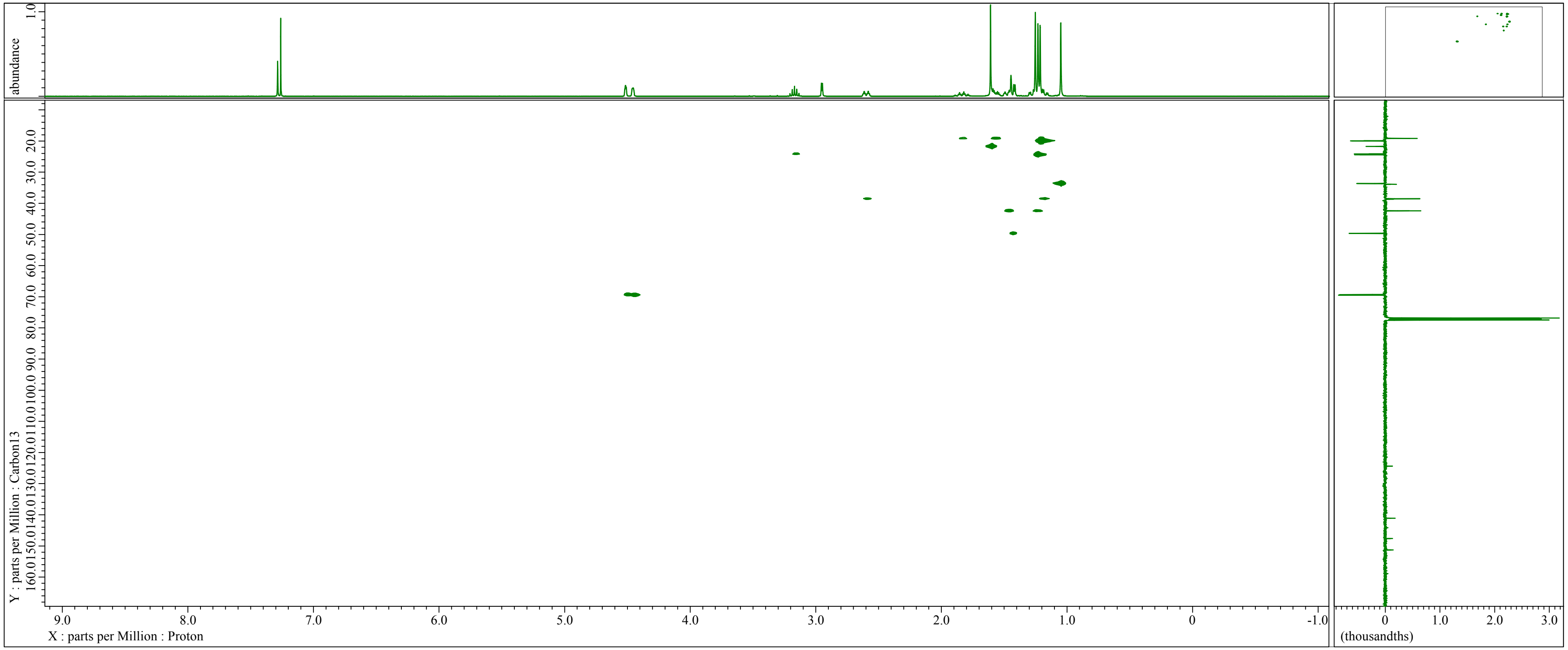

Figure S79. The HSQC (400/100 MHz, $CDCl_3$) spectrum of compound **9**

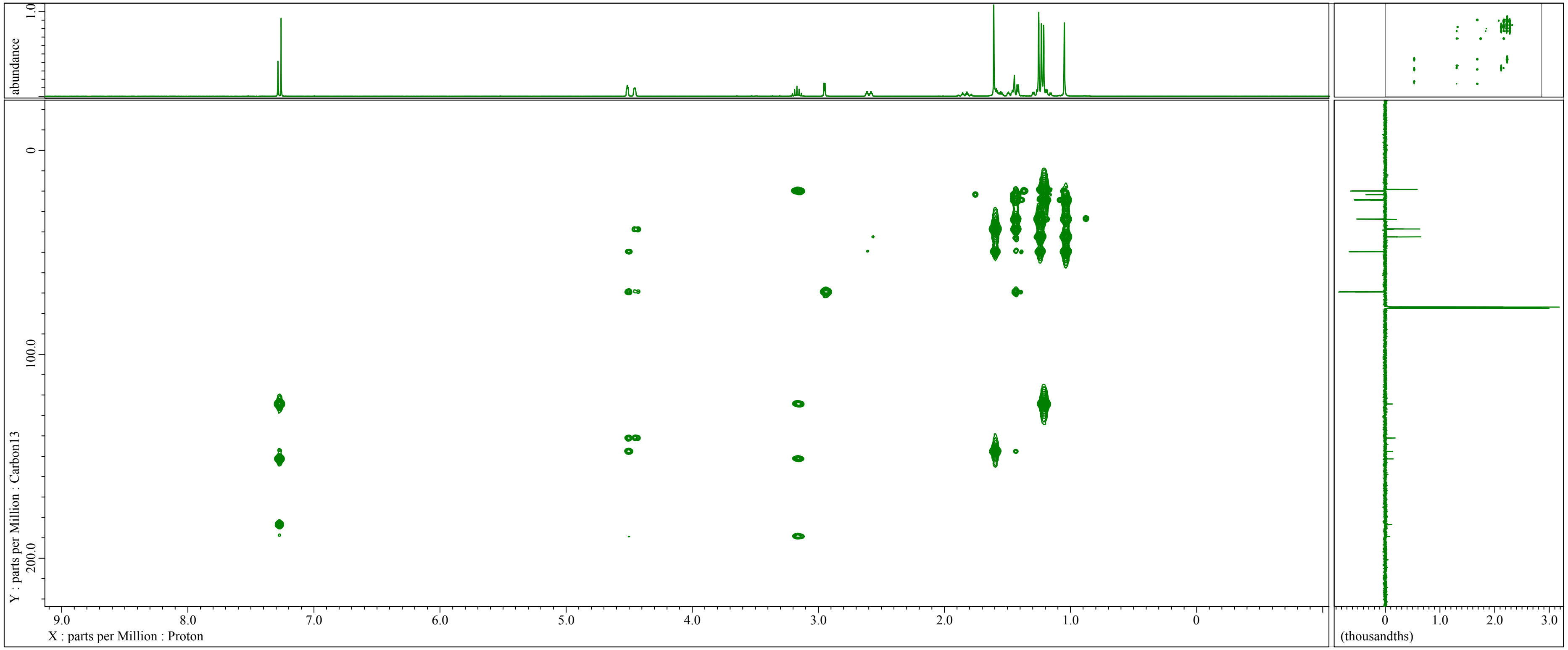

Figure S80. The HMBC (400/100 MHz, $CDCl_3$) spectrum of compound **9**

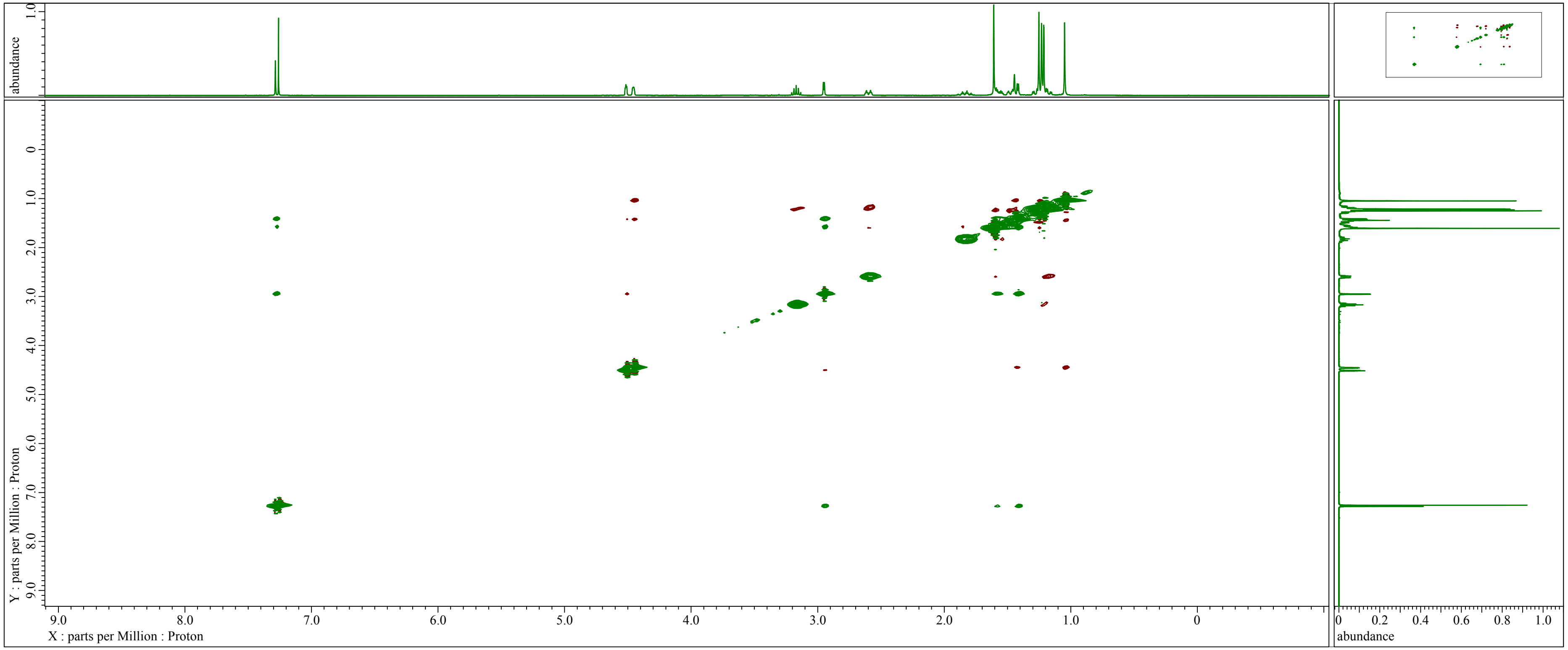

Figure S81. The NOESY (400 MHz, $CDCl_3$) spectrum of compound **9**

14. MS and NMR data for compound 10

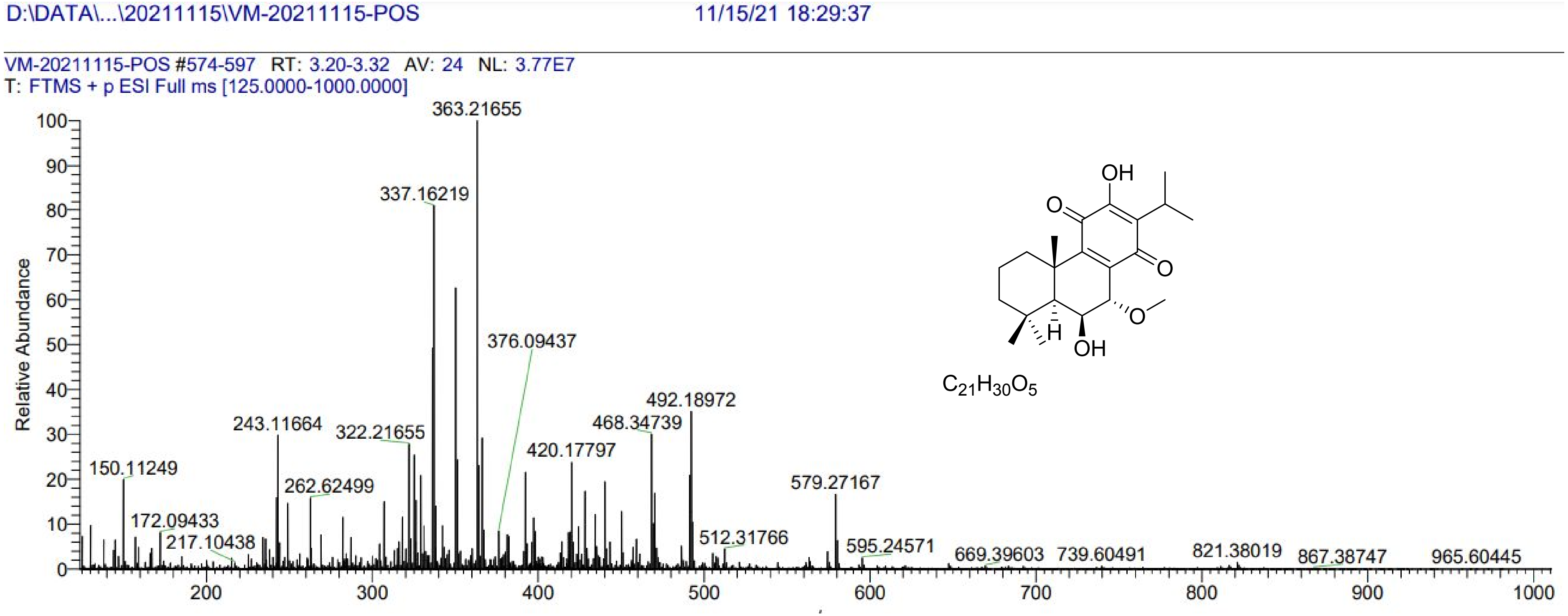

Figure S82. The HRESIMS spectrum of compound **10** (positive mode)

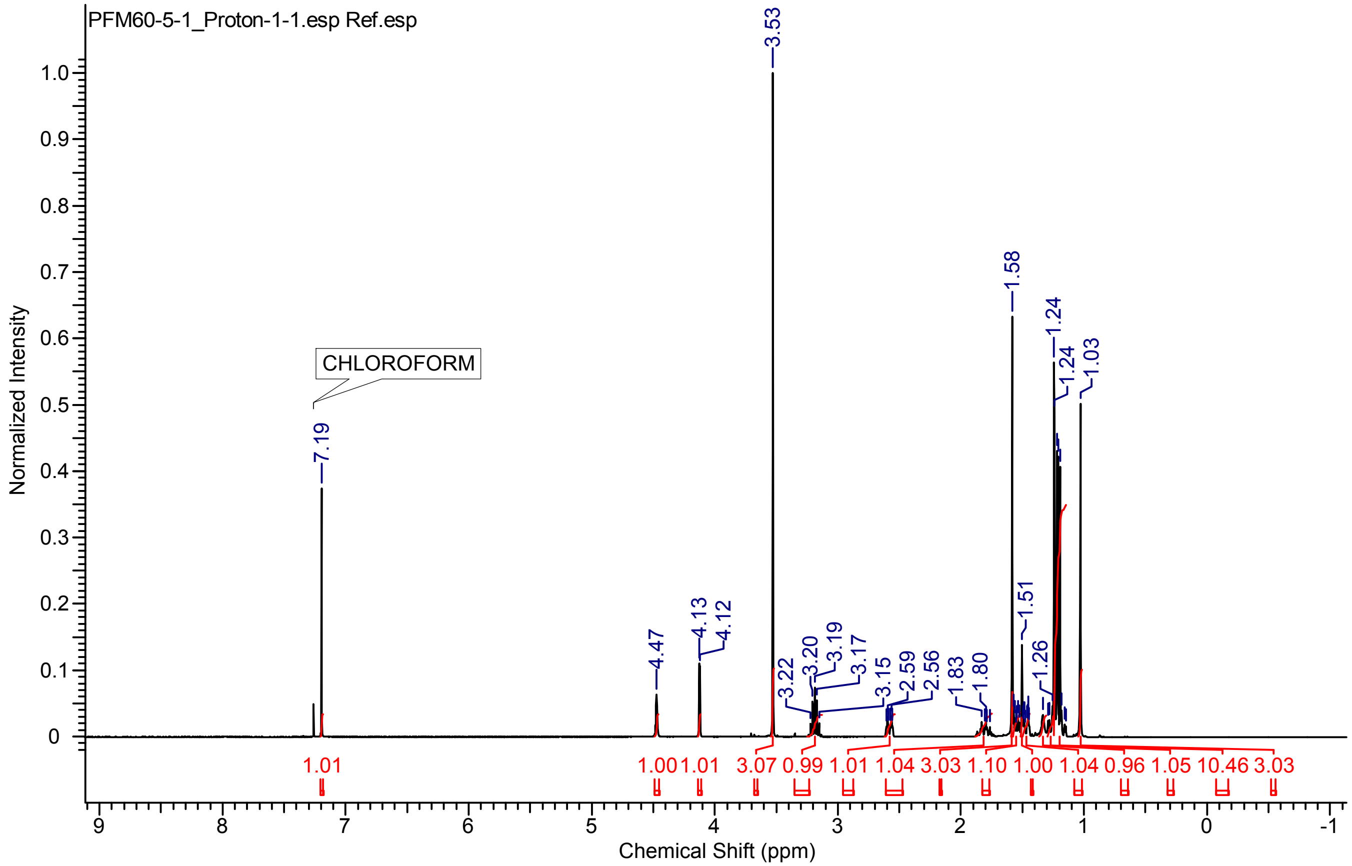

Figure S83. The ^{1}H NMR (400 MHz, $CDCl_3$) spectrum of compound **10**

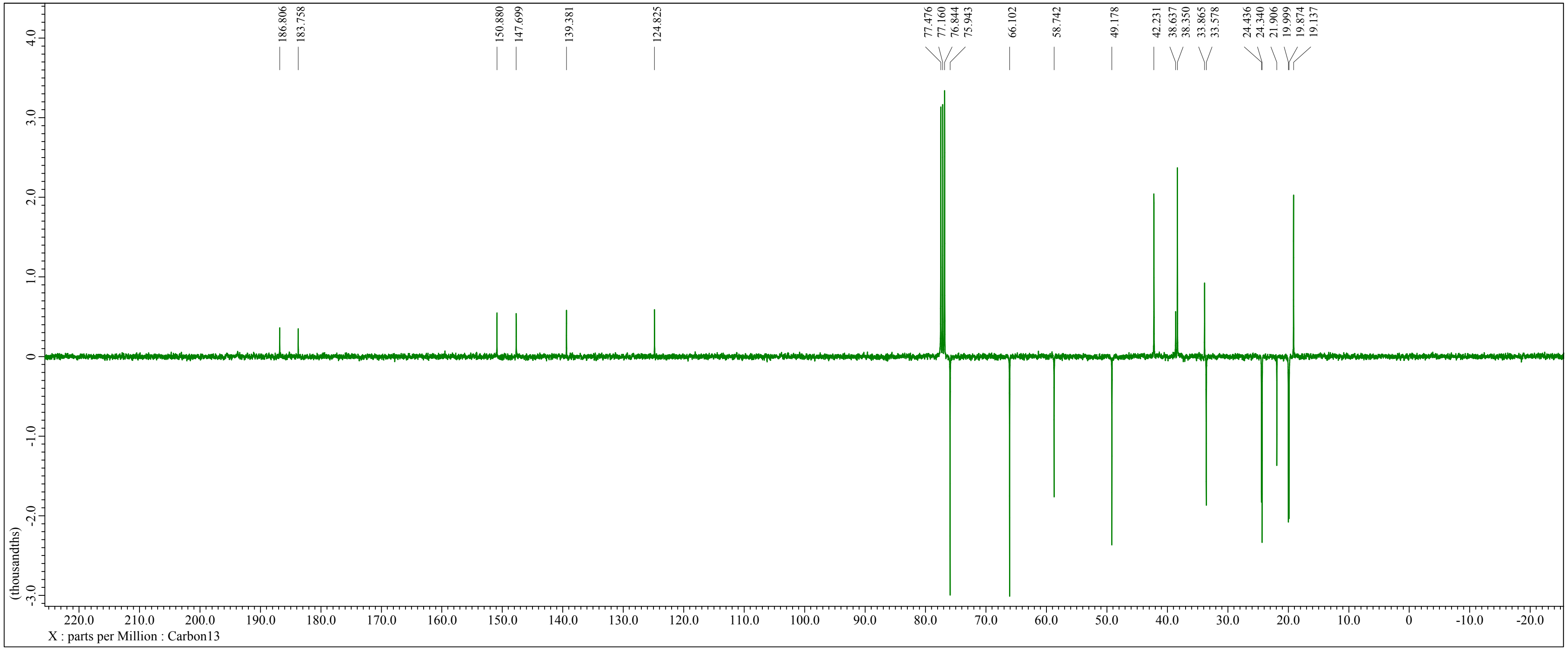

Figure S84. The ^{13}C NMR APT (100 MHz, $CDCl_3$) spectrum of compound **10**

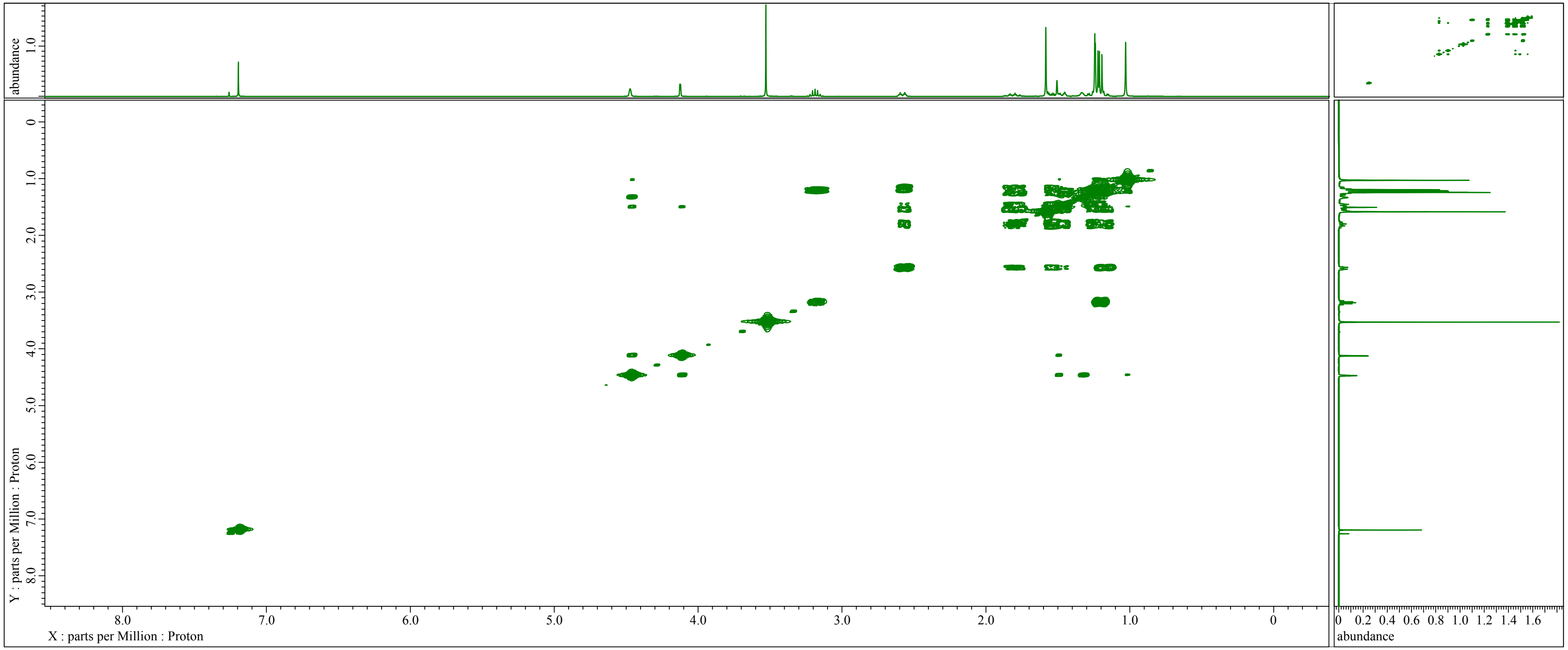

Figure S85. The COSY (400 MHz, $CDCl_3$) spectrum of compound **10**

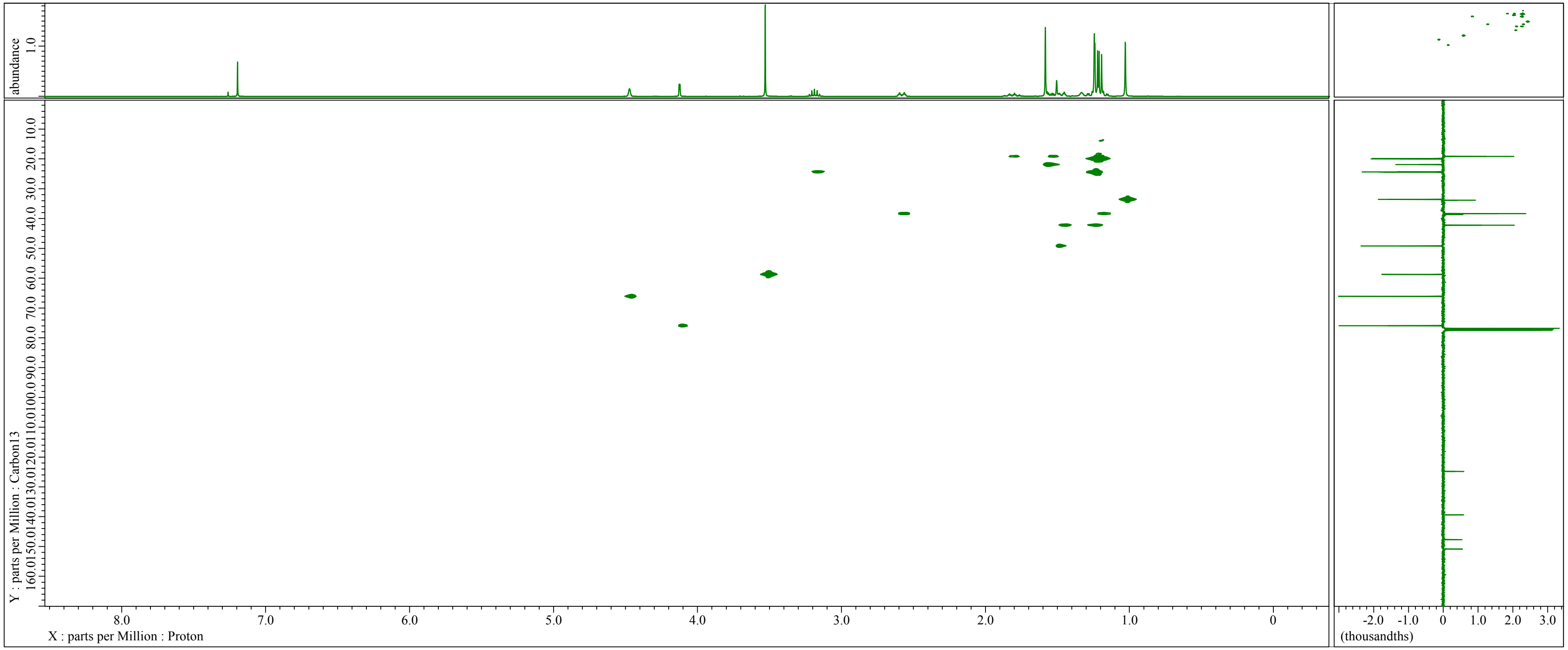

Figure S86. The HSQC (400/100 MHz, $CDCl_3$) spectrum of compound **10**

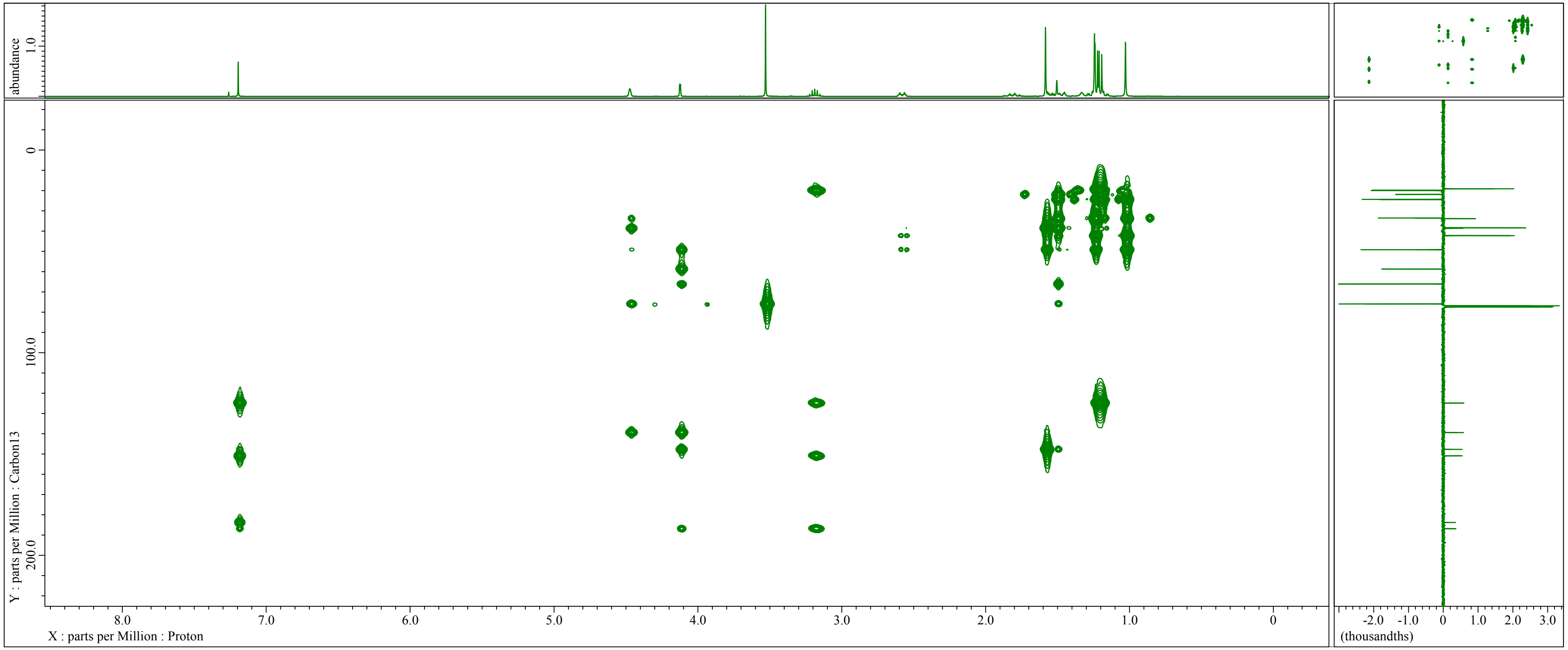

Figure S87. The HMBC (400/100 MHz, $CDCl_3$) spectrum of compound **10**

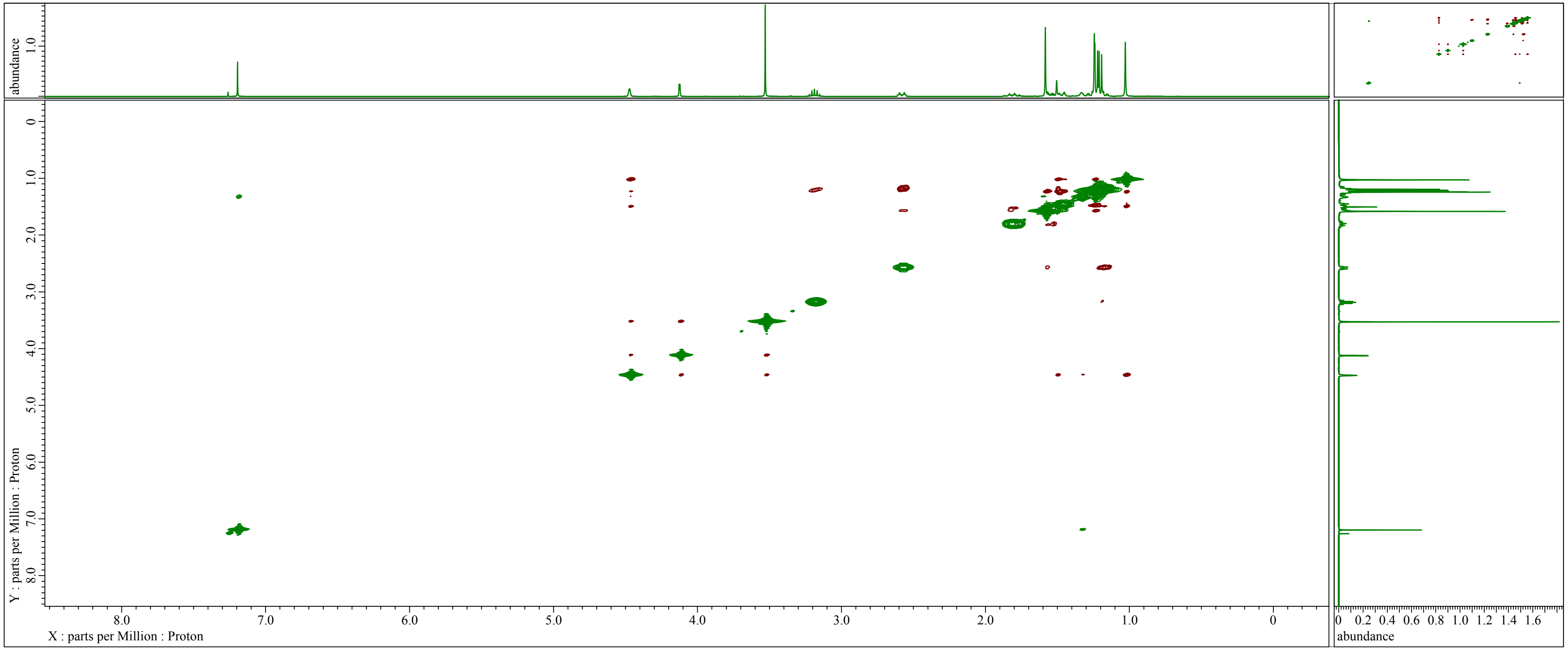

Figure S88. The NOESY (400 MHz, $CDCl_3$) spectrum of compound **10**

15. MS and NMR data for compound 11

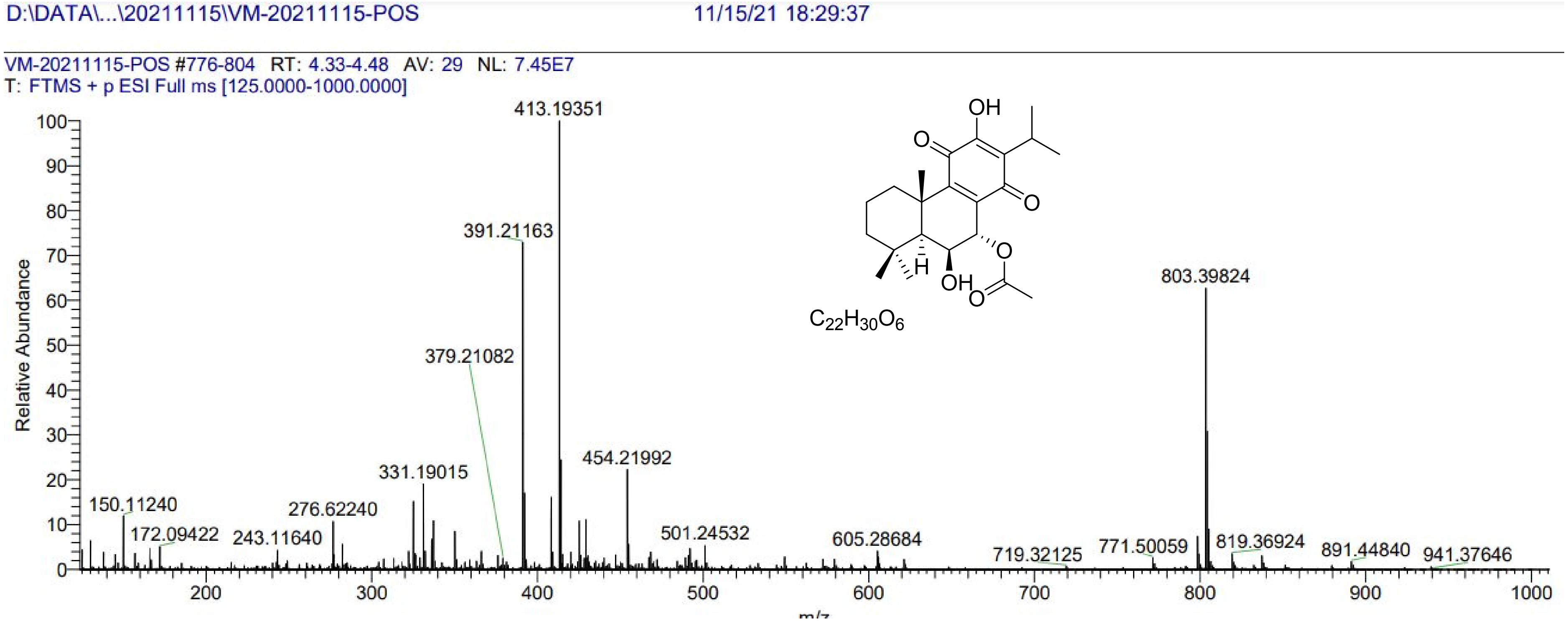

Figure S89. The HRESIMS spectrum of compound **11** (positive mode)

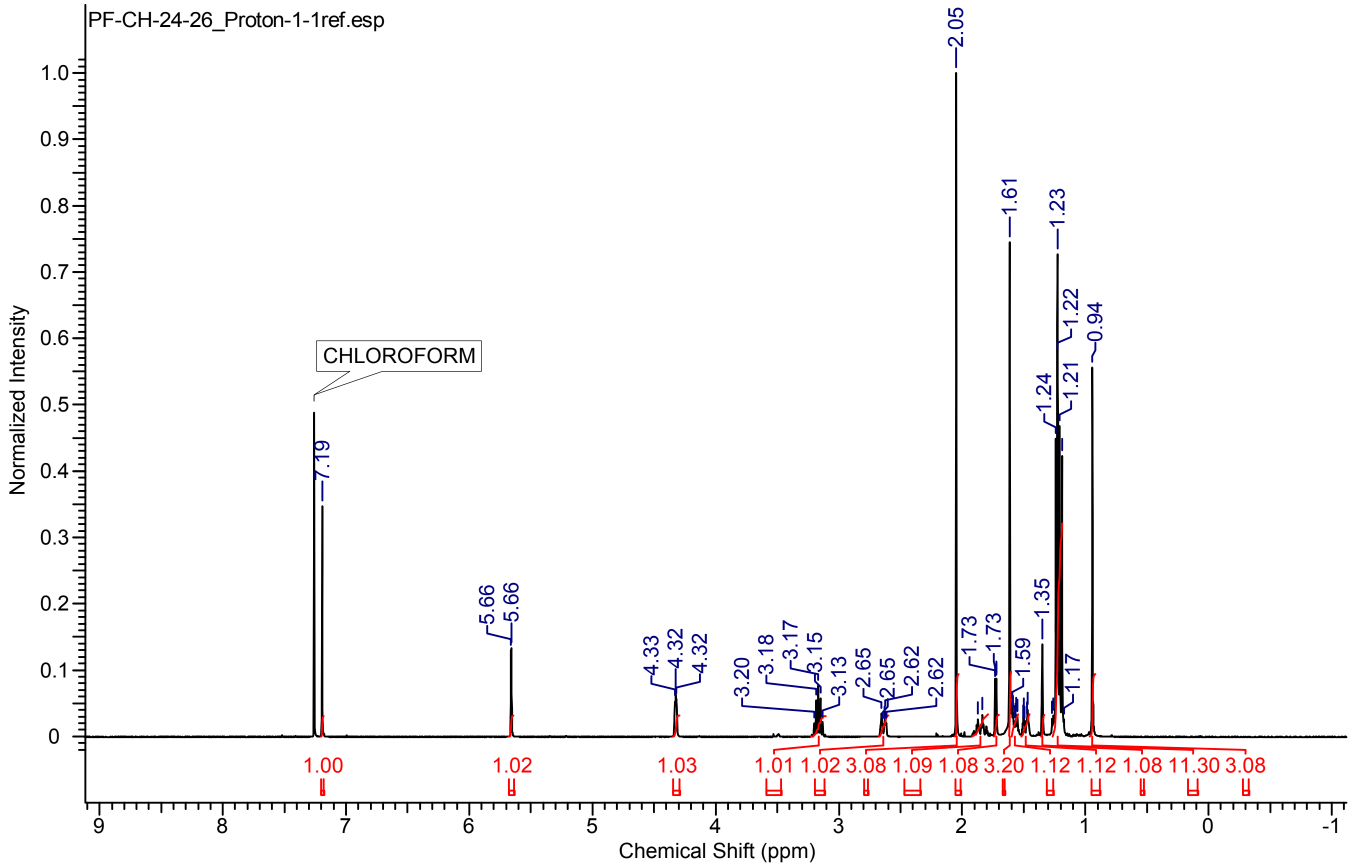

Figure S90. The ^{1}H NMR (400 MHz, $CDCl_3$) spectrum of compound **11**

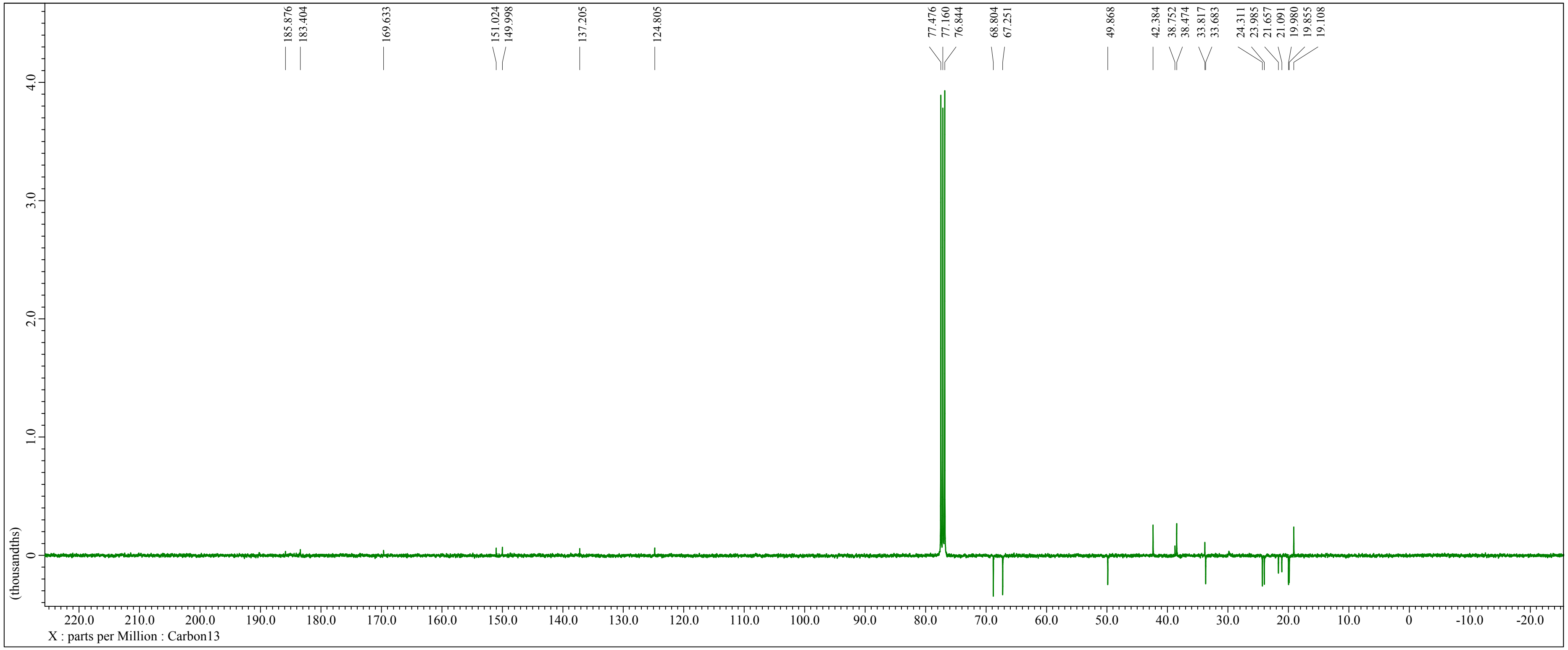

Figure S91. The ^{13}C NMR APT (100 MHz, $CDCl_3$) spectrum of compound **11**

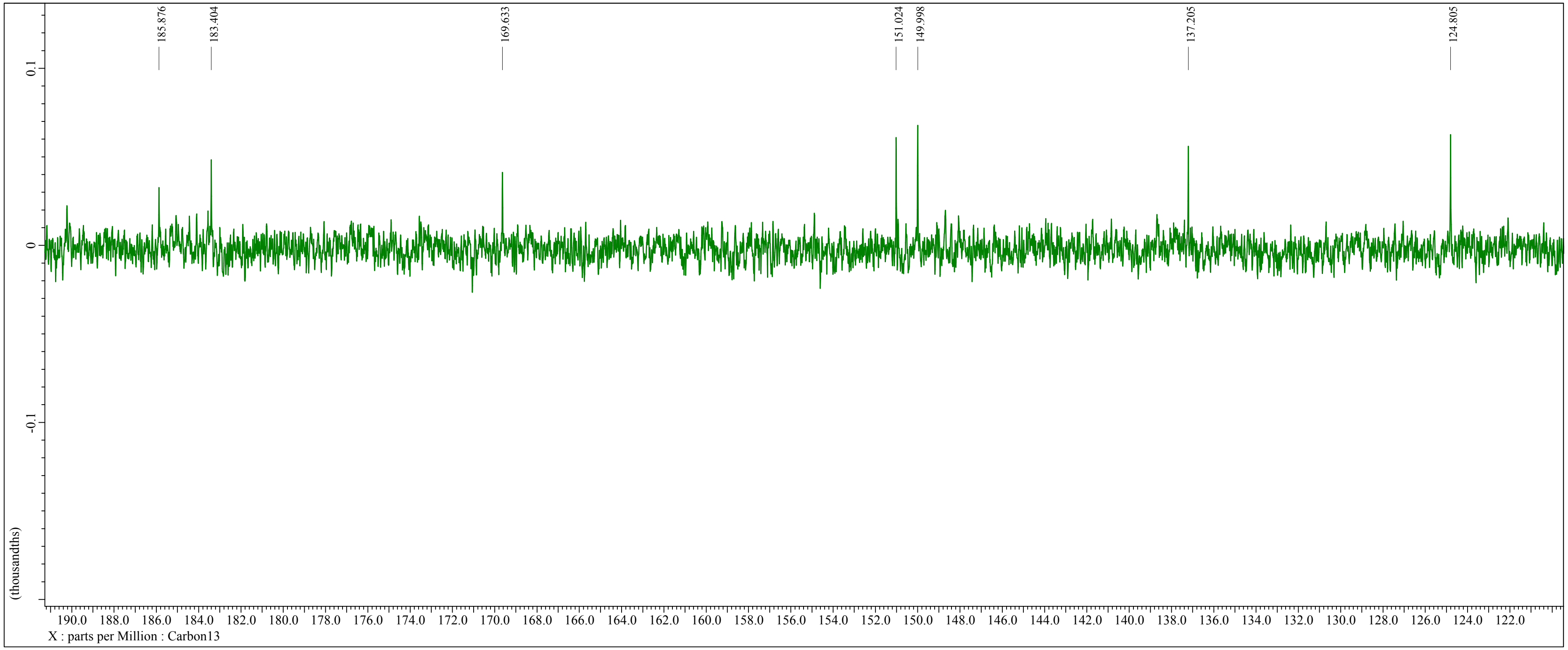

Figure S92. The magnified, zoomed in 120–200 ppm section of the ^{13}C NMR APT (100 MHz, $CDCl_3$) spectrum of compound **11**

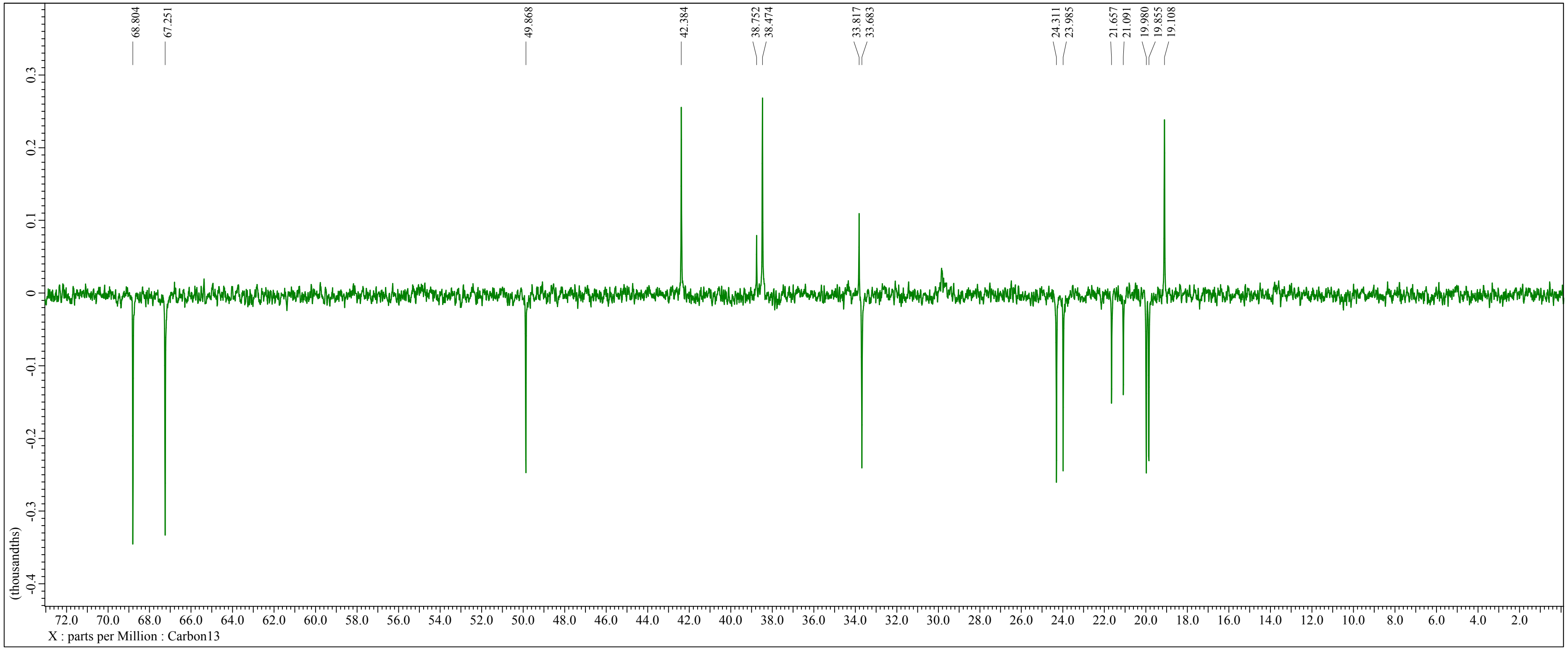

Figure S93. The magnified, zoomed in 0–72 ppm section ^{13}C NMR APT (100 MHz, $CDCl_3$) spectrum of compound **11**

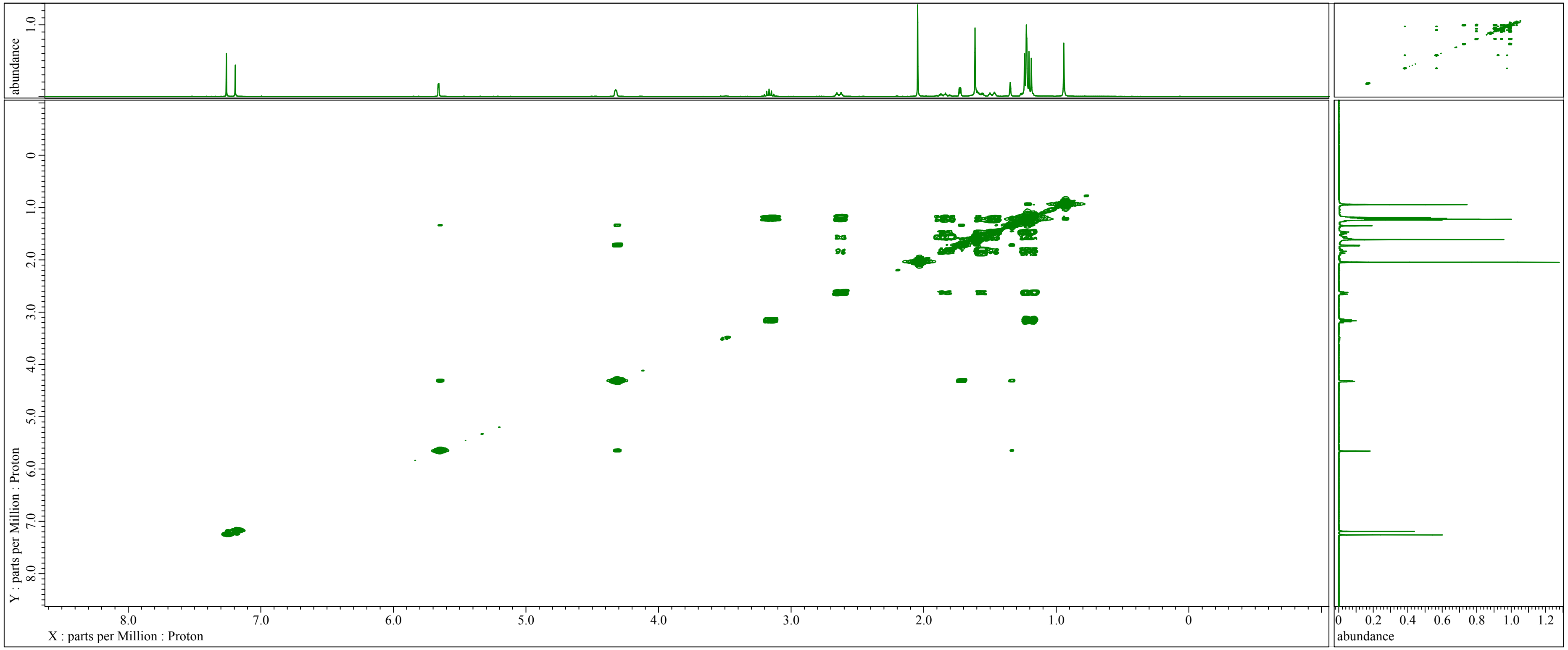

Figure S94. The COSY (400 MHz, $CDCl_3$) spectrum of compound **11**

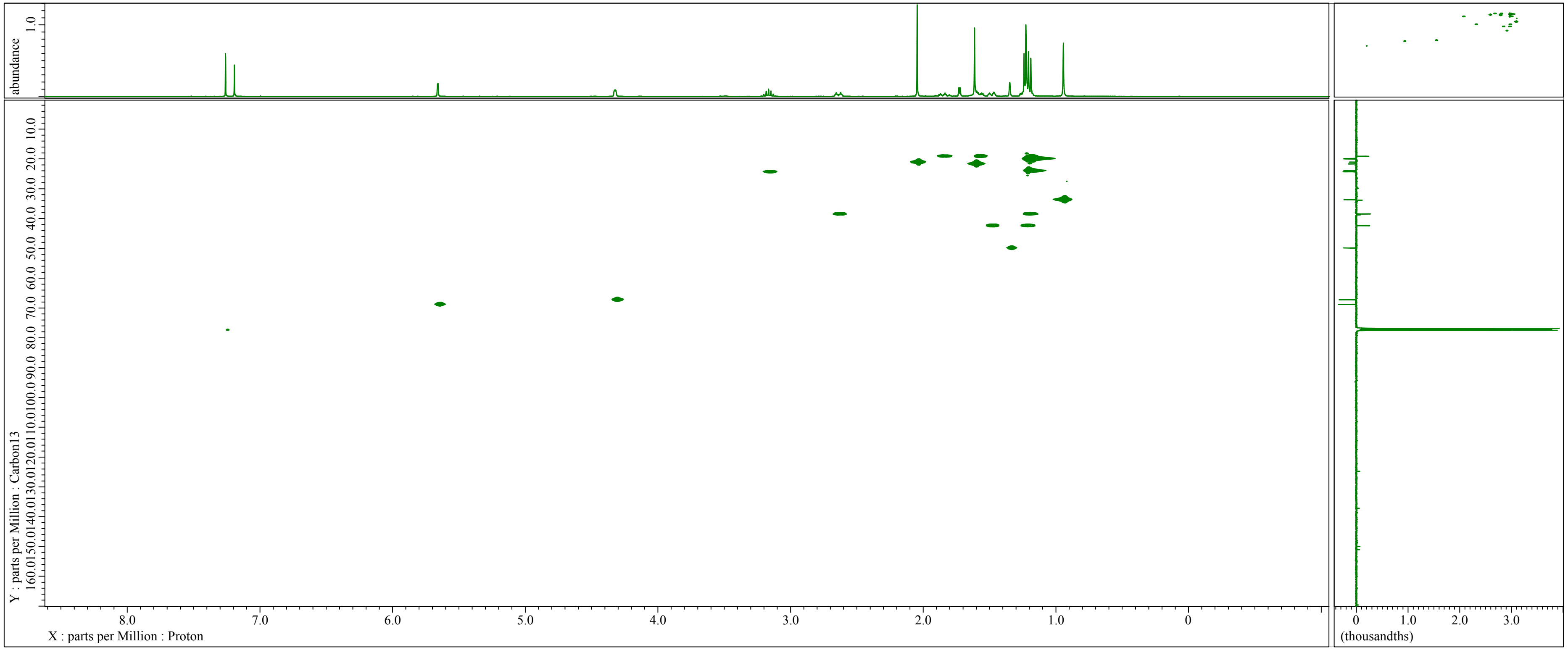

Figure S95. The HSQC (400/100 MHz, $CDCl_3$) spectrum of compound **11**

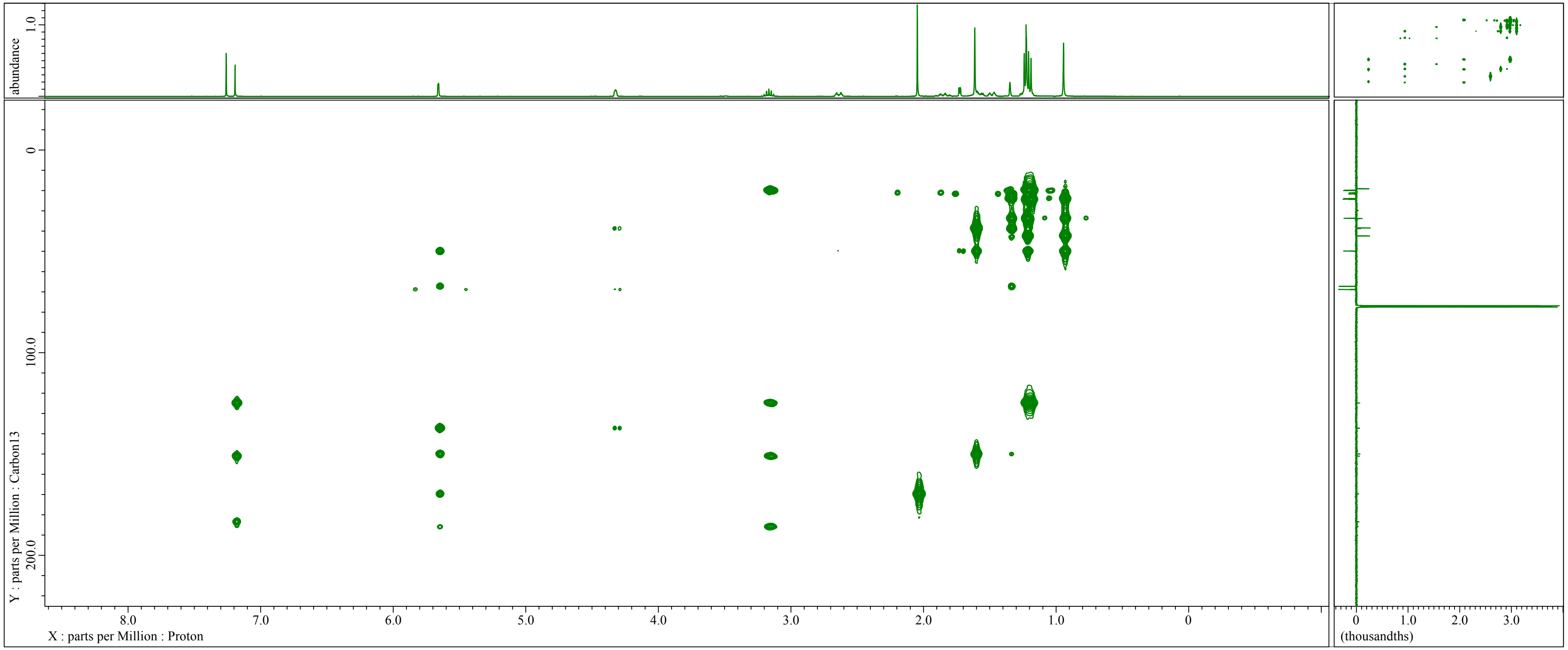

Figure S96. The HMBC (400/100 MHz, $CDCl_3$) spectrum of compound **11**

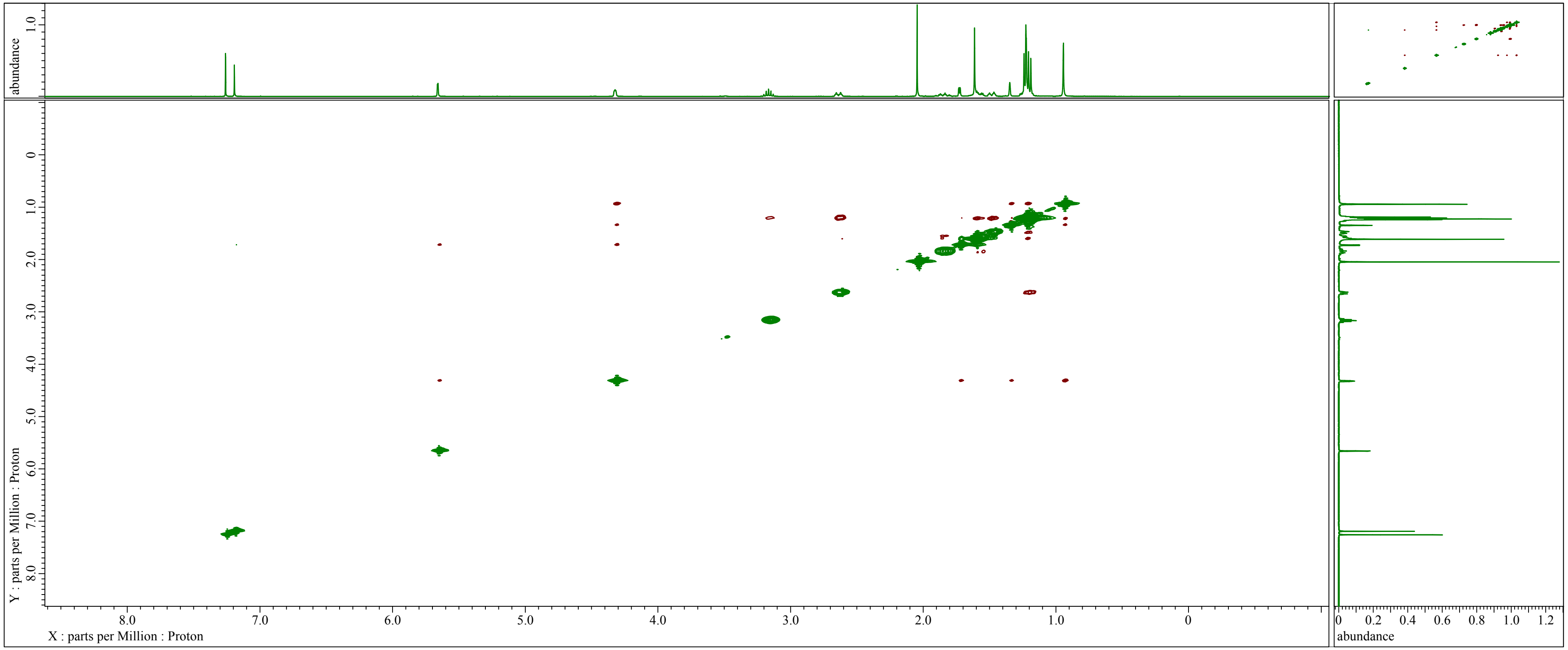

Figure S97. The NOESY (400 MHz, $CDCl_3$) spectrum of compound **11**

16. MS and NMR data for compound 13

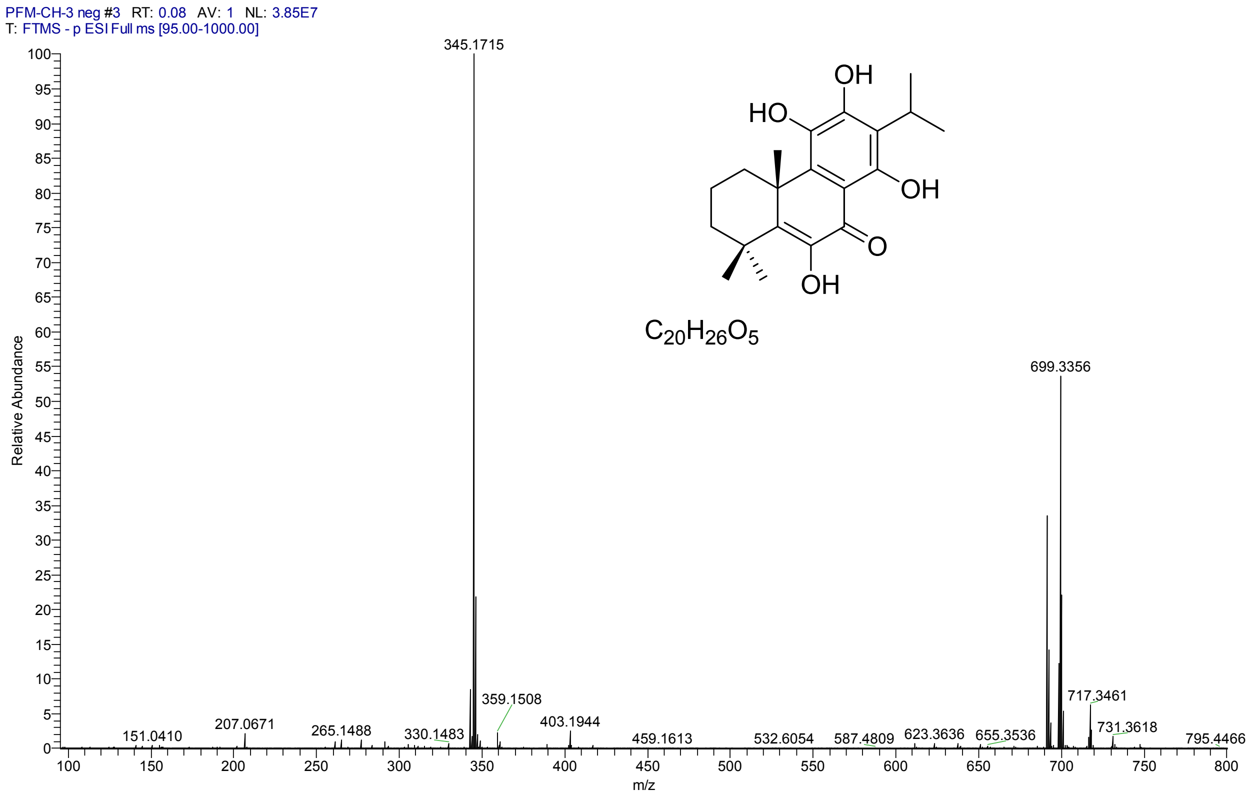

Figure S98. The HRESIMS spectrum of compound **13** (negative mode)

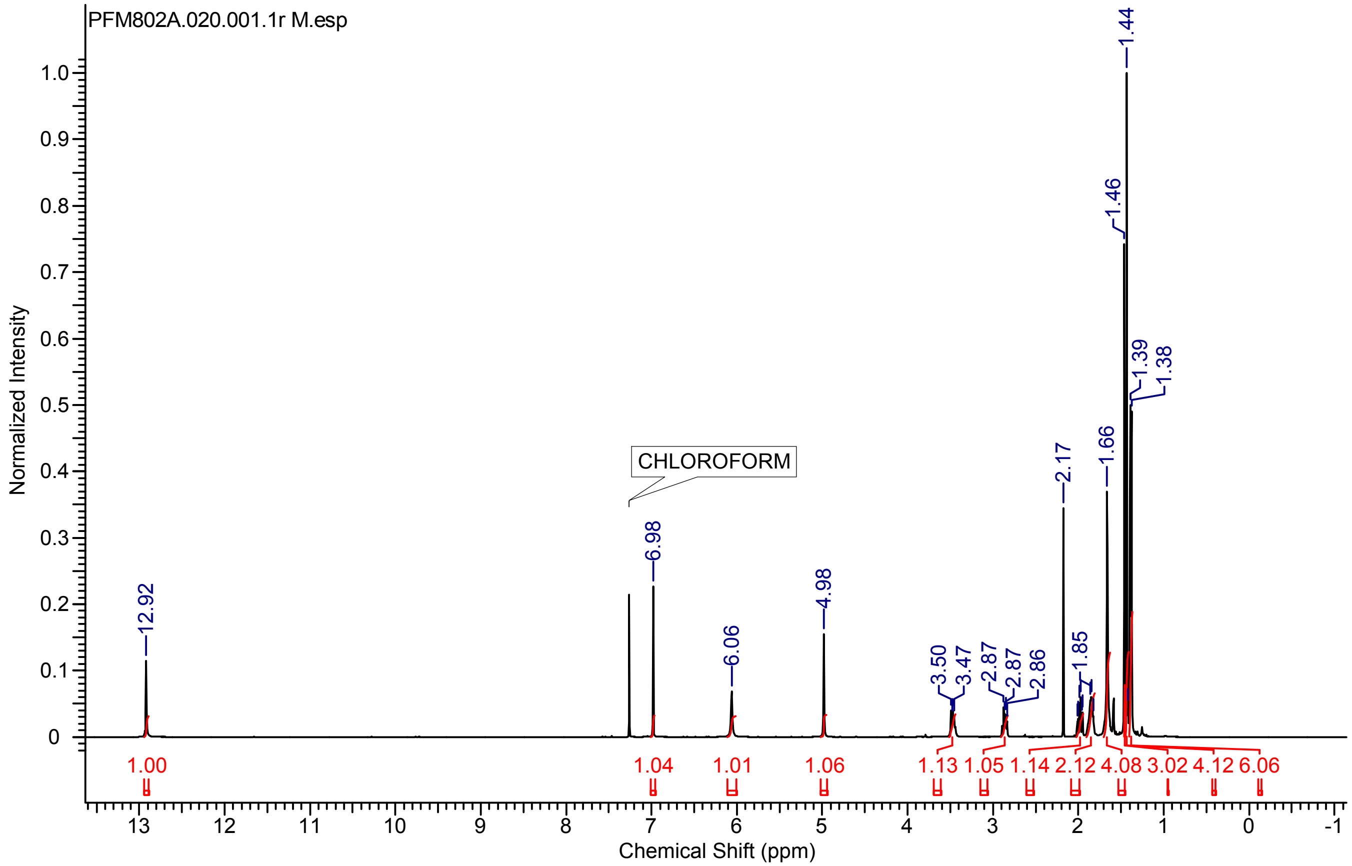

Figure S99. The ^{1}H NMR (500 MHz, $CDCl_3$) spectrum of compound **13**

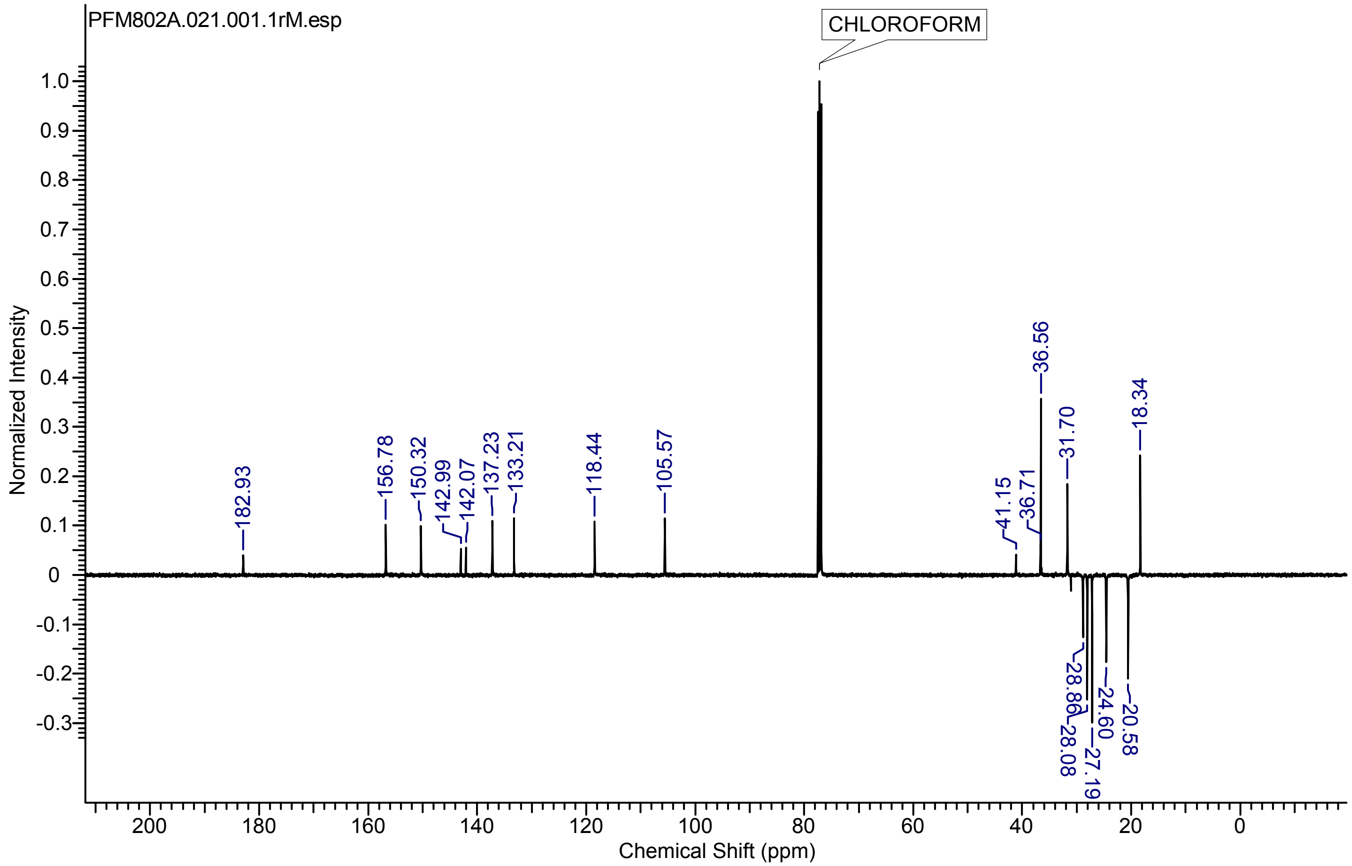

Figure S100. The ^{13}C NMR APT (125 MHz, $CDCl_3$) spectrum of compound **13**

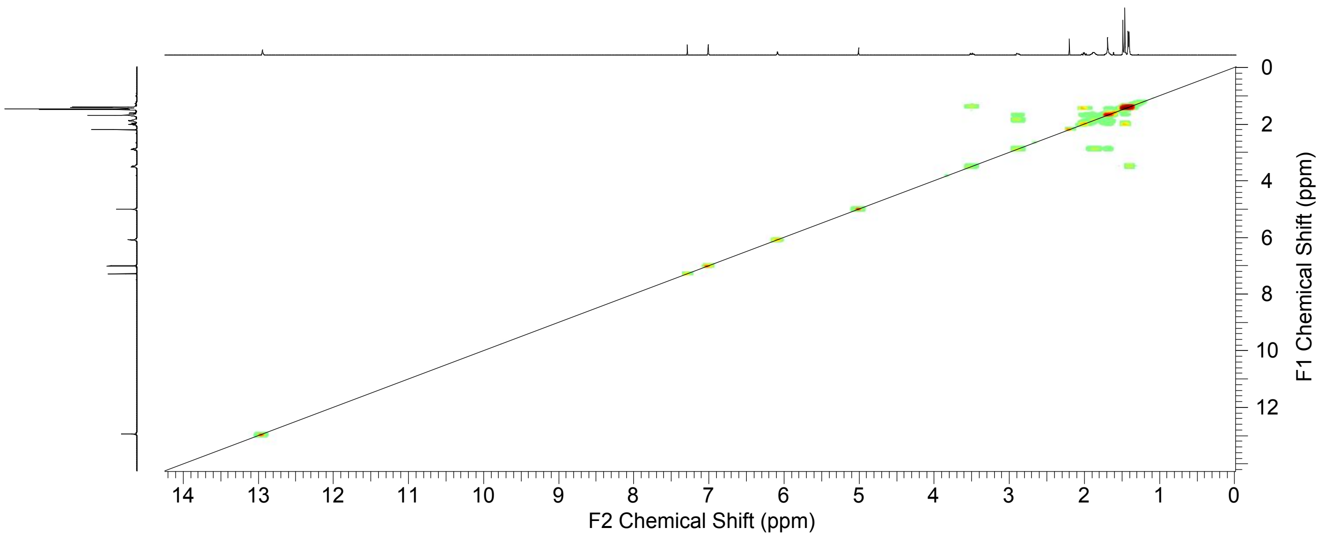

Figure S101. The COSY (500 MHz, $CDCl_3$) spectrum of compound **13**

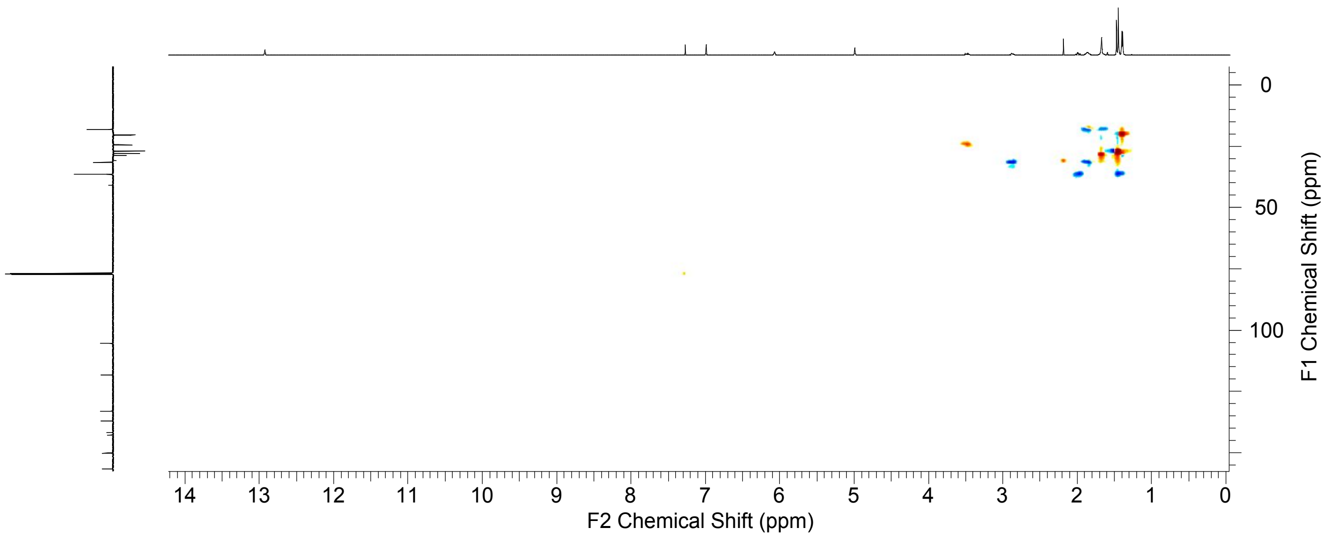

Figure S102. The HSQC (500/125 MHz, $CDCl_3$) spectrum of compound **13**

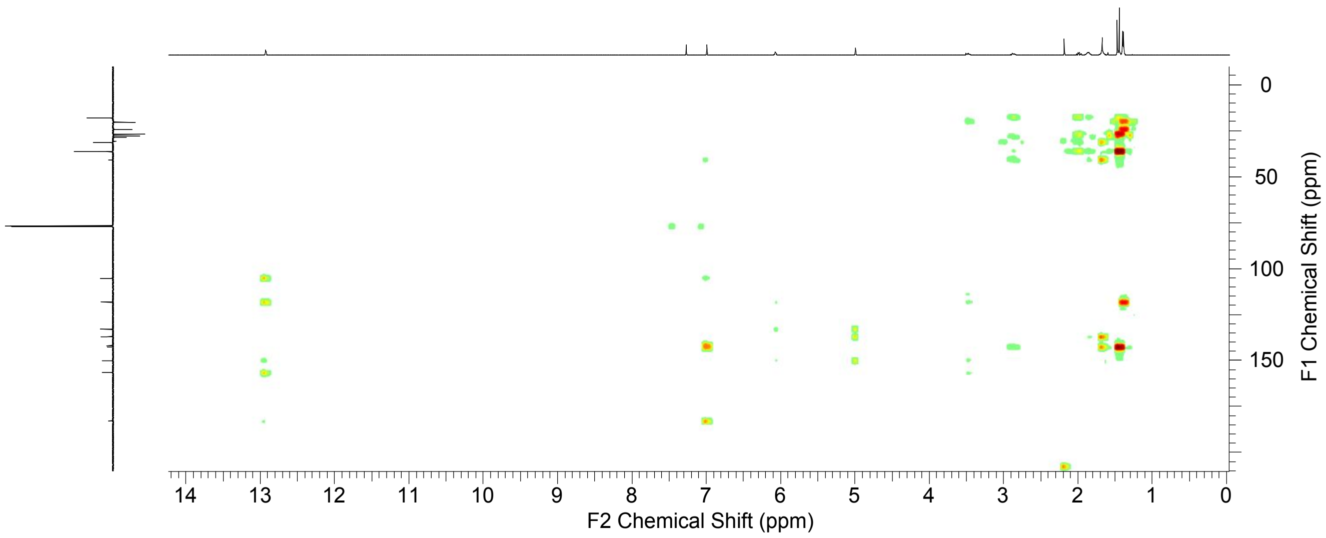

Figure S103. The HMBC (500/125 MHz, $CDCl_3$) spectrum of compound **13**

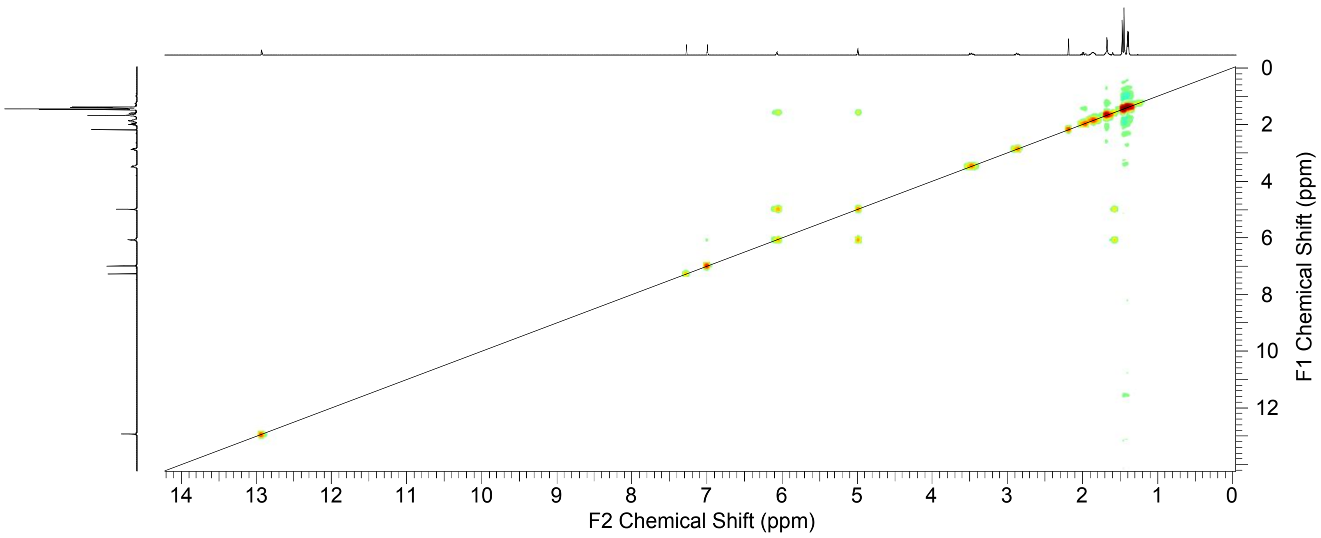

Figure S104. The NOESY (500 MHz, $CDCl_3$) spectrum of compound **13**

17. MS and NMR data for compound 14

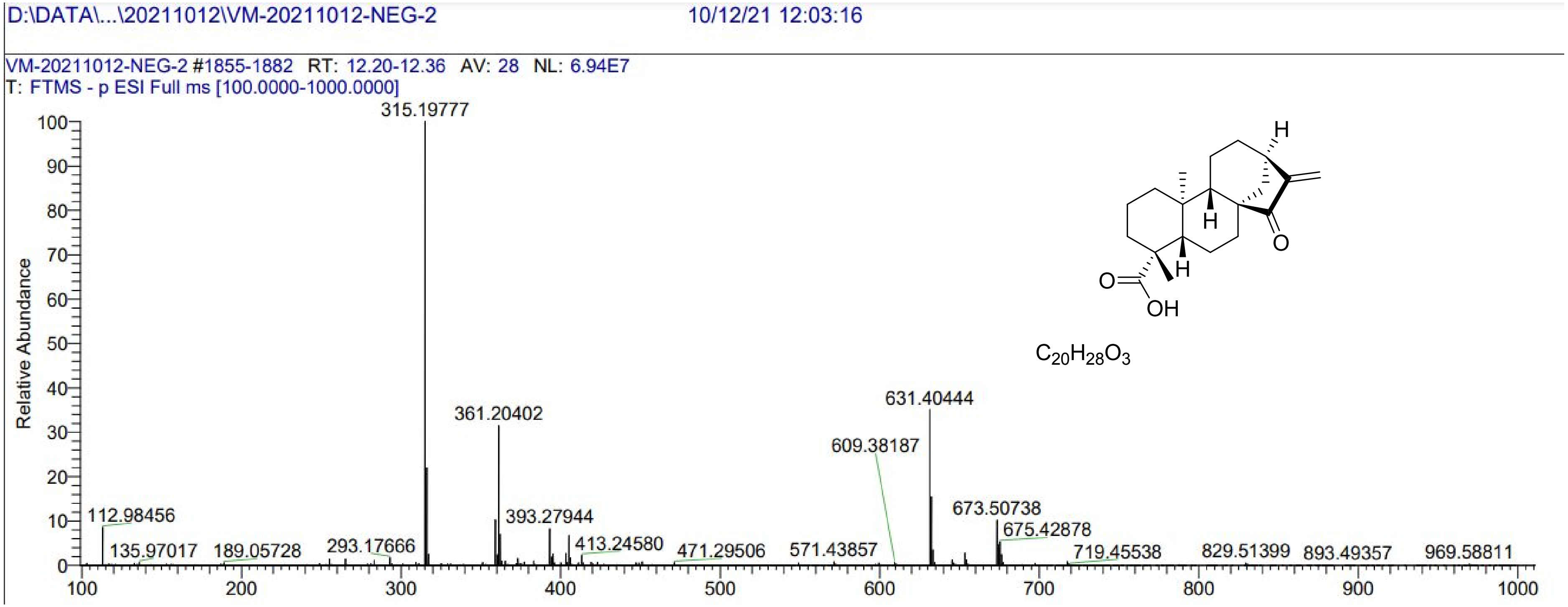

Figure S105. The HRESIMS spectrum of compound **14** (negative mode)

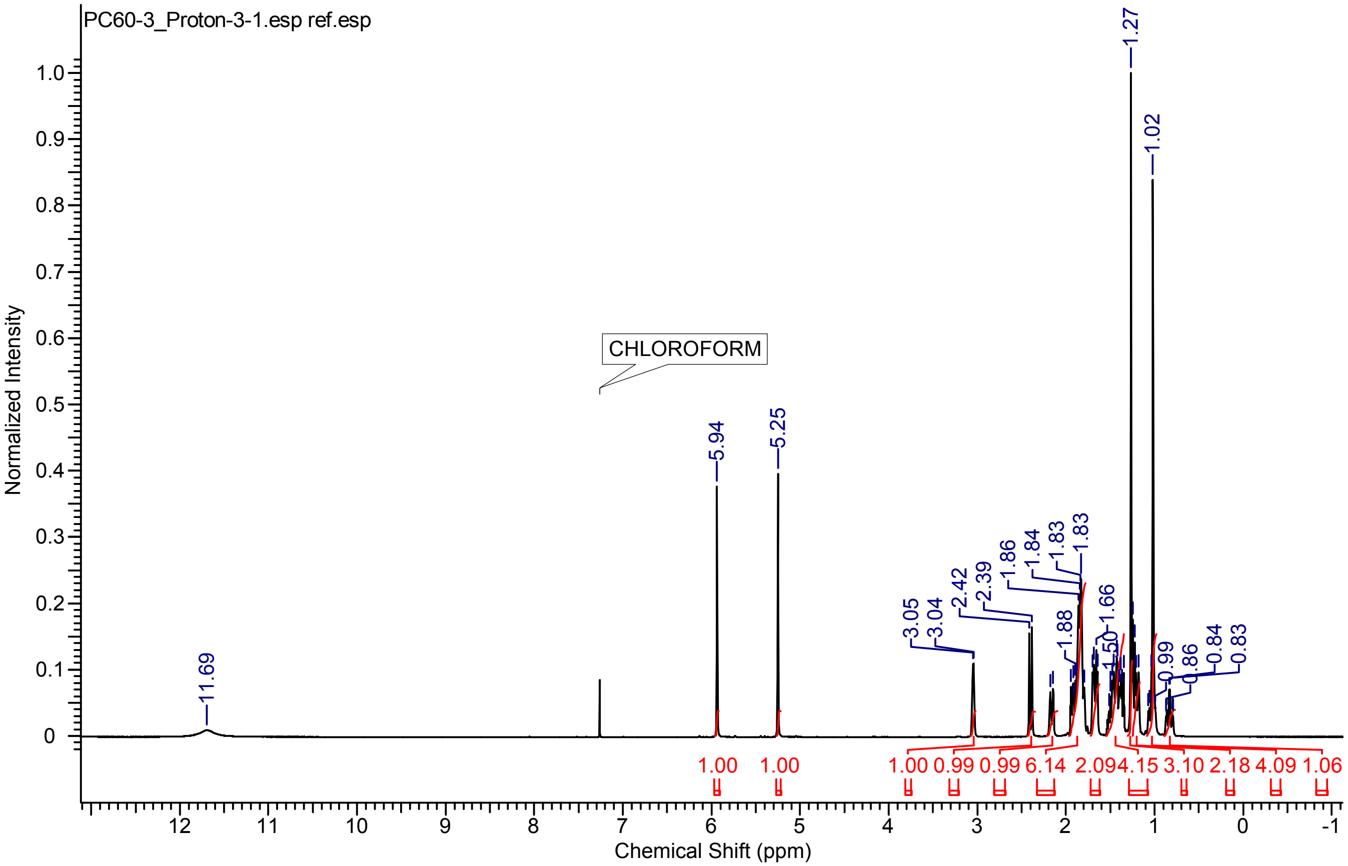

Figure S106. The ^{1}H NMR (400 MHz, $CDCl_3$) spectrum of compound **14**

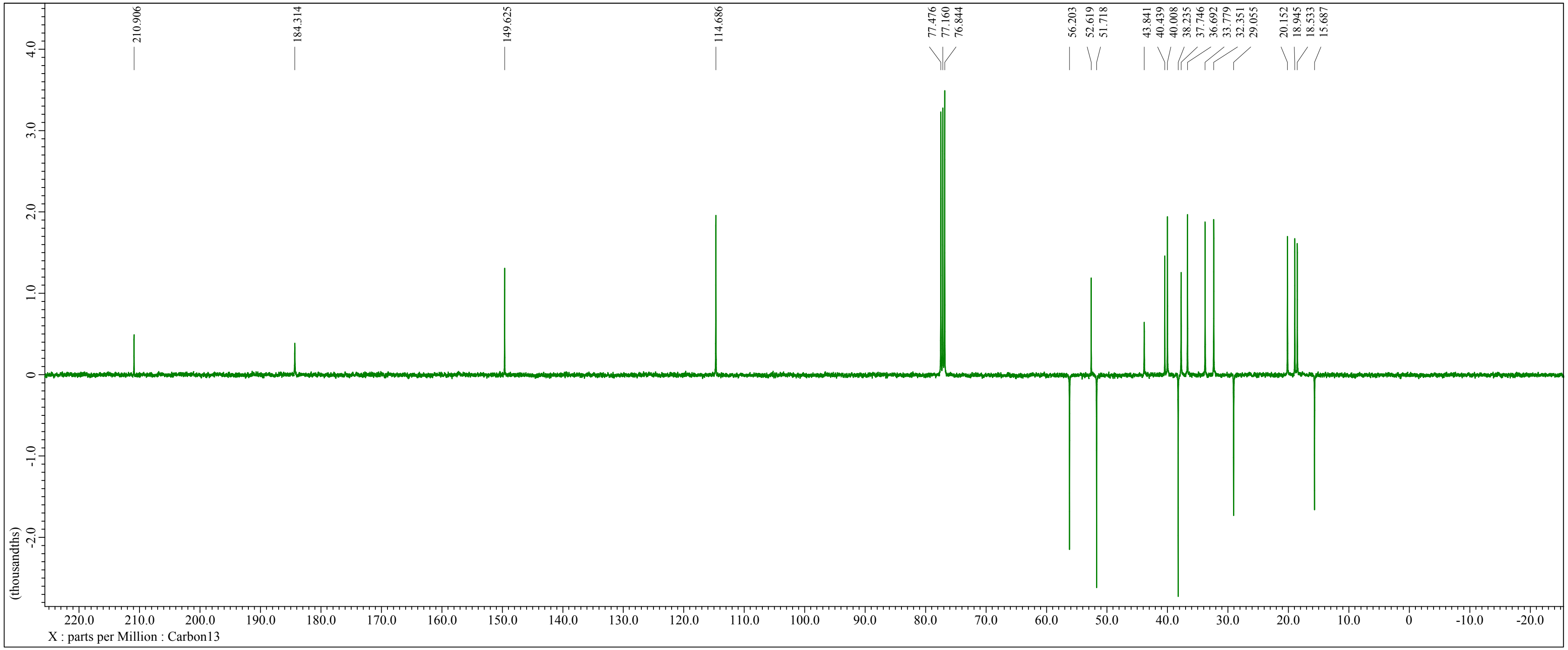

Figure S107. The ^{13}C NMR APT (100 MHz, $CDCl_3$) spectrum of compound **14**

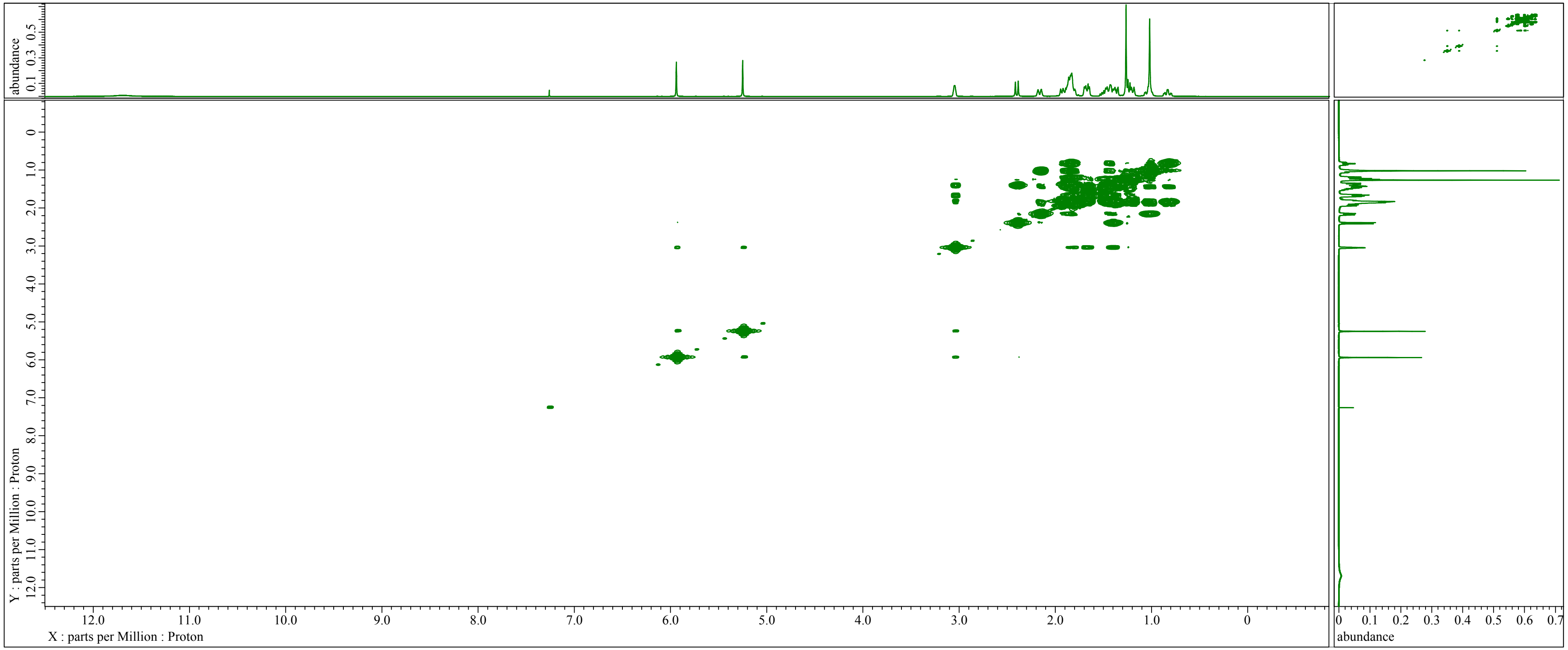

Figure S108. The COSY (400 MHz, $CDCl_3$) spectrum of compound **14**

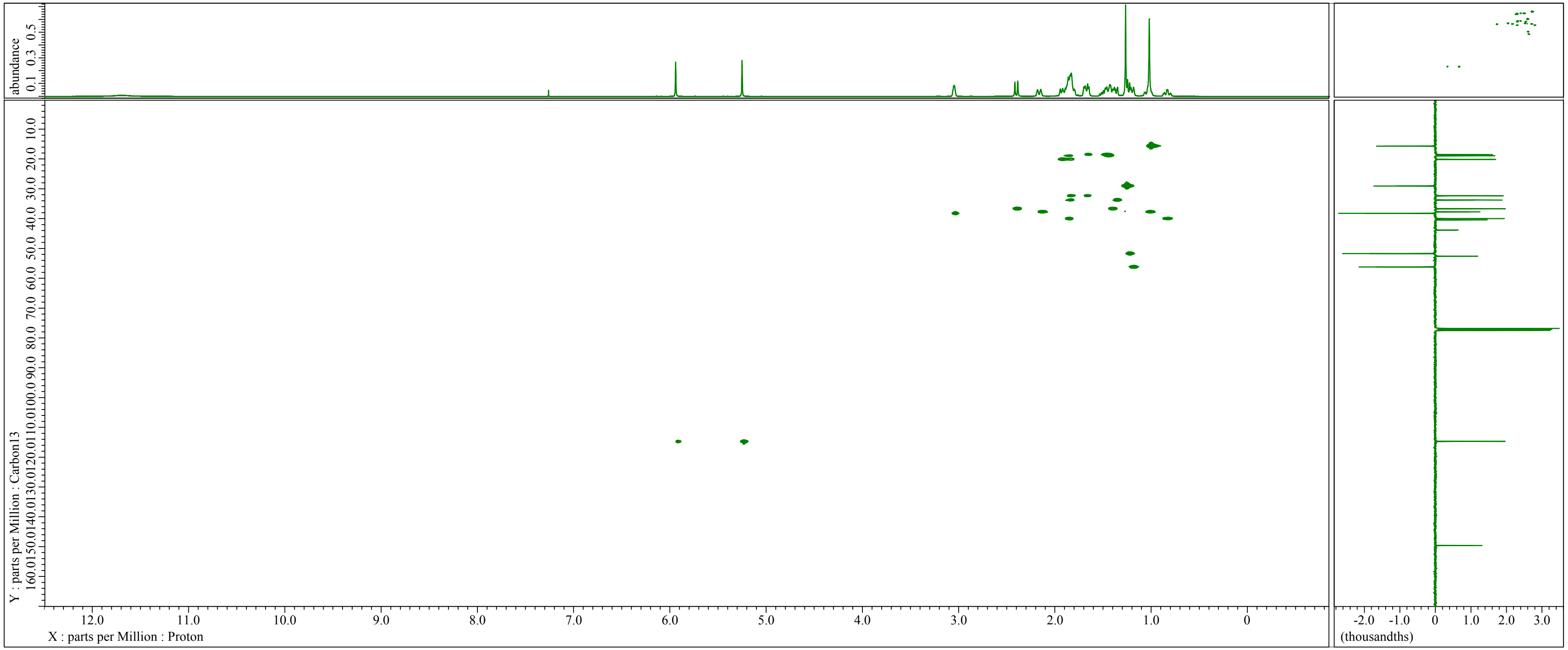

Figure S109. The HSQC (400/100 MHz, $CDCl_3$) spectrum of compound **14**

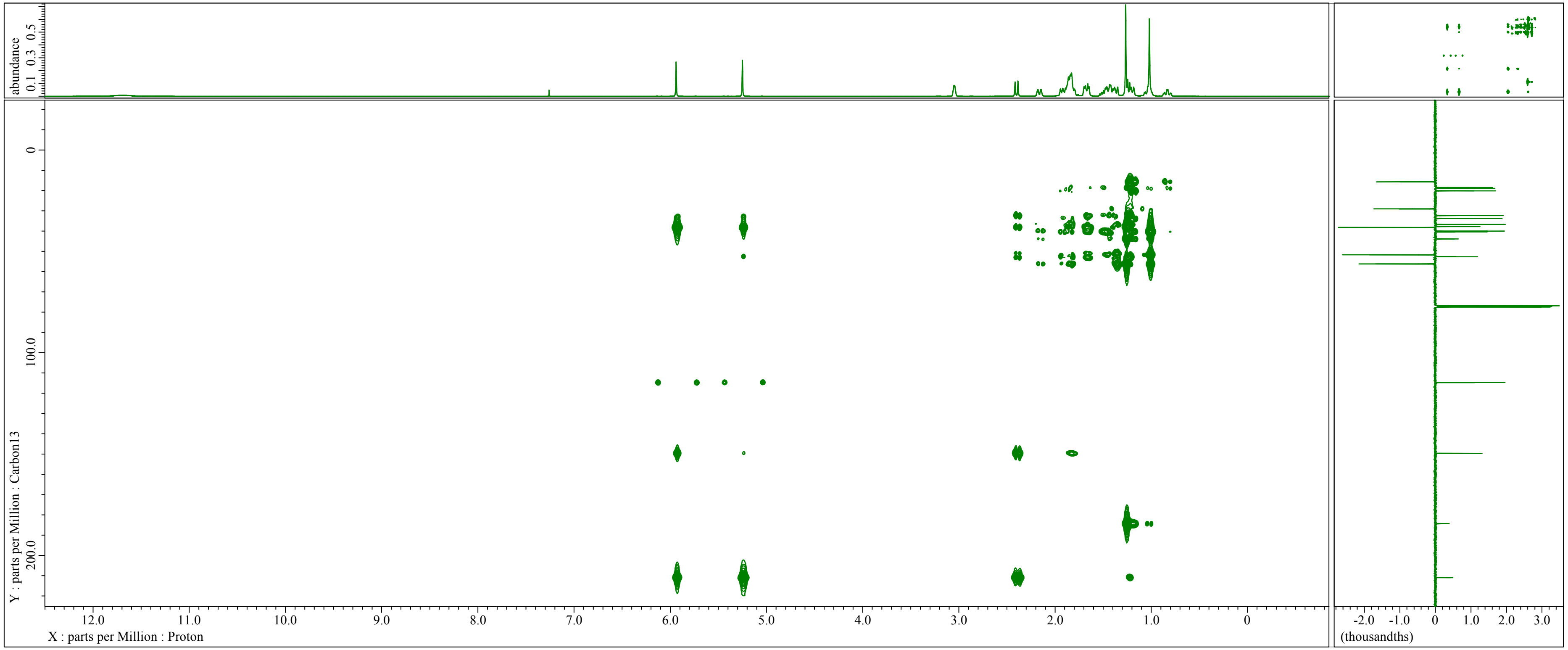

Figure S110. The HMBC (400/100 MHz, $CDCl_3$) spectrum of compound **14**

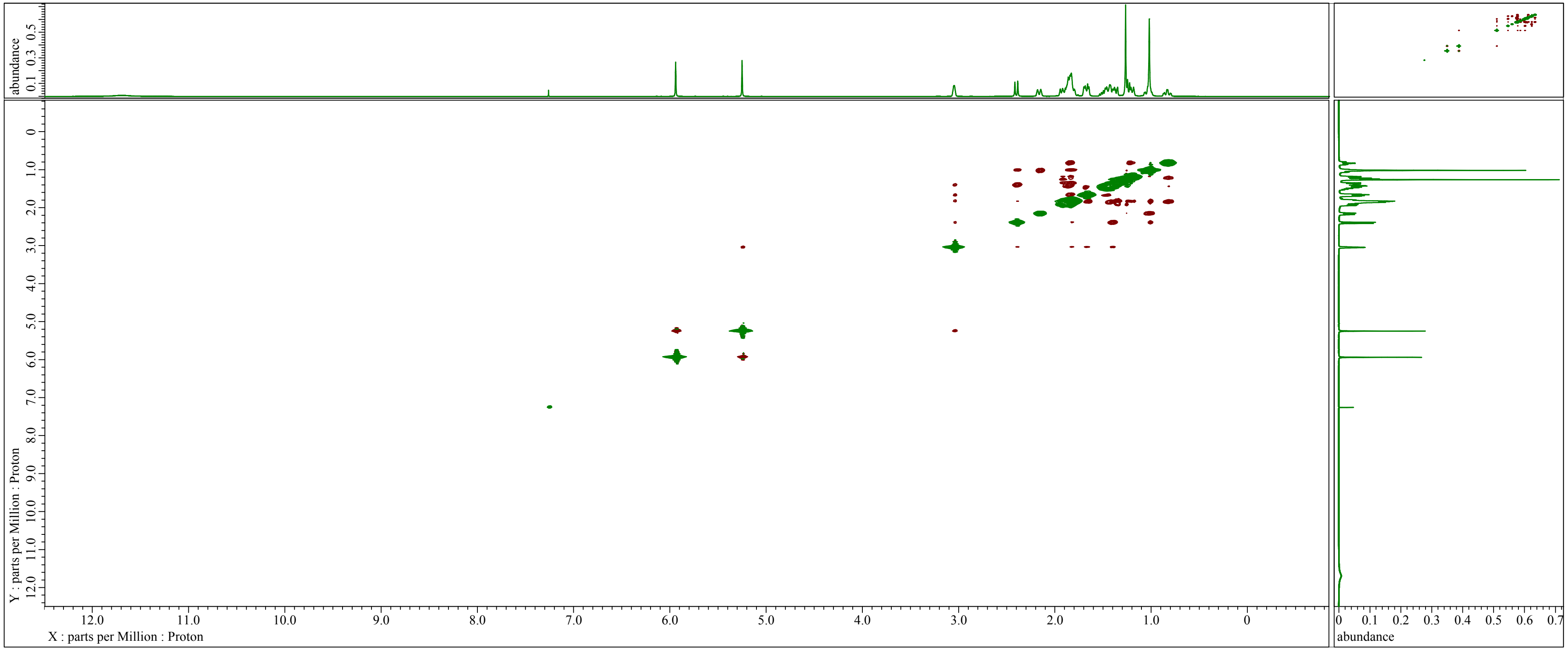

Figure S111. The NOESY (400 MHz, $CDCl_3$) spectrum of compound **14**

18. MS and NMR data for compound 15

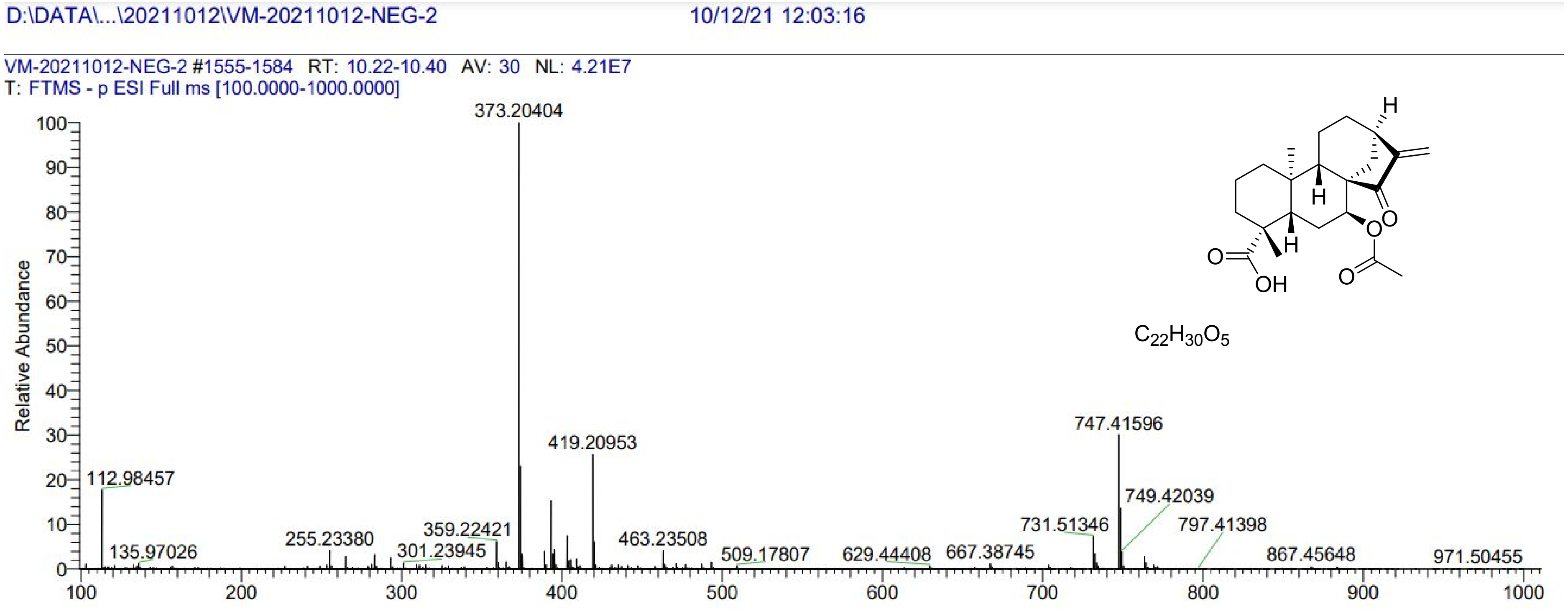

Figure S112. The HRESIMS spectrum of compound **15** (negative mode)

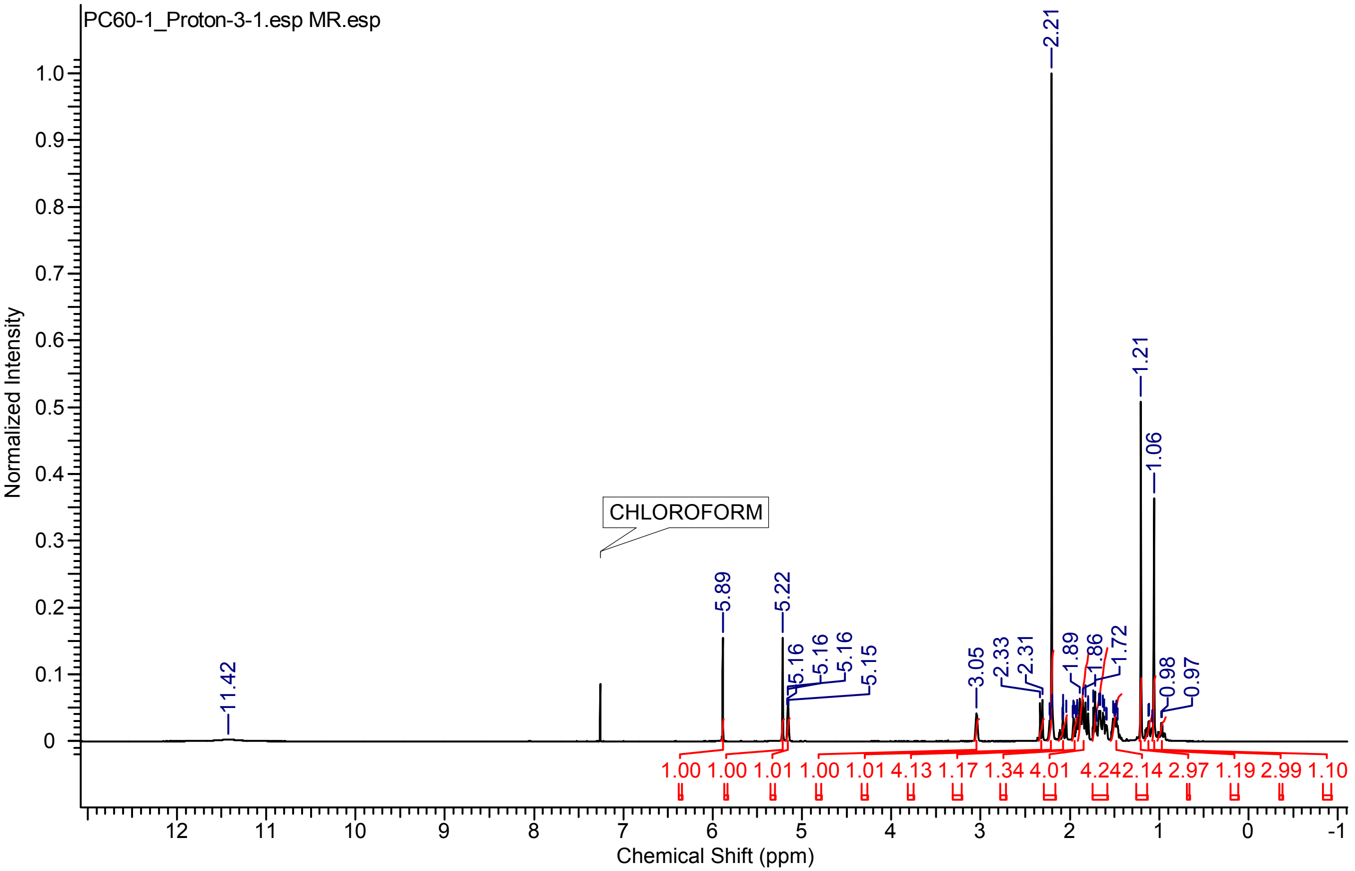

Figure S113. The ^{1}H NMR (400 MHz, $CDCl_3$) spectrum of compound **15**

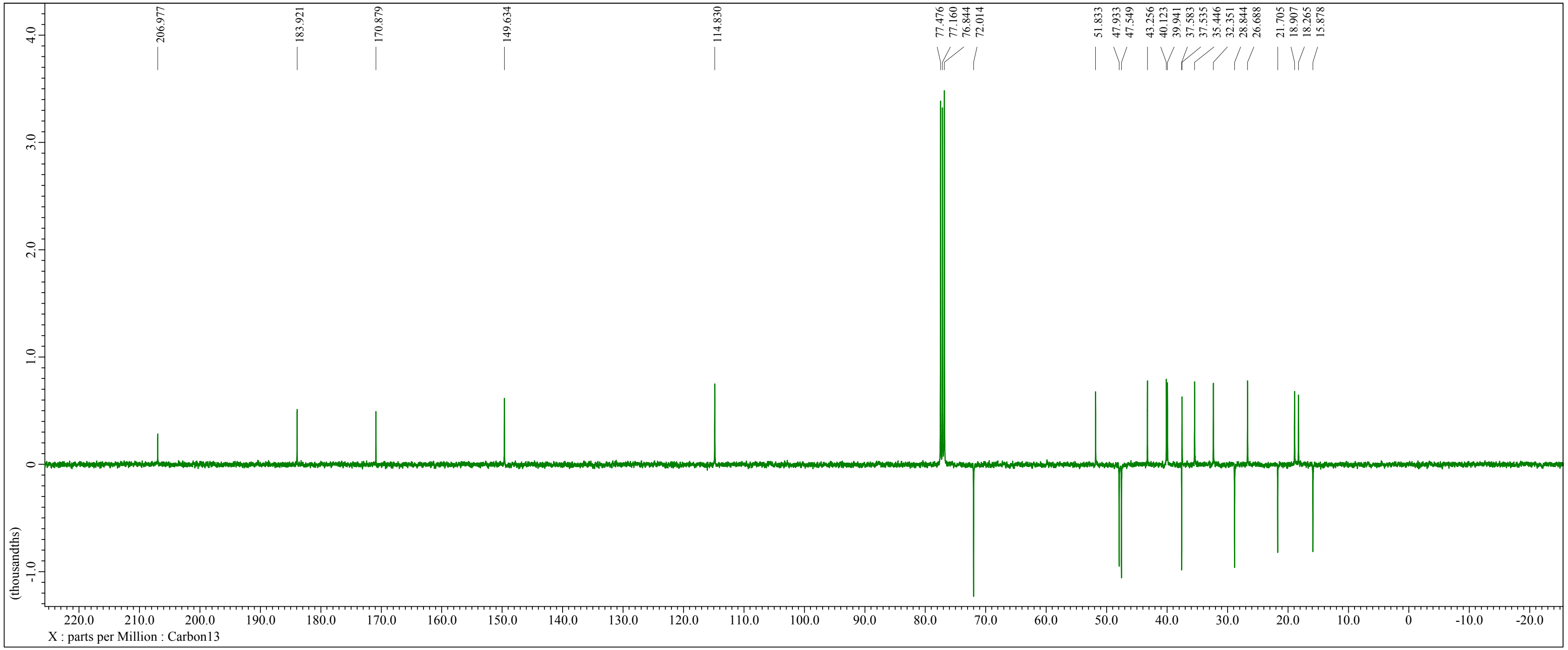

Figure S114. The ^{13}C NMR APT (100 MHz, $CDCl_3$) spectrum of compound **15**

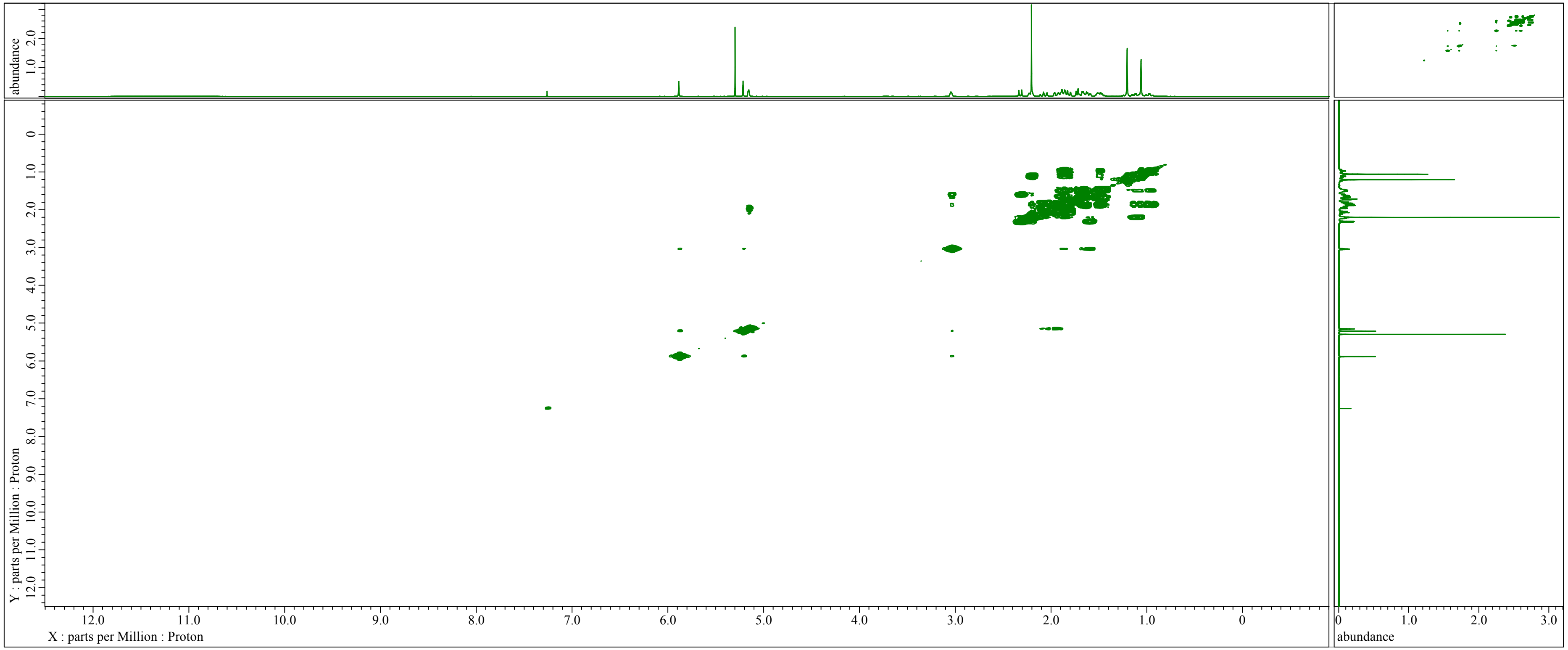

Figure S115. The COSY (400 MHz, $CDCl_3$) spectrum of compound **15**

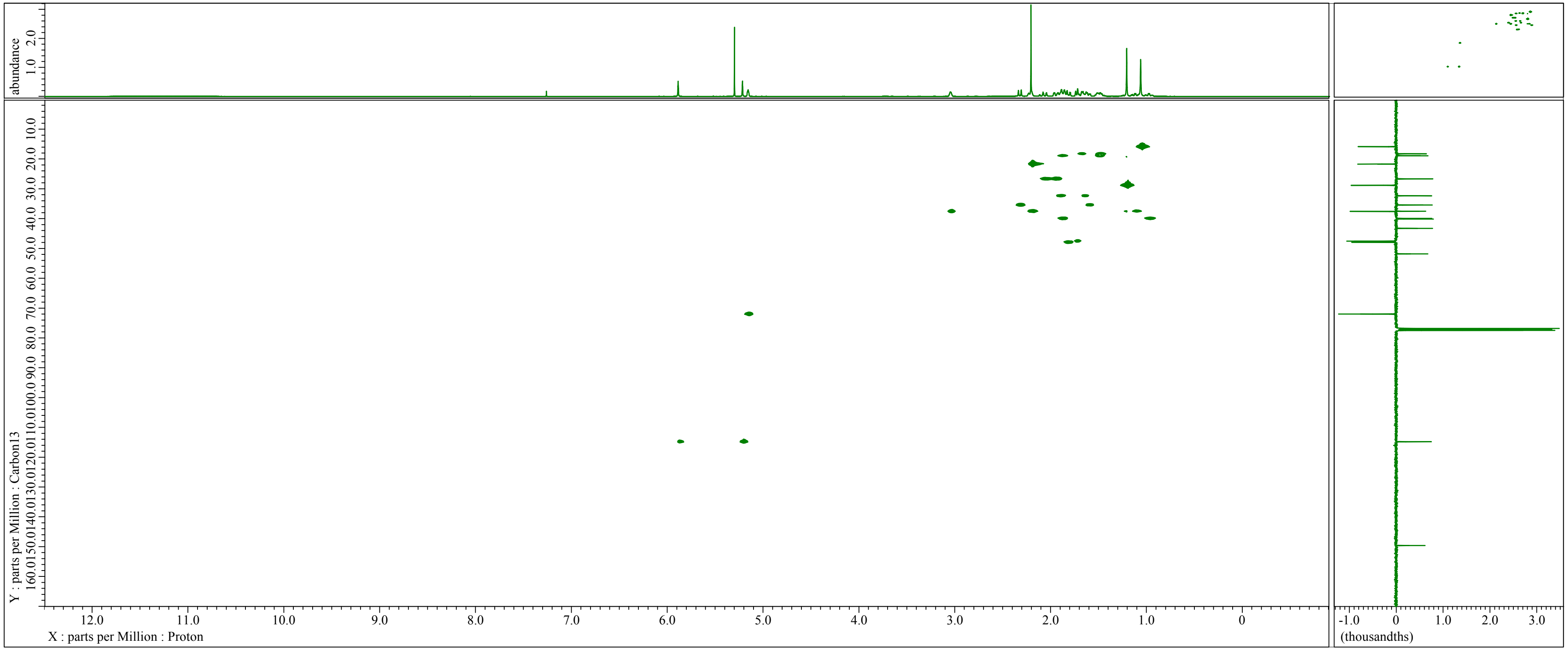

Figure S116. The HSQC (400/100 MHz, $CDCl_3$) spectrum of compound **15**

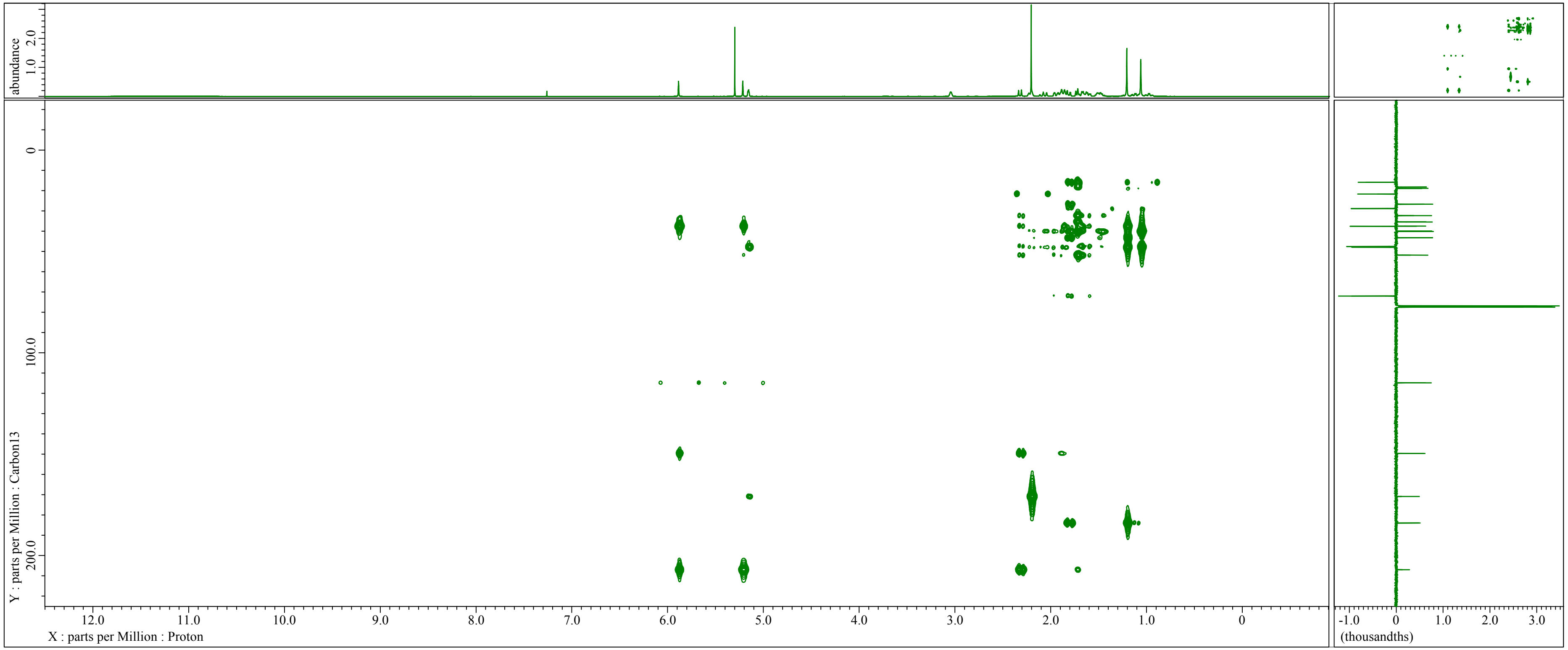

Figure S117. The HMBC (400/100 MHz, $CDCl_3$) spectrum of compound **15**

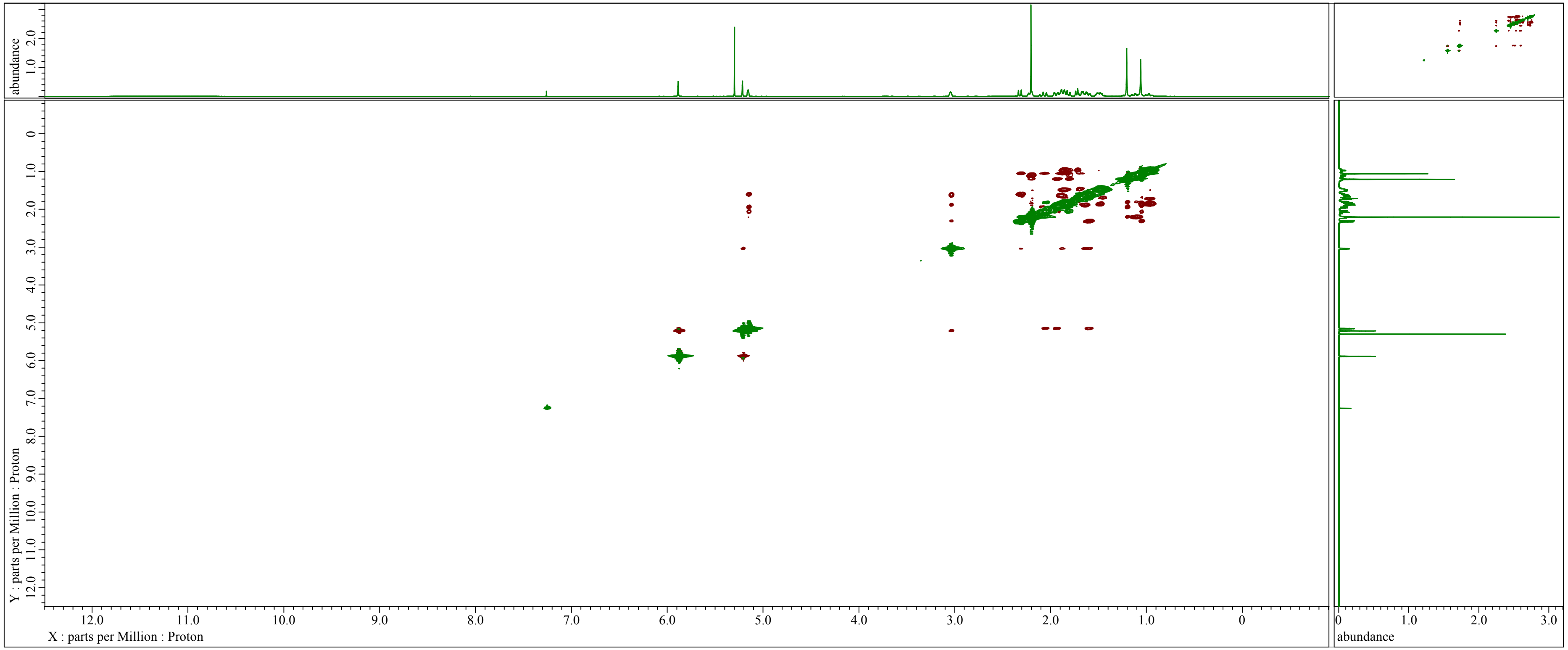

Figure S118. The NOESY (400 MHz, $CDCl_3$) spectrum of compound **15**
